# Chemical activation of prolyl hydroxylase-2 by BBAP-1 down regulates hypoxia inducible factor-1α and fatty acid synthase for mammary gland chemoprevention†

**DOI:** 10.1039/c8ra01239c

**Published:** 2018-04-04

**Authors:** Manjari Singh, Uma Devi, Subhadeep Roy, Pushpraj S. Gupta, Gaurav Kaithwas

**Affiliations:** Department of Pharmaceutical Sciences, Babasaheb Bhimrao Ambedkar University (A Central University) Vidya Vihar, Raebareli Road Lucknow-226025 UP India gauravpharm@hotmail.com gauravk@bbau.ac.in +91 9670204349; Department of Pharmaceutical Sciences, Faculty of Health and Medical Sciences, Sam Higginbottom University of Agricultural Sciences and Technology Naini Allahabad UP India

## Abstract

(4-[7-(Acetyloxy)-2-ethyl-2*H*-chromen-3-yl] phenyl acetate) (BBAP-1) was identified as a potential prolyl hydroxylase-2 activator and tested for this activity using the 2-oxoglutarate dependent *in vitro* assay. BBAP-1 was evaluated for its cytotoxic potential against ER + MCF-7 cells, and *N*-methyl-*N*-nitrosourea induced estrogen positive mammary gland carcinoma model. The effect of BBAP-1 on cellular morphology was evaluated using *in vitro* acridine orange/ethidium bromide and JC-1 staining. The morphological symptoms of apoptosis were evident after BBAP-1 treatment when studied through cell staining using acridine orange/ethidium bromide and JC-1 dye. Flow cytometric analysis revealed that BBAP-1 treatment arrested the cell cycle in the G2/M phase. *In vivo* study revealed the morphological changes of mammary gland tissue when scrutinized using carmine staining, hematoxylin and eosin staining and scanning electron microscopy. BBAP-1 treatment produced a marked effect on histopathological and morphological features when scrutinized against *N*-methyl-*N*-nitrosourea induced mammary gland carcinoma. Treatment with BBAP-1 also attenuated the deleterious effects of *N*-methyl-*N*-nitrosourea as measured on the basis of oxidative stress markers. Immunoblotting and qRT-PCR analysis revealed the participation of BBAP-1 in the mitochondrial mediated death apoptosis pathway and BBAP-1 also downregulated the hypoxic pathway through activation of prolyl hydroxylase-2. It was concluded that BBAP-1 activated the prolyl hydroxylase-2 enzyme and curtailed the over expression of hypoxia inducible factor-1α and fatty acid synthase along with the mitochondrial mediated death apoptosis pathway.

## Abstract 2

(4-[7-(Acetyloxy)-2-ethyl-2*H*-chromen-3-yl] phenyl acetate) (BBAP-1) was identified as a potential prolyl hydroxylase-2 activator and tested for this activity using the 2-oxoglutarate dependent *in vitro* assay.

## Graphical abstract

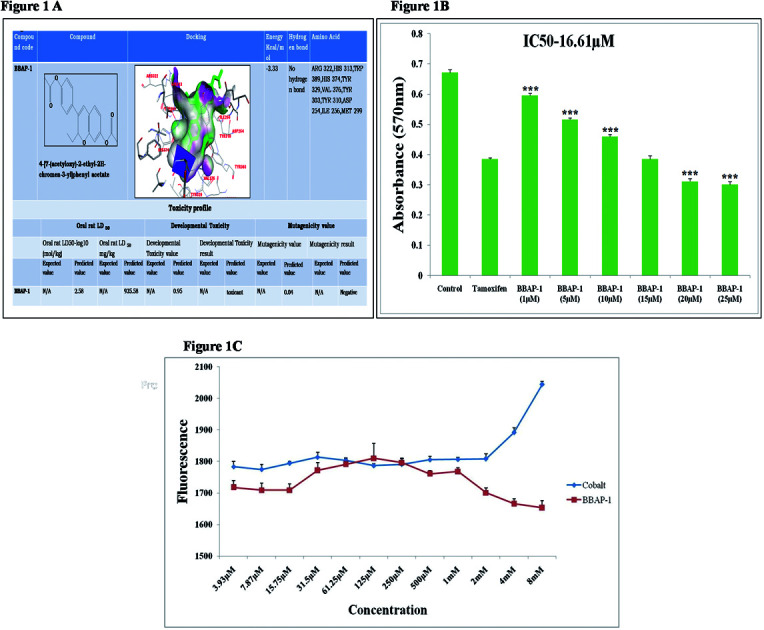

## Introduction

Tumor hypoxia is a condition where solid tumors have insufficient oxygen supply, leading to an imbalance in their consumption of oxygen. Thus, hypoxia alters the cellular energy production mechanism, with the anaerobic mode prevailing over aerobic respiration.^1^ In the hypoxic state, an imbalance is developed between the supply and consumption of oxygen.^2,3^ Importantly, hypoxia distress in cancer cells leads to an alteration in their metabolite signaling, consequently promoting glycolysis and lipid biogenesis (Warburg effect) over the tri-carboxylic acid (TCA) cycle and beta-oxidation.^4^

The hypoxic microenvironment triggers hypoxic signaling pathways in which hypoxia inducible factor (HIF) is the most clearly understood transcription factor.^5^ The structure of HIF is a basic helix-loop-helix PAS (Per, Arnt, Sim) domain, which consists of two subunits HIF-1α and HIF-1β.^6^ HIF-1α is found to be overexpressed in all types of cancer and enables tumor cells to grow under hypoxic conditions.^7^ It would be appropriate to note that increased glycolysis and lipogenic activity is one of the major methods by which the tumor cells meet their high energy and lipid requirements.^8^ In addition to the above, HIF-1α regulates the process of angiogenesis through binding with vascular endothelial growth factor (VEGF).^9^

During normoxia, HIF-1α goes through proteasomal degradation, which is regulated by prolyl hydroxylases (PHDs), followed by binding with von Hippel–Lindau (pVHL) suppresser protein.^10^ PHDs include the iron and 2-oxoglutarate (2-OG) dependent dioxygenase and are responsible for the hydroxylation of HIF-1α.^11^ In mammals, three isoforms of PHDs (PHD-1, PHD-2, and PHD-3) exist, each having different sub cellular localization, and PHD-2 is the most important protein in regulating HIF-1α.^12^ However, during hypoxia, PHD-2 remains dormant and HIF-1α escapes proteasomal degradation. Subsequently, HIF-1α translocates into the nucleus and forms a complex with HIF-1β which binds with hypoxia-responsive elements. Altogether, HIF-1α and PHD-2 regulate the process of energy production, angiogenesis and cellular proliferation in cancer cells.^13^

The association between PHD-2 deficiency and metastasis in pancreatic tumor cells has been reported earlier, which further strengthens its role in tumor progression.^14^ It was previously hypothesized that the chemical activation of PHD-2 can down-regulate the overexpressed level of HIF-1α and fatty acid synthase (FASN) in tumor cells.^15,16^ In fact, previous preliminary reports also suggested three activators of PHD-2, named KRH102053, KRH102140 and R59949, with anticancer potential.^17–19^ In consideration of the above and in search of potential activators of PHD-2 with anticancer activity, we considered it worth screening structural analogs of PHD-2 and validating their anticancer efficacy through *in vitro* and *in vivo* studies.

## Materials and methods

### Drugs and chemicals

4-[7-(Acetyloxy)-2-ethyl-2*H*-chromen-3-yl] phenyl acetate (BBAP-1) was received as a generous gift from National Cancer Institute, National Institutes of Health, USA (export reference: SL#201500077, job number-0348). Other materials included RPMI 1640 medium (Gibco-11875093); l-glutamine (Gibco-25030081); fetal bovine serum (FBS) (Gibco-10270); trypsin (Gibco-R001100); eagle's balanced salt solution (EBSS) (Gibco-2018-11); hank's balanced salt solution (HBSS) (Himedia-TL1190); ethidium bromide (EtBr) (Himedia-MB071-1G); acridine orange (AO) (Himedia-MB116-10G); propidium iodide (PI) (SC-3541); JC-1 assay kit (Thermo Scientific M34152); tamoxifen citrate (Biochem Pharmaceuticals); penicillin-streptomycin (Thermo Scientific, 15410-163); gentamycin (Thermo Scientific, 15710-049); 3-(4,5-dimethyl-2-thiazolyl)-2,5-diphenyl-2*H*-tetrazolium bromide (MTT) (TC191-1G); RNase (SRL, 9001-99-4); dimethyl sulfoxide (DMSO) (Merck, 1.16743.0521); ponceau S (Himedia-ML045); sodium cacodylate (Sigma-Aldrich, C0250); collagenase type IV (TC214); hyaluronidase (TC331); hematoxylin (Himedia-S058); eosin (Himedia-S007); RIPA lysis buffer (Amresco, N653); protein assay kit (Amresco, M173); bovine serum albumin (BSA) (Genetix, PG-2330); transfer buffer (Genetix, GX-9411AR); *N*-methyl-*N*-nitrosourea (MNU) (Sigma-Aldrich, N1517). Caspase 3 (SC-4263) and caspase 8 (SC-4267) assay kits were procured from Santa Cruz Biotechnology Inc., California. All other chemicals were of molecular grade and purchased from Genetix Biotech Asia Pvt. Ltd., New Delhi, India unless otherwise stated within the text.

### Screening of compounds

The compounds were screened based on 50% structural similarity with the previously reported PHD-2 activator KRH102140 ^18^ from the zinc database. The compounds were screened for their drug likeness (ADME, Lipinski, MDDR and CMC) using the DruLiTo tool.^20^

### C-DRUG web server

Approximately 5055 compounds were retrieved from the structural similarity and drug likeness search. The compounds were further screened for the possibility of anticancer activity, using the C-DRUG web server. The C-DRUG server predicts the anticancer potential of a compound *in silico* by using the molecular description method (relative frequency–weight fingerprint). The results are represented by a set of variables, including query compound(s), matched compound(s), average GI50 value of the matched compound(s), maximum *H*-score and *P*-value. C-DRUG categorizes the results as most promising (green), possible (black), and less likely (gray) molecules depending on the *P*-value. C-DRUG screening retrieved BBAP-1 as a highly possible anti-cancer compound.^21^

### Docking and toxicity study

The highly possible anti-cancer compound BBAP-1 obtained through the C-DRUG server was docked with the PHD-2 enzyme (PDB id-2G19) using Autodock 4.2.^22^ The toxicity profile was estimated through the toxicity estimation software tool (TEST).^23^ TEST is a simple QSAR modeling tool to calculate the toxicity of test molecules.^24^

### Metabolic profiling of BBAP-1

The metabolic profile of BBAP-1 was evaluated using Metaprint2D,^25^ licensed under the Apache license, 2.0. BBAP-1 was further screened for the type of isoform of CYP450 involved in its metabolism using the WhichCyp web server.^26^

### *In vitro* PHD-2 assay

Serum samples were collected from control animals, pooled and homogenized with five volumes of 0.25 M sucrose containing HEPES buffer (50 mM, pH 7.0). The homogenizing medium also contained PMSF as an anti-proteolytic agent (50 μg ml^−1^), 10^−4^ M dithiothreitol (DTT), 10^−5^ M EDTA and 0.1% Triton X-100. Homogenates were centrifuged at 14 000 rpm for 10 min and supernatants were collected. To 100 μl of the supernatant, 990 μl of PHD-2 activation buffer [sodium ascorbate (1 mM); proline (1 mM); BSA (0.2%); catalase (0.04%); α-ketoglutarate (0.1 mM)] was added and incubated at 30 °C for 3 h. After incubation, 100 μl HCl (0.5 M) was added to stop the reaction, followed by 50 μl of *o*-phenylenediamine (OPD) (10 mg ml^−1^ prepared in 0.5 M HCl). The reaction mixture was heated at 95 °C for 10 min followed by centrifugation at 10 000 rpm for 5 min to collect the supernatant. To 100 μl of the supernatant, 60 μl of 1.2 M sodium hydroxide was added and fluorescence was read at 340 nm excitation, 420 nm emission wavelengths. The assay was divided among three groups: control (serum); inhibitor [serum + cobalt (inhibitor)]; activator [serum + BBAP-1 (activator)]. The relative PHD-2 activities of the cobalt and BBAP-1 were calculated by considering the activity in the control samples as 100%.^27,28^

### Cell culture

ER + MCF-7 cells were cultured and routinely maintained in RPMI 1640 medium supplemented with 10% heat-inactivated FBS, penicillin (100 units per ml), streptomycin (100 μg ml^−1^), and gentamycin (0.25 μg ml^−1^) and incubated at 37 °C in a humidified atmosphere containing 5% CO_2_ inside a CO_2_ incubator. The cells were counted using a trypan blue exclusion method in hemocytometer.^29^

### Cell viability assay

ER + MCF-7 cells (1 × 10^5^) were seeded in 96-well sterile plates and treated with different concentrations of BBAP-1 (1 μM, 5 μM, 10 μM, 15 μM, 20 μM, 25 μM) for 24 h against control and standard drug (tamoxifen). After 24 h of incubation at 37 °C in an atmosphere of 5% CO_2_ and 95% humidity, the apical and basolateral media were pooled on a 24-well plate and the cell viability was estimated by MTT assay through measuring the emitted fluorescence with a spectrofluorometric multi plate reader (excitation-555 nm, emission-585 nm). The percentage of cell viability for each sample was calculated compared to non-exposed cells (corresponding to 100% viability).^30^

### Fluorescence microscopic analysis of cell death using AO/EtBr dual staining

AO/EtBr dual staining was performed to identify the morphological symptoms of apoptosis in ER + MCF-7 cells. After 24 h cell attachment, the cells were treated with the IC50 dose of BBAP-1 for the duration of 1 day. The cells were washed with phosphate buffered saline (PBS) (pH 7.4) and fixed for 15 min at room temperature in the dark with a BD cytofix/cytoperm assay kit. After that, the samples were processed with 10 μl of AO/EtBr cocktail (100 μg ml^−1^ of AO and 100 μg ml^−1^ of EtBr) and made up to 100 μl using PBS and incubated for 5 min. The cells were later washed with PBS and treated with background suppressor for the respective green and red channels to decrease the signal-to-noise ratio and non-specific labeling, then air dried and examined under an inverted fluorescence microscope with gold anti-fade mountant in coverslips (Nikon Leica M165 FC).^31^

### Measurement of mitochondrial membrane potential (Δ*ψ*) using JC-1 staining

JC-1 is a cationic potential-dependent J-aggregate forming dye used to determine the loss of mitochondrial membrane potential (indicative of apoptosis). For the experimental schedule, ER + MCF-7 cells were seeded at a density of 2 × 10^5^ cells per well in a 75 cm^2^ culture flask and incubated overnight for cellular attachment. The cells were then treated with the IC50 dose of BBAP-1. The untreated control cells were included. After 24 h, 100 μl of JC-1 staining solution (1 : 10 dilution) was added into each 1 ml of complete RPMI 1640 medium in a 6-well multiplate and incubated for 15 min at 37 °C in a CO_2_ incubator. The cells were later washed with PBS three times and treated with background suppressor for the red channel. Transition of fluorescence (red to green) under different experimental conditions was observed in the TRITC channel under an inverted fluorescence microscope (Nikon Leica M165 FC).^32^

### Flow cytometry analysis of cell cycle and apoptosis using PI staining

The ER + MCF-7 cells were seeded in 6-well plates at a density of 2 × 10^5^ cells per well. Then, the cells were treated with the IC50 dose of BBAP-1. After 24 h, the cells were trypsinized, washed with PBS and collected. The resulting pellet was fixed in ice chilled methanol and stored at −20 °C for 5 min. The fixed cells were washed with PBS and treated with RNase (add 50 μl of a 100 μg ml^−1^ sock of RNase) which ensure RNA free positive DNA solution and Triton-X (0.1%) and then stained with 200 μl PI (from 50 μg ml^−1^ stock solution). followed by 15 min dark incubation. The cell cycle phase distribution of nuclear DNA was determined by adjusting forward scatter (FS) and side scatter (SS) to identify single cells and pulse processing is used to exclude cell doublets from the analysis. A fluorescence activated cell sorter (FACS), using a fluorescence detector equipped with a 488 nm argon laser light source and 623 nm bandpass filter (linear scale) using BD FACS software 1.2.0.87 (BD Influx cell sorter, USA) with a suitable bandpass filter.^33^

### *In vivo* study

Female albino Wistar rats (100–120 g) were procured from the central animal house facility and acclimatized for a period of two weeks prior to the experiment. Animals were housed in polypropylene cages under controlled conditions (23 °C, 12 h light/dark cycle) with free access to standard pellet diet and water *ad libitum*. Animals were randomized and divided among four groups of eight animals each. Group I, served as normal control, received 0.9% normal saline 1 ml kg^−1^, p.o.; group II, served as toxic control, received MNU 8 mg kg^−1^, i.v.; group III and group IV, the drug treated groups, received BBAP-1 (61.55 μg kg^−1^, s.c.) + MNU (8 mg kg^−1^, i.v.) and BBAP-1 (123.11 μg kg^−1^, s.c.) + MNU (8 mg kg^−1^, i.v.) respectively. The animal dose of BBAP-1 was calculated using the IC50 dose of BBAP-1 and tamoxifen.^34^ The IC50 and rat dose of tamoxifen was taken as 27 μM and 200 μg per rat per day respectively, as reported in previous literature.^35^ Toxicity was induced by single tail vein injection of MNU on the 1st and 21st day followed by daily administration of BBAP-1 up to the 38th day of the study. All the animal experiments were performed in compliance with CPCSEA guidelines for laboratory animals and ethics, Department of Animal Welfare, Government of India. Ethical approval for the use of animals was received from Sam Higginbottom University of Agricultural Sciences and Technology, Naini, Allahabad (IAEC/SHIATS/PA16III/SUDPG01).

### Morphological evaluation of mammary gland tissue

#### Whole mount carmine staining of mammary gland tissue

Mammary gland tissues were kept overnight and stained with carmine alum solution as described previously.^36^ Stained whole mounts were examined using a 10× optical in biological microscope. The mammary gland whole mounts were evaluated for the presence of alveolar buds (ABs) (both type 1 and type 2), lobules and differentiation (DF) score. The evaluation was performed according to the principles described elsewhere.^36,37^

#### Histological analysis of mammary gland tissue

Mammary gland whole mounts were manually embedded in paraffin wax and cut into 5 μm sections for histological analysis. Mammary gland sections were stained with hematoxylin and eosin (H&E) for the examination of the basic histopathology of the tissue. The tissues were evaluated for the presence of cellular structures like cuboidal epithelial cells (CECs), myoepithelial cells (MECs), lymphocytes, ducts, adipocytes, loose connective tissue (LCT) and dense connective tissue (DCT).^32,38^

#### Scanning electron microscopy (SEM) of mammary gland tissue

SEM analysis was performed according to the method elaborated by us previously.^32^ The tissues were treated with 100 μl ml^−1^ collagenase (type 4) and 2.5 turbidity reduction unit (TRU)/ml of hyaluronidase. The tissues were fixed with glutaraldehyde and post fixation was done using osmium tetraoxide. After HCl digestion, all the tissue samples were dried by the critical point method and examined under SEM (JEOL JSM-6490LV).^39^

### Biochemical evaluation

The mammary gland tissues (10% w/v) were homogenized in 0.15 M KCl and centrifuged at 10 000 rpm. The supernatants were scrutinized for various antioxidant markers including thiobarbituric acid reactive substances (TBARs), superoxide dismutase (SOD), catalase, glutathione (GSH) and protein carbonyl (PC) using the method established at our laboratory.^40^

### Evaluation of caspase 3 and caspase 8

The activity of caspase 3 and caspase 8 was evaluated using the commercial kits. The DEVD-AFC and IETD-AFC synthetic substrates were used for the estimation of caspase 3 and caspase 8 respectively. Equal volumes of serum sample from control, toxic and drug treated groups were diluted with reaction buffer and DTT was added to a final concentration of 10 mM. To the reaction mixture 5 μl of IETD-AFC/DEVD-AFC substrate was added and incubated for 1 h at 37 °C. Free AFC levels formed were measured with the plate reader at 400 nm excitation and 505 nm emission. The results of experimental samples were compared with the control and expressed as fluorescence units per mg of protein.^41^

### Western blotting

Protein samples were prepared from the mammary gland tissue through acetone precipitation and quantified using the Bradford reagent.^42^ The immunoblotting analysis of Bcl-xl (MA-5-15142), Bcl-2 (SC-7382), BAX (SC-23959), VDAC (SC-390996), cytochrome c (SC-13561), Apaf-1 (SC-65891), procaspase 9 (SC-73548), NFκBp65 (MA5-1616), UCHL-1 (MA1-83428), PHD-2 (SC-67030), HIF-1α (SC-13515), FASN (SC-55580) and SREBP-1c (SC-13551) was performed according to the method elaborated elsewhere.^43^ β-actin (MA5-15739-HRP) was used as a standard reference. The membrane was washed with TBST thrice and incubated with the corresponding anti-rabbit (SC-2030), anti-goat (SC-2020) and anti-mouse (31430, Pierce Thermo Scientific, USA) HRP conjugated secondary antibody (1 : 5000 dilutions) at room temperature for 3 h.

### qRT-PCR

The fold change analysis of various genes was performed according to the method elaborated by us previously.^32^ The specific sequences of the forward and reverse primers are specified in Table S1.†

### Statistical analysis

All data were presented as mean ± SD and analyzed by one-way ANOVA followed by Bonferroni test for identification of the possible significance between various groups. *p < 0.05, **p < 0.01, ***p < 0.001 were considered as statistically significant. Statistical analysis was performed using Graph Pad Prism software (5.02).

## Results

### BBAP-1 inhibits *in vitro* PDH-2 activity

#### *In silico* drug screening

**Fig. 1:**
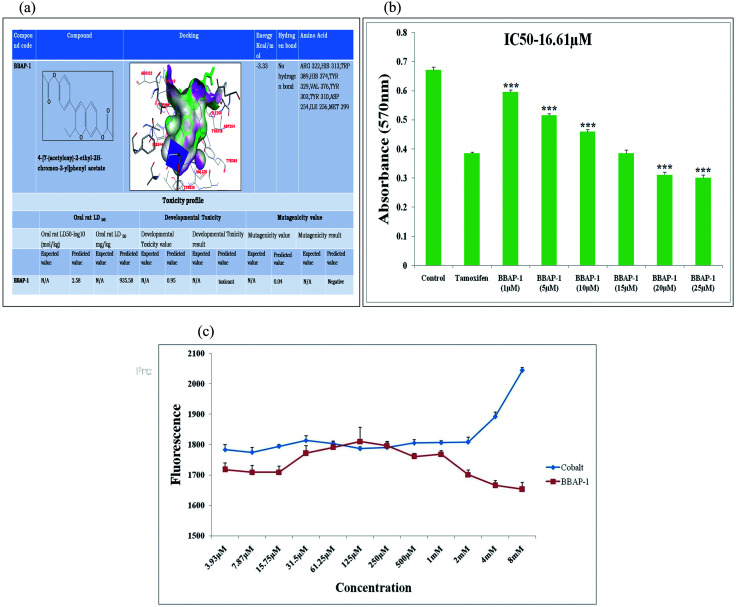
(A) *In silico* binding efficacy and toxicity profiling of BBAP-1: BBAP-1 was docked with PHD-2 protein (PDB id-2G19). Toxicity profile of BBAP-1 was estimated with TEST. (B) Effect of BBAP-1 upon MTT assay: the histogram revealed the effect of BBAP-1 on ER + MCF-7 cells by MTT assay. The cell growth was compared with the standard (tamoxifen). BBAP-1 displayed substantial cytotoxicity against ER + MCF-7 cells. Data is expressed as mean ± SD. The comparisons are made on the basis of one-way ANOVA followed by Bonferroni multiple test. *p < 0.05, **p < 0.01, ***p < 0.001 is considered statistically significant. Experiment was performed in triplicate. (C) Effect of BBAP-1 and cobalt upon *in vitro* PHD-2 activity: BBAP-1 demonstrates the *in vitro* activation of PHD-2. Cobalt was used as a negative control. The results are represented using control value as 100% PHD-2 activity. Values are represented as mean ± SD. Experiment was performed in triplicate.

PHD-2 modulators have not previously been explored to assess their impact on the mammary gland model of cancer. For this, we explored the zinc databases and retrieved 5055 potential hits using KRH102140 as the standard molecule (Table S2†). For the estimation of their anti-cancer property, the compounds were screened using the C-DRUG web server. BBAP-1 (4-[7-(acetyloxy)-2-ethyl-2*H*-chromen-3-yl] phenyl acetate) was retrieved with the highest possibility of showing anticancer activity. BBAP-1 was evaluated for its mutagenic and developmental toxicity potential through *in silico* TEST (Fig. 1A). The predicted oral LD50 value of BBAP-1 in rats was found to be 935.58 mg kg^−1^. BBAP-1 exhibited the binding energy of −3.33 kcal mol^−1^ when docked with PHD-2 (PDB id-2G19) using Autodock 4.2 (Fig. 1A). BBAP-1 significantly inhibited the growth of ER + MCF-7 cells in comparison to tamoxifen (a standard anticancer agent). The IC50 value of BBAP-1 was found to be 16.61 μM (Fig. 1B).

The metabolic positions of BBAP-1 were studied using the *in silico* tool Metaprint 2D which revealed that the 11th, 12th, 21st and 22nd carbon positions were major sites for metabolism through 12- and 22-dealkylation and 11- and 21-ester hydrolysis. CYP3A4 and CYP2D6 were the major cytochrome P450 enzymes involved in the metabolism of BBAP-1 when scrutinized through the WhichCyp web server (Fig. S1†).

#### 2-OG dependent *in vitro* PHD-2 assay

For the estimation of PHD-2 levels in biological serum samples, 2-OG dependent *in vitro* PHD-2 evaluation was performed. The fluorescence intensity is directly correlated with the amount of 2-OG remaining in the reaction mixture. A higher amount of 2-OG (higher fluorescence intensity) reflects lower PHD-2 activity. BBAP-1 showed a marked decrease in fluorescence intensity with the decrease in 2-OG concentration, suggesting increased PHD-2 activity. Increasing the concentration of cobalt (a known inhibitor of PHD-2) was found to increase the fluorescence intensity, with the increase in concentration reflecting inhibition of PHD-2. The concentrations of BBAP-1 and cobalt were 3.93 μM, 7.87 μM, 15.75 μM, 31.5 μM, 61.25 μM, 125 μM, 250 μM, 500 μM, 1 mM, 2 mM, 4 mM and 8 mM. The effective inhibition and activation of PHD-2 were observed at 8 mM concentration of cobalt (inhibitor) and 8 mM concentration of BBAP-1 (activator) (Fig. 1C).

### Anti-proliferative effect of BBAP-1 upon cellular parameters

#### AO/EtBr staining of ER+MCF-7 cells

**Fig. 2:**
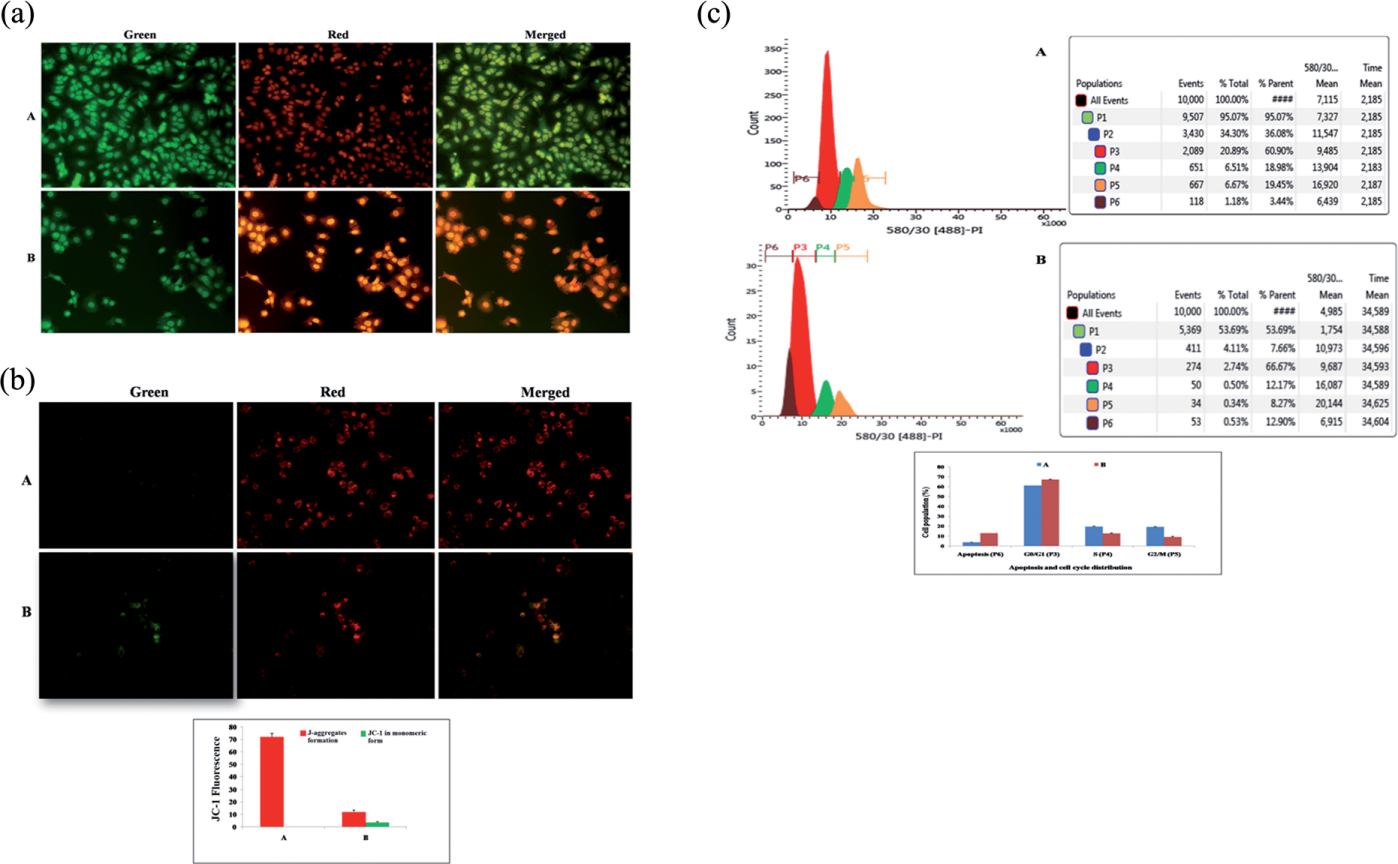
(A) Effect of BBAP-1 upon morphological symptoms of apoptosis: control (A) and BBAP-1 (IC50: 16.62 μM) treated (B) cells were dual stained with AO/EtBr (100 μg ml^−1^) in 1 : 1 ratio. The fluorescence was measured in green, red and merged channels. BBAP-1 treatment induced morphological features of apoptosis including fragmentation, chromatin condensation, apoptotic body formation, membrane blebbing and dotted nuclei. (B) Effect of BBAP-1 upon mitochondria membrane potential: control (A) and BBAP-1 (B) treated cells were incubated for 24 h followed by JC-1 staining (5 mg ml^−1^) for 1 h. The cells were examined under inverted fluorescence microscope in green, red and merged channels. Increase in green fluorescence reflects induction of apoptosis. (C) Effect of BBAP-1 upon cell cycle arrest and apoptotic cell burden: cell phase distribution assay through flow cytometry was performed in control (A) and BBAP-1 treated (16.62 μM) (B) cells. Histogram represents content of DNA with actual number of cells (*x* axis denotes fluorescence intensity of PE Texas red and *y* axis denotes cell count). BBAP-1 treatment arrests the cell cycle in G2/M and increases apoptotic cell burden.

The morphological analysis of cells was studied to determine the presence of cellular abnormalities in the cells, by which apoptotic cells can be differentiated from necrotic cells by their characteristic nuclear changes. To confirm whether the reduced cell viability was due to apoptosis, ER + MCF-7 cells were labeled by AO/EtBr after BBAP-1 treatment. The cells were examined under a fluorescent microscope. Apoptotic signals were not detected in the control group, which emitted a bright green signal from live cells. Early and late apoptotic signals (chromatin condensation, apoptotic body formation, membrane blebbing and dotted nuclei) were clearly visible in the BBAP-1 treated cells, which emitted orange to red fluorescence (Fig. 2A).

#### Measurement of mitochondrial membrane potential (Δ*ψ*)

The efficacy of BBAP-1 in the MTT and AO/EtBr tests motivated us to extend our study to evaluate the role of mitochondrial membrane potential (MMP), as decreased MMP is an important characteristic of early apoptosis. JC-1 is a cationic potential-dependent J-aggregate forming dye used to determine the loss of MMP (indicative of apoptosis). A decrease in the orange/red fluorescence indicates the loss of the dimeric form of JC-1 aggregates, and an increase in the green fluorescence intensity ratio indicates mitochondrial depolarization by the monomeric form of JC-1, both were observed after the BBAP-1 treatment (Fig. 2B).

#### Flow cytometry analysis

To identify the phase of cell cycle arrest caused by BBAP-1 treatment, flow cytometric analysis of the ER + MCF-7 cells was performed after PI staining for the evaluation of changes in the DNA content between the different experimental conditions. Treatment with BBAP-1 caused a distinct increase in DNA content by 1.094 fold in G0/G1 in comparison to the control (66.67% against 60.90%). Apoptotic cell burdens were multiplied 3.75 fold after BBAP-1 treatment in comparison to the control (12.90% against 3.44%). The cells were arrested in the G2/M phase, as the number of cells were decreased in the G2/M phase in comparison to the control, with a larger population of cells in the G0/G1 phase (8.27% in G2/M against 19.25% in G0/G1 in the treated group) (Fig. 2C).

### Effect of BBAP-1 upon tissue morphology in albino Wistar rats

#### Evaluation of ABs and DF score

**Fig. 3:**
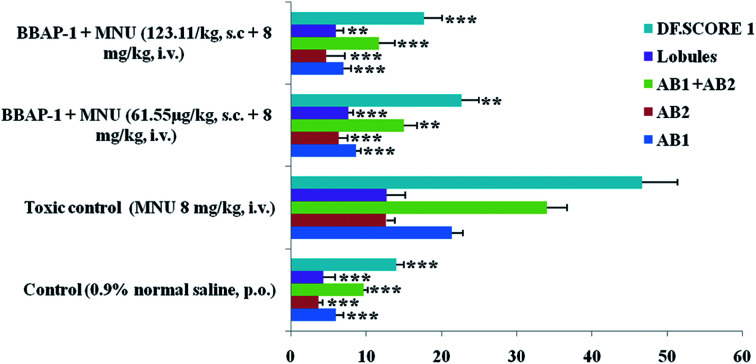
Effect of BBAP-1 on cellular proliferation of mammary gland of the animals treated with MNU: bar diagram represents the difference between the control, toxic and BBAP-1 treated groups. The presence of ABs, lobules and increased DF score was more prominent in MNU treated group. After BBAP-1 treatment, the numbers of ABs, lobules and DF score were minimized dose dependently. All the data is presented as mean ± SD. Comparisons are made on the basis of one-way ANOVA followed by Bonferroni multiple test and all groups are compared to the toxic control group (*p < 0.05, **p < 0.01, ***p < 0.001).

**Fig. 4:**
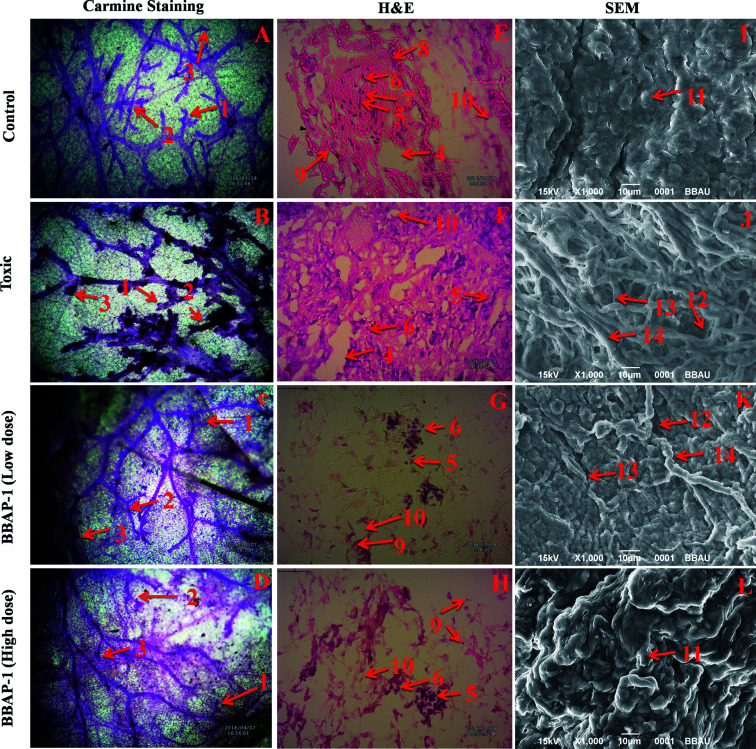
Effect of BBAP-1 upon cellular, morphological and surface architecture of mammary gland tissue: carmine staining of tissue revealed the presence of AB1 (1), AB2 (2), and lobules (3) in respective groups (A, B, C and D). The numbers of ABs and lobules were high in the toxic group (B) and reduced in the treatment groups (C and D). H&E staining of four respective groups (E, F, G and H) revealed ducts (4), MECs (5), CECs (6), LCT (7), DCT (8), lymphocytes (9) and adipocytes (10). In toxic control group (F), the cellular histology was distorted. SEM analysis of control (I) group revealed collagenous covering. Treatment with MNU shows loss of collagenous cushions (11), formation of ducts (12), development of tumor micro-vessels (13) and large blood vessels (14). MNU treatment induces cellular proliferation and treatment with BBAP-1 shows protection against deleterious effects of MNU.

AB count, lobules and DF score are characteristics of cellular proliferation and angiogenesis in tissues and were analyzed using carmine staining of the whole mount mammary gland tissue. MNU treatment increased the AB count (34.02 ± 2.64), lobules (12.69 ± 2.51) and DF score (46.67 ± 4.72) in comparison to the control. Treatment with BBAP-1 induced a significant decrease in the AB count (11.66 ± 2.12***), lobules (6.00 ± 0.98**) and DF score (17.66 ± 2.43***). The results of carmine staining indicated a noteworthy curtailment of cellular proliferation by BBAP-1 (Fig. 3 and 4A–D).

#### Identification of sub cellular structural abnormalities using H&E staining

The histopathological analysis of the mammary gland tissue was performed for the evaluation of the cellular architecture of the tissues. The H&E staining of the mammary gland tissue from the control animals revealed normal cellular architecture evidenced by the presence of CECs, MECs, lymphocytes, ducts, adipocytes, LCT and DCT. In contrast, MNU treatment resulted in the loss of normal cellular architecture along with the absence of lymphocytes, LCT and DCT. Concomitant BBAP-1 administration considerably restored the mammary gland architecture in terms of re-establishment of CECs, MECs, lymphocytes, ducts, adipocytes, LCT and DCT (Fig. 4E–H).

#### Analysis of surface architecture through SEM

The surface texture of the tissues was analyzed using SEM for the evaluation of formation of nodules, blood vessels and micro vessels in the respective treatment groups. In the control group, the collagenous covering was found. However, MNU treatment induced the loss of the collagenous cushion along with the development of small tumor micro vessels (representing proliferation) and large blood vessels. BBAP-1 diminished the number of microvessels as well as reestablishing the collagenous cushion in a dose dependent manner (Fig. 4I–L).

### Effect of BBAP-1 upon antioxidant parameters

**Fig. 5:**
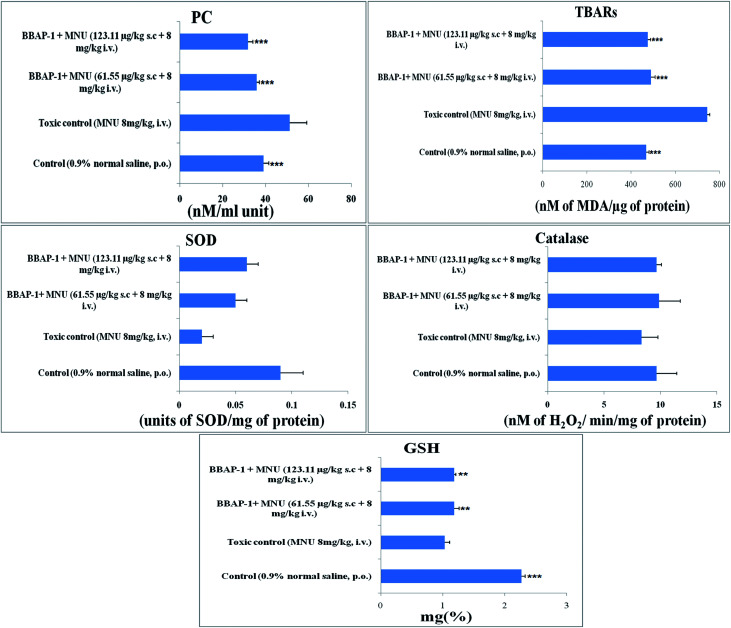
Effect of BBAP-1 on the antioxidant markers: bar diagram represents the difference between the control, toxic and BBAP-1 treated groups. The markers of protein and lipid (PC and TBARs) were increased in MNU treated group and reduced after BBAP-1 treatment. Other parameters like SOD, catalase and GSH were decreased after MNU treatment and restored after BBAP-1 treatment. All the data is presented as mean ± SD. Comparisons are made on the basis of one-way ANOVA followed by Bonferroni multiple test and all groups are compared to the toxic control group (*p < 0.05, **p < 0.01, ***p < 0.001).

Cancerous tissues generate oxidative stress and this was measured using antioxidant parameters including TBARs, SOD, catalase, GSH and PC. MNU treatment was found to increase the peroxidation products of lipids and protein, as apparent through the increased levels of TBARs (743.59 ± 11.91 nM of MDA per μg of protein) and PC (51.29 ± 7.95 nM ml^−1^ unit) in comparison to the control. BBAP-1 treatment diminished the deleterious effects of MNU. The biological levels of enzymatic antioxidant defense of GSH, SOD and catalase were diminished after the MNU treatment and were recorded as 1.03 ± 0.08 mg%, 0.02 ± 0.002 units of SOD per mg of protein and 8.33 ± 1.45 nM of H_2_O_2_ per min per mg of protein respectively. In line with the peroxidative markers, BBAP-1 treatment helped to reinstate the enzymatic antioxidant defense towards the level of the control (Fig. 5).

### Evaluation of serum caspase 3 and caspase 8 level

**Fig. 6:**
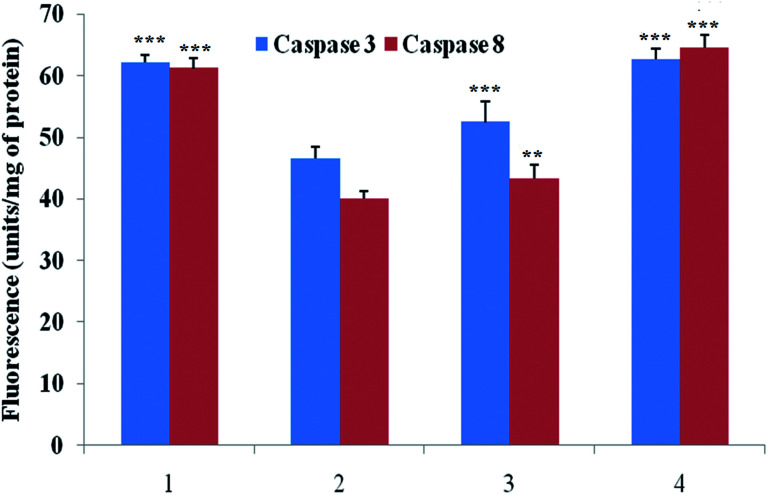
Effect of BBAP-1 on biological levels of caspase 3 and caspase 8 in the serum of experimental animals: the activity of caspase was determined using fluorescence based commercial kits. The data are represented as mean ± SD. Comparisons are made on the basis of one-way ANOVA followed by Bonferroni multiple test. All groups are compared to the toxic control group (*p < 0.05, **p < 0.01, ***p < 0.001). The groups were divided numerically as 1: control; 2: toxic control; 3: BBAP-1 + MNU (61.55 μg kg^−1^ s.c. + 8 mg kg^−1^ i.v.); 4: BBAP-1 + MNU (123.11 μg kg^−1^ s.c. + 8 mg kg^−1^ i.v.).

Caspase is an important marker for cell apoptosis and was evaluated using commercial serum based kits. The caspase 3 and caspase 8 levels were substantially down-regulated after the MNU administration, and BBAP-1 treatment helped to restore the levels of caspase 3 and caspase 8 towards the control (Fig. 6).

### Effect of BBAP-1 upon quantitative immunoblotting analysis

**Fig. 7:**
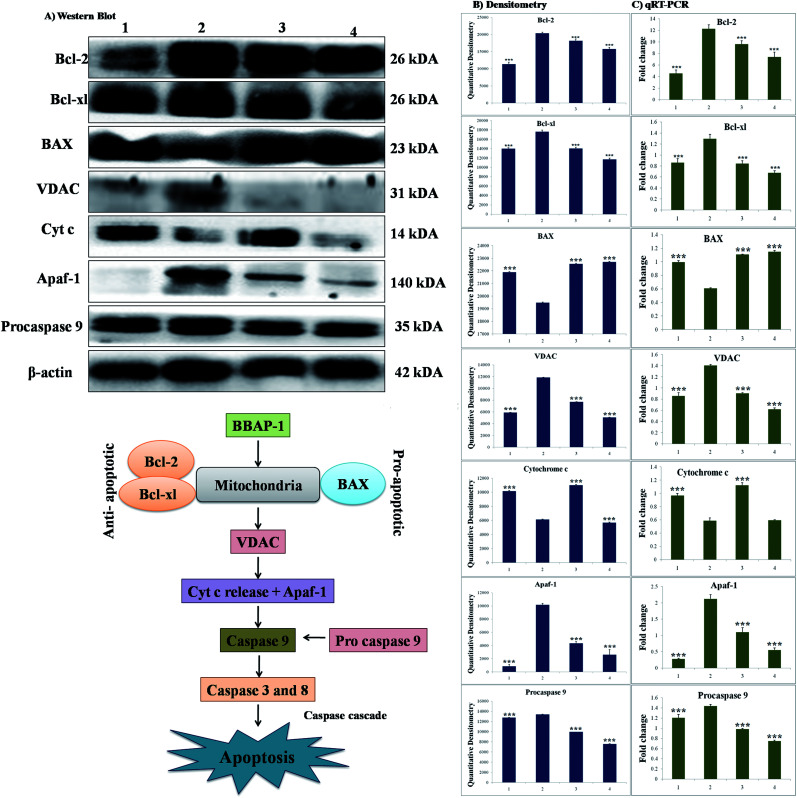
Effect of BBAP-1 treatment on mitochondrial mediated apoptosis: the protein was extracted from individual groups [1: control; 2: toxic control; 3: BBAP-1 + MNU (61.55 μg kg^−1^ s.c. + 8 mg kg^−1^ i.v.); 4: BBAP-1 + MNU (123.11 μg kg^−1^, s.c. + 8 mg kg^−1^ i.v.)] and subjected to immunoblotting of anti-apoptotic (Bcl-2 and Bcl-xl) and pro-apoptotic (BAX) proteins with downstream apoptotic markers (VDAC, cytochrome-c, Apaf-1 and procaspase 9) of mitochondrial mediated apoptotic pathway. The respective phenotypes for the different proteins were also validated using qRT-PCR. β-actin was used as internal loading control. The experiment was performed in triplicate. Values are presented as mean ± SD. Comparisons are made on the basis of one-way ANOVA followed by Bonferroni multiple test. All groups are compared to the toxic control group (*p < 0.05, **p < 0.01, ***p < 0.001).

**Fig. 8:**
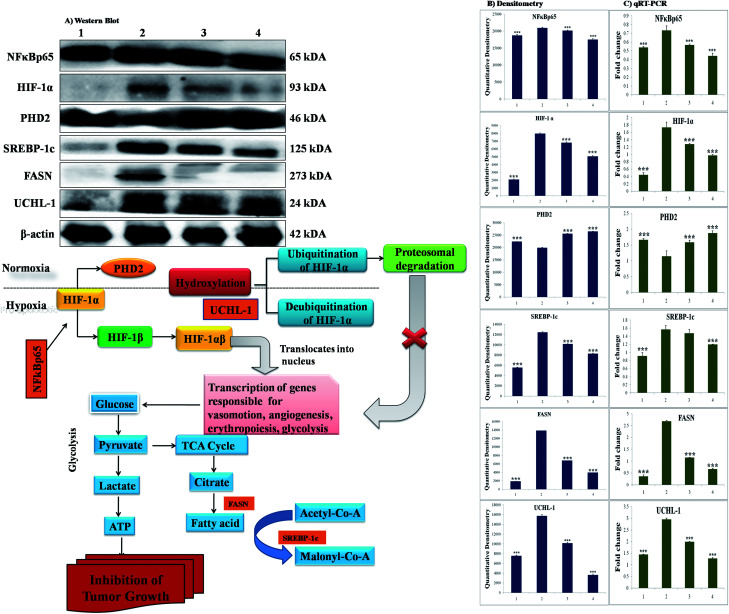
Effect of BBAP-1 treatment on hypoxic pathway in mammary gland cancer: protein was extracted from individual groups [1: control; 2: toxic control; 3: BBAP-1 + MNU (61.55 μg kg^−1^ s.c. + 8 mg kg^−1^ i.v.); 4: BBAP-1 + MNU (123.11 μg kg^−1^ s.c. + 8 mg kg^−1^ i.v.)] and subjected to immunoblotting of PHD-2, HIF-1α, FASN and other successive markers (NFκBp65, UCHL-1 and SREBP-1c). The up-regulated expression of PHD-2 and down-regulated HIF-1α expression demonstrate the activation of PHD-2 by BBAP-1 treatment. Subsequent reduced expression of FASN validates the triggering of PHD-2 by BBAP-1. β-actin was used as a loading control. Experimental values represent data derived from three individual experiments and are presented as mean ± SD. Comparisons are made on the basis of one-way ANOVA followed by Bonferroni multiple test. All groups are compared to the toxic control group (*p < 0.05, **p < 0.01, ***p < 0.001).

The participation of the mitochondrial mediated death apoptosis pathway was confirmed after immunoblotting analysis. The pathway is strongly regulated by the Bcl-2 family of proteins. The results revealed that after MNU treatment, the expressions of anti-apoptotic proteins Bcl-2 and Bcl-xl were upregulated, with the opposite effect on the pro-apoptotic protein BAX. BBAP-1 treatment restored the expression of Bcl-2, Bcl-xl and BAX towards the control. Other proteins of the mitochondrial mediated pathway (VDAC, Apaf-1 and cytochrome c) were strongly regulated after BBAP-1 treatment. BBAP-1 treatment downregulated the expression of VDAC, Apaf-1 and procaspase 9 while up-regulating expression of cytochrome c (Fig. 7). When measured through the biological markers of tumor hypoxia, the expression of PHD-2 was significantly up-regulated after BBAP-1 administration, suggesting activation of PHD-2. PHD-2 activation was also confirmed through down-regulated expression of HIF-1α, FASN and SREBP-1c after BBAP-1 treatment. Subsequently, the decreased expression of HIF-1α was furthermore affirmed through the abated expression of UCHL-1 and NF-κBp65 (Fig. 8).

### Quantification of mRNA expression of the targeted genes

The quantification of fold changes in genes was evaluated using qRT-PCR. The immunoblotting assay ascertained that mitochondrial-mediated apoptotic death was induced by BBAP-1 (Fig. 7) along with *in vivo* actuation of PHD-2, and the same findings were confirmed through the study of genomic contributors using qRT-PCR (Fig. 8).

## Discussion

The present study revealed the potential role of PHD-2 activators in curtailing the growth of tumor cells by down-regulating the level of HIF-1α and FASN. BBAP-1 was identified as a potential activator of PHD-2 through a series of *in silico* tools and subjected to additional evaluation (Fig. 1A). BBAP-1 demonstrated noteworthy cytotoxic effects against ER + MCF-7 cells and was therefore considered for further evaluation (Fig. 1B). BBAP-1 treatment upregulated the level of PHD-2 when scrutinized through 2-OG dependent assay (Fig. 1C). Treatment with BBAP-1 induced morphological changes associated with apoptosis including membrane blebbing, fragmentation, chromatin condensation, the formation of apoptotic bodies and dotted nuclei when examined through AO/EtBr staining (Fig. 2A). BBAP-1 treatment also produced a marked decrease in mitochondrial membrane potential as visualized through JC-1 staining (Fig. 2B). Finally, cell cycle analysis was studied through PI staining to identify the population of cells existing in the G0/G1, S and G2/M phases.^44^ BBAP-1 treatment reduced the number of cells in the G2/M phase, as evidenced by the higher cell population in the G0/G1 phase along with the increase in apoptotic cell burden (Fig. 2C).

Subsequently, the efficacy of BBAP-1 was also validated against the MNU induced mammary gland carcinoma model, which enables us to give a comprehensive assessment of its potential. The *in vivo* efficacy of BBAP-1 was further scrutinized on morphological grounds using carmine staining, H&E staining and SEM. The MNU induced mammary gland model is a well-established model for mammary gland carcinoma in rats.^45^ MNU is an alkylating agent, and one possible site of alkylation is the guanine base pair of DNA.^46^ The alkylation leads to the formation of a short-lived *N*-methyl guanine and a long-lived *O*-methyl guanine intermediate. Methylation of the *O* position changes the hydrogen bonding properties of guanine, thereby inducing the guanine to adenosine transition and causing DNA damage.^47^ MNU treatment increased the AB count and DF score and the findings are in line with a previous report.^48^ BBAP-1 treatment diminished both the AB count and DF score, suggesting the anti-proliferative effect of BBAP-1 (Fig. 3 and 4A–D). The results derived from carmine staining were subsequently affirmed by the histopathological evaluation and SEM analysis. Histopathological examination of the mammary gland tissue of the animals treated with MNU revealed the distorted histological architecture characterized by the scattered pattern of CECs, loss of ducts and MECs. The LCT and DCT were hard to identify, which is in agreement with the earlier report (Fig. 4E–H).^39^ In addition, SEM analysis of the mammary gland tissue of the MNU treated group demonstrated the presence of tumor micro-vessels, enlarged capillaries along with the loss of intra-arterial cushion. BBAP-1 treatment restored the surface architecture (Fig. 4I–L).

Progression of carcinogenesis is also related to generation of reactive oxygen species (ROS) and oxidative stress markers in multiple ways.^49^ Treatment with MNU intensified the lipid and protein peroxidation as manifested through increased levels of TBARs and PC, suggesting ROS generation. Increased ROS generation was also affirmed through the down-regulated levels of enzymatic defense of SOD/catalase/GSH.^50^ The increase in TBARs and PC could be attributed to the alkylating effect of MNU. The down-regulated enzymatic antioxidant defense could be attributed to the increased utilization of these enzymes to curtail progressive ROS generation. SOD dismutase catalyzes the O˙ to molecular O_2_ and H_2_O_2_. The H_2_O_2_ thus generated is catalyzed by catalase and GSH peroxidase to water. SOD, catalase and GSH work together to nudge ROS (O˙) .^51^ Concomitant treatment with BBAP-1 helped to restore the levels of SOD/catalase/GSH and curtail the production of TBARs and PC thereby reflecting the diminution of oxidative stress induced by MNU (Fig. 5).

We further extended the horizon of the study to elaborate the effect of BBAP-1 upon the biological markers of apoptosis. The expression of anti-apoptotic Bcl-2 and Bcl-xl was decreased after BBAP-1 treatment with the opposite effect upon pro-apoptotic BAX. The modulation of the apoptotic signals at the protein level was also marked at the mRNA level.^52^ The triggering of apoptosis can be further observed through decreased expression of VDAC and release of cytochrome c from mitochondria to cytosol.^53^ Treatment with BBAP-1 caused decreased VDAC and increased cytochrome c expression, which was also evident for the respective phenotypes at the mRNA level. Released cytochrome c in the cytosol interacts with Apaf-1, resulting in apoptosome formation for the execution of apoptosis.^54^ Apoptosome formation can be confirmed through decreased expression of Apaf-1 and this was observed after BBAP-1 treatment. The formation of apoptosome cleaves the procaspase 9 to give active caspase 9, and BBAP-1 treatment reduced the expression of procaspase 9.^55^ It is worth mentioning that activated caspase 9 further actuates the effectors caspase 3 and 8 for execution of apoptosis.^56^ BBAP-1 treatment up-regulated the levels of caspase 3 and 8 and thereby accelerated apoptosis (Fig. 6) which affirms the induction of mitochondrial mediated apoptosis after BBAP-1 treatment (Fig. 7).

In order to validate our hypothesis, the effect of BBAP-1 upon PHD-2 mediated proteasomal degradation of HIF-1α was scrutinized. It would be appropriate to remark that during hypoxia, HIF-1α escapes proteasomal degradation and promotes tumor growth through up-regulating fatty acid synthesis.^57,58^ The increased fatty acid demand of the tumor cells is fulfilled through FASN, which is very well reported to be overexpressed in tumor cells.^59,60^ It would be appropriate to mention that the transcriptional activity of FASN is regulated with the help of transcription factor SREBP1-c.^61^ The expression of PHD-2 was increased after BBAP-1 treatment followed by decreased expression of HIF-1α, demonstrating its proteasomal degradation. Treatment with BBAP-1 also downregulated the expression of FASN and SREBP-1c, confirming decreased fatty acid synthesis and lipogenic activity in mammary gland tissues. The PHD-2 mediated proteasomal degradation of HIF-1α was further validated through UCHL-1.^62^ The 20s immunoproteosomal subunit of UCHL-1 participated in the deubiquitination of HIF-1α, making HIF-1α more stable and thereby promoting the hypoxic microenvironment of the cancerous cells.^63^ In fact, UCHL-1 has been shown to provide diagnostic and anti-metastatic strategies *via* its deubiquitination effect upon HIF-1α.^63^ Treatment with BBAP-1 decreased UCHL-1 expression, affirming the proteasomal degradation of HIF-1α (Fig. 8). The role of UCHL-1 was validated through inhibition of NFκBp65 subunit in treatment groups, which is in line with our previous experimental outcome.^36^

## Conclusion

The authors would like to conclude that the line of evidence derived from the present work validates our hypothesis that the exogenous chemical activation of PHD-2 can favorably regulate cellular proliferation through mitochondrial mediated apoptosis. In this context, BBAP-1 was recorded to be a chemical activator of PHD-2 with subsequent down-regulation of the expression of HIF-1α and FASN. The authors would also like to submit that the present study explicitly endorses the *in vivo* efficacy of BBAP-1 against mammary gland carcinoma and illustrates the need for clinical validation for exploring its future prospect as an anticancer agent.

## Funding

None.

## Conflicts of interest

Authors declare no conflict of interest.

## Supplementary Material

RA-008-C8RA01239C-s001
