# Supplementary material for: Chemical activation of prolyl hydroxylase-2 by BBAP-1 down regulates hypoxia inducible factor-1α and fatty acid synthase for mammary gland chemoprevention
Source: RSC Adv. 2018 Apr 4;8(23):12848–60. doi: 10.1039/c8ra01239c (PMC9079607; doi:10.1039/c8ra01239c)
Supplement: RA-008-C8RA01239C-s001 [file RA-008-C8RA01239C-s001.pdf]

Electronic Supplementary Material (ESI) for RSC Advances.
This journal is © The Royal Society of Chemistry 2018

Fig S1

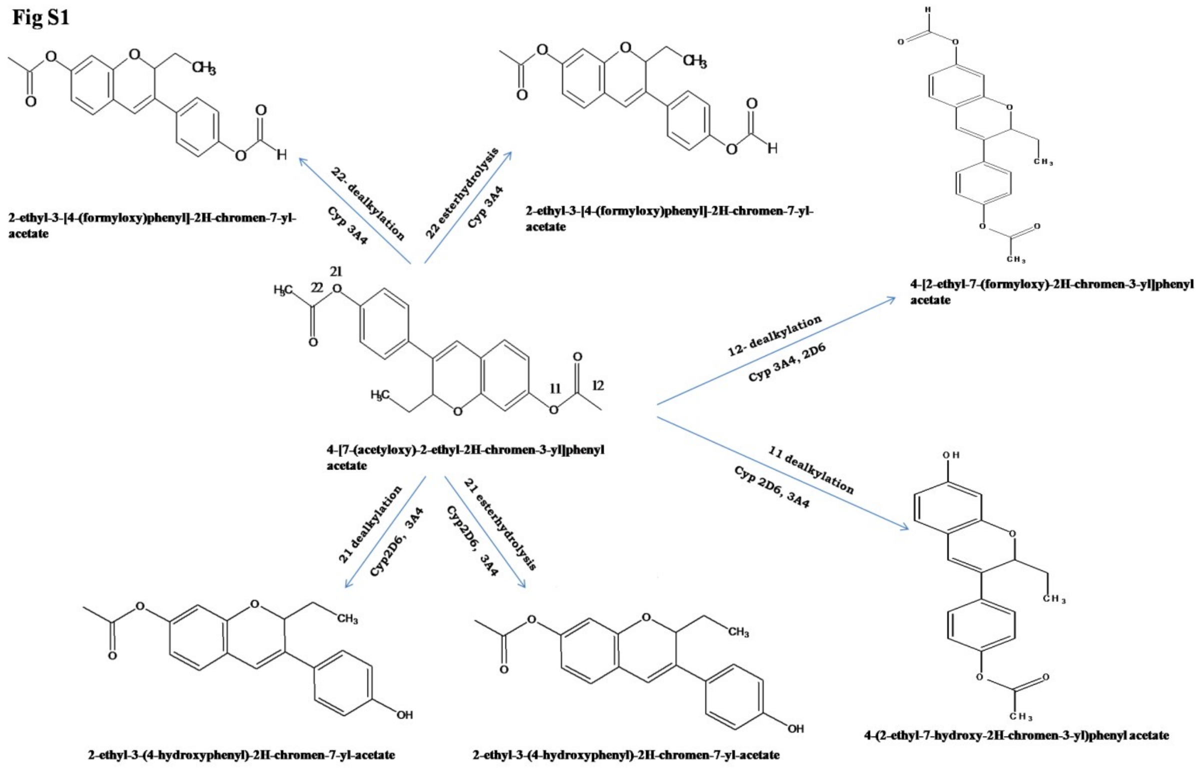

Supplementary figure 1: Metabolic profiling of BBAP-1

The metabolic profiling of BBAP-1 performed through metaprint 2D and WhichCyp revealed 11th, 12th, 21st and 22nd carbon positions as major site for metabolism through dealkylation and ester hydrolysis and major cytochrome P450 enzymes involved were CYP3A4 and CYP2D6.

Table S1: Sequence of forward and reverse primers used for quantitative RT-PCR

Primer	Sequence
Bcl2 F	GTGGATGACTGAGTACCTGAAC
Bcl2 R	GAGACAGCCAGGAGAAATCAA
Bcl-xl F	CCCTCGTATCTGGAAGCCAC
Bcl-xl-R	CAGCGGAGACCTCGTTTTCT
BAX F	TGCTACAGGGTTTCATCCAG
BAX R	GACACTCGCTCAGCTTCTT
VDAC F	GGAGTTTGGTGGCTCCATTTA
VDAC R	GACCTGATACTTGGCTGCTATTC
Cytochrome-c F	TCCATTTCCCTTCCTTGGGC
Cytochrome-c R	ATCGGGGCTGTCCAACAAAA
Apaf-1F	GAACATAGACTCCCGGGTAAAG
Apaf-1R	CTTGTCTCCCAGACCCTTATTG
Procaspase9F	GGCTCTCTGGCTTCATTCTT
Procaspase9R	GGGTCCAGCTTCACTACTTTC
PHD2 F	ACGCAGTTCATACCCAGTTAG
PHD2 R	CCTGTCCACTCTCAGCTTTAC
HIF1-α F	GATGGGTTATGAGCCAGAAGAA
HIF1-α R	CTGTGGTGACTTGTCCTTTAGT
FASN F	GGCGAGTCTATGCCACTATTC
FASN R	GCTGATACAGAGAACGGATGAG
SREBP-1cF	TCCGAGTTCCAGGTAGGGTT
SREBP-1cR	CTTGGCGCACACCAAATACC
UCHL-1 F	CGCCTCTGCCCTGAGTTATT
UCHL-1 R	CCGTCTGGGTCAATCCTCTG
NFκBp65 F	GGGCTACGAAGTCAAACCCA
NKκBp65 R	TTCTCCTCAATCCGGTGACG
β-actin F	TGCAGGATCGTGAGGAACAC
β-actin R	AGCGTGATTGTAACGCCTGA

Table S2: Screened compounds from Zinc database

COc1cccc2c1O[C@H](C(=C2)[N+](=O)[O-])c3ccc(cc3)F
CC1(C=Cc2cc(c(cc2O1)OC)OC)C
CCOc1cc2c(cc1OC)C=CC(O2)(C)C
c1ccc(cc1)c2cn3cc(cc(c3n2)Cl)C(F)(F)F
c1cc2cccc3c2c(c1)C(=C3Br)Br
c1ccc2c(c1)ccc3c2C=C([C@H](O3)c4ccc(cc4)F)[N+](=O)[O-]
c1ccc2c(c1)ccc3c2C=C([C@@H](O3)c4ccc(cc4)F)[N+](=O)[O-]
COc1cccc2c1O[C@@H](C(=C2)[N+](=O)[O-])c3ccc(cc3)F
CCOc1cccc2c1O[C@H](C(=C2)[N+](=O)[O-])c3ccc(cc3)F
CCOc1cccc2c1O[C@@H](C(=C2)[N+](=O)[O-])c3ccc(cc3)F
CC(C)c1ccc(cc1)[C@H]2C(=Cc3ccccc3O2)[N+](=O)[O-]
CC(C)c1ccc(cc1)[C@@H]2C(=Cc3ccccc3O2)[N+](=O)[O-]
c1ccc(cc1)S(=O)(=O)n2ccc3c2cccc3
c1cc2c(cc1CNS(=O)(=O)c3ccc(c(c3)Cl)F)OCO2
CC1(C=Cc2ccc(cc2O1)OCc3ccc(cc3)[N+](=O)[O-])C
C[C@@H](C(=O)Nc1ccccc1)Oc2ccc3c(c2)OC(C=C3)(C)C
C[C@H](C(=O)Nc1ccccc1)Oc2ccc3c(c2)OC(C=C3)(C)C
CC1=NC2=C([C@@H](C1C#N)c3ccc(c(c3)OC)OC)C(=O)CCC2
CC1=NC2=C([C@@H](C1C#N)c3ccc(cc3OC)OC)C(=O)CCC2
COc1ccccc1N2CCN(CC2)S(=O)(=O)c3cccs3
CCOC1=C[C@H](Oc2c1cccc2)c3ccc(cc3)F
CCOC1=C[C@@H](Oc2c1cccc2)c3ccc(cc3)F
Cc1cc(ccc1OC)CCCC(=O)NC2CCCCC2
CC[n+]1c2ccccc2n(c1N)CC(=O)c3ccco3
COc1ccc(cc1OC)NS(=O)(=O)c2cccs2
Cc1cccc(c1)N2CCN(CC2)S(=O)(=O)c3ccccc3
c1ccc(cc1)COc2ccc(cc2)CNc3ccc(cc3)F
COc1cc(ccc1OCc2ccccc2)CNc3cccc(c3)F
c1ccc(cc1)COc2ccc(cc2)CNc3cccc(c3)F

c1ccc(cc1)COc2ccc(cc2)CNc3ccccc3F
COc1ccc(cc1OCc2ccccc2)CNc3cccc(c3)F
COc1cc(ccc1OCc2ccccc2)CNc3ccccc3F
COc1cc(ccc1OCc2ccccc2)CNc3ccc(cc3)F
c1ccc(cc1)COc2cccc(c2)CNc3ccccc3F
c1cc(c(cc1Cl)NC(=O)[C@@H]2CC=CC[C@H]2C(=O)[O-])Cl
Cc1cc(cc(c1)NC(=O)c2ccccc2OCc3ccccc3F)C
Cc1ccc(cc1C)C(=O)NC(=S)Nc2ccc(c(c2)C)C
Cc1ccc(c(c1)C)OCC(=O)Nc2cc(ccc2F)[N+](=O)[O-]
CC[NH+](CC)CCN1[C@@H](Nc2ccccc2C1=O)c3cccc(c3)F
CCOc1cccc(c1OCc2ccccc2)CNc3ccc(cc3)O
c1ccc(c(c1)CNc2ccc(cc2)O)OCc3ccccc3F
c1cc(cc(c1)OCc2ccc(cc2)F)CNc3ccc(cc3)O
c1ccc(cc1)COc2ccccc2CNc3ccc(cc3)O
COc1c2c(ccc(=O)o2)cc3c1OCC3
c1ccc(cc1)S(=O)(=O)N2CCc3c2ccc(c3)Br
CC1(C=Cc2cc(c(cc2O1)OC)O)C
Cc1c(c(=O)n2ccccc2n1)NC(=O)c3ccccc3
Cc1nc(cs1)c2ccc(cc2)NC(=O)C3CCCCC3
c1ccc(cc1)Nc2nnns2
CC1(C=Cc2ccc(cc2O1)OC)C
COc1ccc(cc1)C[NH+]2CCN(CC2)C(=O)c3cccnc3
c1cc(ncc1Cl)NC(=O)NC2CCCCC2
c1ccc(cc1)NCc2ccccc2OCc3cccc(c3)F
c1ccc(cc1)NCc2ccc(cc2)OCc3ccc(cc3)F
Cc1cccc(c1)NCc2ccccc2OCc3ccccc3F
Cc1ccc(cc1C)NCc2cccc(c2)OCc3cccc(c3)F
Cc1ccc(cc1)NCc2ccccc2OCc3ccccc3F
c1ccc(cc1)NC(=O)Nc2ccc(nc2Cl)Cl
CCO[C@H]1C=C(Oc2c1cccc2)c3ccc(cc3)F
CCO[C@@H]1C=C(Oc2c1cccc2)c3ccc(cc3)F

CN(c1ccccc1)S(=O)(=O)c2ccc3ccccc3c2
c1ccc(cc1)c2nnc(n2N)SCC(=O)[O-]
c1ccc(cc1)NCc2ccc(cc2)OCc3ccccc3F
CC1(CC2=C([C@@H](C(=C(O2)N)C(=O)OC)c3cc(ccc3OCc4ccccc4)Br)C(=O)C1)C
Cc1cc(ccc1OC)[C@@H]2C(=C(N(C3=C2C(=O)CC(C3)(C)C)Nc4ccccc4)N)C#N
c1ccc(cc1)C(=O)N2CCN(CC2)c3ccc(cc3)NC(=S)NC(=O)c4ccco4
COc1ccc(cc1NC(=O)c2ccc(o2)c3ccc(cc3)Br)c4nc5ccccc5o4
Cc1ccccc1NC(=O)c2cc(c(c(c2)Br)OCc3ccccc3)OC
COc1ccccc1NC(=O)c2cc(c(c(c2)I)OCc3ccccc3)OC
COc1cc(cc(c1OCc2ccccc2)I)C(=O)Nc3cccc(c3)O
CCOc1cc(cc(c1OCc2ccccc2)I)C(=O)Nc3ccc(cc3)O
COc1cccc(c1OCc2ccccc2F)CNc3ccc(cc3)S(=O)(=O)N
c1ccc(c(c1)C#N)NC(=O)Cn2cnc3ccc(cc3c2=O)I
c1ccc(cc1)C(=O)C2=C(C(=O)N([C@H]2c3ccccc3Cl)CCc4c[nH]c5c4cccc5)[O-]
c1ccc(cc1)C(=O)C2=C(C(=O)N([C@@H]2c3ccccc3Cl)CCc4c[nH]c5c4cccc5)[O-]
COc1cccc(c1)[C@H]2C(=C(C(=O)N2CCc3c[nH]c4c3cccc4)[O-])C(=O)c5ccccc5
COc1cccc(c1)[C@@H]2C(=C(C(=O)N2CCc3c[nH]c4c3cccc4)[O-])C(=O)c5ccccc5
CCc1ccc(cc1)[C@H]2C(=C(C(=O)N2CCc3c[nH]c4c3cccc4)[O-])C(=O)c5ccccc5
CCc1ccc(cc1)[C@@H]2C(=C(C(=O)N2CCc3c[nH]c4c3cccc4)[O-])C(=O)c5ccccc5
COc1ccc(cc1)C(=O)C2=C(C(=O)N([C@H]2c3cccnc3)CCc4ccc(c(c4)OC)OC)[O-]
COc1ccc(cc1)C(=O)C2=C(C(=O)N([C@@H]2c3cccnc3)CCc4ccc(c(c4)OC)OC)[O-]
Cc1ccc(cc1)C(=O)C2=C(C(=O)N([C@@H]2c3cccnc3)CCc4ccc(c(c4)OC)OC)[O-]
COC(=O)C1=C(S[C@]2([C@@H]1C(=O)n3c2nc4c3cccc4)c5ccccc5)c6ccccc6
COC(=O)C1=C(S[C@]2([C@H]1C(=O)n3c2nc4c3cccc4)c5ccccc5)c6ccccc6
Cc1cccc(c1)N2CCN(CC2)c3ccc(cc3)NC(=O)/C=C(\c4ccccc4)/C(=O)[O-]
c1ccc(c(c1)C(=O)Nc2ccc(cc2)N3CCN(CC3)c4cccc(c4)Cl)C(=O)[O-]
c1ccc(c(c1)C(=O)Nc2ccc(cc2)N3CCN(CC3)c4ccc(cc4)Cl)C(=O)[O-]
c1ccc(c(c1)C(=O)Nc2ccc(cc2)N3CCN(CC3)c4cccc(c4Cl)Cl)C(=O)[O-]
c1ccc(c(c1)C(=O)Nc2ccc(cc2)N3CCN(CC3)c4ccc(c(c4)Cl)Cl)C(=O)[O-]
c1cc(cc(c1)Cl)N2CCN(CC2)c3ccc(cc3)NC(=O)[C@H]4CC=CC[C@H]4C(=O)[O-]
c1cc(cc(c1)Cl)N2CCN(CC2)c3ccc(cc3)NC(=O)[C@@H]4CC=CC[C@H]4C(=O)[O-]

c1cc(cc(c1)Cl)N2CCN(CC2)c3ccc(cc3)NC(=O)[C@H]4CC=CC[C@@H]4C(=O)[O-]
c1cc(cc(c1)Cl)N2CCN(CC2)c3ccc(cc3)NC(=O)[C@@H]4CC=CC[C@@H]4C(=O)[O-]
c1cc(c(c(c1)Cl)Cl)N2CCN(CC2)c3ccc(cc3)NC(=O)[C@H]4CC=CC[C@H]4C(=O)[O-]
c1cc(c(c(c1)Cl)Cl)N2CCN(CC2)c3ccc(cc3)NC(=O)[C@@H]4CC=CC[C@H]4C(=O)[O-]
c1cc(c(c(c1)Cl)Cl)N2CCN(CC2)c3ccc(cc3)NC(=O)[C@H]4CC=CC[C@@H]4C(=O)[O-]
c1cc(c(c(c1)Cl)Cl)N2CCN(CC2)c3ccc(cc3)NC(=O)[C@@H]4CC=CC[C@@H]4C(=O)[O-]
c1cc(c(c(c1)Cl)Cl)N2CCN(CC2)c3ccc(cc3)NC(=O)[C@H]4CCCC[C@H]4C(=O)[O-]
c1cc(c(c(c1)Cl)Cl)N2CCN(CC2)c3ccc(cc3)NC(=O)[C@@H]4CCCC[C@H]4C(=O)[O-]
c1cc(c(c(c1)Cl)Cl)N2CCN(CC2)c3ccc(cc3)NC(=O)[C@H]4CCCC[C@@H]4C(=O)[O-]
c1cc(c(c(c1)Cl)Cl)N2CCN(CC2)c3ccc(cc3)NC(=O)[C@@H]4CCCC[C@@H]4C(=O)[O-]
Cc1cccc(c1)N2CCN(CC2)C(=O)c3ccccc3NC(=O)[C@H]4CCCC[C@H]4C(=O)[O-]
Cc1cccc(c1)N2CCN(CC2)C(=O)c3ccccc3NC(=O)[C@@H]4CCCC[C@H]4C(=O)[O-]
Cc1cccc(c1)N2CCN(CC2)C(=O)c3ccccc3NC(=O)[C@H]4CCCC[C@@H]4C(=O)[O-]
Cc1cccc(c1)N2CCN(CC2)C(=O)c3ccccc3NC(=O)[C@@H]4CCCC[C@@H]4C(=O)[O-]
Cc1ccc(cc1)N2CCN(CC2)C(=O)c3ccccc3NC(=O)[C@H]4CCCC[C@H]4C(=O)[O-]
Cc1ccc(cc1)N2CCN(CC2)C(=O)c3ccccc3NC(=O)[C@@H]4CCCC[C@H]4C(=O)[O-]
Cc1ccc(cc1)N2CCN(CC2)C(=O)c3ccccc3NC(=O)[C@H]4CCCC[C@@H]4C(=O)[O-]
Cc1ccc(cc1)N2CCN(CC2)C(=O)c3ccccc3NC(=O)[C@@H]4CCCC[C@@H]4C(=O)[O-]
COc1ccccc1N2CCN(CC2)C(=O)c3ccccc3NC(=O)[C@H]4CCCC[C@H]4C(=O)[O-]
COc1ccccc1N2CCN(CC2)C(=O)c3ccccc3NC(=O)[C@@H]4CCCC[C@H]4C(=O)[O-]
COc1ccccc1N2CCN(CC2)C(=O)c3ccccc3NC(=O)[C@H]4CCCC[C@@H]4C(=O)[O-]
COc1ccccc1N2CCN(CC2)C(=O)c3ccccc3NC(=O)[C@@H]4CCCC[C@@H]4C(=O)[O-]
c1ccc(c(c1)C(=O)N2CCN(CC2)c3ccc(cc3)F)NC(=O)[C@H]4CCCC[C@@H]4C(=O)[O-]
Cc1ccccc1N2CCN(CC2)C(=O)c3ccccc3NC(=O)[C@H]4CC=CC[C@H]4C(=O)[O-]
Cc1ccccc1N2CCN(CC2)C(=O)c3ccccc3NC(=O)[C@@H]4CC=CC[C@H]4C(=O)[O-]
Cc1ccccc1N2CCN(CC2)C(=O)c3ccccc3NC(=O)[C@H]4CC=CC[C@@H]4C(=O)[O-]
Cc1ccccc1N2CCN(CC2)C(=O)c3ccccc3NC(=O)[C@@H]4CC=CC[C@@H]4C(=O)[O-]
Cc1cccc(c1)N2CCN(CC2)C(=O)c3ccccc3NC(=O)[C@H]4CC=CC[C@H]4C(=O)[O-]
Cc1cccc(c1)N2CCN(CC2)C(=O)c3ccccc3NC(=O)[C@@H]4CC=CC[C@H]4C(=O)[O-]
Cc1cccc(c1)N2CCN(CC2)C(=O)c3ccccc3NC(=O)[C@H]4CC=CC[C@@H]4C(=O)[O-]
Cc1cccc(c1)N2CCN(CC2)C(=O)c3ccccc3NC(=O)[C@@H]4CC=CC[C@@H]4C(=O)[O-]

Cc1ccc(cc1)N2CCN(CC2)C(=O)c3ccccc3NC(=O)[C@H]4CC=CC[C@H]4C(=O)[O-]
Cc1ccc(cc1)N2CCN(CC2)C(=O)c3ccccc3NC(=O)[C@@H]4CC=CC[C@H]4C(=O)[O-]
Cc1ccc(cc1)N2CCN(CC2)C(=O)c3ccccc3NC(=O)[C@H]4CC=CC[C@@H]4C(=O)[O-]
Cc1ccc(cc1)N2CCN(CC2)C(=O)c3ccccc3NC(=O)[C@@H]4CC=CC[C@@H]4C(=O)[O-]
CC(C)c1ccc(cc1)N2CCN(CC2)C(=O)c3ccccc3NC(=O)[C@H]4CC=CC[C@H]4C(=O)[O-]
CC(C)c1ccc(cc1)N2CCN(CC2)C(=O)c3ccccc3NC(=O)[C@@H]4CC=CC[C@H]4C(=O)[O-]
CC(C)c1ccc(cc1)N2CCN(CC2)C(=O)c3ccccc3NC(=O)[C@H]4CC=CC[C@@H]4C(=O)[O-]
CC(C)c1ccc(cc1)N2CCN(CC2)C(=O)c3ccccc3NC(=O)[C@@H]4CC=CC[C@@H]4C(=O)[O-]
c1ccc(c(c1)C(=O)Nc2ccc(c(c2)Cl)Cl)NC(=O)[C@H]3CCCC[C@H]3C(=O)[O-]
c1ccc(c(c1)C(=O)Nc2ccc(c(c2)Cl)Cl)NC(=O)[C@@H]3CCCC[C@H]3C(=O)[O-]
c1ccc(c(c1)C(=O)Nc2ccc(c(c2)Cl)Cl)NC(=O)[C@H]3CCCC[C@@H]3C(=O)[O-]
c1ccc(c(c1)C(=O)Nc2ccc(c(c2)Cl)Cl)NC(=O)[C@@H]3CCCC[C@@H]3C(=O)[O-]
Cc1ccccc1N2CCN(CC2)C(=O)c3ccccc3NC(=O)c4ccccc4C(=O)[O-]
Cc1ccc(cc1)N2CCN(CC2)C(=O)c3ccccc3NC(=O)c4ccccc4C(=O)[O-]
c1ccc(c(c1)C(=O)Nc2ccccc2C(=O)Nc3ccc(c(c3)Cl)Cl)C(=O)[O-]
CC1=C([C@H](n2c3ccccc3nc2N1)c4ccccc4[N+](=O)[O-])C(=O)OC[C@H]5CCCO5
CC1=C([C@@H](n2c3ccccc3nc2N1)c4ccccc4[N+](=O)[O-])C(=O)OC[C@H]5CCCO5
CC1=C([C@H](n2c3ccccc3nc2N1)c4ccccc4[N+](=O)[O-])C(=O)OC[C@@H]5CCCO5
CC1=C([C@@H](n2c3ccccc3nc2N1)c4ccccc4[N+](=O)[O-])C(=O)OC[C@@H]5CCCO5
Cc1cc2c(cc1C)n3c(n2)NC(=C([C@@H]3c4cccc(c4)Br)C(=O)OC)C
COc1cc(ccc1OC2CCCC2)CCNC(=S)Nc3c(cncc3Cl)Cl
c1cc(sc1)C(=O)Cn2cnc3c(c2=O)cc(cc3Br)Br
c1cc(ccc1c2cn3ccsc3n2)NC(=O)c4ccc(cc4)I
c1cc(cc(c1)I)C(=O)Nc2ccc(cc2)c3cn4ccsc4n3
Cc1ccn2cc(nc2c1)c3cccc(c3)NC(=O)c4cccc(c4)I
COc1ccc(cc1OC)CC(=O)Nc2cccc(c2)c3csc(n3)c4ccccc4
CCOc1cc(cc(c1OCC)OCC)C(=O)Nc2ccc(cc2)c3csc(n3)C
Cc1ccc(cc1)[C@@H]([C@@H](Cn2cncn2)C(=O)c3ccccc3)Sc4ccc(cc4)C
Cc1ccc(cc1)[C@H]([C@@H](Cn2cncn2)C(=O)c3ccccc3)Sc4ccc(cc4)C
Cc1ccc(cc1)[C@@H]([C@H](Cn2cncn2)C(=O)c3ccccc3)Sc4ccc(cc4)C
Cc1ccc(cc1)[C@H]([C@H](Cn2cncn2)C(=O)c3ccccc3)Sc4ccc(cc4)C

```
c1ccc(cc1)[C@@H]([C@@H](Cn2cncn2)C(=O)c3ccccc3)Sc4ccc(cc4)Cl
c1ccc(cc1)[C@H]([C@@H](Cn2cncn2)C(=O)c3ccccc3)Sc4ccc(cc4)Cl
c1ccc(cc1)[C@@H]([C@H](Cn2cncn2)C(=O)c3ccccc3)Sc4ccc(cc4)Cl
c1ccc(cc1)[C@H]([C@H](Cn2cncn2)C(=O)c3ccccc3)Sc4ccc(cc4)Cl
c1ccc(cc1)[C@@H]([C@@H](Cn2cncn2)C(=O)c3ccc(cc3)Cl)Sc4ccccc4
c1ccc(cc1)[C@H]([C@@H](Cn2cncn2)C(=O)c3ccc(cc3)Cl)Sc4ccccc4
c1ccc(cc1)[C@@H]([C@H](Cn2cncn2)C(=O)c3ccc(cc3)Cl)Sc4ccccc4
c1ccc(cc1)[C@H]([C@H](Cn2cncn2)C(=O)c3ccc(cc3)Cl)Sc4ccccc4
COc1ccc(cc1)C(=O)[C@@H](Cn2cncn2)[C@@H](c3ccccc3)Sc4ccccc4
COc1ccc(cc1)C(=O)[C@H](Cn2cncn2)[C@@H](c3ccccc3)Sc4ccccc4
COc1ccc(cc1)C(=O)[C@@H](Cn2cncn2)[C@H](c3ccccc3)Sc4ccccc4
COc1ccc(cc1)C(=O)[C@H](Cn2cncn2)[C@H](c3ccccc3)Sc4ccccc4
c1ccc(cc1)[C@@H]([C@@H](Cn2cncn2)C(=O)c3ccc(cc3)Br)Sc4ccccc4
c1ccc(cc1)[C@H]([C@@H](Cn2cncn2)C(=O)c3ccc(cc3)Br)Sc4ccccc4
c1ccc(cc1)[C@@H]([C@H](Cn2cncn2)C(=O)c3ccc(cc3)Br)Sc4ccccc4
c1ccc(cc1)[C@H]([C@H](Cn2cncn2)C(=O)c3ccc(cc3)Br)Sc4ccccc4
CC(=O)Oc1ccc2c(c1)O[C@]3(CCC[C@@H]3C24CCCC4)c5ccc(cc5OC(=O)C)OC(=O)C
c1cc(cc(c1)I)C(=O)Nc2ccc(cc2)c3cn4ccsc4n3
Cc1ccn2cc(nc2c1)c3cccc(c3)NC(=O)c4cccc(c4)I
COc1ccc(cc1OC)CC(=O)Nc2cccc(c2)c3csc(n3)c4ccccc4
CCOc1cc(cc(c1OCC)OCC)C(=O)Nc2ccc(cc2)c3csc(n3)C
Cc1ccc(cc1)[C@@H]([C@@H](Cn2cncn2)C(=O)c3ccccc3)Sc4ccc(cc4)C
Cc1ccc(cc1)[C@H]([C@@H](Cn2cncn2)C(=O)c3ccccc3)Sc4ccc(cc4)C
Cc1ccc(cc1)[C@@H]([C@H](Cn2cncn2)C(=O)c3ccccc3)Sc4ccc(cc4)C
Cc1ccc(cc1)[C@H]([C@H](Cn2cncn2)C(=O)c3ccccc3)Sc4ccc(cc4)C
c1ccc(cc1)[C@@H]([C@@H](Cn2cncn2)C(=O)c3ccccc3)Sc4ccc(cc4)Cl
c1ccc(cc1)[C@H]([C@@H](Cn2cncn2)C(=O)c3ccccc3)Sc4ccc(cc4)Cl
c1ccc(cc1)[C@@H]([C@H](Cn2cncn2)C(=O)c3ccccc3)Sc4ccc(cc4)Cl
c1ccc(cc1)[C@H]([C@H](Cn2cncn2)C(=O)c3ccccc3)Sc4ccc(cc4)Cl
c1ccc(cc1)[C@@H]([C@@H](Cn2cncn2)C(=O)c3ccc(cc3)Cl)Sc4ccccc4
c1ccc(cc1)[C@H]([C@@H](Cn2cncn2)C(=O)c3ccc(cc3)Cl)Sc4ccccc4
```

```
c1ccc(cc1)[C@@H]([C@H](Cn2cncn2)C(=O)c3ccc(cc3)Cl)Sc4ccccc4
c1ccc(cc1)[C@H]([C@H](Cn2cncn2)C(=O)c3ccc(cc3)Cl)Sc4ccccc4
COc1ccc(cc1)C(=O)[C@@H](Cn2cncn2)[C@@H](c3ccccc3)Sc4ccccc4
COc1ccc(cc1)C(=O)[C@H](Cn2cncn2)[C@@H](c3ccccc3)Sc4ccccc4
COc1ccc(cc1)C(=O)[C@@H](Cn2cncn2)[C@H](c3ccccc3)Sc4ccccc4
COc1ccc(cc1)C(=O)[C@H](Cn2cncn2)[C@H](c3ccccc3)Sc4ccccc4
c1ccc(cc1)[C@@H]([C@@H](Cn2cncn2)C(=O)c3ccc(cc3)Br)Sc4ccccc4
c1ccc(cc1)[C@H]([C@@H](Cn2cncn2)C(=O)c3ccc(cc3)Br)Sc4ccccc4
c1ccc(cc1)[C@@H]([C@H](Cn2cncn2)C(=O)c3ccc(cc3)Br)Sc4ccccc4
c1ccc(cc1)[C@H]([C@H](Cn2cncn2)C(=O)c3ccc(cc3)Br)Sc4ccccc4
CC(=O)Oc1ccc2c(c1)O[C@]3(CCC[C@@H]3C24CCCC4)c5ccc(cc5OC(=O)C)OC(=O)C
CC(=O)Oc1ccc2c(c1)O[C@@]3(CCC[C@H]3C24CCCC4)c5ccc(cc5OC(=O)C)OC(=O)C
CC(=O)Oc1ccc2c(c1)O[C@@]3(CCC[C@@H]3C24CCCC4)c5ccc(cc5OC(=O)C)OC(=O)C
CCOc1ccc(cc1)NS(=O)(=O)c2ccc(cc2)c3ccc(cc3)C
c1cc(ccc1c2csc3n2c(nn3)SCC(=O)Nc4ccc(cc4)F)Cl
COc1ccc(cc1)NC(=O)CCSc2nnc3n2c(cs3)c4ccc(cc4)Cl
CC(C)(C)c1ccc(cc1)NC(=O)CCSc2nnc3n2c(cs3)c4ccccc4
c1ccc2c(c1)nc(s2)c3ccc(cc3)NC(=O)CCSc4nnc5n4cccc5
C=CCn1c(=O)c2c(csc2nc1SCC(=O)NC[C@@H]3CCCO3)c4ccccc4Cl
C=CCn1c(=O)c2c(csc2nc1SCC(=O)N3CCOCC3)c4ccccc4Cl
C[C@H]1CCc2c(sc3c2c(=O)n(c(n3)SCC(=O)Nc4cccc(c4)C(=O)C)CC=C)C1
C[C@@H]1CCc2c(sc3c2c(=O)n(c(n3)SCC(=O)Nc4cccc(c4)C(=O)C)CC=C)C1
CCOc1ccc(cc1)NC(=O)CCSc2nc3c(c4c(s3)CCCC4)c(=O)n2CC=C
c1ccc(cc1)n2c(=O)c3c4c(sc3nc2SCC(=O)NC[C@H]5CCCO5)CCCC4
c1ccc(cc1)n2c(=O)c3c4c(sc3nc2SCC(=O)NC[C@@H]5CCCO5)CCCC4
C[C@H]1CCc2c(sc3c2c(=O)n(c(n3)SCC(=O)Nc4cccc(c4)C(=O)C)c5ccccc5)C1
C[C@@H]1CCc2c(sc3c2c(=O)n(c(n3)SCC(=O)Nc4cccc(c4)C(=O)C)c5ccccc5)C1
CC(=O)c1cccc(c1)NC(=O)CSc2nc3c(c4c(s3)CCC4)c(=O)n2c5ccccc5
CC(C)Oc1ccc(cc1I)c2cc(=O)c3c(o2)cc(cc3OC)OC
CC(=O)c1c(cc(cc1OC(=O)c2ccc(c(c2)I)OC)OC)OC
CC(=O)c1c(cc(c(c1OC(=O)c2ccc(cc2)OC)I)OC)OC
```

```
CC(C)Oc1cc(c(c(c1C(=O)C)OC(=O)c2ccc(cc2)OC)I)OC(C)C
c1ccc(c(c1)NC(=O)CSc2nnc(n2N)c3ccc(cc3Cl)Cl)F
CCc1c(nc(s1)N(c2ccc(cc2)C(=O)OC)C(=O)CC)c3ccc(cc3)OCC
CCOc1ccccc1NC(=O)CSc2nc3c(c4c(s3)C[C@H](CC4)C)c(=O)n2C
CCOc1ccccc1NC(=O)CSc2nc3c(c4c(s3)C[C@@H](CC4)C)c(=O)n2C
COc1ccc(cc1OC)CCNC(=O)CSc2nc(cs2)c3ccc(cc3)Cl
COc1cccc(c1)C(=O)Nc2cccc(c2)NC(=O)c3cc(c(cc3Cl)F)F
Cc1ccc(cc1)NC(=O)Cc2c(n(c3c2cc(cc3)OC)C(=O)c4ccc(cc4)Cl)C
COc1ccc(cc1)c2nnc(n2CC=C)SCC(=O)NCc3cc(ccc3O)C(=O)c4ccccc4
CCn1c(c(c(n1)C)NC(=O)CSc2nnc(n2c3ccccc3)c4ccc(cc4)OC)C
Cc1c(nc(s1)N(c2ccc(cc2OC)OC)C(=O)C3CCCC3)c4ccccc4
Cc1c(nc(s1)N(c2ccccc2OC)C(=O)CCc3ccccc3)c4ccccc4
Cc1c(nc(s1)N(c2cccc(c2)OC)C(=O)CCc3ccccc3)c4ccc(cc4)OC
C[C@H](C(=O)Nc1nccs1)Sc2nnc(n2c3ccccc3)COc4cccc5c4cccc5
Cc1c(nc(s1)N(c2ccc(cc2)F)C(=O)CCc3ccccc3)c4ccc(cc4)OC
Cc1c(c2cc(ccc2n1C(=O)c3ccc(cc3)Cl)OC)CC(=O)Nc4nc5c(s4)CCCC5
CCCC(=O)N(c1ccc(cc1OC)OC)c2nc(c(s2)C)c3ccc(cc3)Cl
COC(=O)c1ccccc1NC(=O)COc2ccc(cc2)C3CCCCC3
C[C@@H](c1ccccc1)NC(=O)CSc2nnc(n2Cc3ccccc3)c4ccc(cc4)OC
C[C@H](c1ccccc1)NC(=O)CSc2nnc(n2Cc3ccccc3)c4ccc(cc4)OC
Cc1cccc(c1N2C(=O)c3ccccc3N[C@@]24c5ccccc5N(C4=O)CCN6C(=O)c7ccccc7C6=O)C
COc1ccccc1NC(=O)c2ccccc2NC(=O)C(c3ccccc3)c4ccccc4
CCN(CC)c1ccc(cc1)NC(=O)c2ccccc2OCC(=O)N3CCc4c3cccc4
Cc1c(cccc1Cl)NC(=O)CSc2nnc(n2CC=C)[C@H](c3ccccc3)O
Cc1c(cccc1Cl)NC(=O)c2ccccc2OCC(=O)Nc3ccccc3OC(C)C
c1cc(ccc1c2csc(n2)SCC(=O)Nc3ccc(c(c3)Cl)N4CCOCC4)F
c1ccc(c(c1)C(=O)NC2CCCC2)NC(=O)[C@H]3CC(=O)N(C3)c4ccc(cc4)Cl
c1ccc(c(c1)C(=O)NC2CCCC2)NC(=O)[C@@H]3CC(=O)N(C3)c4ccc(cc4)Cl
CCc1ccc(cc1)N2C[C@H](CC2=O)C(=O)Nc3ccccc3C(=O)NC4CCCC4
CCc1ccc(cc1)N2C[C@@H](CC2=O)C(=O)Nc3ccccc3C(=O)NC4CCCC4
CCc1ccc(cc1)N2C[C@H](CC2=O)C(=O)Nc3ccccc3C(=O)Nc4c(cccc4C)C
```

```
CCc1ccc(cc1)N2C[C@@H](CC2=O)C(=O)Nc3ccccc3C(=O)Nc4c(cccc4C)C
CCc1ccc(cc1)N2C[C@H](CC2=O)C(=O)Nc3ccccc3C(=O)Nc4ccccc4C
CCc1ccc(cc1)N2C[C@@H](CC2=O)C(=O)Nc3ccccc3C(=O)Nc4ccccc4C
c1ccc(cc1)c2c(c[nH]n2)[C@@H]3C(=C(N(C4=C3C(=O)CCC4)c5cccc(c5)C(F)(F)F)N)C#N
Cc1cc(c(cc1COc2ccc(cc2)Br)[C@H]3c4c(n[nH]c4OC(=C3C#N)N)C)C
Cc1cc(c(cc1COc2ccc(cc2)Br)[C@@H]3c4c(n[nH]c4OC(=C3C#N)N)C)C
CCn1c2ccccc2[nH+]c1N[C@H]3CC(=O)N(C3=O)c4ccccc4I
Cc1c2c([nH]n1)OC(=C([C@H]2c3ccc(c(c3)COc4ccc(cc4Cl)Br)OC)C#N)N
Cc1c2c([nH]n1)OC(=C([C@@H]2c3ccc(c(c3)COc4ccc(cc4Cl)Br)OC)C#N)N
Cc1cc(c(cc1COc2ccc(cc2Cl)F)[C@H]3c4c(n[nH]c4OC(=C3C#N)N)C)C
Cc1cc(c(cc1COc2ccc(cc2Cl)F)[C@@H]3c4c(n[nH]c4OC(=C3C#N)N)C)C
c1ccc(cc1)c2c3c([nH]n2)C(=O)N([C@@H]3c4ccccc4F)c5cccc(c5)C(F)(F)F
CCOc1cc(ccc1OCC(=C)C)/C=C\2/C(=O)OC(=N2)c3ccccc3Br
CC(=C)COc1ccc(cc1OC)/C=C\2/C(=O)OC(=N2)c3ccc(cc3)I
CC(=C)COc1ccccc1/C=C\2/C(=O)OC(=N2)c3ccc(cc3)I
CC(=C)COc1ccccc1/C=C\2/C(=O)OC(=N2)c3cccc(c3)I
CCOc1cc(cc(c1OCC)OCC)C(=O)Nc2cccc(c2)c3nc4c(o3)cccn4
Cc1ccc(cc1C)NC(=O)COc2ccc(cc2OC)[C@H]3C(=C(OC4=C3C(=O)CC(C4)(C)C)N)C#N
Cc1ccc(cc1C)NC(=O)COc2ccc(cc2OC)[C@@H]3C(=C(OC4=C3C(=O)CC(C4)(C)C)N)C#N
COc1cc(ccc1OCC(=O)Nc2ccc(cc2)C(=O)OC)/C=C\3/C(=O)OC(=N3)c4ccccc4
CC(=C)COc1c(cc(cc1Br)/C=C\2/C(=O)OC(=N2)c3ccc(cc3)[N+](=O)[O-])OC
CCOc1cc(cc(c1OCC)OCC)C(=O)Nc2cccc(c2)c3nc4ccccc4o3
CC(=C)COc1c(cc(cc1Br)/C=C\2/C(=O)OC(=N2)c3cccs3)OC
c1cc(cc(c1)[N+](=O)[O-])c2ccc(o2)C(=O)Nc3ccc(cc3)NC(=O)c4ccc(cc4Cl)Cl
CC[C@@H](C)c1ccc2c(c1)nc(o2)c3ccc(cc3)NC(=O)c4cc(c(c(c4)OC)OC)OC
CC[C@H](C)c1ccc2c(c1)nc(o2)c3ccc(cc3)NC(=O)c4cc(c(c(c4)OC)OC)OC
Cc1cc(c2c(c1)nc(o2)c3cc(ccc3Cl)NC(=O)c4cc(c(c(c4)OC)OC)OC)C
CCOC(=O)c1c2c(sc1NC(=S)NC(=O)c3c(cn(n3)C)Cl)C[C@H](CC2)C
COc1ccc(cc1Br)c2nc3cc(ccc3o2)NC(=O)c4ccc(o4)I
Cc1ccc(cc1)OCC(=O)Nc2ccc3c(c2)nc(o3)c4ccc(c(c4)Br)OC
Cc1ccc(cc1C)C(=O)Nc2ccc3c(c2)nc(o3)c4ccc(c(c4)Br)OC
```

```
COc1ccc(cc1)c2nc3cc(ccc3o2)NC(=O)c4ccc(o4)c5ccc(cc5)Cl
COc1ccc(cc1C[NH+]2CCN(CC2)C(=O)c3cccnc3)Br
CCOC(=O)c1c2c(sc1NC(=O)Cn3c(c(c(n3)C(F)(F)F)Cl)C)CCCC2
CCOC(=O)c1c2c(sc1NC(=O)c3c(c4nc(cc(n4n3)C(F)(F)F)c5ccco5)Cl)CCCC2
c1cc(ccc1c2cc(n3c(n2)c(c(n3)C(=O)N4CCN(CC4)c5ccc(cc5)F)Cl)C(F)(F)F)F
COc1ccc(cc1OC)c2cc(n3c(n2)c(c(n3)C(=O)Nc4cccc5c4cccc5)Cl)C(F)(F)F
COc1ccc(cc1OC)c2cc(n3c(n2)c(c(n3)C(=O)Nc4ccc5c(c4)OCO5)Cl)C(F)(F)F
Cc1ccc(cc1)c2cc(n3c(n2)c(cn3)C(=O)Nc4c(n(n(c4=O)c5ccccc5)C)C)C(F)(F)F
CCOc1ccc(cc1)NC(=O)c2c3c(sc2NC(=O)c4ccccc4)C[C@H](CC3)C
CCOc1ccc(cc1)NC(=O)c2c3c(sc2NC(=O)c4ccccc4Cl)CCC3
Cc1ccc(cc1)[C@@H]2C3([C@H](C4(O2)C(=O)c5ccccc5C4=O)c6cccc(c6)Cl)C(=O)c7ccccc7C3=O
Cc1ccc(cc1)[C@H]2C3([C@@H](C4(O2)C(=O)c5ccccc5C4=O)c6cccc(c6)Cl)C(=O)c7ccccc7C3=O
Cc1ccc(cc1)[C@@H]2C3([C@@H](C4(O2)C(=O)c5ccccc5C4=O)c6cccc(c6)Cl)C(=O)c7ccccc7C3=O
Cc1c(c(n(n1)c2ccc(cc2)I)O)/C=C\3/C(=NN(C3=O)c4ccc(cc4)Cl)C
Cc1c(c(n(n1)c2ccc(cc2)I)O)/C=C\3/C(=NN(C3=O)c4cccc(c4)Cl)C
CCOc1cc(cc(c1OC)I)C2C3=C(CC(CC3=O)(C)C)OC4=C2C(=O)CC(C4)(C)C
c1ccc(cc1)COc2ccccc2/C=C\3/C(=O)NN(C3=O)c4ccc(cc4)I
COc1cc(ccc1OC(=O)c2cc(c(c(c2)OC)OC)OC)C(=S)N3CCCCC3
Cc1ccc2cccc(c2n1)OC(=O)c3cc(ccc3Cl)I
CCOC(=O)C1=C(N=c2n(c(=O)/c(=C/c3ccc(o3)Br)/s2)[C@H]1c4ccccc4Cl)C
CCOC(=O)C1=C(N=c2n(c(=O)/c(=C/c3ccc(o3)Br)/s2)[C@@H]1c4ccccc4Cl)C
CCOc1ccc(cc1Br)C(=O)Nc2ccc3c(c2)nc(o3)c4ccccc4F
c1cc2cccnc2c(c1)OC(=O)c3cc(ccc3Cl)I
COc1cc(cc(c1OC)OC)C(=O)Nc2ccc(c(c2)c3nc4ccccc4o3)Cl
CCOC(=O)C1=C(N=c2n(c(=O)/c(=C/c3cccs3)/s2)[C@H]1c4ccc(c(c4)OC)OC)C
CCOC(=O)C1=C(N=c2n(c(=O)/c(=C/c3cccs3)/s2)[C@@H]1c4ccc(c(c4)OC)OC)C
CCOc1ccc(cc1)NCc2ccc(cc2)OCc3ccccc3F
COc1ccc(cc1)NCc2ccc(c(c2)OC)OCc3cccc(c3)F
CCOc1cccc-2c1N(C(c3c2c(=S)ss3)(C)C)C(=O)c4ccc(cc4)OC
CC1(c2c(c(=S)ss2)-c3cccc(c3N1C(=O)c4ccc(cc4)F)OC)C
c1cc(oc1)C(=O)Nc2ccc(cc2)NC(=O)c3ccc(cc3)I
```

```
c1ccc(cc1)C(=O)COC(=O)c2ccccc2NC(=O)c3ccc(cc3Cl)Cl
CCOC(=O)c1c2c(sc1NC(=O)c3ccccc3Cl)CN(CC2)C(=O)OCC
Cc1cc(c(n1c2ccc(cc2)OC(=O)C)C)C=C3C(=O)N(C(=S)N(C3=O)c4ccccc4)c5ccccc5
CC1=C([C@H](NC(=O)N1)c2ccc(c(c2)OC)O)C(=O)OCC3CCCCC3
CC1=C([C@@H](NC(=O)N1)c2ccc(c(c2)OC)O)C(=O)OCC3CCCCC3
CC1=C([C@H](NC(=O)N1)c2ccccc2OC(C)C)C(=O)OCC3CCCCC3
CC1=C([C@@H](NC(=O)N1)c2ccccc2OC(C)C)C(=O)OCC3CCCCC3
CC1=C([C@H](NC(=O)N1)c2cc(ccc2OC)OC)C(=O)OCc3ccc4c(c3)OCO4
CC1=C([C@@H](NC(=O)N1)c2cc(ccc2OC)OC)C(=O)OCc3ccc4c(c3)OCO4
CCN(CC)c1ccc(cc1)[C@H]2C(=C(NC(=O)N2)C)C(=O)OCC3CCCCC3
CCN(CC)c1ccc(cc1)[C@@H]2C(=C(NC(=O)N2)C)C(=O)OCC3CCCCC3
COc1ccc(cc1)C(=O)N/C(=C/c2cc(c(cc2[N+](=O)[O-])OC)OC)/C(=O)N3CCCCC3
COc1ccc(cc1)C(=O)N/C(=C/c2cc(c(cc2[N+](=O)[O-])OC)OC)/C(=O)N3CCCCC3
COc1ccccc1NC(=O)c2cc3nc(cc(n3n2)C(F)(F)F)c4ccc(cc4)Br
Cc1ccc(c(c1)NC(=O)CSc2nc3c(c4c(s3)CCC4)c(=O)n2c5ccccc5)C
Cc1ccc(cc1)n2nc3ccc(cc3n2)NC(=O)c4ccc(o4)c5cccc(c5)[N+](=O)[O-]
Cc1ccc(cc1)n2nc3ccc(cc3n2)NC(=O)c4ccc(o4)c5ccc(cc5)Cl
COc1ccc(cc1Br)c2nc3cc(ccc3o2)NC(=O)/C=C/c4ccccc4
CC1(c2c(c(=S)ss2)-c3cccc(c3N1C(=O)c4cccc(c4)OC)OC)C
c1ccc(cc1)C(=O)Nc2ccc(cc2)NC(=O)c3ccc(o3)c4cc(ccc4Cl)Cl
Cc1ccc(cc1)N2C(=O)/C(=C/c3ccc(c(c3)OC)OCc4ccc(cc4Cl)Cl)/C(=O)NC2=O
CCOc1cc(ccc1OC)/C=c\2/c(=O)n3c(=NC(=C([C@H]3c4ccc(c(c4)OCC)OC)C(=O)OCC)C)s2
CCOc1cc(ccc1O)/C=c\2/c(=O)n3c(=NC(=C([C@H]3c4ccc(c(c4)OCC)OC)C(=O)OCC)C)s2
CCOc1cc(ccc1O)/C=c\2/c(=O)n3c(=NC(=C([C@@H]3c4ccc(c(c4)OCC)OC)C(=O)OCC)C)s2
CCOC(=O)c1ccc(cc1)NC(=O)CSc2nnnn2c3cccc(c3)Cl
CCN1C(=O)C(=Cc2ccc(o2)c3ccc(cc3)C(=O)OC(C)C)C(=O)N(C1=S)CC
CC(C)OC(=O)c1ccc(cc1)NC(=O)CSc2nnnn2c3ccc(cc3)Cl
Cc1ccc(cc1Br)N2C(=O)[C@@H]3[C@@H](C2=O)C4(c5ccccc5C3c6c4cccc6)C
Cc1ccc(cc1Br)N2C(=O)[C@H]3[C@H](C2=O)C4(c5ccccc5C3c6c4cccc6)C
Cc1ccc(cc1Br)N2C(=O)[C@H]3[C@@H](C2=O)C4(c5ccccc5C3c6c4cccc6)C
CC12c3ccccc3C(c4c1cccc4)[C@H]5[C@H]2C(=O)N(C5=O)c6ccc(c(c6)C(F)(F)F)Cl
```

CC12c3ccccc3C(c4c1cccc4)[C@@H]5[C@H]2C(=O)N(C5=O)c6ccc(c(c6)C(F)(F)F)Cl
CC12c3ccccc3C(c4c1cccc4)[C@H]5[C@@H]2C(=O)N(C5=O)c6ccc(c(c6)C(F)(F)F)Cl
CC12c3ccccc3C(c4c1cccc4)[C@@H]5[C@@H]2C(=O)N(C5=O)c6ccc(c(c6)C(F)(F)F)Cl
CC1=Cc2cc(ccc2O[C@]13C=Cc4c5ccccc5ccc4O3)[N+](=O)[O-]
CC1=Cc2cc(ccc2O[C@@]13C=Cc4c5ccccc5ccc4O3)[N+](=O)[O-]
Cc1ccc(cc1)CN(CC(=O)Nc2cc(ccc2OC)Cl)S(=O)(=O)c3ccccc3
CCN(CC)c1ccc(cc1)/C=C\2/C(=O)N(C(=S)N2)c3ccc(cc3)Br
COc1cc(cc(c1OCc2ccccc2C#N)CC=C)/C=C\3/C(=O)N(C(=S)N3)c4ccccc4
CCOC(=O)C1=C(N=c2n(c(=O)/c(=C/c3ccco3)/s2)[C@H]1c4cccc(c4)OC)c5ccccc5
CCOC(=O)C1=C(N=c2n(c(=O)/c(=C/c3ccco3)/s2)[C@@H]1c4cccc(c4)OC)c5ccccc5
CCOc1cc(cc(c1O)Cl)/C=C\2/C(=NN(C2=O)c3ccc(cc3)C(=O)OCC)C(F)(F)F
c1ccc(c(c1)COc2ccc(cc2Br)C3C4=C(CCCC4=O)OC5=C3C(=O)CCC5)F
CCOC(=O)COc1cc(c(cc1OC)/C=C\2/C(=NN(C2=O)c3ccccc3)C)Br
CCOc1cc(c(cc1OCC(=O)OC)Br)/C=C\2/C(=NN(C2=O)c3ccccc3)C
CCOc1cc(cc(c1OCC(=O)OCC)Br)/C=C\2/C(=NN(C2=O)c3ccccc3)C
COc1cc(cc(c1OCc2ccccn2)Br)/C=C/3\C(=O)NC(=S)N3
CC(C)Oc1cccc(c1)C(=O)NC(=S)Nc2ccc(cc2)NC(=O)c3cccc(c3)Cl
c1ccc(c(c1)C(=O)NC(=S)Nc2ccc(cc2)NC(=O)c3cccc(c3)Cl)F
c1cc(cc(c1)Cl)C(=O)Nc2ccc(cc2)NC(=S)NC(=O)c3ccc(cc3)Br
COc1cc(ccc1OC(=O)c2ccco2)C=C3C(=NN(C3=O)c4ccc(cc4)Cl)C(F)(F)F
CCOc1ccc(cc1)C(=O)C2=C(C(=O)N([C@H]2c3cccc(c3)O)Cc4ccccc4)[O-]
CCOc1ccc(cc1)C(=O)C2=C(C(=O)N([C@@H]2c3cccc(c3)O)Cc4ccccc4)[O-]
c1ccc2c(c1)cccc2CSc3nnc(s3)NC(=O)COc4ccc(cc4)F
CCOc1ccc(cc1)C(=O)Nc2nnc(s2)SCc3cccc4c3cccc4
Cc1cc(cc(c1)OCC(=O)Nc2nnc(s2)SCc3cccc4c3cccc4)C
CC(C)(C)c1ccc(cc1)OCC(=O)Nc2nnc(s2)SCc3ccc(cc3)F
CC1([C@H]([C@@H]1C(=O)Nc2nnc(s2)SCc3ccc(cc3)F)C=C(Cl)Cl)C
CC1([C@@H]([C@H]1C(=O)Nc2nnc(s2)SCc3ccc(cc3)F)C=C(Cl)Cl)C
COc1ccc(cc1)OCC(=O)Nc2nnc(s2)SCc3ccc(cc3)Cl
Cc1ccc(cc1C)O[C@@H](C)C(=O)Nc2nnc(s2)SCc3ccc(cc3)Cl
Cc1ccc(cc1C)O[C@H](C)C(=O)Nc2nnc(s2)SCc3ccc(cc3)Cl

```
Cc1ccc(cc1C)OCC(=O)Nc2nnc(s2)SCc3ccc(cc3)Cl
CC1([C@H]([C@H]1C(=O)Nc2nnc(s2)SCc3ccccc3F)C=C(Cl)Cl)C
CC1([C@H]([C@@H]1C(=O)Nc2nnc(s2)SCc3ccccc3F)C=C(Cl)Cl)C
CC1([C@@H]([C@H]1C(=O)Nc2nnc(s2)SCc3ccccc3F)C=C(Cl)Cl)C
CC1([C@@H]([C@@H]1C(=O)Nc2nnc(s2)SCc3ccccc3F)C=C(Cl)Cl)C
c1ccc(c(c1)CSc2nnc(s2)NC(=O)[C@H]3COc4ccccc4O3)Cl
c1ccc(c(c1)CSc2nnc(s2)NC(=O)[C@@H]3COc4ccccc4O3)Cl
CCOc1ccc(cc1)C(=O)C2=C(C(=O)N([C@H]2c3ccc(cc3)C)CCc4ccccc4)[O-]
CCOc1ccc(cc1)C(=O)C2=C(C(=O)N([C@@H]2c3ccc(cc3)C)CCc4ccccc4)[O-]
CCOc1ccc(cc1)C(=O)C2=C(C(=O)N([C@H]2c3ccc(c(c3)OC)O)CCc4ccccc4)[O-]
CCOc1ccc(cc1)C(=O)C2=C(C(=O)N([C@@H]2c3ccc(c(c3)OC)O)CCc4ccccc4)[O-]
CCOc1ccc(cc1)C(=O)C2=C(C(=O)N([C@H]2c3ccncc3)CCc4ccccc4)[O-]
C[C@@]12CC[C@@](C1(C)C)(CC2=O)C(=O)Oc3c(cc(c4c3nccc4)Br)Br
Cn1c(nnc1SCC(=O)c2ccc(cc2)c3ccccc3)c4ccc5c(c4)OCO5
Cn1c(nnc1SCC(=O)c2ccc(cc2)c3ccccc3)c4cc(c(c(c4)OC)OC)OC
Cn1c(nnc1SCC(=O)c2ccc(cc2Cl)Cl)c3cc(c(c(c3)OC)OC)OC
Cn1c(nnc1SCC(=O)c2ccc(cc2)Cl)c3c(c4ccccc4s3)Cl
CCOc1cccc(c1)C(=O)C2=C(C(=O)N([C@H]2c3cccc(c3)OC)Cc4ccncc4)[O-]
CCOc1cccc(c1)C(=O)C2=C(C(=O)N([C@@H]2c3cccc(c3)OC)Cc4ccncc4)[O-]
CCOc1cccc(c1)C(=O)C2=C(C(=O)N([C@H]2c3cccc(c3)O)Cc4ccncc4)[O-]
COc1cccc(c1)C(=O)C2=C(C(=O)N([C@H]2c3ccc(cc3)O)CCc4ccccc4)[O-]
COc1cccc(c1)C(=O)C2=C(C(=O)N([C@@H]2c3ccc(cc3)O)CCc4ccccc4)[O-]
Cc1ccc(c(c1)C(=O)C2=C(C(=O)N([C@H]2c3ccc(cc3)C(C)C)c4nnc(s4)C)[O-])C
CCOc1cc(ccc1O)[C@@H]2C(=C(C(=O)N2CCc3ccccc3)[O-])C(=O)c4cc(ccc4C)C
Cc1ccc(c(c1)C(=O)C2=C(C(=O)N([C@@H]2c3cccc(c3)Cl)CCCn4ccnc4)[O-])C
Cc1ccc(cc1C)NC(=S)N[C@H](C(Cl)(Cl)Cl)NC(=O)c2ccccc2F
CC(=O)c1ccc(cc1)NC(=S)N[C@@H](C(Cl)(Cl)Cl)NC(=O)c2cccc3c2cccc3
CC(=O)c1ccc(cc1)NC(=S)N[C@H](C(Cl)(Cl)Cl)NC(=O)c2cccc3c2cccc3
CCOC(=O)c1ccc(cc1)NC(=S)N[C@@H](C(Cl)(Cl)Cl)NC(=O)c2cccc(c2)Cl
CCOC(=O)c1ccc(cc1)NC(=S)N[C@H](C(Cl)(Cl)Cl)NC(=O)c2cccc(c2)Cl
COc1ccc(cc1OC)[C@@H]2c3c(=O)[nH]c(nc3NC4=C2C(=O)CCC4)SCc5ccccc5F
```

```
CC1(CC2=C([C@@H](c3c(nc(nc3N2)SC)N)c4ccc(c(c4)OC)OC)C(=O)C1)C
CC1(CC2=C([C@H](c3c(=O)[nH]c(nc3N2)SCc4ccccc4F)c5cccc(c5OC)OC)C(=O)C1)C
CC1(CC2=C([C@@H](c3c(=O)[nH]c(nc3N2)SCc4ccccc4F)c5cccc(c5OC)OC)C(=O)C1)C
Cc1cccc(c1)[C@H]2c3c(=O)[nH]c(nc3NC4=C2C(=O)CC(C4)(C)C)SCc5ccccc5F
Cc1cccc(c1)[C@@H]2c3c(=O)[nH]c(nc3NC4=C2C(=O)CC(C4)(C)C)SCc5ccccc5F
CCSc1[nH]c2c(c(=O)n1)[C@@H](C3=C(N2)CCCC3=O)c4ccc(cc4)OCc5ccccc5
CCSc1[nH]c2c(c(=O)n1)[C@H](C3=C(N2)CCCC3=O)c4ccc(cc4)OCc5ccccc5
CCN(CC)c1ccc(cc1)[C@@H]2c3c([nH]c(nc3=O)SC)NC4=C2C(=O)CC(C4)(C)C
CC1(CC2=C([C@H](c3c(=O)[nH]c(nc3N2)SC)c4ccc(c(c4)Cl)Cl)C(=O)C1)C
CC1(CC2=C([C@@H](c3c(=O)[nH]c(nc3N2)SC)c4ccc(c(c4)Cl)Cl)C(=O)C1)C
CCSc1[nH]c2c(c(=O)n1)[C@@H](C3=C(N2)CC(CC3=O)(C)C)c4ccc(c(c4)OC)OC
CCOC(=O)c1c2cc(ccc2oc1c3ccc(cc3)OC)OC(=O)c4ccc(c(c4)OC)OC
C[C@@H](C(=O)Nc1c(c2c(s1)CCCC2)C(=O)NCc3ccco3)Oc4ccccc4F
C[C@H](C(=O)Nc1c(c2c(s1)CCCC2)C(=O)NCc3ccco3)Oc4ccccc4F
c1cc(cnc1)CNC(=O)c2c3c(sc2NC(=O)c4ccc(cc4)Br)CCCC3
c1cc(cnc1)CNC(=O)c2c3c(sc2NC(=O)COc4ccc(cc4Cl)Cl)CCCC3
Cc1c(sc(n1)N2[C@@H](C(=C(C2=O)[O-])C(=O)c3ccc(cc3)OC)c4ccccc4)C(=O)C
Cc1c(sc(n1)N2[C@H](C(=C(C2=O)O)C(=O)c3ccc(cc3)OC)c4ccc(c(c4)OC)OCc5ccccc5)C(=O)C
Cc1c(sc(n1)N2[C@@H](C(=C(C2=O)O)C(=O)c3ccc(cc3)OC)c4ccc(c(c4)OC)OCc5ccccc5)C(=O)C
Cc1cccn2c1nc3c(c2=O)cc(c([n+]3C4CCCCC4)N)S(=O)(=O)c5ccccc5
Cc1cccn2c1nc3c(c2=O)cc(c([n+]3Cc4ccccc4)N)S(=O)(=O)c5ccccc5
CCOc1ccc(cc1)c2cc(c3c(c(sc3n2)C(=O)Nc4ccc(cc4)OC)N)C(F)(F)F
CCOC(=O)c1c(nc(s1)N2[C@H](C(=C(C2=O)O)C(=O)c3ccc(c(c3)F)C)c4ccc(c(c4)OC)OC)C
CCOC(=O)c1c(nc(s1)N2[C@@H](C(=C(C2=O)O)C(=O)c3ccc(c(c3)F)C)c4ccc(c(c4)OC)OC)C
c1cc(cnc1)CNC(=O)c2c3c(sc2NC(=O)c4ccc(cc4)Cl)CCCC3
Cc1cccn2c1nc3c(c2=O)cc(c([n+]3C4CCCC4)N)S(=O)(=O)c5ccc(cc5)Cl
CCOc1ccc(cc1C)C(=O)C2=C(C(=O)N([C@@H]2c3ccc(cc3)C)CCc4ccc(cc4)OC)[O-]
Cc1ccc(cc1)[C@H]2C(=C(C(=O)N2CCc3ccc(cc3)OC)O)C(=O)c4cccs4
Cc1ccc(cc1)[C@@H]2C(=C(C(=O)N2CCc3ccc(cc3)OC)O)C(=O)c4cccs4
CC1=C([C@H](N2C(=O)CCSC2=N1)c3ccc(cc3Cl)Cl)C(=O)OCC(C)C
CC1=C([C@@H](N2C(=O)CCSC2=N1)c3ccc(cc3Cl)Cl)C(=O)OCC(C)C
```

```
CC1=C([C@H](N2C(=O)CCSC2=N1)c3ccc(cc3Cl)Cl)C(=O)OCc4ccccc4
CC1=C([C@@H](N2C(=O)CCSC2=N1)c3ccc(cc3Cl)Cl)C(=O)OCc4ccccc4
CC1=C([C@H](N2C(=O)CCSC2=N1)c3cccc(c3)Oc4ccccc4)C(=O)OCC(C)C
CC1=C([C@@H](N2C(=O)CCSC2=N1)c3cccc(c3)Oc4ccccc4)C(=O)OCC(C)C
CCOc1ccc(cc1OC)[C@H]2C(=C(N=C3N2C(=O)CCS3)C)C(=O)OCc4ccccc4
CCOc1ccc(cc1OC)[C@@H]2C(=C(N=C3N2C(=O)CCS3)C)C(=O)OCc4ccccc4
Cc1cccn2c1nc3c(c2=O)cc(c([n+]3Cc4ccco4)N)S(=O)(=O)c5ccc(cc5)Cl
CCC[n+]1c(c(cc2c1nc3c(cccn3c2=O)C)S(=O)(=O)c4ccc(cc4)C)N
CCOC(=O)C1CCN(CC1)C2=NC(=O)/C(=C/c3cn(nc3c4cccs4)c5ccccc5)/S2
CCOC(=O)c1c(nc(s1)N2[C@H](C(=C(C2=O)[O-])C(=O)c3ccc(c(c3)F)C)c4cccnc4)C
CCOC(=O)c1c(nc(s1)N2[C@@H](C(=C(C2=O)[O-])C(=O)c3ccc(c(c3)F)C)c4cccnc4)C
CCOc1ccc(cc1)C(=O)C2=C(C(=O)N([C@H]2c3ccc(c(c3)OC)OCC(C)C)Cc4ccco4)[O-]
CC(C)CCOc1ccc(cc1OC)[C@@H]2C(=C(C(=O)N2Cc3ccco3)[O-])C(=O)c4ccc(cc4)F
CCOc1ccc(cc1)C(=O)C2=C(C(=O)N([C@H]2c3ccc(c(c3)OC)OCC(C)C)C[C@H]4CCCO4)[O-]
CCOc1ccc(cc1)C(=O)C2=C(C(=O)N([C@@H]2c3ccc(c(c3)OC)OCC(C)C)C[C@H]4CCCO4)[O-]
CCOc1ccc(cc1)C(=O)C2=C(C(=O)N([C@H]2c3ccc(c(c3)OC)OCC(C)C)C[C@@H]4CCCO4)[O-]
CCOc1ccc(cc1)C(=O)C2=C(C(=O)N([C@@H]2c3ccc(c(c3)OC)OCC(C)C)C[C@@H]4CCCO4)[O-]
CCOc1ccc(cc1OC)[C@H]2C(=C(C(=O)N2C[C@H]3CCCO3)[O-])C(=O)c4ccc(cc4)F
CCOc1ccc(cc1OC)[C@@H]2C(=C(C(=O)N2C[C@H]3CCCO3)[O-])C(=O)c4ccc(cc4)F
CCOc1ccc(cc1OC)[C@@H]2C(=C(C(=O)N2C[C@@H]3CCCO3)[O-])C(=O)c4ccc(cc4)F
Cc1ccc(o1)C(=O)C2=C(C(=O)N([C@H]2c3cccc(c3)Oc4ccccc4)C[C@H]5CCCO5)O
Cc1ccc(o1)C(=O)C2=C(C(=O)N([C@@H]2c3cccc(c3)Oc4ccccc4)C[C@H]5CCCO5)O
Cc1ccc(o1)C(=O)C2=C(C(=O)N([C@H]2c3cccc(c3)Oc4ccccc4)C[C@@H]5CCCO5)O
Cc1ccc(o1)C(=O)C2=C(C(=O)N([C@@H]2c3cccc(c3)Oc4ccccc4)C[C@@H]5CCCO5)O
Cc1ccc(cc1)C(=O)C2=C(C(=O)N([C@H]2c3cccc(c3)Oc4ccccc4)C[C@H]5CCCO5)[O-]
Cc1ccc(cc1)C(=O)C2=C(C(=O)N([C@@H]2c3cccc(c3)Oc4ccccc4)C[C@H]5CCCO5)[O-]
Cc1ccc(cc1)C(=O)C2=C(C(=O)N([C@H]2c3cccc(c3)Oc4ccccc4)C[C@@H]5CCCO5)[O-]
Cc1ccc(cc1)C(=O)C2=C(C(=O)N([C@@H]2c3cccc(c3)Oc4ccccc4)C[C@@H]5CCCO5)[O-]
Cc1ccc(o1)C(=O)C2=C(C(=O)N([C@H]2c3ccc(c(c3)OC)OC)Cc4ccco4)[O-]
Cc1ccc(o1)C(=O)C2=C(C(=O)N([C@@H]2c3ccc(c(c3)OC)OC)Cc4ccco4)[O-]
CCOc1ccc(cc1)C(=O)C2=C(C(=O)N([C@H]2c3ccc(c(c3)OC)OC)Cc4ccco4)[O-]
```

```
COc1ccc(cc1OC)[C@H]2C(=C(C(=O)N2Cc3ccco3)[O-])C(=O)c4ccc(cc4)F
COc1ccc(cc1OC)[C@@H]2C(=C(C(=O)N2Cc3ccco3)[O-])C(=O)c4ccc(cc4)F
Cc1ccc(o1)C(=O)C2=C(C(=O)N([C@H]2c3ccc(c(c3)OC)OC)C[C@H]4CCCO4)[O-]
Cc1ccc(o1)C(=O)C2=C(C(=O)N([C@@H]2c3ccc(c(c3)OC)OC)C[C@H]4CCCO4)[O-]
Cc1ccc(o1)C(=O)C2=C(C(=O)N([C@H]2c3ccc(c(c3)OC)OC)C[C@@H]4CCCO4)[O-]
Cc1ccc(o1)C(=O)C2=C(C(=O)N([C@@H]2c3ccc(c(c3)OC)OC)C[C@@H]4CCCO4)[O-]
Cc1ccc(o1)C(=O)C2=C(C(=O)N([C@@H]2c3cccc(c3)Br)C[C@H]4CCCO4)O
Cc1ccc(o1)C(=O)C2=C(C(=O)N([C@H]2c3cccc(c3)Br)C[C@@H]4CCCO4)O
Cc1ccc(o1)C(=O)C2=C(C(=O)N([C@@H]2c3cccc(c3)Br)C[C@@H]4CCCO4)O
CCOc1ccc(cc1)C(=O)C2=C(C(=O)N([C@H]2c3ccc(cc3)Br)Cc4ccco4)[O-]
CCOc1ccc(cc1)C(=O)C2=C(C(=O)N([C@H]2c3ccc(cc3)Br)C[C@H]4CCCO4)[O-]
CCOc1ccc(cc1)C(=O)C2=C(C(=O)N([C@@H]2c3ccc(cc3)Br)C[C@H]4CCCO4)[O-]
CCOc1ccc(cc1)C(=O)C2=C(C(=O)N([C@H]2c3ccc(cc3)Br)C[C@@H]4CCCO4)[O-]
CCOc1ccc(cc1)C(=O)C2=C(C(=O)N([C@@H]2c3ccc(cc3)Br)C[C@@H]4CCCO4)[O-]
CCOc1ccc(cc1)C(=O)C2=C(C(=O)N([C@H]2c3ccc(cc3)F)Cc4ccco4)[O-]
CCOc1ccc(cc1)C(=O)C2=C(C(=O)N([C@@H]2c3ccc(cc3)F)Cc4ccco4)[O-]
c1ccc(cc1)COc2ccc(cc2)[C@@H]3C(=C(C(=O)N3Cc4ccco4)[O-])C(=O)c5ccc(cc5)Cl
c1ccc(cc1)COc2ccc(cc2)[C@H]3C(=C(C(=O)N3C[C@H]4CCCO4)[O-])C(=O)c5ccc(cc5)Cl
c1ccc(cc1)COc2ccc(cc2)[C@@H]3C(=C(C(=O)N3C[C@H]4CCCO4)[O-])C(=O)c5ccc(cc5)Cl
c1ccc(cc1)COc2ccc(cc2)[C@H]3C(=C(C(=O)N3C[C@@H]4CCCO4)[O-])C(=O)c5ccc(cc5)Cl
c1ccc(cc1)COc2ccc(cc2)[C@@H]3C(=C(C(=O)N3C[C@@H]4CCCO4)[O-])C(=O)c5ccc(cc5)Cl
CCOc1ccc(cc1)C(=O)C2=C(C(=O)N([C@H]2c3ccc(cc3)OCc4ccccc4)C[C@H]5CCCO5)[O-]
CCOc1ccc(cc1)C(=O)C2=C(C(=O)N([C@@H]2c3ccc(cc3)OCc4ccccc4)C[C@H]5CCCO5)[O-]
CCOc1ccc(cc1)C(=O)C2=C(C(=O)N([C@H]2c3ccc(cc3)OCc4ccccc4)C[C@@H]5CCCO5)[O-]
CCOc1ccc(cc1)C(=O)C2=C(C(=O)N([C@@H]2c3ccc(cc3)OCc4ccccc4)C[C@@H]5CCCO5)[O-]
c1ccc(cc1)COc2ccc(cc2)[C@H]3C(=C(C(=O)N3C[C@H]4CCCO4)[O-])C(=O)c5ccc(cc5)F
c1ccc(cc1)COc2ccc(cc2)[C@@H]3C(=C(C(=O)N3C[C@H]4CCCO4)[O-])C(=O)c5ccc(cc5)F
c1ccc(cc1)COc2ccc(cc2)[C@H]3C(=C(C(=O)N3C[C@@H]4CCCO4)[O-])C(=O)c5ccc(cc5)F
c1ccc(cc1)COc2ccc(cc2)[C@@H]3C(=C(C(=O)N3C[C@@H]4CCCO4)[O-])C(=O)c5ccc(cc5)F
CCOc1ccc(cc1)C(=O)C2=C(C(=O)N([C@H]2c3ccc(cc3)Cl)C[C@H]4CCCO4)[O-]
CCOc1ccc(cc1)C(=O)C2=C(C(=O)N([C@@H]2c3ccc(cc3)Cl)C[C@H]4CCCO4)[O-]
```

```
CCOc1ccc(cc1)C(=O)C2=C(C(=O)N([C@H]2c3ccc(cc3)Cl)C[C@@H]4CCCO4)[O-]
CCOc1ccc(cc1)C(=O)C2=C(C(=O)N([C@@H]2c3ccc(cc3)Cl)C[C@@H]4CCCO4)[O-]
CCOc1ccc(cc1)C(=O)C2=C(C(=O)N([C@H]2c3ccc(cc3)C(C)(C)C)Cc4ccco4)[O-]
CCOc1ccc(cc1)C(=O)C2=C(C(=O)N([C@@H]2c3ccc(cc3)C(C)(C)C)Cc4ccco4)[O-]
CCOC(=O)c1ccc(cc1)NC(=O)CCSc2nc3c(c(s3)C)C)c(=O)n2CC=C
c1ccc(cc1)n2c(nnc2SCC(=O)c3ccc(cc3)Cl)COc4ccc5c(c4)OCO5
c1ccc(cc1)C(=O)CSc2nnc(n2c3ccccc3)COc4ccc5c(c4)OCO5
CCn1c(nnc1SCC(=O)c2ccc(cc2)Cl)COc3ccc4c(c3)OCO4
Cc1c(sc(n1)N2[C@H](C(=C(C2=O)O)C(=O)c3cc4ccccc4o3)c5ccccc5)C(=O)C
Cc1ccc(cc1)[C@H]2C(=C(C(=O)N2c3nc(c(s3)C(=O)C)C)O)C(=O)c4cc5ccccc5o4
Cc1ccc(cc1)[C@@H]2C(=C(C(=O)N2c3nc(c(s3)C(=O)C)C)O)C(=O)c4cc5ccccc5o4
Cc1c(sc(n1)N2[C@@H](C(=C(C2=O)O)C(=O)c3cc4ccccc4o3)c5ccc(cc5)F)C(=O)C
Cc1c(sc(n1)N2[C@H](C(=C(C2=O)O)C(=O)c3cc4ccccc4o3)c5ccc(c(c5)Cl)Cl)C(=O)C
Cc1c(sc(n1)N2[C@@H](C(=C(C2=O)O)C(=O)c3cc4ccccc4o3)c5ccc(c(c5)Cl)Cl)C(=O)C
Cc1c(sc(n1)N2[C@H](C(=C(C2=O)O)C(=O)c3cc4ccccc4o3)c5ccc(cc5)Br)C(=O)C
Cc1c(sc(n1)N2[C@@H](C(=C(C2=O)O)C(=O)c3cc4ccccc4o3)c5ccc(cc5)Br)C(=O)C
Cc1c(sc(n1)N2[C@H](C(=C(C2=O)O)C(=O)c3cc4ccccc4o3)c5ccc(cc5)O)C(=O)C
Cc1c(sc(n1)N2[C@@H](C(=C(C2=O)O)C(=O)c3cc4ccccc4o3)c5ccc(cc5)O)C(=O)C
CCOc1ccc(cc1OC)[C@H]2C(=C(C(=O)N2c3nc(c(s3)C(=O)C)C)O)C(=O)c4cc5ccccc5o4
Cc1c(sc(n1)N2[C@H](C(=C(C2=O)O)C(=O)c3cc4ccccc4o3)c5ccc(cc5)OCc6ccccc6)C(=O)C
Cc1c(sc(n1)N2[C@H](C(=C(C2=O)O)C(=O)c3cc4ccccc4o3)c5ccc(c(c5)OC)OCc6ccccc6)C(=O)C
Cc1c(sc(n1)N2[C@@H](C(=C(C2=O)O)C(=O)c3cc4ccccc4o3)c5ccc(c(c5)OC)OCc6ccccc6)C(=O)C
Cc1c(sc(n1)N2[C@H](C(=C(C2=O)O)C(=O)c3cc4ccccc4o3)c5ccc(cc5)N(C)C)C(=O)C
Cc1c(sc(n1)N2[C@@H](C(=C(C2=O)O)C(=O)c3cc4ccccc4o3)c5ccc(cc5)N(C)C)C(=O)C
Cc1c(sc(n1)N2[C@H](C(=C(C2=O)O)C(=O)c3cc4ccccc4o3)c5cccc(c5)Cl)C(=O)C
Cc1c(sc(n1)N2[C@@H](C(=C(C2=O)O)C(=O)c3cc4ccccc4o3)c5cccc(c5)Cl)C(=O)C
c1ccc(cc1)Oc2cccc(c2)[C@H]3C(=C(C(=O)N3CCCn4ccnc4)O)C(=O)c5cc6ccccc6o5
c1ccc(cc1)Oc2cccc(c2)[C@@H]3C(=C(C(=O)N3CCCn4ccnc4)O)C(=O)c5cc6ccccc6o5
CC(C)(C)c1ccc(cc1)[C@H]2C(=C(C(=O)N2Cc3ccco3)O)C(=O)c4cc5ccccc5o4
CC(C)(C)c1ccc(cc1)[C@@H]2C(=C(C(=O)N2Cc3ccco3)O)C(=O)c4cc5ccccc5o4
c1ccc(cc1)Oc2cccc(c2)[C@H]3C(=C(C(=O)N3Cc4ccco4)O)C(=O)c5cc6ccccc6o5
```

```
c1ccc(cc1)Oc2cccc(c2)[C@@H]3C(=C(C(=O)N3Cc4ccco4)O)C(=O)c5cc6ccccc6o5
COc1cc(ccc1OCc2ccccc2)[C@H]3C(=C(C(=O)N3Cc4ccco4)O)C(=O)c5cc6ccccc6o5
COc1cc(ccc1OCc2ccccc2)[C@@H]3C(=C(C(=O)N3Cc4ccco4)O)C(=O)c5cc6ccccc6o5
c1ccc(cc1)n2cc(c(n2)c3cc4ccccc4o3)/C=C\5/C(=O)N=C(S5)N6CCOCC6
CCOC(=O)C1CCN(CC1)C2=NC(=O)/C(=C/c3cn(nc3c4cc5ccccc5o4)c6ccccc6)/S2
CCOc1ccc(cc1)[C@@H]2C(=C(C(=O)N2C[C@H]3CCCO3)[O-])C(=O)c4ccc5c(c4)OCCO5
CCOc1ccc(cc1)[C@H]2C(=C(C(=O)N2C[C@@H]3CCCO3)[O-])C(=O)c4ccc5c(c4)OCCO5
CCOc1ccc(cc1)[C@@H]2C(=C(C(=O)N2C[C@@H]3CCCO3)[O-])C(=O)c4ccc5c(c4)OCCO5
c1cc(cnc1)[C@@H]2C(=C(C(=O)N2C[C@H]3CCCO3)[O-])C(=O)c4ccc5c(c4)OCCO5
c1cc(cnc1)[C@H]2C(=C(C(=O)N2C[C@@H]3CCCO3)[O-])C(=O)c4ccc5c(c4)OCCO5
c1cc(cnc1)[C@@H]2C(=C(C(=O)N2C[C@@H]3CCCO3)[O-])C(=O)c4ccc5c(c4)OCCO5
C[C@@H]1Cc2cc(ccc2O1)C(=O)C3=C(C(=O)N([C@@H]3c4cccc(c4)OC)C[C@@H]5CCCO5)[O-]
C[C@H]1Cc2cc(ccc2O1)C(=O)C3=C(C(=O)N([C@@H]3c4ccc(c(c4)OC)OCCC(C)C)C[C@@H]5CCCO5)[O-]
C[C@@H]1Cc2cc(ccc2O1)C(=O)C3=C(C(=O)N([C@H]3c4ccc(c(c4)OC)OCc5ccccc5)C[C@@H]6CCCO6)O
C[C@H]1Cc2cc(ccc2O1)C(=O)C3=C(C(=O)N([C@H]3c4cccc(c4)Oc5ccccc5)Cc6ccco6)[O-]
C[C@@H]1Cc2cc(ccc2O1)C(=O)C3=C(C(=O)N([C@H]3c4cccc(c4)Oc5ccccc5)Cc6ccco6)[O-]
C[C@H]1Cc2cc(ccc2O1)C(=O)C3=C(C(=O)N([C@@H]3c4cccc(c4)Oc5ccccc5)Cc6ccco6)[O-]
C[C@@H]1Cc2cc(ccc2O1)C(=O)C3=C(C(=O)N([C@@H]3c4cccc(c4)Oc5ccccc5)Cc6ccco6)[O-]
C[C@H]1Cc2cc(ccc2O1)C(=O)C3=C(C(=O)N([C@H]3c4cc(c(c(c4)OC)OC)OC)Cc5ccco5)[O-]
C[C@@H]1Cc2cc(ccc2O1)C(=O)C3=C(C(=O)N([C@H]3c4cc(c(c(c4)OC)OC)OC)Cc5ccco5)[O-]
C[C@H]1Cc2cc(ccc2O1)C(=O)C3=C(C(=O)N([C@@H]3c4cc(c(c(c4)OC)OC)OC)Cc5ccco5)[O-]
C[C@@H]1Cc2cc(ccc2O1)C(=O)C3=C(C(=O)N([C@@H]3c4cc(c(c(c4)OC)OC)OC)Cc5ccco5)[O-]
C[C@H]1Cc2cc(ccc2O1)C(=O)C3=C(C(=O)N([C@H]3c4ccc(c(c4)OC)OCCC(C)C)Cc5ccco5)[O-]
C[C@@H]1Cc2cc(ccc2O1)C(=O)C3=C(C(=O)N([C@H]3c4ccc(c(c4)OC)OCCC(C)C)Cc5ccco5)[O-]
C[C@H]1Cc2cc(ccc2O1)C(=O)C3=C(C(=O)N([C@@H]3c4ccc(c(c4)OC)OCCC(C)C)Cc5ccco5)[O-]
C[C@@H]1Cc2cc(ccc2O1)C(=O)C3=C(C(=O)N([C@@H]3c4ccc(c(c4)OC)OCCC(C)C)Cc5ccco5)[O-]
CC(C)CCOc1ccc(cc1OC)[C@H]2C(=C(C(=O)N2Cc3ccco3)O)C(=O)c4ccc5c(c4)OCCO5
C[C@@H]1Cc2cc(ccc2O1)C(=O)C3=C(C(=O)N([C@H]3c4ccc(c(c4)OC)OCc5ccccc5)Cc6ccco6)O
C[C@H]1Cc2cc(ccc2O1)C(=O)C3=C(C(=O)N([C@@H]3c4ccc(c(c4)OC)OCc5ccccc5)Cc6ccco6)O
C[C@@H]1Cc2cc(ccc2O1)C(=O)C3=C(C(=O)N([C@@H]3c4ccc(c(c4)OC)OCc5ccccc5)Cc6ccco6)O
COc1cc(ccc1OCc2ccccc2)[C@@H]3C(=C(C(=O)N3Cc4ccco4)O)C(=O)c5ccc6c(c5)OCCO6
```

```
c1cc(sc1)[C@H]2C(=C(C(=O)N2C[C@H]3CCCO3)[O-])C(=O)c4ccc5c(c4)OCCO5
c1cc(sc1)[C@@H]2C(=C(C(=O)N2C[C@H]3CCCO3)[O-])C(=O)c4ccc5c(c4)OCCO5
c1cc(sc1)[C@H]2C(=C(C(=O)N2C[C@@H]3CCCO3)[O-])C(=O)c4ccc5c(c4)OCCO5
c1cc(sc1)[C@@H]2C(=C(C(=O)N2C[C@@H]3CCCO3)[O-])C(=O)c4ccc5c(c4)OCCO5
Cc1ccc2c(c1)sc(n2)N3[C@H](C(=C(C3=O)O)C(=O)c4cc5ccccc5o4)c6ccccc6
Cc1ccc2c(c1)sc(n2)N3[C@@H](C(=C(C3=O)O)C(=O)c4cc5ccccc5o4)c6ccccc6
Cc1ccc2c(c1)sc(n2)N3[C@H](C(=C(C3=O)[O-])C(=O)c4cc5ccccc5o4)c6cccs6
Cc1ccc2c(c1)sc(n2)N3[C@@H](C(=C(C3=O)[O-])C(=O)c4cc5ccccc5o4)c6cccs6
CCOc1cc(ccc1O)[C@@H]2C(=C(C(=O)N2c3nc4ccc(cc4s3)C)O)C(=O)c5cc6ccccc6o5
Cc1ccc2c(c1)sc(n2)N3[C@H](C(=C(C3=O)O)C(=O)c4cc5ccccc5o4)c6ccc(cc6)OC
Cc1ccc2c(c1)sc(n2)N3[C@@H](C(=C(C3=O)O)C(=O)c4cc5ccccc5o4)c6ccc(cc6)OC
Cc1ccc2c(c1)sc(n2)N3[C@H](C(=C(C3=O)O)C(=O)c4cc5ccccc5o4)c6ccccc6F
Cc1ccc2c(c1)sc(n2)N3[C@@H](C(=C(C3=O)O)C(=O)c4cc5ccccc5o4)c6ccccc6F
Cc1ccc2c(c1)sc(n2)N3[C@H](C(=C(C3=O)O)C(=O)c4cc5ccccc5o4)c6cccc(c6)O
Cc1ccc2c(c1)sc(n2)N3[C@@H](C(=C(C3=O)O)C(=O)c4cc5ccccc5o4)c6cccc(c6)O
Cc1cc(c2c(c1)sc(n2)N3[C@H](C(=C(C3=O)O)C(=O)c4cc5ccccc5o4)c6ccccc6)C
Cc1cc(c2c(c1)sc(n2)N3[C@@H](C(=C(C3=O)O)C(=O)c4cc5ccccc5o4)c6ccccc6)C
Cc1cc(c2c(c1)sc(n2)N3[C@H](C(=C(C3=O)O)C(=O)c4cc5ccccc5o4)c6ccc(cc6)F)C
Cc1cc(c2c(c1)sc(n2)N3[C@@H](C(=C(C3=O)O)C(=O)c4cc5ccccc5o4)c6ccc(cc6)F)C
Cc1cc(c2c(c1)sc(n2)N3[C@H](C(=C(C3=O)O)C(=O)c4cc5ccccc5o4)c6cccs6)C
Cc1cc(c2c(c1)sc(n2)N3[C@@H](C(=C(C3=O)O)C(=O)c4cc5ccccc5o4)c6cccs6)C
Cc1cc(c2c(c1)sc(n2)N3[C@H](C(=C(C3=O)O)C(=O)c4cc5ccccc5o4)c6ccc(cc6)O)C
Cc1cc(c2c(c1)sc(n2)N3[C@@H](C(=C(C3=O)O)C(=O)c4cc5ccccc5o4)c6ccc(cc6)O)C
CCOc1cc(ccc1O)[C@H]2C(=C(C(=O)N2c3nc4c(cc(cc4s3)C)C)O)C(=O)c5cc6ccccc6o5
CCOc1cc(ccc1O)[C@@H]2C(=C(C(=O)N2c3nc4c(cc(cc4s3)C)C)O)C(=O)c5cc6ccccc6o5
Cc1cc(c2c(c1)sc(n2)N3[C@H](C(=C(C3=O)O)C(=O)c4cc5ccccc5o4)c6cc(c(c(c6)OC)OC)OC)C
Cc1cc(c2c(c1)sc(n2)N3[C@@H](C(=C(C3=O)O)C(=O)c4cc5ccccc5o4)c6cc(c(c(c6)OC)OC)OC)C
CCOc1ccc2c(c1)sc(n2)N3[C@H](C(=C(C3=O)O)C(=O)c4cc5ccccc5o4)c6ccc(cc6)C
CCOc1ccc2c(c1)sc(n2)N3[C@@H](C(=C(C3=O)O)C(=O)c4cc5ccccc5o4)c6ccc(cc6)C
CCOc1ccc2c(c1)sc(n2)N3[C@H](C(=C(C3=O)O)C(=O)c4cc5ccccc5o4)c6ccc(cc6)F
CCOc1ccc2c(c1)sc(n2)N3[C@@H](C(=C(C3=O)O)C(=O)c4cc5ccccc5o4)c6ccc(cc6)F
```

```
CCOc1ccc2c(c1)sc(n2)N3[C@H](C(=C(C3=O)O)C(=O)c4cc5ccccc5o4)c6ccc(cc6)O
CCOc1ccc2c(c1)sc(n2)N3[C@@H](C(=C(C3=O)O)C(=O)c4cc5ccccc5o4)c6ccc(cc6)O
CCOc1ccc2c(c1)sc(n2)N3[C@H](C(=C(C3=O)O)C(=O)c4cc5ccccc5o4)c6ccc(c(c6)OC)OC
CCOc1ccc2c(c1)sc(n2)N3[C@@H](C(=C(C3=O)O)C(=O)c4cc5ccccc5o4)c6ccc(c(c6)OC)OC
CC1(CC2=C([C@@H](Nc3c2c4ccccc4cc3)c5ccc(c(c5)[N+](=O)[O-])Sc6nccn6C)C(=O)C1)C
c1cc(ccc1[C@H]2C(=C(C(=O)N2c3nccs3)[O-])C(=O)c4ccc(cc4)[N+](=O)[O-])F
c1cc(ccc1[C@@H]2C(=C(C(=O)N2c3nccs3)[O-])C(=O)c4ccc(cc4)[N+](=O)[O-])F
CC1=C([C@H](C(=C(N1)SCc2cccc(c2)[N+](=O)[O-])C#N)c3ccc(cc3)Cl)C(=O)C
CC1=NC2=C([C@@H](C1C(=O)OC)c3cccc(c3)Oc4ccccc4)C(=O)C[C@@H](C2)c5cccs5
CC1=NC2=C([C@H](C1C(=O)OC)c3cccc(c3)Oc4ccccc4)C(=O)C[C@@H](C2)c5cccs5
CC1=NC2=C([C@@H](C1C(=O)OC)c3cccc(c3)Oc4ccccc4)C(=O)C[C@H](C2)c5cccs5
CC1=NC2=C([C@H](C1C(=O)OC)c3cccc(c3)Oc4ccccc4)C(=O)C[C@H](C2)c5cccs5
c1ccc(cc1)CCN2[C@H](C(=C(C2=O)O)C(=O)c3cccs3)c4ccc(cc4)Cl
c1ccc(cc1)CCN2[C@@H](C(=C(C2=O)O)C(=O)c3cccs3)c4ccc(cc4)Cl
c1ccc(cc1)CCN2[C@@H](C(=C(C2=O)O)C(=O)c3cccs3)c4ccc(cc4)Br
CC1=NC2=C([C@@H](C1C(=O)OC)c3ccc(c(c3OC)OC)OC)C(=O)C[C@@H](C2)c4cccs4
CC1=NC2=C([C@H](C1C(=O)OC)c3ccc(c(c3OC)OC)OC)C(=O)C[C@@H](C2)c4cccs4
CC1=NC2=C([C@@H](C1C(=O)OC)c3ccc(c(c3OC)OC)OC)C(=O)C[C@H](C2)c4cccs4
CC1=NC2=C([C@H](C1C(=O)OC)c3ccc(c(c3OC)OC)OC)C(=O)C[C@H](C2)c4cccs4
Cn1c2c(c(=O)n(c1=O)C)n3cc(n(c3n2)c4ccc(c(c4)OC)OC)c5ccccc5
Cn1c2c(c(=O)n(c1=O)C)n(cn2)Cc3nc(nc(n3)N4CCOCC4)N
Cn1c2c(c(=O)n(c1=O)C)n3cc(n(c3n2)c4cccc5c4cccc5)c6ccc(cc6)Br
c1ccc(cc1)CCN2[C@H](C(=C(C2=O)O)C(=O)c3cccs3)c4ccccc4Cl
c1ccc(cc1)CCN2[C@@H](C(=C(C2=O)O)C(=O)c3cccs3)c4ccccc4Cl
CCOC(=O)CN1[C@H](C(=C(C1=O)[O-])C(=O)c2ccc(cc2)Cl)c3ccc(cc3)Br
CCOC(=O)CN1[C@@H](C(=C(C1=O)[O-])C(=O)c2ccc(cc2)Cl)c3ccc(cc3)Br
c1ccc(cc1)Cn2c3ccc(cc3nc2SCC(=O)c4ccccc4)Br
Cc1ccc(cc1)N2[C@H](C(=C(C2=O)O)C(=O)/C=C/c3ccccc3)c4ccc(cc4)OC
Cc1ccc(cc1)N2[C@@H](C(=C(C2=O)O)C(=O)/C=C/c3ccccc3)c4ccc(cc4)OC
CC1=C([C@H](NC(=O)N1)c2cccc(c2)Br)C(=O)OCC3CCCCC3
CC1=C([C@@H](NC(=O)N1)c2cccc(c2)Br)C(=O)OCC3CCCCC3
```

```
CCOC(=O)C1=C(N=c2n(c(=O)/c(=C/c3ccc(o3)C)/s2)[C@H]1c4ccccc4Cl)C
CCOC(=O)C1=C(N=c2n(c(=O)/c(=C/c3ccc(o3)C)/s2)[C@@H]1c4ccccc4Cl)C
COc1cc(cc(c1O)Br)[C@@H]2C3=C(CCCC3=O)c4c5ccccc5ccc4N2
Cc1ccc(cc1)n2c(c(c(n2)C)C=C3C(=O)N(C(=S)N(C3=O)c4ccccc4)c5ccccc5)O
CCOC(=O)c1c2c(sc1NC(=O)c3ccccc3Br)CN(CC2)C(=O)OCC
C[C@@H](C(=O)Nc1cccc(c1)c2[nH]c3cc(ccc3n2)C(=O)c4ccccc4)Oc5ccccc5
C[C@H](C(=O)Nc1cccc(c1)c2[nH]c3cc(ccc3n2)C(=O)c4ccccc4)Oc5ccccc5
CCOc1ccc(cc1)n2nc3ccc(cc3n2)NC(=O)c4cc(ccc4Cl)Br
c1ccc(cc1)c2nc3cc(ccc3o2)NC(=O)c4cc(ccc4Cl)Br
Cc1ccc(o1)c2c3c(nc(c2C#N)SCC(=O)c4ccc(cc4)Cl)CCCC3
Cc1ccc(cc1)NC(=O)COc2ccc(cc2OC)[C@H]3C(=C(OC4=C3C(=O)CCC4)N)C#N
Cc1ccc(cc1)NC(=O)COc2ccc(cc2OC)[C@@H]3C(=C(OC4=C3C(=O)CCC4)N)C#N
c1cc(cc(c1)NC(=O)[C@H]2CC(=O)N(C2)c3ccc(cc3)Br)C(F)(F)F
c1cc(cc(c1)NC(=O)[C@@H]2CC(=O)N(C2)c3ccc(cc3)Br)C(F)(F)F
c1ccc(cc1)N2[C@H](c3c(n[nH]c3C2=O)c4ccc(cc4)Br)c5ccc(cc5)[N+](=O)[O-]
c1ccc(cc1)N2[C@@H](c3c(n[nH]c3C2=O)c4ccc(cc4)Br)c5ccc(cc5)[N+](=O)[O-]
c1ccc(cc1)c2c3c([nH]n2)C(=O)N([C@H]3c4ccccc4)c5cccc(c5)C(F)(F)F
c1ccc(cc1)c2c3c([nH]n2)C(=O)N([C@@H]3c4ccccc4)c5cccc(c5)C(F)(F)F
c1ccc(cc1)c2c3c([nH]n2)C(=O)N([C@H]3c4ccc(cc4)Cl)c5cccc(c5)C(F)(F)F
c1ccc(cc1)c2c3c([nH]n2)C(=O)N([C@@H]3c4ccc(cc4)Cl)c5cccc(c5)C(F)(F)F
COc1ccc(cc1OC)[C@H]2c3c(n[nH]c3C(=O)N2c4cccc(c4)C(F)(F)F)c5ccccc5
COc1ccc(cc1OC)[C@@H]2c3c(n[nH]c3C(=O)N2c4cccc(c4)C(F)(F)F)c5ccccc5
CCOc1cc(cc(c1OCC)OCC)C(=O)Nc2ccc(c(c2)Cl)NC(=O)c3ccco3
CC1=C([C@H](NC(=O)N1)c2ccc(c(c2)Cl)Cl)C(=O)OCc3ccc(cc3)OC
CC1=C([C@@H](NC(=O)N1)c2ccc(c(c2)Cl)Cl)C(=O)OCc3ccc(cc3)OC
CC1=C([C@H](NC(=O)N1)c2cc(c(c(c2)Br)O)OC)C(=O)OC3CCCCCC3
CC1=C([C@@H](NC(=O)N1)c2cc(c(c(c2)Br)O)OC)C(=O)OC3CCCCCC3
CC1=C([C@H](NC(=O)N1)c2cc(c(c(c2)Br)O)OC)C(=O)OCC(F)(F)F
CC1=C([C@@H](NC(=O)N1)c2cc(c(c(c2)Br)O)OC)C(=O)OCC(F)(F)F
CC1=C([C@H](NC(=O)N1)c2cccc(c2)Br)C(=O)OCc3ccc(cc3)OC
Cc1ccc(cc1)c2cc(c(c(n2)SCC(=O)Nc3ccccc3)C#N)c4ccc(cc4)OC
```

```
CCOc1ccc(cc1)NC(=O)C2=C(NC(=S)N[C@@H]2c3ccc(cc3Cl)Cl)C
Cn1c(cc(n1)C(=O)Nc2cccc(c2)Br)C(=O)Nc3cccc(c3)Br
CC(C)[C@@H](C(=O)Nc1ccccc1Br)NC(=O)c2ccccc2NC(=O)c3ccc(cc3)OC
Cc1ccc(c(c1)C)NC(=O)[C@@H](C(C)C)NC(=O)c2ccccc2NC(=O)c3ccc(cc3)OC
Cc1ccc(c(c1)C)NC(=O)[C@H](C(C)C)NC(=O)c2ccccc2NC(=O)c3ccc(cc3)OC
CC(C)[C@H](C(=O)Nc1ccc2ccccc2c1)NC(=O)c3ccccc3NC(=O)c4ccc(cc4)OC
Cc1ccc(cc1)NC(=O)[C@H](Cc2ccc(cc2)O)NC(=O)c3ccccc3NC(=O)c4ccc(cc4)OC
COc1cc2ccccc2cc1C(=O)Nc3ccc(c(c3)c4nc5c(o4)cccn5)Cl
CCOc1ccc2c(c1)sc(n2)NC(=O)c3cc(c(c(c3)OCC)OCC)OCC
CC1=NC2=C([C@@H](C1C(=O)OC[C@H]3CCCO3)c4ccc(c(c4)OC)OCc5ccccc5)C(=O)CC(C2)(C)C
CC1=NC2=C([C@H](C1C(=O)OC[C@H]3CCCO3)c4ccc(c(c4)OC)OCc5ccccc5)C(=O)CC(C2)(C)C
CC1=NC2=C([C@@H](C1C(=O)OC[C@@H]3CCCO3)c4ccc(c(c4)OC)OCc5ccccc5)C(=O)CC(C2)(C)C
c1ccc(cc1)COc2ccc(cc2)OC(=O)CSc3nnc(o3)c4ccc(cc4)F
COc1ccc(cc1)c2cc(n3c(n2)c(c(n3)C(=O)NC4CCCC4)Cl)C(F)(F)F
c1ccc(cc1)N2CCN(CC2)C(=O)c3c(c4nc(cc(n4n3)C(F)(F)F)c5ccc(cc5)Cl)Cl
Cc1ccc(cc1)c2cc(n3c(n2)c(cn3)C(=O)Nc4cccc5c4cccc5)C(F)(F)F
Cc1ccc(cc1)c2cc(n3c(n2)c(cn3)C(=O)Nc4ccc5c(c4)OCO5)C(F)(F)F
CCOc1ccc(cc1)n2nc3ccc(cc3n2)NC(=O)c4ccc5c6c4cccc6CC5
c1cc(cc(c1)Cl)n2nc3ccc(cc3n2)NC(=O)c4ccc5c6c4cccc6CC5
Cc1c(c(n(n1)c2ccc(cc2)Br)O)/C=C\3/C(=NN(C3=O)c4ccc(cc4)I)C
c1ccc(cc1)c2csc(n2)NC(=O)c3ccc(o3)c4cc(ccc4Cl)Cl
c1cc(cc(c1)NC(=O)c2ccc(o2)c3cc(ccc3Cl)Cl)c4nc5c(o4)cccn5
COc1ccc(cc1)n2nc3ccc(cc3n2)NC(=O)c4ccc(o4)c5cc(ccc5Cl)Cl
c1cc(ccc1N2C(=O)/C(=C/c3ccc(o3)c4ccc(c(c4)Cl)Cl)/C(=O)NC2=S)F
c1cc(cc(c1)NC(=O)CSc2nnc(o2)c3ccc(cc3)Br)C(F)(F)F
c1cc(cc(c1)Cl)NC(=O)CSc2nnc(o2)c3ccc(cc3)Br
c1ccc(c(c1)c2nnc(o2)SCC(=O)Nc3cc(cc(c3)Cl)Cl)Cl
Cc1cccc(c1)NC(=O)COc2ccc(cc2OC)[C@H]3C(=C(OC4=C3C(=O)CC(C4)(C)C)N)C#N
Cc1cccc(c1)NC(=O)COc2ccc(cc2OC)[C@@H]3C(=C(OC4=C3C(=O)CC(C4)(C)C)N)C#N
Cc1cccc(c1)NC(=O)COc2ccc(cc2OC)[C@@H]3c4ccc5ccccc5c4OC(=C3C#N)N
COc1cc(cc(c1OC)OC)c2nnc(o2)SCc3ccc(cc3Cl)Cl
```

```
COc1ccc(cc1C(=O)Nc2cccc(c2)c3nc4ccccc4o3)Br
Cc1ccc2c(c1)nc(o2)c3cccc(c3)NC(=O)c4cc(ccc4OC)Br
Cc1ccc2c(c1)oc(n2)c3cccc(c3)NC(=O)c4cc(ccc4OC)Br
Cc1ccc(cc1)n2nc3ccc(cc3n2)NC(=O)/C=C/c4ccc(o4)c5ccc(cc5)Cl
c1ccc(c(c1)c2nnc(o2)SCC(=O)Nc3ccc(cc3)Br)Cl
Cc1ccc(cc1[N+](=O)[O-])NC(=O)CSc2nnc(o2)c3ccccc3Br
CCOc1ccc(cc1)c2cc(c(c(n2)SCC(=O)NNC(=O)Cc3ccccc3)C#N)c4ccccc4
c1ccc(c(c1)c2nnc(o2)SCC(=O)Nc3cccc(c3)C(F)(F)F)Br
c1ccc(c(c1)c2nnc(o2)SCC(=O)Nc3cccc(c3)[N+](=O)[O-])Br
c1ccc(c(c1)c2nnc(o2)SCC(=O)Nc3ccc(cc3)Cl)Br
Cn1c2c(c(=O)n(c1=O)C)n(c(n2)Oc3ccc4c(c3)OCO4)Cc5ccc(cc5Cl)Cl
Cn1c2c(c(=O)n(c1=O)C)n(c(n2)Sc3ccccc3)Cc4ccc(c(c4)Cl)Cl
Cn1c2c(nc1Oc3ccc(c4c3nccc4)Cl)n(c(=O)n(c2=O)Cc5ccc(cc5)F)C
Cn1c2c(c(=O)n(c1=O)C)n(cn2)CC(=O)Nc3ccccc3I
c1cc(cc(c1)Br)c2nc([nH]n2)SCC(=O)Nc3ccc(cc3)Cl
Cc1cccc(c1)NCc2nc([nH]n2)SCC(=O)Nc3c(cc(cc3Cl)Cl)Cl
c1cc(sc1)C(=O)Nc2ccc(c(c2)NC(=O)c3ccc(cc3Cl)Cl)Cl
Cc1cc(c(n1c2ccccc2)C)/C=C\3/C(=O)NC(=O)N(C3=O)c4ccc(cc4)Br
c1cc(cc(c1)Br)N2C(=O)/C(=C/c3cc(c(c(c3)Br)[O-])Br)/C(=NC2=S)[O-]
c1cc(ccc1c2c[nH]c(n2)SCC(=O)Nc3c(cc(cc3Cl)Cl)Cl)Cl
CCOC(=O)c1c(c(sc1NC(=O)CSc2[nH]cc(n2)c3ccc(cc3)Cl)C)C
c1ccc(c(c1)c2nnc(o2)SCC(=O)Nc3ccccc3C(F)(F)F)Br
Cc1ccc(cc1)c2cc(c(c(n2)SCC(=O)Nc3ccccc3OC)C#N)c4ccccc4
CCc1ccccc1NC(=O)CSc2nnc(o2)c3ccccc3Br
COc1ccc(cc1NC(=O)CSc2nnc(o2)c3ccccc3Br)Cl
Cc1ccc(cc1)c2cc(c(c(n2)SCC(=O)Nc3cc(ccc3OC)OC)C#N)c4ccccc4
Cc1ccccc1NC(=O)CSc2nc3c(c4cc(cc(c4[nH]3)Br)Br)nn2
Cc1ccc(c(c1)Br)NC(=O)CSc2nnc(o2)c3cccc(c3)[N+](=O)[O-]
Cc1ccc(cc1[N+](=O)[O-])NC(=O)CSc2nnc(o2)c3ccc(cc3)Br
c1ccc(c(c1)NC(=O)CSc2nnc(o2)c3ccc(cc3)Br)Br
c1cc(ccc1c2nnc(o2)SCC(=O)Nc3ccc(cc3)Br)Br
```

```
Cc1c(c(no1)c2c(cccc2Cl)F)C(=O)N3CCN(CC3)c4cccc(c4)C(F)(F)F
COc1cc(cc(c1OC)OC)c2nnc(o2)c3cc4c5ccccc5ccc4oc3=O
COc1ccc2c(c1)cc(c(=O)o2)c3nnc(s3)Nc4cccc(c4)C(F)(F)F
CCOC(=O)c1ccc(cc1)Nc2nnc(s2)c3cc4cc(ccc4oc3=O)OC
c1cc(cc(c1)F)Nc2nnc(s2)c3cc4cc(ccc4oc3=O)Br
COc1ccc(c(c1)OC)Nc2nnc(s2)c3cc4c5ccccc5ccc4oc3=O
COc1ccc2c(c1)sc3n2cc(n3)c4cc5cc(ccc5oc4=O)Br
CCOC(=O)c1ccc(cc1)Nc2nc(cs2)c3cc4cccc(c4oc3=O)OC
CCOC(=O)c1ccc(cc1)Nc2nc(cs2)c3cc4cc(ccc4oc3=O)Cl
COc1ccc2c(c1)cc(c(=O)o2)c3csc(n3)Nc4cccc(c4)C(F)(F)F
c1cc(cnc1)c2nnc(o2)SCC(=O)Nc3c(cc(cc3Cl)Cl)Cl
COc1ccc(cc1NC[C@@H](Cn2c3ccc(cc3c4c2ccc(c4)Cl)Cl)O)Cl
COc1ccc(cc1NC[C@H](Cn2c3ccc(cc3c4c2ccc(c4)Cl)Cl)O)Cl
c1cc(oc1)CN(CC(=O)Nc2c(cc(cc2Cl)Cl)Cl)C(=O)c3ccc(cc3)Cl
c1ccc(c(c1)C(=O)NN[C@@H]2CC(=O)N(C2=O)c3ccc(cc3)Br)Cl
c1ccc(c(c1)C(=O)NN[C@@H]2CC(=O)N(C2=O)c3ccc(cc3)Br)Br
c1cc(cc(c1)Br)C(=O)NN[C@H]2CC(=O)N(C2=O)c3ccc(cc3)Br
c1ccc(cc1)C(c2ccccc2)C(=O)NN[C@@H]3CC(=O)N(C3=O)c4ccc(cc4)Br
c1cc(cc(c1)Br)C(=O)NN[C@@H]2CC(=O)N(C2=O)c3ccc(cc3)Cl
c1ccc(c(c1)C(=O)NN[C@@H]2CC(=O)N(C2=O)c3ccc(cc3)Cl)I
c1cc(cc(c1)I)C(=O)NN[C@@H]2CC(=O)N(C2=O)c3ccc(cc3)Cl
CC(=O)N(CC1(CCCC1)c2ccc(c(c2)OC)OC)C(=O)c3ccc(cc3)[N+](=O)[O-]
Cc1ccc(cc1)c2cc(c(c(n2)SCC(=O)Nc3ccccc3OC)C#N)c4ccc(cc4)OC
Cc1ccc2cc(c(nc2c1)Cl)C3=NN([C@H](C3)c4ccc(cc4)Br)C(=O)CCC(=O)[O-]
Cc1ccc2cc(c(nc2c1)Cl)C3=NN([C@@H](C3)c4ccc(cc4)Br)C(=O)CCC(=O)[O-]
CCc1cccc(c1N(COC)C(=O)Cn2c3ccccc3nc2Cc4ccccc4)CC
CCc1cccc(c1N(COC)C(=O)Cn2c3ccccc3nc2COc4ccccc4)CC
COC(=O)c1cc(ccc1Cl)c2ccc(o2)C=C3C(=O)OC4(CCCCC4)OC3=O
CCOC(=O)C1=C(N=c2n(c(=O)/c(=C/c3cn(c4c3cccc4)C)/s2)[C@@H]1c5ccc(cc5)OC)C
CC(=O)N1c2ccccc2NC3=C([C@H]1c4ccccc4OCc5ccccc5)C(=O)CC(C3)(C)C
CC(=O)N1c2ccccc2NC3=C([C@@H]1c4ccccc4OCc5ccccc5)C(=O)CC(C3)(C)C
```

```
CCOc1ccc(cc1)c2c3cc(c(cc3c4c(nn(c4n2)c5ccccc5)C)OC)OC
CCc1c2c3cc(c(cc3c(nc2n(n1)c4ccccc4)c5ccc(c(c5)OCC)O)OC)OC
CCc1c2c3cc(c(cc3c(nc2n(n1)c4ccccc4)c5ccc(cc5)OCC)OC)OC
CCc1c2c3cc(c(cc3c(nc2n(n1)c4ccccc4)c5ccc(cc5)N(C)C)OC)OC
CCc1ccc(cc1)N2C(=O)/C(=C/c3cc(n(c3C)c4cc(cc(c4)C)C)C)/C(=NC2=S)[O-]
CCc1ccc(cc1)N2C(=O)/C(=C/c3cc(n(c3C)C4CCCC4)C)/C(=NC2=S)[O-]
CCc1ccc(cc1)N2C(=O)/C(=C/c3cc(n(c3C)c4ccc(cc4)C(=O)OCC)C)/C(=NC2=S)[O-]
c1ccc(cc1)C(c2ccccc2)(C(=O)NN(c3ccccc3)C(=O)c4ccncc4)O
CCOc1cccc(c1)N2C(=O)/C(=C/c3cc(n(c3C)c4ccc(cc4)N(C)C)C)/C(=NC2=S)[O-]
CCOc1c(cc(cc1OC)/C=C\2/C(=O)NN(C2=O)c3ccc(c(c3)Cl)C)[N+](=O)[O-]
COc1ccc(cc1)NC(=O)CSc2nc(c(nn2)c3ccccc3)c4ccccc4
CCOC(=O)c1c(c(sc1NC(=O)c2ccccc2C)C(=O)Nc3ccccc3F)C
CC1=C([C@H](NC(=S)N1)c2cc(c(c(c2)OC)OC)OC)C(=O)Nc3ccccc3OC
CC1=C([C@@H](NC(=S)N1)c2cc(c(c(c2)OC)OC)OC)C(=O)Nc3ccccc3OC
CCOc1ccc(cc1)NC(=O)C2=C(NC(=S)N[C@H]2c3ccc(cc3OC)OC)C
CCOc1ccc(cc1)NC(=O)C2=C(NC(=S)N[C@@H]2c3ccc(cc3OC)OC)C
Cc1c(c(nn1CC(=O)Nc2ccc(cc2C(=O)c3ccccc3)Cl)[N+](=O)[O-])Cl
CN(C)c1ccc(cc1)[C@H]2N(CCS2)C(=O)c3cc4cc(ccc4oc3=O)Br
CN(C)c1ccc(cc1)[C@@H]2N(CCS2)C(=O)c3cc4cc(ccc4oc3=O)Br
CCOc1ccc(cc1)C[NH+](CCc2ccc3c(c2)OCO3)Cc4cc(ccc4O)OC
c1ccc(cc1)[C@@H](CC(=O)Nc2nc3ccc(cc3s2)[N+](=O)[O-])NC(=O)c4ccccc4
c1ccc(cc1)[C@H](CC(=O)Nc2nc3ccc(cc3s2)[N+](=O)[O-])NC(=O)c4ccccc4
COc1cc(cc(c1OC)OC)C(=O)Nc2nc(c(s2)c3ccccc3)c4ccc(cc4)[N+](=O)[O-]
c1ccc(cc1)c2nc3cc(ccc3o2)NC(=S)NC(=O)c4ccc(cc4)[N+](=O)[O-]
c1cc(ccc1c2nc3cc(ccc3o2)NC(=S)NC(=O)c4ccc(cc4)[N+](=O)[O-])F
c1ccc(cc1)n2nc3ccc(cc3n2)NC(=S)NC(=O)c4ccc(cc4)[N+](=O)[O-]
Cc1cc2c(cc1NC(=S)NC(=O)c3ccc(cc3)[N+](=O)[O-])nn(n2)c4ccccc4
Cc1ccc(cc1)n2nc3cc(c(cc3n2)NC(=S)NC(=O)c4ccc(cc4)[N+](=O)[O-])C
Cc1ccc2c(c1)nc(o2)c3cccc(c3C)NC(=S)NC(=O)c4ccc(cc4)[N+](=O)[O-]
Cc1ccc(cc1NC(=S)NC(=O)c2ccc(cc2)[N+](=O)[O-])c3nc4ccccc4o3
Cc1ccc2c(c1)nc(o2)c3ccc(cc3)NC(=S)NC(=O)c4ccc(cc4)[N+](=O)[O-]
```

```
C[C@@H](C(=O)c1ccccc1)OC(=O)c2ccc(cc2)NC(=O)c3ccc(cc3)[N+](=O)[O-]
C[C@H](C(=O)c1ccccc1)OC(=O)c2ccc(cc2)NC(=O)c3ccc(cc3)[N+](=O)[O-]
C[C@@H](C(=O)c1ccccc1)OC(=O)c2ccc(cc2)NC(=O)c3cccc(c3)[N+](=O)[O-]
C[C@H](C(=O)c1ccccc1)OC(=O)c2ccc(cc2)NC(=O)c3cccc(c3)[N+](=O)[O-]
Cc1ccc(cc1)C(=O)Nc2ccc(cc2)C(=O)NNC(=O)c3cc(c(c(c3)OC)OC)OC
CN(C)c1ccc(cc1)NC(=S)NC(=O)c2ccc(cc2)I
Cc1ccc(cc1C)C(=O)NC(=S)Nc2cccc(c2)NC(=O)c3ccccc3Cl
COc1ccc(cc1OC)C(=O)NC(=S)Nc2ccc3c(c2)nn(n3)c4ccccc4
Cc1ccc(cc1)n2nc3ccc(cc3n2)NC(=S)NC(=O)c4ccc(c(c4)OC)OC
CCOc1ccc(cc1)n2nc3ccc(cc3n2)NC(=S)NC(=O)c4ccc(c(c4)OC)OC
Cc1ccc(cc1)n2nc3cc(c(cc3n2)NC(=S)NC(=O)c4ccc(c(c4)OC)OC)C
Cc1cc2c(cc1NC(=S)NC(=O)c3ccc(c(c3)OC)OC)nn(n2)c4ccc(cc4)OC
COc1ccc(cc1OC)C(=O)NC(=S)Nc2ccc3c(c2)nc(o3)c4ccc(cc4)Cl
Cc1ccccc1c2nc3cc(ccc3o2)NC(=S)NC(=O)c4ccc(c(c4)OC)OC
Cc1cccc(c1)c2nc3cc(ccc3o2)NC(=S)NC(=O)c4ccc(c(c4)OC)OC
Cc1ccc(cc1)c2nc3cc(ccc3o2)NC(=S)NC(=O)c4ccc(c(c4)OC)OC
COc1ccc(cc1)c2nc3cc(ccc3o2)NC(=S)NC(=O)c4ccc(c(c4)OC)OC
Cc1ccc(c(c1)C)c2nc3cc(ccc3o2)NC(=S)NC(=O)c4ccc(c(c4)OC)OC
COc1cccc(c1)c2nc3cc(ccc3o2)NC(=S)NC(=O)c4ccc(c(c4)OC)OC
COc1ccc(cc1OC)C(=O)NC(=S)Nc2ccc(c(c2)Cl)N3CCOCC3
COc1ccc(cc1OC)C(=O)NC(=S)Nc2ccc(c(c2)Cl)N3CCCCC3
Cc1ccc(cc1NC(=S)NC(=O)c2ccc(c(c2)OC)OC)c3nc4c(o3)cccn4
Cc1c(cccc1NC(=S)NC(=O)c2ccc(c(c2)OC)OC)c3nc4c(o3)cccn4
COc1ccc(cc1OC)C(=O)NC(=S)Nc2cccc(c2)c3nc4ccccc4s3
Cc1c(cccc1NC(=S)NC(=O)c2ccc(c(c2)OC)OC)c3nc4ccccc4s3
COc1ccc(cc1OC)C(=O)NC(=S)Nc2cccc(c2)c3nc4cc(ccc4o3)Cl
Cc1c(cccc1NC(=S)NC(=O)c2ccc(c(c2)OC)OC)c3nc4cc(ccc4o3)Cl
CCOC(=O)c1c(oc2c1cc(cc2)N(C(=O)C3CCCCC3)S(=O)(=O)c4ccc(cc4)F)C
c1ccc(cc1)c2c(n(c(n2)SCC(=O)NCc3ccco3)c4ccccc4)c5ccccc5
c1ccc(c(c1)/C=N/NC(=O)CSc2[nH]c3ccccc3n2)I
CC1=NC2=C([C@@H](C1C(=O)OC)c3ccc(c(c3)Cl)Cl)C(=O)C[C@@H](C2)c4cccs4
```

CC1=NC2=C([C@H](C1C(=O)OC)c3ccc(c(c3)Cl)Cl)C(=O)C[C@@H](C2)c4cccs4
CC1=NC2=C([C@@H](C1C(=O)OC)c3ccc(c(c3)Cl)Cl)C(=O)C[C@H](C2)c4cccs4
CC1=NC2=C([C@H](C1C(=O)OC)c3ccc(c(c3)Cl)Cl)C(=O)C[C@H](C2)c4cccs4
c1ccc(cc1)c2c3cc(ccc3nc(n2)N4CCN(CC4)C(=O)c5ccccc5)Cl
C[C@H]1CCc2c(c(c(s2)NC(=O)c3ccccc3)C#N)C1
COc1ccc(cc1)NC(=O)c2c3c(sc2NC(=O)c4cccs4)CCCC3
c1cc(sc1)C(=O)Nc2c(c3c(s2)CCC3)C(=O)Nc4ccc(cc4)Br
CC1=CC(N(c2c1cc(cc2)OC(=O)c3ccc(cc3)OC)C(=O)c4ccc(cc4)OC)(C)C
c1cc(cc(c1)[N+](=O)[O-])/C=C/C(=O)Nc2ccc(cc2)c3nc4cc(ccc4o3)Cl
c1ccc2c(c1)cccc2n3nc4ccc(cc4n3)NC(=O)c5ccc6c(c5)OCCO6
Cc1ccc(cc1NC(=O)Cn2cnc3c2c(=O)n(c(=O)n3C)C)[N+](=O)[O-]
CC(C)[C@@H](C(=O)Nc1ccccc1)NC(=O)c2ccccc2NC(=O)c3ccc(cc3)OC
CC(C)[C@H](C(=O)Nc1ccccc1)NC(=O)c2ccccc2NC(=O)c3ccc(cc3)OC
CC(C)[C@@H](C(=O)Nc1ccc(cc1)OC)NC(=O)c2ccccc2NC(=O)c3ccc(cc3)OC
CC(C)[C@H](C(=O)Nc1ccc(cc1)OC)NC(=O)c2ccccc2NC(=O)c3ccc(cc3)OC
Cc1c(c(n(n1)c2c(cc(cc2Cl)Cl)Cl)[O-])/C=C/3\C(=NN(C3=O)c4ccccc4)C
Cc1c(c2ccccc2[nH]1)/C=c\3/c(=O)n4c(=NC(=C([C@H]4c5c6ccccc6ccc5OC)C(=O)C)C)s3
Cc1c(c2ccccc2[nH]1)/C=c\3/c(=O)n4c(=NC(=C([C@@H]4c5c6ccccc6ccc5OC)C(=O)C)C)s3
Cc1cc(ccc1N2C(=O)/C(=C/c3ccc(c(c3)Br)O)/C(=O)NC2=S)Br
Cc1c(c2ccccc2[nH]1)/C=c\3/c(=O)n4c(=NC(=C([C@H]4c5ccc(cc5)C(C)C)C(=O)OC)C)s3
Cc1c(c2ccccc2[nH]1)/C=c\3/c(=O)n4c(=NC(=C([C@@H]4c5ccc(cc5)C(C)C)C(=O)OC)C)s3
Cc1ccc2c(c1)sc3n2cc(n3)c4cc5cc(ccc5oc4=O)Br
c1ccc(cc1)CN(CC(=O)Nc2c(cc(cc2Cl)Cl)Cl)C(=O)c3ccco3
Cn1c2c(c(=O)n(c1=O)C)n(cn2)C[C@@H](COCc3ccc(cc3)F)OCc4ccc(cc4)F
Cn1c2c(c(=O)n(c1=O)C)n(cn2)C[C@H](COCc3ccc(cc3)F)OCc4ccc(cc4)F
Cn1c2c(c(=O)n(c1=O)C)n(cn2)C[C@@H](COCc3ccccc3F)OCc4ccccc4F
Cn1c2c(c(=O)n(c1=O)C)n(cn2)C[C@H](COCc3ccccc3F)OCc4ccccc4F
COc1ccc2c(c1)C=C[C@@]3(O2)c4ccc(c5c4c(ccc5)O3)OC
COc1ccc2c(c1)C=C[C@]3(O2)c4ccc(c5c4c(ccc5)O3)OC
COc1ccc2c3c1cccc3O[C@]24C=Cc5cc(c(cc5O4)OC)O
COc1ccc2c3c1cccc3O[C@@]24C=Cc5cc(c(cc5O4)OC)O

```
C[C@@H](c1ccccc1)NC(=O)c2cc3cc(cc(c3oc2=O)Br)Br
C[C@H](c1ccccc1)NC(=O)c2cc3cc(cc(c3oc2=O)Br)Br
c1cc(cc(c1)Cl)C(=O)NN[C@@H]2CC(=O)N(C2=O)c3ccc(cc3)Br
c1ccc(c(c1)C(=O)NN[C@H]2CC(=O)N(C2=O)c3ccc(cc3)Br)I
c1ccc(c(c1)C(=O)NN[C@@H]2CC(=O)N(C2=O)c3ccc(cc3)Cl)Br
c1ccc(cc1)CC(=O)Nc2ccc(cc2Cl)N3C(=O)[C@@H]4[C@@H](C3=O)C5c6ccccc6C4c7c5cccc7
c1ccc(cc1)CC(=O)Nc2ccc(cc2Cl)N3C(=O)[C@H]4[C@H](C3=O)C5c6ccccc6C4c7c5cccc7
c1ccc(cc1)C[C@@H](C(=O)OCC(=O)c2ccccc2)N3C(=O)c4cccc(c4C3=O)[N+](=O)[O-]
c1ccc(cc1)C[C@H](C(=O)OCC(=O)c2ccccc2)N3C(=O)c4cccc(c4C3=O)[N+](=O)[O-]
Cc1ccc(cc1)C(=O)COC(=O)[C@@H](Cc2ccccc2)N3C(=O)c4cccc(c4C3=O)[N+](=O)[O-]
Cc1ccc(cc1)C(=O)COC(=O)[C@H](Cc2ccccc2)N3C(=O)c4cccc(c4C3=O)[N+](=O)[O-]
COc1ccc(cc1)C(=O)COC(=O)[C@@H](Cc2ccccc2)N3C(=O)c4cccc(c4C3=O)[N+](=O)[O-]
COc1ccc(cc1)C(=O)COC(=O)[C@H](Cc2ccccc2)N3C(=O)c4cccc(c4C3=O)[N+](=O)[O-]
C[C@H](C(=O)c1ccccc1)OC(=O)c2ccc(cc2)N3C(=O)c4cccc(c4C3=O)[N+](=O)[O-]
C[C@@H](C(=O)c1ccccc1)OC(=O)c2ccc3c(c2)C(=O)N(C3=O)c4cccc(c4)[N+](=O)[O-]
C[C@H](C(=O)c1ccccc1)OC(=O)c2ccc3c(c2)C(=O)N(C3=O)c4cccc(c4)[N+](=O)[O-]
C[C@@H](C(=O)c1ccccc1)OC(=O)c2cccc(c2)N3C(=O)c4cccc(c4C3=O)[N+](=O)[O-]
C[C@H](C(=O)c1ccccc1)OC(=O)c2cccc(c2)N3C(=O)c4cccc(c4C3=O)[N+](=O)[O-]
COc1ccc(cc1OC)C(=O)NC(=S)Nc2ccc(c(c2)c3nc4c(o3)cccn4)Cl
CCOc1cc(ccc1OCc2ccc(cc2)F)CNc3ccccc3
Cc1ccc(c(c1)NCc2ccc(c(c2)OC)OCc3ccccc3F)C
COc1ccc(cc1)CNC(=O)c2ccccn2
Cn1c2c(nc1Sc3nc4ccccc4n3Cc5cccc(c5)Br)n(c(=O)n(c2=O)C)C
COc1ccc2ccc(c(c2c1)/C=C\3/C(=O)N(C(=O)S3)c4ccc(cc4)Cl)OC
COc1ccc2ccc(c(c2c1)/C=C\3/C(=O)N(C(=O)S3)c4cccc(c4)Cl)OC
COc1cc(ccc1OCc2ccc(cc2)Cl)/C=C\3/C(=O)NN(C3=O)c4cccc(c4)Cl
COc1cc(ccc1OCc2ccc(cc2)Cl)/C=C\3/C(=O)NN(C3=O)c4ccc(cc4)F
COc1cc(ccc1OCc2ccc(cc2)Cl)/C=C\3/C(=O)NN(C3=O)c4ccc(cc4)C(=O)OC
COc1cc(c(cc1/C=C\2/C(=O)N(C(=O)S2)c3cccc(c3)Cl)Br)OC
COc1ccc(cc1Br)/C=C\2/C(=O)N(C(=O)S2)c3cccc(c3)Cl
COc1cc(cc(c1O)I)/C=C\2/C(=O)N(C(=O)S2)c3cccc(c3)Cl
```

```
c1cc(cc(c1)Cl)N2C(=O)/C(=C/c3ccc(c(c3)I)O)/SC2=O
COc1cc(cc(c1OC)Br)/C=C\2/C(=O)N(C(=O)S2)c3cccc(c3)Cl
CCOC(=O)COc1ccc(cc1OC)/C=C\2/C(=O)N(C(=O)S2)c3cccc(c3)Cl
COc1cc(c(cc1OC)Br)/C=C\2/C(=O)N(C(=O)S2)c3cccc(c3)Cl
CCOc1cc(cc(c1O)/C=C\2/C(=O)N(C(=O)S2)c3cccc(c3)Cl)Br
COc1ccc(cc1/C=C\2/C(=O)N(C(=O)S2)c3cccc(c3)Cl)Br
CCOc1cc(ccc1OCC(=O)OC)/C=C\2/C(=O)N(C(=O)S2)c3cccc(c3)Cl
Cc1ccc(cc1)CN(CC(=O)Nc2ccc(cc2)C)S(=O)(=O)c3ccccc3
COc1cc(c(cc1O)Br)/C=C\2/C(=O)N(C(=O)S2)c3ccc(cc3)Cl
COc1cc(c(cc1/C=C\2/C(=O)N(C(=O)S2)c3ccc(cc3)Cl)Br)OC
c1cc(ccc1N2C(=O)/C(=C/c3ccc(c(c3)I)O)/SC2=O)Cl
COc1cc(c(cc1OC)[N+](=O)[O-])/C=C\2/C(=O)N(C(=O)S2)c3ccc(cc3)Cl
CCOC(=O)COc1ccc(cc1OC)/C=C\2/C(=O)N(C(=O)S2)c3ccc(cc3)Cl
CCOc1cc(cc(c1O)/C=C\2/C(=O)N(C(=O)S2)c3ccc(cc3)Cl)Br
CCOC(=O)COc1ccc(cc1Cl)/C=C\2/C(=O)N(C(=O)S2)c3ccc(cc3)Cl
COc1ccc(cc1/C=C\2/C(=O)N(C(=O)S2)c3ccc(cc3)Cl)Br
COc1cc(ccc1OCC(=O)OC)/C=C\2/C(=O)N(C(=O)S2)c3ccc(cc3)Cl
COc1cc(c(cc1O)Br)/C=C\2/C(=O)N(C(=O)S2)c3cccc(c3)Cl
CCN\1C(=O)/C(=C/c2ccc(c(c2)Br)OC)/S/C1=N/c3ccc(cc3)OCC
CCN\1C(=O)/C(=C/c2ccc(o2)Br)/S/C1=N/c3ccc(cc3)OCC
CCN\1C(=O)/C(=C/c2cc(c(c(c2)OC)OC)OC)/S/C1=N/c3ccc(cc3)OCC
CCN\1C(=O)/C(=C/c2cc(c(cc2Cl)OC)OC)/S/C1=N/c3ccc(cc3)OCC
CCN\1C(=O)/C(=C/c2cc(c(c(c2)Cl)O)OCC)/S/C1=N/c3ccc(cc3)OCC
CCN\1C(=O)/C(=C/c2cc(ccc2O)Br)/S/C1=N/c3ccc(cc3)OCC
CCN\1C(=O)/C(=C/c2cccn2c3cccc(c3)[N+](=O)[O-])/S/C1=N/c4ccc(cc4)OCC
CCN\1C(=O)/C(=C/c2cc(ccc2OC)Br)/S/C1=N/c3ccc(cc3)OCC
CN\1C(=O)/C(=C/c2ccc(o2)Sc3ccccc3)/S/C1=N/c4ccc(cc4)C(=O)OC
CN\1C(=O)/C(=C/c2cc(ccc2O)Br)/S/C1=N/c3ccc(cc3)C(=O)OC
CN\1C(=O)/C(=C/c2cccn2c3cccc(c3)[N+](=O)[O-])/S/C1=N/c4ccc(cc4)C(=O)OC
CN\1C(=O)/C(=C/c2ccccc2OCc3ccccc3C#N)/S/C1=N/c4ccc(cc4)C(=O)OC
CCOC(=O)c1ccc(cc1)/N=C/2\N(C(=O)/C(=C/c3ccc(o3)Sc4ccccc4)/S2)C
```

CCOC(=O)c1ccc(cc1)/N=C/2\N(C(=O)/C(=C/c3cc(c(c(c3)Cl)O)OC)/S2)C
CCOC(=O)c1ccc(cc1)/N=C/2\N(C(=O)/C(=C/c3cc(ccc3O)Br)/S2)C
CCOC(=O)COc1ccc(cc1OC)/C=C/2\C=C(OC2=O)c3ccc(cc3)Br
Cc1ccc(cc1)/N=C/2\N(C(=O)/C(=C/c3cc(c(c(c3)Br)O)OC)/S2)C
CCCN1C(=O)/C(=C/c2cc(c(c(c2)I)O)OC)/SC1=O
CCOc1ccc(cc1)/C=C/2\C(=O)N(C(=O)S2)Cc3cc4c(cc3Cl)OCO4
COc1ccccc1NC(=O)COc2ccc(cc2)/C=C/3\C(=O)N(C(=O)S3)Cc4cc5c(cc4Cl)OCO5
CCOc1cc(cc(c1O)I)/C=C/2\C(=O)N(C(=O)S2)CC(=O)Nc3ccccc3C
Cc1ccccc1NC(=O)CN2C(=O)/C(=C/c3cc(c(c(c3)OC)OC)CC=C)/SC2=O
Cc1ccccc1NC(=O)CN2C(=O)/C(=C/c3cc(c(c(c3)Cl)O)Cl)/SC2=O
Cc1ccc(cc1c2ccc(o2)/C=C\3/C(=O)N(C(=O)S3)CC(=O)Nc4ccccc4)C(=O)[O-]
CC(=O)c1ccc(cc1)c2ccc(o2)/C=C/3\C(=O)N(C(=O)S3)CC(=O)N4CCOCC4
Cc1ccc(cc1c2ccc(o2)/C=C\3/C(=O)N(C(=O)S3)Cc4ccc(cc4)F)C(=O)[O-]
Cn1c2ccc(cc2n(c1=O)C)/C=C/3\C(=O)N(C(=O)S3)Cc4ccc(cc4Cl)Cl
CCOc1cc(ccc1OC(C)C)/C=C/2\C(=O)N(C(=O)S2)CC(=O)Nc3ccc(c(c3)C)C
CC[C@H](C)Oc1ccc(cc1OC)/C=C\2/C(=O)N(C(=O)S2)CC(=O)Nc3ccc(c(c3)C)C
c1ccc(cc1)c2ccc(o2)/C=C\3/C(=O)N(C(=O)S3)CC(=O)Nc4ccc(cc4)F
CCOc1cc(ccc1OC(C)C)/C=C/2\C(=O)N(C(=O)S2)CC(=O)Nc3ccc(cc3)F
CC(C)n1cc(c2c1cccc2)/C=C/3\C(=O)N(C(=O)S3)CC(=O)Nc4ccc(cc4)F
Cc1cccc(c1)NC(=O)CN2C(=O)/C(=C/c3cc(c(c(c3)OC)OC)CC=C)/SC2=O
Cc1cccc(c1)NC(=O)CN2C(=O)/C(=C/c3cc(c(c(c3)Cl)O)Cl)/SC2=O
Cc1ccc(cc1)CN2C(=O)/C(=C/c3ccc(cc3)OCC(=O)Nc4ccccc4C)/SC2=O
COc1cc(cc(c1)OC)/C=C/2\C(=O)N(C(=O)S2)Cc3cc4c(cc3Cl)OCO4
c1cc2c(cc1Br)c(c[nH]2)/C=C\3/C(=O)N(C(=O)S3)Cc4cc5c(cc4Cl)OCO5
CCc1cccc2c1[nH]cc2/C=C\3/C(=O)N(C(=O)S3)Cc4cc5c(cc4Cl)OCO5
CCOc1cc(cc(c1O)Cl)/C=C\2/C(=O)N(C(=O)S2)Cc3cc4c(cc3Cl)OCO4
CCOc1cc(ccc1OC(C)C)/C=C/2\C(=O)N(C(=O)S2)Cc3cc4c(cc3Cl)OCO4
CCOc1cc(ccc1OCC(=O)Nc2ccccc2C)/C=C\3/C(=O)N(C(=O)S3)Cc4cc5c(cc4Cl)OCO5
c1cc(ccc1C(=O)CN2C(=O)/C(=C/c3cc4c(cc3Cl)OCO4)/SC2=O)Cl
c1cc(ccc1C(=O)CN2C(=O)/C(=C/c3cc4c(cc3Br)OCO4)/SC2=O)Cl
Cc1ccc(cc1)NC2=C([C@H]([C@H](C(=C2)C)C(=O)OC)c3ccc(cc3)Cl)C(=O)OC

```
Cc1ccc(cc1)NC2=C([C@H]([C@@H](C(=C2)C)C(=O)OC)c3ccc(cc3)Cl)C(=O)OC
Cc1ccc(cc1)NC2=C([C@@H]([C@H](C(=C2)C)C(=O)OC)c3ccc(cc3)Cl)C(=O)OC
Cc1ccc(cc1)NC2=C([C@@H]([C@@H](C(=C2)C)C(=O)OC)c3ccc(cc3)Cl)C(=O)OC
Cc1ccc(cc1)NC2=C([C@H]([C@H](C(=C2)C)C(=O)OC)c3ccc(cc3)Br)C(=O)OC
Cc1ccc(cc1)NC2=C([C@H]([C@@H](C(=C2)C)C(=O)OC)c3ccc(cc3)Br)C(=O)OC
Cc1ccc(cc1)NC2=C([C@@H]([C@H](C(=C2)C)C(=O)OC)c3ccc(cc3)Br)C(=O)OC
Cc1ccc(cc1)NC2=C([C@@H]([C@@H](C(=C2)C)C(=O)OC)c3ccc(cc3)Br)C(=O)OC
CCOc1ccc(cc1)[C@H]2[C@H](C(=CC(=C2C(=O)OC)Nc3ccc(cc3)C)C)C(=O)OC
CCOc1ccc(cc1)[C@@H]2[C@H](C(=CC(=C2C(=O)OC)Nc3ccc(cc3)C)C)C(=O)OC
CCOc1ccc(cc1)[C@H]2[C@@H](C(=CC(=C2C(=O)OC)Nc3ccc(cc3)C)C)C(=O)OC
CCOc1ccc(cc1)[C@@H]2[C@@H](C(=CC(=C2C(=O)OC)Nc3ccc(cc3)C)C)C(=O)OC
c1ccc(c(c1)C(=O)Nc2cccc(c2Cl)Cl)NC(=O)[C@H]3CC=CC[C@H]3C(=O)[O-]
c1ccc(c(c1)C(=O)Nc2cccc(c2Cl)Cl)NC(=O)[C@@H]3CC=CC[C@H]3C(=O)[O-]
c1ccc(c(c1)C(=O)Nc2cccc(c2Cl)Cl)NC(=O)[C@@H]3CC=CC[C@@H]3C(=O)[O-]
c1ccc(cc1)Cn2c3ccc(cc3nc2CNc4ccc(cc4)N5CCOCC5)Cl
CCc1ccc(cc1)S(=O)(=O)N(c2c(nc3n2cccc3)c4ccccc4)S(=O)(=O)c5ccc(cc5)CC
C[C@@H]1CN(C[C@H](O1)C)C2=NC(=O)/C(=C/c3cn(nc3c4cc5ccccc5o4)c6ccccc6)/S2
C[C@@H]1Cc2cc(ccc2O1)C(=O)C3=C(C(=O)N([C@@H]3c4ccc(c(c4)OC)O)C[C@@H]5CCCO5)[O-]
C[C@@H]1Cc2cc(ccc2O1)C(=O)C3=C(C(=O)N([C@H]3c4cccc(c4)OC)C[C@@H]5CCCO5)[O-]
C[C@H]1Cc2cc(ccc2O1)C(=O)C3=C(C(=O)N([C@@H]3c4cccs4)C[C@@H]5CCCO5)[O-]
CCc1cccc(c1NC(=O)COc2ccc3c(c2)OC(C=C3)(C)C)C
CCc1cccc(c1NC(=O)COc2cc(c3c(c2)OC(C=C3)(C)C)C)CC
CCc1cccc(c1NC(=O)COc2ccc3c(c2C)OC(C=C3)(C)C)CC
Cc1ccc(cc1)NC(=O)COc2cc3c(cc2OC)OC(C=C3)(C)C
CCc1cccc(c1NC(=O)COc2cc3c(cc2OC)OC(C=C3)(C)C)C
CCc1cccc(c1NC(=O)COc2cc3c(cc2OC)C=CC(O3)(C)C)C
CCc1cccc(c1NC(=O)COc2cc3c(cc2OC)C=CC(O3)(C)C)CC
CN(C)c1ccc(cc1)/C=C\2/[C@H](Oc3ccccc3C2=O)c4ccccc4
CN(C)c1ccc(cc1)/C=C\2/[C@@H](Oc3ccccc3C2=O)c4ccccc4
COc1ccc(cc1Cl)NCc2ccc(cc2)OCc3ccccc3F
COc1ccc(cc1OCc2ccccc2)CNc3ccc(c(c3)F)F
```

```
COc1cc(ccc1OCc2ccccc2)CNc3ccc(c(c3)F)F
CCCc1nnc(s1)NC(=O)c2cn(c3cc(c(cc3c2=O)F)N4CCN(CC4)C(=O)c5cccc(c5)Cl)C6CC6
Cc1ccc(cc1)n2c(cc(c2C)/C=C/3\C(=NC(=S)N(C3=O)c4ccc(cc4)F)[O-])C
CCOC1=C([C@H](Oc2c1cccc2)c3ccc(cc3)C)C[NH+](C)C
CCOC1=C([C@@H](Oc2c1cccc2)c3ccc(cc3)C)C[NH+](C)C
CCOC1=C([C@H](Oc2c1cccc2)c3ccc(cc3)F)C[NH+](C)C
CCOC1=C([C@@H](Oc2c1cccc2)c3ccc(cc3)F)C[NH+](C)C
CCOC1=C([C@H](Oc2c1cccc2)c3ccc(cc3)F)C[NH+]4CCCCC4
CCOC1=C([C@@H](Oc2c1cccc2)c3ccc(cc3)F)C[NH+]4CCCCC4
c1ccc2c(c1)c(c[nH]2)CCN3[C@H](C(=C(C3=O)[O-])C(=O)c4ccc(cc4)F)c5ccc(cc5)Cl
c1ccc2c(c1)c(c[nH]2)CCN3[C@@H](C(=C(C3=O)[O-])C(=O)c4ccc(cc4)F)c5ccc(cc5)Cl
c1ccc2c(c1)c(c[nH]2)CCN3[C@H](C(=C(C3=O)[O-])C(=O)c4ccc(cc4)F)c5ccccc5Cl
c1ccc2c(c1)c(c[nH]2)CCN3[C@@H](C(=C(C3=O)[O-])C(=O)c4ccc(cc4)F)c5ccccc5Cl
COC(=O)c1ccc(cc1)[C@H]2C(=C(C(=O)N2CCc3c[nH]c4c3cccc4)[O-])C(=O)c5ccc(cc5)F
COC(=O)c1ccc(cc1)[C@@H]2C(=C(C(=O)N2CCc3c[nH]c4c3cccc4)[O-])C(=O)c5ccc(cc5)F
COc1ccccc1[C@H]2C(=C(C(=O)N2CCc3c[nH]c4c3cccc4)[O-])C(=O)c5ccc(cc5)Cl
COc1ccccc1[C@@H]2C(=C(C(=O)N2CCc3c[nH]c4c3cccc4)[O-])C(=O)c5ccc(cc5)Cl
c1ccc2c(c1)c(c[nH]2)CCN3[C@@H](C(=C(C3=O)[O-])C(=O)c4ccc(cc4)Cl)c5cccc(c5)O
COc1ccc(cc1)C(=O)C2=C(C(=O)N([C@H]2c3ccccc3)CCc4c[nH]c5c4cccc5)[O-]
COc1ccc(cc1)C(=O)C2=C(C(=O)N([C@@H]2c3ccccc3)CCc4c[nH]c5c4cccc5)[O-]
Cc1ccc(cc1)[C@H]2C(=C(C(=O)N2CCc3c[nH]c4c3cccc4)[O-])C(=O)c5ccc(cc5)OC
Cc1ccc(cc1)[C@@H]2C(=C(C(=O)N2CCc3c[nH]c4c3cccc4)[O-])C(=O)c5ccc(cc5)OC
COc1ccc(cc1)[C@H]2C(=C(C(=O)N2CCc3c[nH]c4c3cccc4)[O-])C(=O)c5ccc(cc5)OC
COc1ccc(cc1)[C@@H]2C(=C(C(=O)N2CCc3c[nH]c4c3cccc4)[O-])C(=O)c5ccc(cc5)OC
COc1ccc(cc1)C(=O)C2=C(C(=O)N([C@H]2c3ccc(cc3)Cl)CCc4c[nH]c5c4cccc5)[O-]
COc1ccc(cc1)C(=O)C2=C(C(=O)N([C@@H]2c3ccc(cc3)Cl)CCc4c[nH]c5c4cccc5)[O-]
COc1ccc(cc1)C(=O)C2=C(C(=O)N([C@H]2c3ccc(cc3)F)CCc4c[nH]c5c4cccc5)[O-]
COc1ccc(cc1)C(=O)C2=C(C(=O)N([C@@H]2c3ccc(cc3)F)CCc4c[nH]c5c4cccc5)[O-]
COc1ccc(cc1)C(=O)C2=C(C(=O)N([C@H]2c3cccc(c3)F)CCc4c[nH]c5c4cccc5)[O-]
COc1ccc(cc1)C(=O)C2=C(C(=O)N([C@@H]2c3cccc(c3)F)CCc4c[nH]c5c4cccc5)[O-]
COc1ccc(cc1)C(=O)C2=C(C(=O)N([C@H]2c3ccccc3F)CCc4c[nH]c5c4cccc5)[O-]
```

```
COc1ccc(cc1)C(=O)C2=C(C(=O)N([C@@H]2c3ccccc3F)CCc4c[nH]c5c4cccc5)[O-]
CCc1ccc(cc1)[C@H]2C(=C(C(=O)N2CCc3c[nH]c4c3cccc4)[O-])C(=O)c5ccc(cc5)OC
CCc1ccc(cc1)[C@@H]2C(=C(C(=O)N2CCc3c[nH]c4c3cccc4)[O-])C(=O)c5ccc(cc5)OC
CC(C)c1ccc(cc1)[C@H]2C(=C(C(=O)N2CCc3c[nH]c4c3cccc4)[O-])C(=O)c5ccc(cc5)OC
CC(C)c1ccc(cc1)[C@@H]2C(=C(C(=O)N2CCc3c[nH]c4c3cccc4)[O-])C(=O)c5ccc(cc5)OC
COc1ccc(cc1)C(=O)C2=C(C(=O)N([C@H]2c3ccc(c(c3)OC)O)CCc4c[nH]c5c4cccc5)[O-]
COc1ccc(cc1)C(=O)C2=C(C(=O)N([C@@H]2c3ccc(c(c3)OC)O)CCc4c[nH]c5c4cccc5)[O-]
CCOc1ccc(cc1)[C@H]2C(=C(C(=O)N2CCc3c[nH]c4c3cccc4)[O-])C(=O)c5ccc(cc5)OC
CCOc1ccc(cc1)[C@@H]2C(=C(C(=O)N2CCc3c[nH]c4c3cccc4)[O-])C(=O)c5ccc(cc5)OC
c1ccc(cc1)[C@H]2C(=C(C(=O)N2CCc3c[nH]c4c3cccc4)[O-])C(=O)c5ccc(cc5)F
c1ccc(cc1)[C@@H]2C(=C(C(=O)N2CCc3c[nH]c4c3cccc4)[O-])C(=O)c5ccc(cc5)F
COc1cc(ccc1O)[C@H]2C(=C(C(=O)N2CCc3c[nH]c4c3cccc4)[O-])C(=O)c5ccc(cc5)F
COc1cc(ccc1O)[C@@H]2C(=C(C(=O)N2CCc3c[nH]c4c3cccc4)[O-])C(=O)c5ccc(cc5)F
c1ccc2c(c1)c(c[nH]2)CCN3[C@H](C(=C(C3=O)[O-])C(=O)c4ccc(cc4)F)c5ccc(cc5)O
c1ccc2c(c1)c(c[nH]2)CCN3[C@@H](C(=C(C3=O)[O-])C(=O)c4ccc(cc4)F)c5ccc(cc5)O
c1ccc(cc1)[C@H]2C(=C(C(=O)N2CCc3c[nH]c4c3cccc4)[O-])C(=O)c5ccc(cc5)Cl
c1ccc(cc1)[C@@H]2C(=C(C(=O)N2CCc3c[nH]c4c3cccc4)[O-])C(=O)c5ccc(cc5)Cl
c1ccc2c(c1)c(c[nH]2)CCN3[C@H](C(=C(C3=O)[O-])C(=O)c4ccc(cc4)Cl)c5ccc(cc5)F
c1ccc2c(c1)c(c[nH]2)CCN3[C@@H](C(=C(C3=O)[O-])C(=O)c4ccc(cc4)Cl)c5ccc(cc5)F
c1ccc2c(c1)c(c[nH]2)CCN3[C@H](C(=C(C3=O)[O-])C(=O)c4ccc(cc4)Cl)c5cccc(c5)F
c1ccc2c(c1)c(c[nH]2)CCN3[C@@H](C(=C(C3=O)[O-])C(=O)c4ccc(cc4)Cl)c5cccc(c5)F
c1ccc2c(c1)c(c[nH]2)CCN3[C@H](C(=C(C3=O)[O-])C(=O)c4ccc(cc4)Cl)c5ccccc5F
c1ccc2c(c1)c(c[nH]2)CCN3[C@@H](C(=C(C3=O)[O-])C(=O)c4ccc(cc4)Cl)c5ccccc5F
c1ccc2c(c1)c(c[nH]2)CCN3[C@H](C(=C(C3=O)[O-])C(=O)c4ccc(cc4)Cl)c5cccnc5
c1ccc2c(c1)c(c[nH]2)CCN3[C@@H](C(=C(C3=O)[O-])C(=O)c4ccc(cc4)Cl)c5cccnc5
c1ccc2c(c1)c(c[nH]2)CCN3[C@H](C(=C(C3=O)[O-])C(=O)c4ccc(cc4)Cl)c5ccccn5
c1ccc2c(c1)c(c[nH]2)CCN3[C@@H](C(=C(C3=O)[O-])C(=O)c4ccc(cc4)Cl)c5ccccn5
COc1ccc(cc1)C2=Cc3ccccc3O[C@H]2c4ccc(cc4)OCC[NH+]5CCCCC5
CC(C)(C)c1ccc2cc(ccc2c1)C(C)(C)C
Cc1ccc(cc1)N[C@@H]2C=C(Oc3c2cccc3)c4ccccc4
Cc1ccc(c(c1)C)N[C@@H]2C=C(Oc3c2cccc3)c4ccccc4
```

```
CC1(C(=C(c2ccc(cc2O1)OC)c3ccc4c(c3)NCCO4)c5ccccc5)C
c1ccc2c(c1)C=C([C@H](O2)c3ccc(cc3)OCC[NH+]4CCCCC4)c5ccc(cc5)O
CCO[C@]1(C(=Cc2ccccc2O1)OC)c3ccccc3
CN(C)c1ccc(cc1)[C@@H]2C=C(Oc3c2cccc3)c4ccccc4
C[C@@H](C[NH+](C)C)C(C#N)(c1ccccc1)c2ccccc2
c1ccc(cc1)C2(C=Cc3ccccc3O2)c4ccccc4
Cc1c2c(cc(c1OC)NC(=O)C)C=CC(O2)(C)C
Cc1c2c(cc(c1OC)N)C=CC(O2)(C)C
Cc1c2c(cc(c1OC)N(C)C)C=CC(O2)(C)C
CCOc1ccc2c(c1)/C(=C/3\SC(=C(S3)C(=O)OC)c4ccccc4)/C(=S)C(N2C(=O)c5ccccc5)(C)C
CC1(C(=S)/C(=C\2/SC(=C(S2)C(=O)OC)c3ccccc3)/c4cc(ccc4N1C(=O)c5ccccc5)OC)C
Cc1cccc(c1OCc2nc3c4c(c(oc4ncn3n2)c5ccccc5)c6ccccc6)C
COc1cc(c(c(c1)[N+](=O)[O-])[O-])[C@H]2Nc3ccc(cc3[C@@H]([NH+]2CC(=O)OC)c4ccccc4)Br
CCCCCCC[n+]1c2ccccc2n(c1N)CC(=O)c3ccco3
Cn1c(nnc1SCC(=O)c2ccc(cc2Cl)Cl)c3c(c4ccccc4s3)Cl
CC1(c2c(c(=S)ss2)-c3cc(ccc3N1C(=O)COc4ccc(cc4)Cl)OC)C
CCCC(=O)N1c2cc(ccc2/C(=C/3\SC(=C(S3)C(=O)OCC)c4ccccc4)/C(=S)C1(C)C)C
Cc1cc-2c(cc1C)N(C(c3c2c(=S)ss3)(C)C)C(=O)COc4ccc(cc4)Cl
CC(=O)N1c2ccc(cc2/C(=C/3\SC(=C(S3)C(=O)OC)c4ccccc4)/C(=S)C1(C)C)OC
CCCCc1ccc(cc1)C(=O)N2c3ccccc3[C@H](C[C@H]2C)N(c4ccccc4)C(=O)c5ccccc5
CCCCc1ccc(cc1)C(=O)N2c3ccccc3[C@H](C[C@@H]2C)N(c4ccccc4)C(=O)c5ccccc5
CCCCc1ccc(cc1)C(=O)N2c3ccccc3[C@@H](C[C@H]2C)N(c4ccccc4)C(=O)c5ccccc5
CCCCc1ccc(cc1)C(=O)N2c3ccccc3[C@@H](C[C@@H]2C)N(c4ccccc4)C(=O)c5ccccc5
C[C@H]1C[C@H](c2ccccc2N1C(=O)c3ccc(cc3)OC)N(c4ccccc4)C(=O)c5ccc(cc5)OC
C[C@@H]1C[C@H](c2ccccc2N1C(=O)c3ccc(cc3)OC)N(c4ccccc4)C(=O)c5ccc(cc5)OC
C[C@H]1C[C@@H](c2ccccc2N1C(=O)c3ccc(cc3)OC)N(c4ccccc4)C(=O)c5ccc(cc5)OC
C[C@@H]1C[C@@H](c2ccccc2N1C(=O)c3ccc(cc3)OC)N(c4ccccc4)C(=O)c5ccc(cc5)OC
CCOC(=O)C1=C(N=c2n(c(=O)/c(=C/c3c[nH]c4c3cccc4)/s2)[C@H]1c5cccc(c5)OC)c6ccccc6
CCOC(=O)C1=C(N=c2n(c(=O)/c(=C/c3c[nH]c4c3cccc4)/s2)[C@@H]1c5cccc(c5)OC)c6ccccc6
CC1(C=Cc2ccccc2O1)C
c1ccc2c(c1)ccc3c2C=CC4(O3)c5ccccc5Nc6c4cccc6
```

```
c1cc(ccc1C2=Nc3nc(nn3[C@@H](C2)c4ccc(cc4Cl)Cl)CCCO)Br
c1cc(ccc1C2=Nc3nc(nn3[C@H](C2)c4ccc(cc4Cl)Cl)CCCO)Br
c1ccc(cc1)c2c3c4nc(nn4cnc3oc2c5ccccc5)c6ccccc6Br
c1ccc(cc1)C2=Cc3ccccc3O[C@H]2c4ccc(cc4)OCC[NH+]5CCCCC5
CCCOc1ccccc1/C=C/C(=O)Nc2ccc(cc2)OCc3ccccc3
Cc1c(c2c(s1)nc(n(c2=O)c3ccccc3)SCC(=O)Nc4cccc(c4)C(F)(F)F)c5ccccc5
Cc1c(c2c(s1)nc(n(c2=O)c3ccccc3)SCC(=O)Nc4cc(ccc4Cl)C(F)(F)F)c5ccccc5
c1cc(c(cc1Cl)Cl)Nc2nnc(s2)c3cc4cc(ccc4oc3=O)Br
CN1c2ccccc2-c3ccccc3[C@]14C=Cc5ccccc5O4
CN1c2ccccc2-c3ccccc3[C@@]14C=Cc5ccccc5O4
COc1ccc(cc1)C2(C=Cc3c4ccc(cc4ccc3O2)OC)c5ccc(cc5)OC
COc1ccc(cc1)[C@]2(C=Cc3c4ccc(cc4ccc3O2)Br)c5ccccc5F
COc1ccc(cc1)[C@@]2(C=Cc3c4ccc(cc4ccc3O2)Br)c5ccccc5F
COc1ccc(cc1)[C@]2(C=Cc3c4ccc(cc4ccc3O2)OC)c5ccccc5F
c1cc(ccc1c2nnc(o2)SCc3ccc(cc3Cl)Cl)Br
CCCCCCCSc1nnc(o1)c2ccc(cc2)Br
Cc1ccc(cc1)S(=O)(=O)N(c2ccc3c(c2)c(c(o3)C)C(=O)C)C(=O)c4ccccc4
c1cc(ccc1[C@H]2C=C(C(=O)N2c3ccc(cc3)Cl)Nc4ccc(cc4)Cl)Br
c1cc(ccc1[C@@H]2C=C(C(=O)N2c3ccc(cc3)Cl)Nc4ccc(cc4)Cl)Br
c1cc(ccc1[C@H]2C=C(C(=O)N2c3ccc(cc3)Cl)Nc4ccc(cc4)Cl)Cl
c1cc(ccc1[C@@H]2C=C(C(=O)N2c3ccc(cc3)Cl)Nc4ccc(cc4)Cl)Cl
c1cc(ccc1[C@H]2C/C(=N/c3ccc(cc3)Cl)/C(=O)N2c4ccc(cc4)Cl)[N+](=O)[O-]
c1cc(ccc1[C@@H]2C/C(=N/c3ccc(cc3)Cl)/C(=O)N2c4ccc(cc4)Cl)[N+](=O)[O-]
c1cc(cc(c1)[N+](=O)[O-])[C@H]2C/C(=N/c3ccc(cc3)Cl)/C(=O)N2c4ccc(cc4)Cl
c1cc(cc(c1)[N+](=O)[O-])[C@@H]2C/C(=N/c3ccc(cc3)Cl)/C(=O)N2c4ccc(cc4)Cl
c1ccc(c(c1)[C@H]2C/C(=N/c3ccc(cc3)Cl)/C(=O)N2c4ccc(cc4)Cl)[N+](=O)[O-]
c1ccc(c(c1)[C@@H]2C/C(=N/c3ccc(cc3)Cl)/C(=O)N2c4ccc(cc4)Cl)[N+](=O)[O-]
c1cc(ccc1[C@H]2C=C(C(=O)N2c3ccc(cc3)F)Nc4ccc(cc4)F)Br
c1cc(ccc1[C@@H]2C=C(C(=O)N2c3ccc(cc3)F)Nc4ccc(cc4)F)Br
c1ccc(cc1)c2c3cc(ccc3nc(n2)Nc4ccc(cc4)C(=O)Nc5cccc(c5)Cl)Cl
c1ccc(c(c1)c2c3cc(ccc3nc(n2)N4CCN(CC4)c5ccccc5Cl)Br)Cl
```

```
c1ccc(c(c1)COc2cccc(c2)/C=C\3/C(=O)OC(=N3)c4ccc(cc4)F)Br
CCOc1cc(cc(c1OCC)OCC)C(=O)Nc2ccc3c(c2)nc(o3)c4ccc(cc4)Br
CCOc1cc(ccc1OCC(=C)C)/C=C\2/C(=O)OC(=N2)c3ccc(cc3)I
CCOc1cc(ccc1OCc2ccccc2Cl)/C=C\3/C(=O)OC(=N3)c4ccccc4Br
CCOc1cc(ccc1OCc2ccccc2Cl)/C=C\3/C(=O)OC(=N3)c4ccc(c(c4)[N+](=O)[O-])C
c1ccc(c(c1)COc2cccc(c2)/C=C\3/C(=O)OC(=N3)c4cc(ccc4Cl)[N+](=O)[O-])Cl
c1ccc(c(c1)COc2ccccc2/C=C\3/C(=O)OC(=N3)c4ccc(cc4)Br)Cl
c1cc(ccc1c2ccc(o2)C(=O)Nc3ccc4c(c3)nc(o4)c5ccc(cc5)Br)Cl
c1ccc2c(c1)nc(s2)c3ccc(cc3)NC(=O)c4ccc(o4)c5ccc(cc5)Cl
Cc1cc(c2c(c1)nc(o2)c3ccc(cc3)NC(=O)c4ccc(o4)c5ccc(cc5)Cl)C
CC(=C)COc1cccc(c1)/C=C\2/C(=O)OC(=N2)c3cc(ccc3Cl)I
CC(=C)COc1c(cc(cc1Br)/C=C\2/C(=O)OC(=N2)c3ccc4ccccc4c3)OC
COc1cc(cc(c1OCc2cccc(c2)Br)Br)/C=C\3/C(=O)OC(=N3)c4ccc(cc4)F
CCOc1cc(cc(c1OCc2ccccc2Br)Cl)/C=C\3/C(=O)OC(=N3)c4cccc(c4)F
CC(=C)COc1c(cc(cc1Br)/C=C\2/C(=O)OC(=N2)c3cccc(c3)I)OC
c1ccc(c(c1)COc2ccccc2/C=C\3/C(=O)OC(=N3)c4cccc(c4)I)Cl
c1cc(cc(c1)NC(=O)c2ccc(o2)c3ccc(cc3)Cl)c4nc5cc(ccc5o4)Cl
COc1cc(cc(c1OCc2ccccc2Cl)Br)/C=C\3/C(=O)OC(=N3)c4cccs4
COc1cc(cc(c1OCc2ccccc2Cl)Br)/C=C\3/C(=O)OC(=N3)c4ccccc4
CCOc1cc(cc(c1OCc2ccccc2Cl)Br)/C=C\3/C(=O)OC(=N3)c4cccc5c4cccc5
COc1cc(cc(c1OCc2ccccc2Cl)OC)/C=C\3/C(=O)OC(=N3)c4cccc(c4)F
COc1cc(cc(c1OCc2ccccc2Cl)Cl)/C=C\3/C(=O)OC(=N3)c4ccc(cc4)F
COc1cc(cc(c1OCc2ccccc2Cl)Cl)/C=C\3/C(=O)OC(=N3)c4cc(c(cc4Cl)F)F
COc1cc(cc(c1OCc2ccccc2Cl)OC)/C=C\3/C(=O)OC(=N3)c4ccc(cc4)I
CCN(CC)c1ccc2cc(c(=O)oc2c1)c3nnc(s3)Nc4ccc(cc4Cl)Cl
Cc1ccc2c(c1)sc(n2)c3ccc(cc3)OC(=O)c4ccccc4I
Cc1ccc2c(c1)sc(n2)c3ccc(cc3)OC(=O)c4ccc(cc4Cl)Cl
CCOc1cc(cc(c1OCC)OCC)C(=O)Oc2ccc(cc2)c3nc4ccc(cc4s3)C
CCOc1ccc(cc1Br)C(=O)Nc2ccc3c(c2)nc(o3)c4ccc(c(c4)Br)OC
COc1ccc(cc1Br)c2nc3cc(ccc3o2)NC(=O)c4cc(ccc4Cl)Br
COc1ccc(cc1Br)c2nc3cc(ccc3o2)NC(=O)COc4ccc(cc4Cl)Cl
```

```
COc1ccc(cc1Br)c2nc3cc(ccc3o2)NC(=O)c4ccc(o4)c5cc(ccc5Cl)Cl
CC(C)Oc1cccc(c1)C(=O)Nc2ccc3c(c2)nc(o3)c4ccc(c(c4)Br)OC
Cc1ccc2c(c1)sc(n2)c3ccc(cc3)OC(=O)COc4ccc(cc4)Br
COc1ccc(cc1Br)c2nc3cc(ccc3o2)NC(=O)c4cc(ccc4Cl)I
Cc1ccc2c(c1)sc(n2)c3ccc(cc3)OC(=O)c4cccc(c4)I
CCOc1ccc(cc1Br)C(=O)Oc2ccc(cc2)c3nc4ccc(cc4s3)C
CCCCCCOc1ccc(cc1)C(=O)Nc2cccc(c2)c3nc4ccccc4o3
c1ccc(cc1)COc2cccc(c2)C(=O)Nc3cccc(c3)c4nc5ccccc5o4
c1ccc(cc1)COc2cccc(c2)C(=O)Nc3ccc4c(c3)nc(o4)c5ccc(cc5)Cl
c1ccc(cc1)COc2cccc(c2)C(=O)Nc3ccc4c(c3)nn(n4)c5cccc(c5)Cl
Cc1ccc(cc1C)NC(=O)c2cc3nc(cc(n3n2)C(F)(F)F)c4ccc(c(c4)Cl)Cl
c1ccc(cc1)c2ccc(cc2)O[C@@H]3[C@H](OCCO3)Oc4ccc(cc4)c5ccccc5
c1ccc(cc1)c2ccc(cc2)O[C@H]3[C@@H](OCCO3)Oc4ccc(cc4)c5ccccc5
c1ccc(cc1)Oc2cccc(c2)c3c4c(c5c6ccccc6ccc5n3)-c7ccccc7C4=O
COc1ccc(cc1)c2nc3c4c(c(oc4ncn3n2)c5ccccc5)c6ccccc6
COC(=O)c1c2c(sc1NC(=O)COc3cccc(c3)NC(=O)c4ccc(cc4)[N+](=O)[O-])CCCC2
c1ccc(c(c1)C(=O)Nc2c(c3c(s2)CCCC3)C(=O)Nc4ccc(cc4)Cl)Cl
Cc1ccccc1NC(=O)c2c3c(sc2NC(=O)c4ccccc4Cl)CCCCC3
c1ccc(cc1)C(=O)Nc2c(c3c(s2)CCCCC3)C(=O)Nc4ccc(cc4)Cl
c1ccc(c(c1)C(=O)Nc2c(c3c(s2)CCCCC3)C(=O)Nc4ccc(cc4)Cl)Cl
c1ccc(cc1)C(=O)Nc2c(c3c(s2)CCCCC3)C(=O)Nc4ccc(cc4)Br
c1ccc(c(c1)C(=O)Nc2c(c3c(s2)CCCCC3)C(=O)Nc4ccc(cc4)Br)Cl
Cc1ccc(cc1)NC(=O)c2c3c(sc2NC(=O)c4ccccc4Cl)CCCCC3
Cc1cccc(c1)NC(=O)c2c3c(sc2NC(=O)c4ccccc4Cl)CCCCC3
CCCCSc1nc2c(nn1)-c3ccccc3N[C@H](O2)c4ccc(c(c4)OC)OC
CCCCSc1nc2c(nn1)-c3ccccc3N[C@@H](O2)c4ccc(c(c4)OC)OC
CC1(CC2=C([C@H](Nc3c2c4ccccc4cc3)c5ccc(cc5)OCc6ccc(cc6Cl)Cl)C(=O)C1)C
CC1(CC2=C([C@@H](Nc3c2c4ccccc4cc3)c5ccc(cc5)OCc6ccc(cc6Cl)Cl)C(=O)C1)C
CCCCCCCCCC(=O)Nc1c(n(n(c1=O)c2ccccc2)C)C
Cc1ccc2c(c1)oc(n2)c3ccc(cc3)NC(=O)c4cc(ccc4Cl)I
CCOc1ccc(cc1Br)C(=O)Nc2ccc3c(c2)nc(o3)c4ccc(cc4Cl)Cl
```

C[C@@]1(CC(N(c2c1cccc2)C(=O)c3ccc(cc3Cl)Cl)(C)C)c4ccccc4
C[C@]1(CC(N(c2c1cccc2)C(=O)c3ccc(cc3Cl)Cl)(C)C)c4ccccc4
CCOC(=O)C1=C(N=c2n(c(=O)/c(=C/c3ccc(o3)c4ccc(cc4)Br)/s2)[C@H]1c5ccccc5Cl)C
CCOC(=O)C1=C(N=c2n(c(=O)/c(=C/c3ccc(o3)c4ccc(cc4)Br)/s2)[C@@H]1c5ccccc5Cl)C
CCOC(=O)C1=C(N=c2n(c(=O)/c(=C/c3ccc(o3)c4ccc(cc4[N+](=O)[O-])OC)/s2)[C@H]1c5ccccc5Cl)C
CCOC(=O)C1=C(N=c2n(c(=O)/c(=C/c3ccc(o3)c4ccc(cc4[N+](=O)[O-])OC)/s2)[C@@H]1c5ccccc5Cl)C
CCOC(=O)C1=C(N(C(=C(C1c2ccc(cc2)N(C)C)C(=O)OCC)C)c3ccccc3)C
c1ccc2c(c1)cccc2c3nc4cc(ccc4o3)NC(=O)c5cc(ccc5I)Br
CCCCOc1ccc(cc1)C(=O)Nc2ccc3c(c2)nc(o3)c4cccc5c4cccc5
CCOc1ccc(cc1Br)C(=O)Nc2ccc3c(c2)nc(o3)c4ccccc4Cl
c1cc(c(cc1C(=O)Nc2ccc3c(c2)nc(o3)c4cc(ccc4Cl)Br)Cl)Cl
COc1ccc(cc1)C(=O)Nc2ccc3c(c2)nc(o3)c4cc(ccc4Cl)Br
c1cc(cc(c1)Cl)c2ccc(o2)/C=C/3\C(=O)N(C(=S)S3)c4cccc(c4)Cl
CCN(CC)c1ccc(cc1)/C=c\2/c(=O)n3c(=NC(=C([C@@H]3c4ccc(c(c4)OC)OC)C(=O)OCC)C)s2
c1cc2c(cccc2Br)c(c1)c3nc4cc(ccc4o3)NC(=O)c5ccc(cc5)F
Cc1cc-2c(cc1C)N(C(c3c2c(=S)ss3)(C)C)C(=O)c4ccc(cc4)c5ccccc5
CCOc1cccc-2c1N(C(c3c2c(=S)ss3)(C)C)C(=O)Cc4ccc(cc4)F
c1ccc(cc1)CC(=O)Nc2c(c3c(s2)CCC3)C(=O)Nc4ccc(cc4)Br
c1ccc(cc1)CC(=O)Nc2c(c3c(s2)CCCC3)C(=O)Nc4ccccc4Cl
CCOC(=O)c1c(c(c(s1)N/C=C/2\C(=O)NC(=O)N(C2=O)c3ccc(cc3)C)C#N)C
Cc1ccccc1[C@H]2c3ccccc3OC(=C2/C=C/C(=O)c4ccc(c(c4)OC)OC)c5ccc(c(c5)OC)OC
Cc1ccccc1[C@@H]2c3ccccc3OC(=C2/C=C/C(=O)c4ccc(c(c4)OC)OC)c5ccc(c(c5)OC)OC
CCOc1ccc(cc1)C(=O)/C=C/C2=C(Oc3ccccc3[C@H]2c4ccccc4C)c5ccccc5
CCOc1ccc(cc1)C(=O)/C=C/C2=C(Oc3ccccc3[C@@H]2c4ccccc4C)c5ccccc5
Cc1ccc(cc1NC(=O)c2cc(ccc2Cl)Br)c3nc4ccccc4s3
c1cc(ccc1c2nc3cc(ccc3o2)NC(=O)c4cc(ccc4Cl)Br)F
c1cc(ccc1c2nc3cc(ccc3o2)NC(=O)c4cc(ccc4Cl)Br)Br
c1cc(cc(c1)F)C(=O)Nc2ccc3c(c2)nc(o3)c4cccc5c4cccc5Br
c1ccc2c(c1)c3c(cc(cc3OCc4ccc(cc4)Cl)OCc5ccc(cc5)Cl)oc2=O
c1cc(ccc1COc2cc3c(c(c2)OCc4ccc(cc4)Cl)c5c(c(=O)o3)CCC5)Cl
COc1cc(c(cc1Cn2c(c(nc2Cl)Cl)Cl)OC)Cn3c(c(nc3Cl)Cl)Cl

```
CCCCCCCCOC(=O)c1ccccc1N2C(=O)[C@@H]3[C@H](C2=O)C4c5ccccc5C3c6c4cccc6
CCCCCCCCOC(=O)c1ccccc1N2C(=O)[C@@H]3[C@@H](C2=O)C4c5ccccc5C3c6c4cccc6
CCCCCCCCOC(=O)c1ccccc1N2C(=O)[C@H]3[C@H](C2=O)C4c5ccccc5C3c6c4cccc6
CCOC(=O)c1ccc(cc1)n2c(cc(c2C)/C=C/3\C(=O)NC(=S)N(C3=O)c4ccc(cc4)Br)C
CCOC(=O)c1ccc(cc1)n2c(cc(c2C)/C=C/3\C(=O)NC(=S)N(C3=O)c4cccc(c4)Cl)C
Cc1cccc(c1)N2C(=O)/C(=C/c3cc(n(c3c4ccccc4)c5ccc(cc5)Cl)c6ccccc6)/C(=NC2=S)[O-]
Cc1ccc(cc1)COc2ccc(cc2OC)[C@H]3C4=C(CC(CC4=O)(C)C)c5c6ccccc6ccc5N3
Cc1ccc(cc1)COc2ccc(cc2OC)[C@@H]3C4=C(CC(CC4=O)(C)C)c5c6ccccc6ccc5N3
CCOc1cc(ccc1OCc2ccc(cc2)Cl)C3C4=C(CC(CC4=O)(C)C)OC5=C3C(=O)CC(C5)(C)C
Cc1cc2c(cc1C)n3c(=O)/c(=C/c4ccc(o4)c5cccc(c5)C(F)(F)F)/sc3n2
Cc1ccc(cc1)Oc2ccc(cc2)N3C(=O)[C@@H]4[C@H](C3=O)C5(c6ccccc6C4c7c5cccc7)Br
Cc1ccc(cc1)Oc2ccc(cc2)N3C(=O)[C@@H]4[C@@H](C3=O)C5(c6ccccc6C4c7c5cccc7)Br
Cc1ccc(cc1)Oc2ccc(cc2)N3C(=O)[C@H]4[C@H](C3=O)C5(c6ccccc6C4c7c5cccc7)Br
Cc1ccc(cc1)Oc2ccc(cc2)N3C(=O)[C@H]4[C@@H](C3=O)C5(c6ccccc6C4c7c5cccc7)Br
c1ccc(cc1)C(=O)c2ccc3c(c2)nc(c(n3)c4ccc(cc4)F)c5ccc(cc5)F
Cc1cccc-2c1N(C(c3c2c(=S)ss3)(C)C)C(=O)c4ccc(cc4)C(C)(C)C
CC1(c2c(c(=S)ss2)-c3ccccc3N1C(=O)COc4ccc(cc4)Cl)C
Cc1ccc\2c(c1)NC(C(=S)/C2=C\3/SC(=C(S3)C(=O)OC)c4ccccc4)(C)C
Cc1ccc2c(c1)/C(=C/3\SC(=C(S3)C(=O)OC)c4ccccc4)/C(=S)C(N2C(=O)c5ccccc5)(C)C
C[C@H]1C[C@H](c2ccccc2N1C(=O)c3ccc(cc3)Cl)N(c4ccccc4)C(=O)c5ccc(cc5)OC
C[C@@H]1C[C@H](c2ccccc2N1C(=O)c3ccc(cc3)Cl)N(c4ccccc4)C(=O)c5ccc(cc5)OC
C[C@H]1C[C@@H](c2ccccc2N1C(=O)c3ccc(cc3)Cl)N(c4ccccc4)C(=O)c5ccc(cc5)OC
C[C@@H]1C[C@@H](c2ccccc2N1C(=O)c3ccc(cc3)Cl)N(c4ccccc4)C(=O)c5ccc(cc5)OC
c1cc2cccnc2c(c1)OS(=O)(=O)c3ccc(cc3)Cl
CCOC(=O)c1c2c(sc1NC(=O)c3cc4nc(cc(n4n3)C(F)(F)F)c5ccc(c(c5)Cl)Cl)CCCC2
c1ccc(cc1)N2C(=O)C(=Cc3ccc(o3)c4cc(ccc4Cl)Cl)C(=O)N(C2=S)c5ccccc5
c1ccc(cc1)c2ccc(cc2)c3csc(n3)c4cc5cc(ccc5oc4=O)Cl
Cc1ccc(cc1)NC(=O)CSc2nc3c(c4c(s3)CCCC4)c(=O)n2c5cccc6c5cccc6
c1ccc2c(c1)cccc2n3c(=O)c4c5c(sc4nc3SCC(=O)Nc6ccc(cc6)Br)CCCC5
c1ccc(cc1)n2c(=O)c3c4c(sc3nc2SCC(=O)Nc5ccc(cc5)Br)CCC4
c1cc(ccc1c2ccc(o2)C(=O)Nc3ccc4c(c3)nc(o4)c5ccc(cc5)Cl)Cl
```

```
CCOc1cc(cc(c1OCC)OCC)C(=O)Nc2ccc(cc2)c3nc4c5ccccc5ccc4o3
Cc1cc(c2c(c1)-c3c(sn(c3=S)c4ccc(c(c4)Cl)Cl)C(N2)(C)C)C
Cc1ccc2c(c1)-c3c(sn(c3=S)c4ccc(c(c4)Cl)Cl)C(N2C)(C)C
Cc1cc-2c(cc1C)N(C(c3c2c(=S)ss3)(C)C)C(=O)CCC4CCCCC4
CC1(c2c(c(=S)ss2)-c3cc(ccc3N1C(=O)CCC4CCCCC4)OC)C
CCOc1ccc2c(c1)-c3c(ssc3=S)C(N2C(=O)CCC4CCCCC4)(C)C
CC1=CC(N(c2c1cc(cc2)OC(=O)c3ccc(cc3)c4ccccc4)C(=O)c5ccc(cc5)c6ccccc6)(C)C
Cc1cc(c2c(c1)-c3c(ssc3=S)C(N2C(=O)CSc4nc5ccccc5s4)(C)C)C
CC1(c2c(c(=S)ss2)-c3ccccc3N1C(=O)c4ccc(cc4Cl)Cl)C
Cc1cc-2c(cc1C)N(C(c3c2c(=S)ss3)(C)C)C(=O)c4ccc(cc4Cl)Cl
CCOc1cccc-2c1N(C(c3c2c(=S)ss3)(C)C)C(=O)c4ccc(cc4Cl)Cl
CCOc1cccc-2c1N(C(c3c2c(=S)ss3)(C)C)C(=O)c4ccc(cc4)c5ccccc5
Cc1ccc(cc1)/C=C/C(=O)N2c3ccc(cc3-c4c(ssc4=S)C2(C)C)OC
Cc1ccc(cc1)/C=C/C(=O)N2c3cc(ccc3-c4c(ssc4=S)C2(C)C)OC
CCCCCCCOc1ccc(cc1)C(=O)Nc2ccc3c(c2)nc(o3)c4ccc(cc4)Cl
Cc1ccccc1c2c3cc(c(cc3c4c(nn(c4n2)c5ccccc5)c6ccccc6)OC)OC
c1cc2c3c(ccc(c3c1)C(=O)Nc4ccc5c(c4)nc(o5)c6cc(ccc6Cl)Cl)CC2
c1ccc2c(c1)nc(o2)c3cccc(c3)NC(=O)c4ccc(o4)c5cc(ccc5Cl)Cl
Cc1ccc2c(c1)nc(o2)c3cccc(c3)NC(=O)c4ccc(o4)c5cc(ccc5Cl)Cl
CCOC(=O)C1=C(N=c2n(c(=O)/c(=C/c3cc(n(c3C)c4ccc(cc4)C)C)/s2)[C@@H]1c5ccc(cc5)C)C
CCOC(=O)C1=C(N=c2n(c(=O)/c(=C/c3cc(n(c3C)c4ccc(cc4)Br)C)/s2)[C@H]1c5ccc(cc5)C)C
CCOC(=O)C1=C(N=c2n(c(=O)/c(=C/c3cc(n(c3C)c4ccc(cc4)Br)C)/s2)[C@@H]1c5ccc(cc5)C)C
Cc1cc2c(cc1C)oc(n2)c3ccc(cc3O)NC(=O)c4ccc(cc4)I
c1ccc(cc1)OC(C(=O)Nc2ccc(cc2)c3nc4ccccc4s3)Oc5ccccc5
Cc1ccc(cc1NC(=O)c2cc(ccc2OC)Br)c3nc4ccccc4s3
Cc1cc(c2c(c1)nc(o2)c3cccc(c3)NC(=O)C(Oc4ccccc4)Oc5ccccc5)C
Cc1ccc(c(c1)C)n2nc3ccc(cc3n2)NC(=O)/C=C/c4ccc(o4)c5ccc(cc5)Cl
CC1(CC2=C([C@H](Nc3c2c4ccccc4cc3)c5ccc(c(c5)OC)OCC=C)C(=O)C1)C
CC1(CC2=C([C@@H](Nc3c2c4ccccc4cc3)c5ccc(c(c5)OC)OCC=C)C(=O)C1)C
c1cc(cc(c1)C(F)(F)F)/C=c/2\c(=O)n3c(=C([C@H](C(=C3N)C#N)c4cccc(c4)C(F)(F)F)C#N)s2
c1cc(cc(c1)C(F)(F)F)/C=c/2\c(=O)n3c(=C([C@@H](C(=C3N)C#N)c4cccc(c4)C(F)(F)F)C#N)s2
```

```
Cc1cc(c2c(c1)nc(o2)c3cccc(c3)NC(=O)c4cc(ccc4OC)Br)C
Cc1cc2c(cc1C)oc(n2)c3cccc(c3)NC(=O)c4cc(ccc4OC)Br
COc1ccc(cc1C(=O)Nc2cc(ccc2Cl)c3nc4ccccc4o3)Br
CCN(c1ccccc1C)C(=O)c2cc(cc(c2)[N+](=O)[O-])C(=O)N(CC)c3ccccc3C
c1cc2c(c(c1)I)N[C@@H]([C@H]3[C@@H]2C=CC3)c4ccc(cc4Cl)Cl
c1ccc(cc1)[C@]2(C=Cc3c4ccccc4ccc3O2)c5ccc(cc5)O
c1ccc(cc1)[C@@]2(C=Cc3c4ccccc4ccc3O2)c5ccc(cc5)O
COc1ccc(cc1)[C@]2(C=Cc3c4ccccc4ccc3O2)c5ccccc5
COc1ccc(cc1)[C@@]2(C=Cc3c4ccccc4ccc3O2)c5ccccc5
Cc1ccc(cc1)Sc2ccc(cc2)[C@]3(C=Cc4c5ccccc5ccc4O3)c6ccccc6
Cc1ccc(cc1)Sc2ccc(cc2)[C@@]3(C=Cc4c5ccccc5ccc4O3)c6ccccc6
Cc1cccc(c1)N2C3=C([C@H](C4=C2c5ccccc5C4=O)c6ccccc6)C(=O)CC(C3)(C)C
Cc1cccc(c1)N2C3=C([C@@H](C4=C2c5ccccc5C4=O)c6ccccc6)C(=O)CC(C3)(C)C
Cc1ccc2c(c1)c(nc(n2)Nc3ccc(cc3C(=O)c4ccccc4)C)c5ccccc5
c1ccc(cc1)c2c3cc(ccc3nc(n2)Nc4ccccc4C(=O)c5ccccc5)Br
c1ccc(cc1)c2c3cc(ccc3nc(n2)Nc4ccc(cc4C(=O)c5ccccc5)Cl)Br
CCOC(=O)C[NH+]1[C@H](c2cc(ccc2N[C@@H]1c3ccccc3OC)Br)c4ccccc4
CCOc1ccc2c(c1)sc(n2)c3ccc(cc3)NC(=O)c4cc(cc(c4)C(C)(C)C)C(C)(C)C
CC(=O)Oc1ccc2ccccc2c1[C@@H](c3ccc(cc3)Cl)NC(=O)Cc4ccccc4
CC(=O)Oc1ccc2ccccc2c1[C@H](c3ccc(cc3)Cl)NC(=O)Cc4ccccc4
c1cc(c(cc1C(F)(F)F)NC(=O)CSCC(=O)Nc2cc(ccc2Cl)C(F)(F)F)Cl
c1ccc2c(c1)ccc3c2cc(c(=O)o3)c4nnc(s4)Nc5cccc(c5)C(F)(F)F
c1ccc2c(c1)cccc2Nc3nnc(s3)c4cc5cc(ccc5oc4=O)Br
c1ccc2c(c1)ccc3c2cc(c(=O)o3)c4nnc(s4)Nc5ccc(cc5)Br
CCOc1cccc2c1oc(=O)c(c2)c3nnc(s3)Nc4ccc(cc4Cl)Cl
CCN(CC)c1ccc2cc(c(=O)oc2c1)c3nnc(s3)Nc4cccc5c4cccc5
c1ccc(cc1)Nc2nnc(s2)c3cc4cc(cc(c4oc3=O)Br)B
CCOC(=O)c1ccc(cc1)Nc2nc(cs2)c3cc4c5ccccc5ccc4oc3=O
CCCCCCc1cc2cc(c(=O)oc2cc1O)c3csc(n3)Nc4ccc(cc4)C(=O)OCC
c1cc(cc(c1)Nc2nc(cs2)c3cc4cc(ccc4oc3=O)Br)C(F)(F)F
Cc1nc2c(c3c(s2)CCCC3)c(=O)n1/N=C/c4ccc(c(c4)OC)OCC(=O)Nc5cccc(c5)[N+](=O)[O-]
```

```
CC(C)(C)c1ccc(cc1)C[n+]2c3ccccc3n(c2N)CC(=O)c4ccc(cc4)c5ccccc5
COc1ccc(cc1)[C@@]2(C=Cc3c(ccc4c3c5ccccc5o4)O2)c6ccccc6F
c1ccc2c(c1)-c3c4c(c(cc(c4no3)N5CCCCC5)Nc6ccc(cc6)Cl)C2=O
CCCCN1c2ccccc2/C(=c/3\c(=O)n4c(=NC(=C([C@H]4c5ccccc5)C(=O)OC)C)s3)/C1=O
CCCCN1c2ccccc2/C(=c/3\c(=O)n4c(=NC(=C([C@@H]4c5ccccc5)C(=O)OC)C)s3)/C1=O
CC(C)NC(=O)/C(=C/c1ccc(o1)c2ccc(cc2Cl)Cl)/NC(=O)c3ccccc3
c1ccc(cc1)CCNC(=O)/C(=C/c2ccc(o2)c3ccc(cc3Cl)Cl)/NC(=O)c4ccccc4
CCCCCCCN1c2ccccc2/C(=c/3\c(=O)n4c(=NC(=C([C@H]4c5ccccc5)C(=O)OCC)C)s3)/C1=O
CCCCCCCN1c2ccccc2/C(=c/3\c(=O)n4c(=NC(=C([C@@H]4c5ccccc5)C(=O)OCC)C)s3)/C1=O
CCOC(=O)C1=C(N=c2n(c(=O)/c(=C/c3cn(c4c3cccc4)CC=C)/s2)[C@@H]1c5ccc(cc5)C(C)C)C
c1nc2c(c3c(s2)CCCC3)c(n1)SCC(=O)N(C4CCCCC4)C5CCCCC5
c1ccc(c(c1)Cn2cc(c3c2cccc3)C=C4C(=O)OC5(CCCCC5)OC4=O)Cl
COc1cc(ccc1OCc2ccccc2F)C=C3C(=O)OC4(CCCCC4)OC3=O
c1ccc(cc1)COc2ccc3ccccc3c2C=C4C(=O)OC5(CCCCC5)OC4=O
CCOc1cc(ccc1OCc2cccc3c2cccc3)C=C4C(=O)OC5(CCCC5)OC4=O
CCc1c2c3cc(c(cc3c(nc2n(n1)c4ccccc4)c5ccc(c(c5)OCC)OCC)OC)OC
CN(C)c1ccc(cc1)c2c3cc(c(cc3c4c(nn(c4n2)c5ccccc5)Cc6ccccc6)OC)OC
CCCCOc1ccc(cc1)c2c(c(nc(c2C(=O)Nc3ccccc3)C)SCC(=O)c4ccc(c(c4)O)O)C#N
Cc1ccc(cc1)c2cc(c3cc(ccc3n2)Br)C(=O)OCC(=O)c4ccccc4
COc1cc2c(cc1OC)c(nc3c2c(nn3c4ccccc4)c5ccccc5)c6ccc(cc6)[N+](=O)[O-]
COc1cc2c(cc1OC)c(nc3c2c(nn3c4ccccc4)Cc5ccccc5)c6cccnc6
Cc1c(sc[n+]1CC(=O)c2ccc(cc2)Br)CCOC(=O)c3ccccc3
CCOC(=O)c1ccccc1N/C=C(\C#N)/c2nc(cs2)c3ccc(cc3)[N+](=O)[O-]
CCOC(=O)c1ccccc1N/C=C(\C#N)/c2nc(cs2)c3cccc(c3)[N+](=O)[O-]
CCCn1c2ccccc2c3c1nc(nn3)SCC(=O)Nc4nc5ccc(cc5s4)[N+](=O)[O-]
COc1cccc(c1)c2nc3cc(ccc3o2)NC(=S)NC(=O)c4ccc(cc4)[N+](=O)[O-]
c1ccc2c(c1)nc(o2)c3cc(ccc3O)NC(=S)NC(=O)c4ccc(cc4)[N+](=O)[O-]
COc1ccc(cc1)C2(C=Cc3c4ccccc4cc(c3O2)OC)c5ccc(cc5)OC
COc1ccc(c(c1)OC)C2(C=Cc3c4ccccc4ccc3O2)c5ccc(cc5OC)OC
CN1c2ccccc2C3(C=Cc4cc(cc(c4O3)OC)Cl)c5c1cccc5
CCc1ccccc1NCc2ccc(c(c2)OCC)OCc3ccc(cc3)F
```

```
c1ccc(cc1)Oc2ccc(cc2)NCc3ccccc3OCc4ccc(cc4)F
CCOc1ccc(cc1)NCc2ccccc2OCc3cccc(c3)F
CCOc1cc(ccc1OCc2ccccc2F)CNc3ccc(cc3)Oc4ccccc4
CCc1ccc(cc1)NCc2c3ccccc3ccc2OCc4cccc(c4)F
CCOc1ccc(cc1)NCc2ccccc2OCc3cccc4c3cccc4
COc1ccc(cc1)NCc2c3ccccc3ccc2OCc4ccc(cc4)F
Cc1ccc(cc1NCc2c3ccccc3ccc2OCc4ccccc4F)Cl
C=CCn1c(nnc1SCC(=O)Nc2nccs2)CCCOc3ccc(cc3Cl)Cl
C=CCn1c(=O)c2ccccc2nc1SCc3nnc(n3c4ccccc4)SCC=C
CC(C)C(=O)Oc1cc2c(c3c1cccc3)C=CC(O2)(c4ccc(cc4)OC)c5ccc(cc5)OC
COc1ccc(cc1OC)C(=O)Oc2ccc3c(c2)occ(c3=O)c4ccc(cc4)Br
c1ccc(cc1)COc2ccc3ccccc3c2CNc4ccc(cc4)O
c1ccc(c(c1)COc2ccc(cc2CNc3ccc(cc3)O)Br)F
CCOc1ccc2ccc(c(c2c1)/C=C\3/C(=O)N(C(=O)S3)c4ccc(cc4)Cl)OCC
c1ccc2c(c1)cccc2COc3ccc(cc3Br)/C=C\4/C(=O)N(C(=O)S4)c5cccc(c5)Cl
CCOC(=O)c1ccc(cc1)/N=C/2\N(C(=O)/C(=C/c3ccc(c(c3)Cl)OCC(=O)[O-])/S2)C
CCN\1C(=O)/C(=C/c2cc(c(cc2Br)OC)OCC)/S/C1=N/c3ccc(cc3)OCC
COc1cc(ccc1OCc2ccccc2)/C=C\3/C(=O)N(C(=O)S3)c4ccc(cc4)Cl
COc1cc(cc(c1OCC=C)Br)/C=C\2/C(=O)N(C(=O)S2)c3ccc(cc3)Cl
CCOc1cc(cc(c1OCC=C)Br)/C=C\2/C(=O)N(C(=O)S2)c3ccc(cc3)Cl
COc1cc(ccc1OCc2cccc(c2)F)/C=C\3/C(=O)N(C(=O)S3)c4ccc(cc4)Cl
COc1cc(ccc1OCc2ccc(cc2)C(=O)[O-])/C=C\3/C(=O)N(C(=O)S3)c4ccc(cc4)Cl
CCOc1cc(cc(c1OCC=C)I)/C=C\2/C(=O)N(C(=O)S2)c3cccc(c3)Cl
CCOc1cc(cc(c1OC)Br)/C=C\2/C(=O)N(C(=O)S2)c3cccc(c3)Cl
CCOc1cc(cc(c1OCC)Br)/C=C\2/C(=O)N(C(=O)S2)c3cccc(c3)Cl
CCOc1cc(c(cc1/C=C\2/C(=O)N(C(=O)S2)c3cccc(c3)Cl)Br)OCC
CCOc1cc(cc(c1OCC#C)Br)/C=C\2/C(=O)N(C(=O)S2)c3cccc(c3)Cl
c1ccc(c(c1)COc2ccccc2/C=C\3/C(=O)N(C(=O)S3)c4cccc(c4)Cl)C#N
CCOc1c(cc(cc1Br)/C=C\2/C(=O)N(C(=O)S2)c3cccc(c3)Cl)OC
CCOc1cc(ccc1OCc2ccccc2C#N)/C=C\3/C(=O)N(C(=O)S3)c4cccc(c4)Cl
CCOc1cc(c(cc1OC)/C=C\2/C(=O)N(C(=O)S2)c3cccc(c3)Cl)Br
```

```
COc1cc(cc(c1OCC=C)Br)/C=C\2/C(=O)N(C(=O)S2)c3cccc(c3)Cl
CCOc1cc(c(cc1OC)Br)/C=C\2/C(=O)N(C(=O)S2)c3cccc(c3)Cl
Cc1ccc(cc1)COc2ccc(cc2OC)/C=C\3/C(=O)N(C(=O)S3)c4cccc(c4)Cl
C#CCOc1c(cc(cc1Cl)/C=C\2/C(=O)N(C(=O)S2)c3cccc(c3)Cl)Cl
COc1c(cc(cc1Cl)Cl)/C=C\2/C(=O)N(C(=O)S2)c3cccc(c3)Cl
c1ccc(c(c1)COc2ccc(cc2Cl)/C=C\3/C(=O)N(C(=O)S3)c4cccc(c4)Cl)C#N
COc1cc(cc(c1OCC#C)Br)/C=C\2/C(=O)N(C(=O)S2)c3cccc(c3)Cl
c1cc(cc(c1)Cl)N2C(=O)/C(=C/c3ccc(o3)c4ccc(c(c4)C(=O)[O-])Cl)/SC2=O
COc1cc(ccc1OCc2cccc(c2)F)/C=C\3/C(=O)N(C(=O)S3)c4cccc(c4)Cl
COc1cc(cc(c1OCC=C)Cl)/C=C\2/C(=O)N(C(=O)S2)c3cccc(c3)Cl
c1cc(cc(c1)Cl)N2C(=O)/C(=C/c3cc(ccc3OCC(=O)[O-])Br)/SC2=O
c1cc(cc(c1)Cl)N2C(=O)/C(=C/c3ccc(o3)c4ccc(c(c4)Cl)Cl)/SC2=O
c1cc(cc(c1)Cl)N2C(=O)/C(=C/c3ccc(o3)c4ccc(cc4Cl)Cl)/SC2=O
CCN(CC)c1ccc(c(c1)OCC)/C=C\2/C(=O)N(C(=O)S2)c3cccc(c3)Cl
COc1cc(c(cc1OCC(=O)[O-])Br)/C=C\2/C(=O)N(C(=O)S2)c3ccc(cc3)Cl
COc1cc(ccc1OCc2ccccc2C#N)/C=C\3/C(=O)N(C(=O)S3)c4ccc(cc4)Cl
CCOc1c(cc(cc1Br)/C=C\2/C(=O)N(C(=O)S2)c3ccc(cc3)Cl)OC
CCOc1cc(ccc1OCc2ccccc2C#N)/C=C\3/C(=O)N(C(=O)S3)c4ccc(cc4)Cl
CCOc1cc(c(cc1OC)/C=C\2/C(=O)N(C(=O)S2)c3ccc(cc3)Cl)Br
CCOc1cc(cc(c1OC)Br)/C=C\2/C(=O)N(C(=O)S2)c3ccc(cc3)Cl
CCOc1cc(cc(c1OCC)Br)/C=C\2/C(=O)N(C(=O)S2)c3ccc(cc3)Cl
COc1c(cc(cc1Cl)Br)/C=C\2/C(=O)N(C(=O)S2)c3ccc(cc3)Cl
CCOc1cc(c(cc1OC)Br)/C=C\2/C(=O)N(C(=O)S2)c3ccc(cc3)Cl
CCOc1cc(ccc1OCc2ccc(cc2)C)/C=C\3/C(=O)N(C(=O)S3)c4ccc(cc4)Cl
Cc1ccc(cc1)COc2ccc(cc2OC)/C=C\3/C(=O)N(C(=O)S3)c4ccc(cc4)Cl
c1cc(ccc1N2C(=O)/C(=C/c3cc(cc(c3O)Br)Br)/SC2=O)Cl
C#CCOc1c(cc(cc1Cl)/C=C\2/C(=O)N(C(=O)S2)c3ccc(cc3)Cl)Cl
COc1c(cc(cc1Cl)Cl)/C=C\2/C(=O)N(C(=O)S2)c3ccc(cc3)Cl
COc1cc(ccc1OCc2ccc(cc2)Cl)/C=C\3/C(=O)N(C(=O)S3)c4ccc(cc4)Cl
CCOc1cc(ccc1OCc2cccc(c2)F)/C=C\3/C(=O)N(C(=O)S3)c4ccc(cc4)Cl
c1ccc(c(c1)COc2ccc(cc2Cl)/C=C\3/C(=O)N(C(=O)S3)c4ccc(cc4)Cl)C#N
```

```
c1cc(ccc1N2C(=O)/C(=C/c3ccc(o3)c4ccc(c(c4)C(=O)[O-])Cl)/SC2=O)Cl
CCOc1cc(cc(c1OCC=C)Cl)/C=C\2/C(=O)N(C(=O)S2)c3ccc(cc3)Cl
COc1cc(cc(c1OCC(=O)[O-])Cl)/C=C\2/C(=O)N(C(=O)S2)c3ccc(cc3)Cl
CCOc1c(cc(cc1Cl)/C=C\2/C(=O)N(C(=O)S2)c3ccc(cc3)Cl)Cl
Cc1ccc(cc1)n2c(cc(c2C)/C=C\3/C(=O)N(C(=O)S3)c4ccc(cc4)Cl)C
CCOc1ccc(cc1/C=C\2/C(=O)N(C(=O)S2)c3ccc(cc3)Cl)Br
c1cc(ccc1N2C(=O)/C(=C/c3cc(ccc3OCC(=O)[O-])Br)/SC2=O)Cl
CCOc1cc(cc(c1[O-])/C=C\2/C(=O)N(C(=O)S2)c3ccc(cc3)Cl)[N+](=O)[O-]
Cc1cc(c(n1c2cccc(c2)[N+](=O)[O-])C)/C=C\3/C(=O)N(C(=O)S3)c4ccc(cc4)Cl
Cc1cc(c(n1c2ccccc2F)C)/C=C\3/C(=O)N(C(=O)S3)c4ccc(cc4)Cl
Cc1cc(ccc1n2c(cc(c2C)/C=C\3/C(=O)N(C(=O)S3)c4ccc(cc4)Cl)C)[N+](=O)[O-]
Cc1cc(c(n1c2ccc(cc2)Cl)C)/C=C\3/C(=O)N(C(=O)S3)c4ccc(cc4)Cl
Cc1cc(c(n1c2ccc(cc2)Br)C)/C=C\3/C(=O)N(C(=O)S3)c4ccc(cc4)Cl
Cc1ccc(c(c1)C)n2c(cc(c2C)/C=C\3/C(=O)N(C(=O)S3)c4ccc(cc4)Cl)C
COc1cc(ccc1OCc2ccccc2)/C=C\3/C(=O)N(C(=O)S3)c4cccc(c4)Cl
CCOC(=O)c1ccc(cc1)/N=C/2\N(C(=O)/C(=C/c3cc(n(c3C)c4ccccc4F)C)/S2)C
CCOC(=O)c1ccc(cc1)/N=C/2\N(C(=O)/C(=C/c3cc(n(c3C)c4cccc(c4)C)C)/S2)C
CCOC(=O)c1ccc(cc1)/N=C/2\N(C(=O)/C(=C/c3cc(n(c3C)c4ccc(cc4)F)C)/S2)C
CCN\1C(=O)/C(=C/c2cc(c(cc2Cl)OCC)OC)/S/C1=N/c3ccc(cc3)OCC
CCN\1C(=O)/C(=C/c2ccc(c(c2)I)O)/S/C1=N/c3ccc(cc3)OCC
CCN\1C(=O)/C(=C/c2ccc(c(c2)Cl)OCC=C)/S/C1=N/c3ccc(cc3)OCC
CCN\1C(=O)/C(=C/c2ccc(o2)Sc3ccccc3)/S/C1=N/c4ccc(cc4)OCC
CCN\1C(=O)/C(=C/c2ccc(o2)c3ccc(cc3)Cl)/S/C1=N/c4ccc(cc4)OCC
CCN\1C(=O)/C(=C/c2ccccc2OCc3ccccc3C#N)/S/C1=N/c4ccc(cc4)OCC
CN\1C(=O)/C(=C/c2cc(ccc2OC)Br)/S/C1=N/c3ccc(c(c3)C(=O)[O-])Cl
CN\1C(=O)/C(=C/c2cc(c(c(c2)Cl)OCC#C)Cl)/S/C1=N/c3ccc(cc3)C(=O)OC
CN\1C(=O)/C(=C/c2ccc(c(c2)OC)OCC(=O)[O-])/S/C1=N/c3ccc(cc3)C(=O)OC
CN\1C(=O)/C(=C/c2cc(c(c(c2)Cl)OCC=C)OC)/S/C1=N/c3ccc(cc3)C(=O)OC
CCOC(=O)c1ccc(cc1)/N=C/2\N(C(=O)/C(=C/c3cc(cc(c3O)Cl)Br)/S2)C
CCOC(=O)c1ccc(cc1)/N=C/2\N(C(=O)/C(=C/c3ccc(c(c3)[N+](=O)[O-])[O-])/S2)C
CCOC(=O)c1ccc(cc1)/N=C/2\N(C(=O)/C(=C/c3ccc(c(c3)OC)OCC(=O)[O-])/S2)C
```

```
CCOC(=O)c1ccc(cc1)/N=C\2/N(C(=O)/C(=C/c3ccc(c(c3)Cl)OCC#C)/S2)C
CCOC(=O)c1ccc(cc1)/N=C/2\N(C(=O)/C(=C/c3ccccc3OCc4ccccc4C#N)/S2)C
CN\1C(=O)/C(=C/c2cc(c(cc2Br)O)OC)/S/C1=N/c3ccc(c(c3)C(=O)[O-])Cl
CN\1C(=O)/C(=C/c2ccc(c(c2)Br)OC)/S/C1=N/c3ccc(c(c3)C(=O)[O-])Cl
CN\1C(=O)/C(=C/c2cc(c(c(c2)OC)OC)OC)/S/C1=N/c3ccc(c(c3)C(=O)[O-])Cl
CN\1C(=O)/C(=C/c2cc(cc(c2OC)Cl)Cl)/S/C1=N/c3ccc(c(c3)C(=O)[O-])Cl
CN\1C(=O)/C(=C/c2cc(c(c(c2)Cl)O)OC)/S/C1=N/c3ccc(c(c3)C(=O)[O-])Cl
c1ccc(cc1)CN2C(=O)/C(=C/c3cc4c(cc3Br)OCO4)/SC2=O
Cc1ccc(cc1C)/N=C/2\N(C(=O)/C(=C/c3ccc(c(c3)OC)OCC(=O)[O-])/S2)C
CN\1C(=O)/C(=C/c2ccc(cc2OC)OC)/S/C1=N/c3ccc(cc3)Br
COc1cc(c(cc1OCC(=O)[O-])Br)/C=C\2/C(=O)N(C(=O)S2)Cc3ccccc3
COc1cc(c(cc1O)Br)/C=C\2/C(=O)N(C(=O)S2)Cc3ccccc3
CCOc1ccc(cc1)N2C(=O)/C(=C/c3ccccc3OCc4ccc(cc4)C(=O)[O-])/NC2=S
Cc1ccc(cc1)/N=C/2\N(C(=O)/C(=C/c3ccccc3OCc4ccccc4C#N)/S2)C
Cc1ccc(cc1C)/N=C/2\N(C(=O)/C(=C/c3cc(c(c(c3)Cl)OCC=C)OC)/S2)C
COc1cc(cc(c1OCC(=O)[O-])Cl)/C=C\2/C(=O)N(C(=O)S2)Cc3ccccc3Cl
COc1cc(c(cc1O)Br)/C=C\2/C(=O)N(C(=O)S2)Cc3ccc(cc3Cl)Cl
COc1ccc2ccc(c(c2c1)/C=C\3/C(=O)N(C(=O)S3)Cc4ccc(c(c4)Cl)Cl)OC
CCOc1cc(cc(c1OCC#C)I)/C=C\2/C(=O)N(C(=S)N2)C
CCCN1C(=O)/C(=C/c2cc(c(c(c2)Cl)OCc3ccccc3C#N)OCC)/SC1=O
CCOC(=O)C1=C(N=c2n(c(=O)/c(=C/c3ccc(o3)[N+](=O)[O-])/s2)[C@H]1c4cccc(c4)OC)c5ccccc5
CCOC(=O)C1=C(N=c2n(c(=O)/c(=C/c3ccc(o3)[N+](=O)[O-])/s2)[C@@H]1c4cccc(c4)OC)c5ccccc5
CCN(CC)c1ccc(o1)/C=c\2/c(=O)n3c(=NC(=C([C@H]3c4cccc(c4)OC)C(=O)OCC)c5ccccc5)s2
CCN(CC)c1ccc(o1)/C=c\2/c(=O)n3c(=NC(=C([C@@H]3c4cccc(c4)OC)C(=O)OCC)c5ccccc5)s2
CCOC(=O)C1=C(N=c2n(c(=O)/c(=C/c3ccc(c(c3)O)OC)/s2)[C@H]1c4cccc(c4)OC)c5ccccc5
CCOC(=O)C1=C(N=c2n(c(=O)/c(=C/c3ccc(c(c3)O)OC)/s2)[C@@H]1c4cccc(c4)OC)c5ccccc5
CCOC(=O)C1=C(N=c2n(c(=O)/c(=C/c3ccc(cc3OC)OC)/s2)[C@H]1c4cccc(c4)OC)c5ccccc5
CCOC(=O)C1=C(N=c2n(c(=O)/c(=C/c3ccc(cc3OC)OC)/s2)[C@@H]1c4cccc(c4)OC)c5ccccc5
CCOC(=O)C1=C(N=c2n(c(=O)/c(=C/c3cc(ccc3OC)OC)/s2)[C@H]1c4cccc(c4)OC)c5ccccc5
CCOC(=O)C1=C(N=c2n(c(=O)/c(=C/c3cc(ccc3OC)OC)/s2)[C@@H]1c4cccc(c4)OC)c5ccccc5
CCOC(=O)C1=C(N=c2n(c(=O)/c(=C/c3cc(ccc3[O-])[N+](=O)[O-])/s2)[C@H]1c4cccc(c4)OC)c5ccccc5
```

```
CCOC(=O)C1=C(N=c2n(c(=O)/c(=C/c3cc(ccc3[O-])[N+](=O)[O-])/s2)[C@@H]1c4cccc(c4)OC)c5ccccc5
Cc1ccc(cc1)COc2ccc(cc2)/C=C\3/C(=O)N(C(=O)S3)CC(=O)N4CCOCC4
CC(C)(C)c1cc(cc(c1O)C(C)(C)C)/C=C\2/C(=O)N(C(=O)S2)CC(=O)c3ccc(cc3)Cl
c1cc(ccc1COc2ccc(cc2)/C=C\3/C(=O)N(C(=O)S3)CC(=O)N4CCOCC4)Br
COc1ccc(cc1Br)/C=C\2/C(=O)N(C(=O)S2)CC(=O)N3CCOCC3
CCOc1cc(cc(c1OCC)Br)/C=C\2/C(=O)N(C(=O)S2)CC(=O)N3CCOCC3
CCOC(=O)C1=C(N=c2n(c(=O)/c(=C/c3cc4c(cc3Cl)OCO4)/s2)[C@H]1c5cccs5)c6ccccc6
CCOC(=O)C1=C(N=c2n(c(=O)/c(=C/c3cc4c(cc3Cl)OCO4)/s2)[C@@H]1c5cccs5)c6ccccc6
CCCOc1ccc(cc1OC)/C=C\2/C(=NN(C2=O)c3ccc(cc3)C(=O)OCC)C(F)(F)F
Cc1cc(c(n1c2c(c3c(s2)CCCC3)C#N)C)/C=N/NC(=O)Cc4cccc(c4)C(F)(F)F
CCOc1cc(ccc1OCC#C)/C=C\2/C(=NN(C2=O)c3ccc(cc3)C(=O)OCC)C(F)(F)F
CC1(CC2=C(C(C3=C(N2C)CC(CC3=O)(C)C)c4cc(ccc4OCC#C)Br)C(=O)C1)C
CCCCC(=O)Nc1cccc(c1)NC(=S)NC(=O)c2ccc(cc2)c3ccccc3
CCCCC(=O)Nc1cccc(c1)NC(=S)NC(=O)c2cc(ccc2OC)Br
CCCCC(=O)Nc1cccc(c1)NC(=S)NC(=O)c2ccc(cc2Cl)Cl
CCCCOc1cccc(c1)C(=O)NC(=S)Nc2ccccc2C(=O)N
Cc1cc2c(cc1C)nc([nH]2)SCC(=O)Nc3cc(c(c(c3)Br)Br)Br
Cc1ccccc1NC(=O)CN2C(=O)/C(=C/c3ccc(o3)c4cccc(c4C)C(=O)[O-])/SC2=O
Cc1ccccc1NC(=O)CN2C(=O)/C(=C/c3ccc(o3)c4cc(ccc4C)C(=O)[O-])/SC2=O
Cc1ccccc1NC(=O)CN2C(=O)/C(=C/c3ccc(o3)c4cc(ccc4Cl)C(=O)[O-])/SC2=O
c1ccc(cc1)NC(=O)CN2C(=O)/C(=C/c3ccc(o3)c4cc(ccc4Cl)C(=O)[O-])/SC2=O
CCOc1cc(ccc1OC(C)C)/C=C/2\C(=O)N(C(=O)S2)CC(=O)N3CCOCC3
c1ccc(c(c1)C#N)c2ccc(o2)/C=C/3\C(=O)N(C(=O)S3)CC(=O)N4CCOCC4
COc1cc(cc(c1OCC#C)I)/C=C/2\C(=O)N(C(=O)S2)Cc3ccccc3F
CC(=O)c1ccc(cc1)c2ccc(o2)/C=C/3\C(=O)N(C(=O)S3)Cc4ccccc4F
c1ccc(c(c1)CN2C(=O)/C(=C/c3ccc(o3)c4ccc(c(c4)Cl)C(=O)[O-])/SC2=O)F
CC(=O)c1ccc(cc1)c2ccc(o2)/C=C/3\C(=O)N(C(=O)S3)Cc4ccc(cc4)F
Cc1c(cccc1C(=O)[O-])c2ccc(o2)/C=C\3/C(=O)N(C(=O)S3)Cc4ccc(cc4)F
c1cc(ccc1CN2C(=O)/C(=C/c3ccc(o3)c4ccc(c(c4)Cl)C(=O)[O-])/SC2=O)F
COc1cc(cc(c1OCC#C)I)/C=C/2\C(=O)N(C(=O)S2)Cc3ccccc3
COc1cc(cc(c1OCC#C)/C=C/2\C(=O)N(C(=O)S2)Cc3ccccc3)Br
```

```
c1ccc(cc1)CN2C(=O)/C(=C/c3ccc(o3)c4ccc(c(c4)Cl)C(=O)[O-])/SC2=O
Cc1ccc(cc1C)NC(=O)CN2C(=O)/C(=C/c3ccc(o3)c4cccc(c4C)C(=O)[O-])/SC2=O
Cc1ccc(cc1C)NC(=O)CN2C(=O)/C(=C/c3ccc(o3)c4cc(ccc4Cl)C(=O)[O-])/SC2=O
CN(C)c1ccc2c(c1)OC(=C([C@@H]2c3cc(ccc3OCc4ccccc4)Br)C(=O)OC)N
CN(C)c1ccc2c(c1)OC(=C([C@H]2c3cc(c(c(c3)Cl)OCc4ccccc4F)Cl)C(=O)OC)N
CN(C)c1ccc2c(c1)OC(=C([C@@H]2c3cc(c(c(c3)Cl)OCc4ccccc4F)Cl)C(=O)OC)N
Cc1ccc(cc1)N(CC(=O)NCCSCc2ccccc2Cl)S(=O)(=O)C
CC1(CC2=C([C@@H](C(=C(N2Nc3ccccc3)N)C#N)c4ccc(c(c4)OC)OCc5cccc(c5)F)C(=O)C1)C
CC1(CC2=C([C@H](C(=C(N2Nc3ccccc3)N)C#N)c4ccc(c(c4)OC)OCC=C)C(=O)C1)C
CC1(CC2=C([C@@H](C(=C(N2Nc3ccccc3)N)C#N)c4ccc(c(c4)OC)OCC=C)C(=O)C1)C
CC1(CC2=C([C@@H](C(=C(N2Nc3ccccc3)N)C#N)c4ccc(c(c4)OC)OCc5ccccc5)C(=O)C1)C
CC1(CC2=C([C@H](C(=C(N2Nc3ccccc3)N)C#N)c4cc(c(c(c4)Br)OCC=C)OC)C(=O)C1)C
CC1(CC2=C([C@@H](C(=C(N2Nc3ccccc3)N)C#N)c4cc(c(c(c4)Br)OCC=C)OC)C(=O)C1)C
Cc1ccc(cc1)CN2C(=O)/C(=C/c3ccc(o3)c4ccc(c(c4)Cl)C(=O)[O-])/SC2=O
CCOc1cc(cc(c1O)Br)/C=C/2\C(=O)N(C(=O)S2)Cc3c(cccc3Cl)F
c1ccc(c(c1)Cn2cc(c3c2cccc3)/C=C/4\C(=O)N(C(=O)S4)Cc5c(cccc5Cl)F)Cl
COc1cc(ccc1OCc2ccccc2F)/C=C/3\C(=O)N(C(=O)S3)Cc4c(cccc4Cl)F
CCOc1cc(cc(c1OC)I)/C=C/2\C(=O)N(C(=O)S2)Cc3c(cccc3Cl)F
c1cc(cc(c1)Cl)COc2ccc(cc2/C=C/3\C(=O)N(C(=O)S3)Cc4c(cccc4Cl)F)Cl
c1ccc(cc1)COc2ccc(cc2)/C=C/3\C(=O)N(C(=O)S3)Cc4c(cccc4Cl)F
COc1cc(cc(c1OCC#C)I)/C=C/2\C(=O)N(C(=O)S2)Cc3cc4c(cc3Cl)OCO4
Cc1cccc(c1)COc2ccc(cc2OC)/C=C\3/C(=O)N(C(=O)S3)Cc4cc5c(cc4Cl)OCO5
COc1cc(cc(c1OCC#C)Br)/C=C\2/C(=O)N(C(=O)S2)Cc3cc4c(cc3Cl)OCO4
c1ccc(cc1)COc2ccccc2/C=C/3\C(=O)N(C(=O)S3)Cc4cc5c(cc4Cl)OCO5
COc1cc(ccc1OCc2ccc(cc2Cl)Cl)/C=C/3\C(=O)N(C(=O)S3)Cc4cc5c(cc4Cl)OCO5
COc1cc(ccc1OCc2ccc(cc2)Cl)/C=C/3\C(=O)N(C(=O)S3)Cc4cc5c(cc4Cl)OCO5
CCc1ccc(cc1)C(=O)NC(=S)Nc2ccc(c(c2)c3nc4ccccc4s3)O
Cc1cc(cc(c1NC(=O)c2ccc(o2)c3ccc(c(c3)Cl)Cl)C)Br
Cc1cccc(c1C)c2nc3cc(ccc3o2)NC(=S)NC(=O)c4cccc(c4)Br
CCCOc1c(cc(cc1Br)C(=O)Nc2ccccc2C(F)(F)F)OC
CC(C)Oc1ccc(cc1)c2cc(=O)c3c(cc(c(c3o2)I)OC(C)C)OC(C)C
```

```
CC(C)Oc1ccc(cc1)C(=O)Oc2c(c(cc(c2I)OC(C)C)OC(C)C)C(=O)C
CCCCOc1ccc(cc1)NC(=O)CSc2nnc(n2N)c3ccc(cc3Cl)Cl
c1ccc(cc1)C(c2ccccc2)NC(=O)c3cc(cc(c3)C(F)(F)F)C(F)(F)F
CC1=C(S[C@@]2(N1Cc3ccccc3)N(N=C(S2)C(=O)OC)c4ccccc4Cl)C(=O)C
CC1=C(S[C@]2(N1Cc3ccccc3)N(N=C(S2)C(=O)OC)c4ccccc4Cl)C(=O)C
Cc1c(nc(s1)N(c2cccc3c2cccc3)C(=O)CCc4ccccc4)c5ccc(cc5)OC
CCCc1c(nc(s1)N(c2ccc(cc2)Br)C(=O)CC)c3ccc(cc3)OCC
CCCC(=O)N(c1ccc(cc1)Br)c2nc(c(s2)CC)c3ccc(cc3)C
CCc1ccccc1NC(=O)CSc2nc3c(c4c(s3)C[C@H](CC4)C(C)(C)C)c(=O)n2Cc5ccccc5
CCc1ccccc1NC(=O)CSc2nc3c(c4c(s3)C[C@@H](CC4)C(C)(C)C)c(=O)n2Cc5ccccc5
C1CCC(CC1)NC(=O)CSc2nc3c(c4c(s3)CCCC4)c(=O)n2C5CCCCC5
CCc1c(nc(s1)N(c2ccc(cc2Cl)Cl)C(=O)CC)c3ccc(cc3)OC
Cc1ccc2c(c1)[C@H](CC(N2C(=O)c3cc(cc(c3)C(F)(F)F)C(F)(F)F)(C)C)C
Cc1ccc2c(c1)[C@@H](CC(N2C(=O)c3cc(cc(c3)C(F)(F)F)C(F)(F)F)(C)C)C
CCc1c(nc(s1)N(c2ccc(cc2Cl)Cl)C(=O)CCc3ccccc3)c4ccc(cc4)Cl
Cc1c(nc(s1)N(c2ccccc2Cl)C(=O)CCc3ccccc3)c4ccc(cc4)OC
CCc1c(nc(s1)N(c2ccc(cc2C)C)C(=O)Cc3ccccc3)c4ccc(cc4)Cl
CCCCC(=O)N(c1ccc(cc1)Cl)c2nc(c(s2)CCC)c3ccc(cc3)OC
CCCc1c(nc(s1)N(c2ccc(c(c2)Cl)Cl)C(=O)CC)c3ccc(cc3)Cl
Cc1ccccc1OCc2nnc(n2Cc3ccccc3)SCC(=O)Nc4ccc(cc4Cl)Cl
COc1ccccc1n2c(=O)c3ccccc3nc2SCC(=O)c4ccc5c(c4)CCCC5
CCc1c(nc(s1)N(c2ccc(cc2)Cl)C(=O)C3CCCCC3)c4ccc(cc4)OCC
C[C@@H](C(=O)Nc1nccs1)Sc2nnc(n2c3ccccc3)c4ccc(cc4)C(C)(C)C
C[C@H](C(=O)Nc1nccs1)Sc2nnc(n2c3ccccc3)c4ccc(cc4)C(C)(C)C
CCc1c(nc(s1)N(c2cccc(c2)F)C(=O)Cc3ccccc3)c4ccc(cc4)C
CCCc1c(nc(s1)N(c2ccccc2F)C(=O)Cc3ccccc3)c4ccc(cc4)OC
CCOc1ccc(cc1)NC(=O)[C@@H](C)Sc2nnc(n2c3ccccc3)c4ccc(cc4)Cl
CCOc1ccc(cc1)NC(=O)[C@H](C)Sc2nnc(n2c3ccccc3)c4ccc(cc4)Cl
CCCCC(=O)N(c1ccc(cc1)OC)c2nc(c(s2)CCC)c3ccc(cc3)OC
CCc1c(nc(s1)N(c2cccc(c2)F)C(=O)C3CCCCC3)c4ccc(cc4)Cl
CCc1c(nc(s1)N(c2ccc(cc2Cl)Cl)C(=O)Cc3ccccc3)c4ccc(cc4)OC
```

```
CCc1c(nc(s1)N(c2cccc(c2)Br)C(=O)C3CCCCC3)c4ccc(cc4)Cl
CCc1c(nc(s1)N(c2ccc(cc2)Br)C(=O)Cc3ccccc3)c4ccc(cc4)OC
CCCC(=O)N(c1ccc(cc1)Br)c2nc(c(s2)CC)c3ccc(cc3)Cl
CCCCC(=O)N(c1ccc(c(c1)Cl)Cl)c2nc(c(s2)CCC)c3ccccc3
CCc1c(nc(s1)N(c2cccc(c2)Br)C(=O)C3CCCCC3)c4ccc(cc4)OC
CCc1c(nc(s1)N(c2cccc(c2)Br)C(=O)Cc3ccccc3)c4ccc(cc4)C
COc1ccccc1C(=O)Nc2cc(ccc2Oc3ccc(cc3)C4CCCCC4)C(F)(F)F
c1ccc(cc1)CC(C(=O)Nc2cc(ccc2Cl)C(F)(F)F)C(=O)Nc3cc(ccc3Cl)C(F)(F)F
CCCc1c(nc(s1)N(c2ccc(c(c2)Cl)Cl)C(=O)CCC)c3ccc(cc3)OCC
CC1=C(S[C@@]2(N1C)N(N=C(S2)C(=O)C)c3c(cccc3Cl)Cl)C(=O)OC
CC1=C(S[C@]2(N1C)N(N=C(S2)C(=O)C)c3c(cccc3Cl)Cl)C(=O)OC
Cc1c(nc(s1)N(c2ccccc2Br)C(=O)Cc3ccccc3)c4ccccc4
CCCc1c(nc(s1)N(c2ccc(cc2)Br)C(=O)CCc3ccccc3)c4ccc(cc4)OC
CCc1c(nc(s1)N(c2ccc(cc2C)C)C(=O)C3CCCCC3)c4ccc(cc4)Cl
CCc1c(nc(s1)N(c2cccc(c2)Br)C(=O)CCc3ccccc3)c4ccc(cc4)Cl
Cc1cccc(c1)N2C(=O)c3ccccc3N[C@]24c5ccccc5N(C4=O)Cc6c(cccc6Cl)Cl
Cc1cccc(c1)N2C(=O)c3ccccc3N[C@@]24c5ccccc5N(C4=O)Cc6c(cccc6Cl)Cl
CCc1c(nc(s1)N(c2ccc3ccccc3c2)C(=O)C4CCCCC4)c5ccc(cc5)OC
c1ccc(cc1)CN2C(=O)c3ccccc3N[C@]24c5ccccc5N(C4=O)C/C=C/c6ccccc6
c1ccc(cc1)CN2C(=O)c3ccccc3N[C@@]24c5ccccc5N(C4=O)C/C=C/c6ccccc6
CCC(=O)N(c1cccc(c1)Br)c2nc(c(s2)C)c3ccc(cc3)OCC
CCc1c(nc(s1)N(c2cccc(c2)F)C(=O)C3CCCCC3)c4ccc(cc4)OC
CCCCC(=O)N(c1cccc2c1cccc2)c3nc(c(s3)C)c4ccc(cc4)Cl
CCOc1ccc(cc1)NC(=O)CSc2nnc(n2c3ccccc3)c4ccc(cc4)C(C)(C)C
CCc1c(nc(s1)N(c2ccc(cc2Cl)Cl)C(=O)Cc3ccccc3)c4ccc(cc4)C
CCCc1c(nc(s1)N(c2ccc(c(c2)Cl)Cl)C(=O)Cc3ccccc3)c4ccc(cc4)OCC
CC(C)(C)c1ccc(cc1)c2nnc(n2c3ccccc3)SCC(=O)Nc4cccc(c4)C(F)(F)F
CCOC(=O)C1=C(N([C@@]2(S1)N(N=C(S2)C(=O)OC)c3ccc(cc3)Cl)Cc4ccccc4)C
CCOC(=O)C1=C(N([C@]2(S1)N(N=C(S2)C(=O)OC)c3ccc(cc3)Cl)Cc4ccccc4)C
CCCc1c(nc(s1)N(c2ccc(c(c2)C)C)C(=O)Cc3ccccc3)c4ccccc4
Cc1ccc(cc1C)OCc2nnc(n2c3ccccc3)SCC(=O)n4c5c(c6c4CCCC6)CCCC5
```

```
CCc1c(nc(s1)N(c2ccccc2OCC)C(=O)C3CCCC3)c4ccc(cc4)C
CCCc1c(nc(s1)N(c2ccc(cc2)C(=O)OC)C(=O)CCC)c3ccc(cc3)OCC
CCOC(=O)C1=C(N([C@]2(S1)N(N=C(S2)C(=O)OC)c3ccc(cc3)C)Cc4ccccc4)C
Cn1c(nnc1SCC(=O)c2ccc(cc2)c3ccccc3)COc4ccc(cc4)C5CCCCC5
CCc1c(nc(s1)N(c2ccccc2Cl)C(=O)C3CCCC3)c4ccc(cc4)Cl
Cc1ccc(cc1)N2[C@]3(N(C(=C(S3)C(=O)OC)C)Cc4ccccc4)SC(=N2)C(=O)C
Cc1ccc(cc1)N2[C@@]3(N(C(=C(S3)C(=O)OC)C)Cc4ccccc4)SC(=N2)C(=O)C
CCCc1c(nc(s1)N(c2ccccc2C)C(=O)Cc3ccccc3)c4ccc(cc4)OC
CCc1c(nc(s1)N(c2ccc3ccccc3c2)C(=O)CCc4ccccc4)c5ccc(cc5)Cl
Cc1ccccc1C(=O)OCCCNC2=NS(=O)(=O)c3c2cccc3
CCc1c(nc(s1)N(c2ccc(cc2C)C)C(=O)CCc3ccccc3)c4ccc(cc4)OC
CCc1c(nc(s1)N(c2ccc(cc2)C(=O)OC)C(=O)Cc3ccccc3)c4ccc(cc4)OCC
Cc1cc(c(c(c1)C)C(=O)CSc2nnc(n2N)c3ccc(cc3Cl)Cl)C
Cc1cccc(c1OCCNC(=O)c2ccccc2OCC(=O)N3CCc4c3cccc4)C
Cc1cccc(c1OCCNC(=O)c2ccccc2OCC(=O)Nc3cccc(c3)OC)C
CCc1cccc(c1NC(=O)COc2ccccc2C(=O)Nc3cccc(c3Cl)Cl)C
C/C=C\1/C(=O)N(C(=S)N1)C2CCCCC2
c1ccc(cc1)C([C@@H]2c3cccc4c3c(ccc4)C2=O)[C@@H]5c6cccc7c6c(ccc7)C5=O
Cc1ccccc1C[C@]2(N(c3ccccc3S2)Cc4ccccc4C)c5nc6ccccc6s5
Cc1ccccc1C[C@@]2(N(c3ccccc3S2)Cc4ccccc4C)c5nc6ccccc6s5
c1ccc(cc1)c2cn3c4ccccc4sc3c2[C@@H](CC(=O)c5ccccc5)C(=O)c6ccccc6
c1ccc(cc1)c2cn3c4ccccc4sc3c2[C@H](CC(=O)c5ccccc5)C(=O)c6ccccc6
c1ccc(cc1)c2cc(c(nn2)c3ccccc3)c4c(cn5c4sc6c5cccc6)c7ccccc7
c1ccc(cc1)C2=C(C(=O)[C@@]3([C@H]2c4cccc5c4c3ccc5)[NH+]6CCCC6)c7ccccc7
c1ccc(cc1)C2=C(C(=O)[C@]3([C@H]2c4cccc5c4c3ccc5)[NH+]6CCCC6)c7ccccc7
COC(=O)c1c(c(c2c(c1c3ccccc3)nsn2)c4ccccc4)c5ccccc5
COc1ccc(cc1)C(=O)c2c(nc(s2)Nc3ccc(cc3)Cl)c4ccccc4
Cc1c(c2cc(ccc2o1)[N-]S(=O)(=O)c3cccs3)C(=O)OCCOC
CCCCOC(=O)c1c(oc2c1cc(cc2)NS(=O)(=O)c3cccs3)C
COc1cccc(c1OCc2ccccc2F)CNc3ccccc3O
c1ccc2c(c1)nc(s2)c3cc(ccc3O)NC(=O)c4cc(ccc4Cl)Cl
```

```
CCc1cc2c(cc1OC(=O)c3ccc(c(c3)OC)OC)oc(c(c2=O)c4ccc5c(c4)OCCCO5)C
C[C@H]1C[C@H](c2ccccc2N1C(=O)c3ccc(cc3)F)N(c4ccccc4)C(=O)c5ccc(cc5)F
C[C@@H]1C[C@H](c2ccccc2N1C(=O)c3ccc(cc3)F)N(c4ccccc4)C(=O)c5ccc(cc5)F
C[C@H]1C[C@@H](c2ccccc2N1C(=O)c3ccc(cc3)F)N(c4ccccc4)C(=O)c5ccc(cc5)F
C[C@@H]1C[C@@H](c2ccccc2N1C(=O)c3ccc(cc3)F)N(c4ccccc4)C(=O)c5ccc(cc5)F
CC1=CC(N(c2c1cc(cc2)OC(=O)c3ccc(cc3)F)C(=O)c4ccc(cc4)F)(C)C
CCc1cc2c(cc1OC(=O)c3cc(c(c(c3)OC)OC)OC)occ(c2=O)c4nc5ccccc5s4
CCOc1ccc2c(c1)/C(=C/3\SC(=C(S3)C(=O)OC)c4ccccc4)/C(=S)C(N2)(C)C
c1ccc2c(c1)ccc3c2C=CC4(O3)c5ccccc5Oc6c4cccc6
c1ccc(cc1)c2c3c4nc(nn4cnc3oc2c5ccccc5)CSc6nc7ccccc7s6
CCOC(=O)C1=C(N=c2n(c(=O)/c(=C/3\c4ccccc4N(C3=O)C)/s2)[C@@H]1c5ccc(c(c5)OC)OC)C
CCOC(=O)C1=C(N=c2n(c(=O)/c(=C/3\c4ccccc4N(C3=O)CC(=O)N)/s2)[C@H]1c5ccccc5)C
CCOC(=O)C1=C(N=c2n(c(=O)/c(=C/3\c4ccccc4N(C3=O)CC(=O)N)/s2)[C@@H]1c5ccccc5)C
COc1ccc(cc1)NC(=O)c2ccc(cc2)Nc3nc4ccc(cc4c(n3)c5ccccc5)Br
COc1ccc(cc1OC)Cc2nc3c4c(c(oc4ncn3n2)c5ccccc5)c6ccccc6
CCOc1cc(ccc1OC)[C@H]2C(=C(N=c3n2c(=O)/c(=C/c4ccc(cc4)F)/s3)C)C(=O)OCC
CCOc1cc(ccc1OC)[C@@H]2C(=C(N=c3n2c(=O)/c(=C/c4ccc(cc4)F)/s3)C)C(=O)OCC
CCOC(=O)c1ccc(cc1)c2ccc(o2)/C=c/3\c(=O)n4c(=NC(=C([C@H]4c5cccs5)C(=O)OCC)C)s3
CCOC(=O)c1ccc(cc1)c2ccc(o2)/C=c/3\c(=O)n4c(=NC(=C([C@@H]4c5cccs5)C(=O)OCC)C)s3
CC(C)Nc1nc(nc(n1)OCCNC(=O)COc2ccc(cc2Cl)Cl)NC(C)C
CC1=C([C@H](n2c(=O)/c(=C/c3cn(c4c3cccc4)CC=C)/sc2=N1)c5ccc(cc5)C(C)C)C(=O)OC
COc1ccc(cc1)C2(C=Cc3c(ccc4c3cc(cc4)O)O2)c5ccc(cc5)OC
COc1ccc(cc1OC)CC[NH2+]Cc2cccnc2
Cc1ccc(cc1)C[n+]2c(c(cc3c2nc4ccccn4c3=O)C(=O)NCCCOC)N
Cc1c(c2cc(ccc2o1)NS(=O)(=O)c3ccc4ccccc4c3)C(=O)OCCOC
CCCCOC(=O)c1c(oc2c1cc(cc2)NS(=O)(=O)c3ccc(cc3)OCC)C
CCCCCOC(=O)c1c(oc2c1cc(cc2)NS(=O)(=O)c3ccc(cc3)OC)C
Cc1cc(c(cc1C)S(=O)(=O)Nc2cc3c(c(oc3c4c2cccc4)C)C(=O)OCCOC)C
CCCC(=O)N(c1ccc2c(c1)c(c(o2)C)C(=O)OC)S(=O)(=O)c3ccc(cc3)OCC
CCOC(=O)c1c(oc2c1cc(c3c2cccc3)NS(=O)(=O)c4cc(ccc4C)C)C
CCOc1ccc(cc1)S(=O)(=O)Nc2cc3c(c(oc3c4c2cccc4)C)C(=O)OC(C)C
```

```
Cc1c(c2cc(c3ccccc3c2o1)NS(=O)(=O)c4ccc(cc4)Cl)C(=O)OC(C)C
Cc1c(c2cc(c3ccccc3c2o1)NS(=O)(=O)c4ccc(cc4)F)C(=O)OC(C)C
Cc1c(c2cc(ccc2o1)NS(=O)(=O)c3ccc(cc3)C(C)(C)C)C(=O)OC(C)C
Cc1cc(c(c(c1)C)S(=O)(=O)Nc2ccc3c(c2)c(c(o3)C)C(=O)OCCOC)C
Cc1c(c2cc(ccc2o1)N(C(=O)C)S(=O)(=O)c3ccc(cc3)C(C)(C)C)C(=O)C
CCCCOC(=O)c1c(oc2c1cc(cc2)NS(=O)(=O)c3ccc4ccccc4c3)C
Cc1cc(c(cc1C)S(=O)(=O)Nc2cc3c(c(oc3c(c2)Br)C)C(=O)C)C
Cc1cc(c(cc1C)S(=O)(=O)Nc2cc3c(c(oc3c4c2cccc4)C)C(=O)OC)C
Cc1c(c2cc(c3ccccc3c2o1)NS(=O)(=O)c4cccs4)C(=O)OCCOC
CCOc1ccc(cc1)S(=O)(=O)Nc2cc3c4c(oc3c5c2cccc5)CCCC4=O
CC(=O)N(c1ccc2c(c1)c3c(o2)CCCC3)S(=O)(=O)c4ccc(cc4)Br
CCOC(=O)c1c(oc2c1cc(cc2)N(C(=O)C)S(=O)(=O)c3c(cc(cc3C)C)C)C
CCOc1ccc(cc1)S(=O)(=O)N(c2ccc3c(c2)c(c(o3)C)C(=O)C)C(=O)c4ccncc4
COc1ccc(cc1)S(=O)(=O)Nc2cc3c4c(oc3c5c2cccc5)CCCC4=O
CCOC(=O)c1c(oc2c1cc(c3c2cccc3)NS(=O)(=O)c4ccc(cc4)OC)C
CCOC(=O)c1c(oc2c1cc(c3c2cccc3)NS(=O)(=O)c4ccc(cc4)F)C
Cc1c(c2cc(ccc2o1)NS(=O)(=O)c3ccc(cc3)Br)C(=O)OC(C)C
CCCCOC(=O)c1c(oc2c1cc(cc2)NS(=O)(=O)c3ccc(cc3)Br)C
CCCCOC(=O)c1c(oc2c1cc(c3c2cccc3)NS(=O)(=O)c4ccc(cc4)OC)C
Cc1c(c2cc(ccc2o1)N(C(=O)C)S(=O)(=O)c3ccc(cc3)Br)C(=O)OC
Cc1c(c2cc(c3ccccc3c2o1)NS(=O)(=O)c4ccc(cc4)C(C)C)C(=O)OC
CCOc1ccc(cc1)S(=O)(=O)Nc2cc3c4c(oc3c5c2cccc5)CC(CC4=O)(C)C
Cc1cc2ccccc2n3c1nnc3SCC(=O)Nc4ccc(cc4)S(=O)(=O)N
c1cc2c(ccc3c2c(c1)C(=O)S3)S(=O)(=O)Nc4ccc(cc4)Br
Cc1ccc(c(c1)C)S(=O)(=O)Nc2ccc3c4c2C(=O)c5ccccc5-c4cc(=O)n3C
Cn1c2ccc(c3c2c(cc1=O)-c4ccccc4C3=O)NS(=O)(=O)c5ccc(cc5)Cl
c1ccc(cc1)CN(Cc2ccccc2)S(=O)(=O)c3ccc4c5c3cccc5C(=O)N4
Cc1c(c2cc(ccc2o1)NS(=O)(=O)c3cccc4c3cccc4)C(=O)OC(C)C
c1cc(ccc1CNc2ccc(cc2)OC(F)(F)F)OCc3ccc(cc3)F
COc1ccc2c(c1)C=C[C@@H](O2)c3ccc(cc3)[N+](=O)[O-]
c1ccc(cc1)c2ccc(cc2)C(=O)CSc3nnc(n3c4ccccc4)c5ccncc5
```

```
COc1ccc(cc1OC)NC(=O)c2ccccc2OCc3ccc(cc3)F
CCCNC(=O)[C@]12CC[C@]([C@@H]1Br)(C2(C)C)C(Br)Br
CC1([C@]2(CC[C@@]1([C@@H]2Br)C(=O)NCc3ccc(cc3)OC)C(Br)Br)C
c1ccc(cc1)CCNC(=O)/C(=C/c2ccc(o2)c3ccccc3Cl)/NC(=O)c4ccccc4
Cn1c(nnc1SCC(=O)c2ccc(cc2)Cl)c3cc(c(c(c3)OC)OC)OC
Cc1c(c2cc(ccc2o1)N(C(=O)C3CCCCC3)S(=O)(=O)c4ccc(cc4)OC)C(=O)C
CCOc1ccc(cc1)S(=O)(=O)N(c2ccc3c(c2)c(c(o3)C)C(=O)C)C(=O)C4CCCCC4
c1cc(ccc1c2cc(n3c(n2)c(cn3)C(=O)Nc4ccc(cc4)Br)C(F)(F)F)Br
CCOc1ccc(cc1)C[NH+](CCc2ccc3c(c2)OCO3)Cc4cc(c(cc4OC)OC)OC
CCOC(=O)c1c2c(sc1NC(=S)NC(=O)c3c(cn(n3)C)Cl)C[C@@H](CC2)C
c1ccc(c(c1)c2nc3cc(ccc3o2)NC(=O)c4cc(ccc4Cl)Br)F
COc1ccc(cc1)NC(=O)c2ccc(cc2)Nc3nc4ccc(cc4c(n3)c5ccccc5)Cl
c1ccc2c(c1)nc(o2)SCC(=O)Nc3ncc(s3)Cc4ccc(cc4Cl)Cl
CN1c2ccccc2-c3ccccc3[C@]14C=Cc5cc(ccc5O4)[N+](=O)[O-]
CN1c2ccccc2-c3ccccc3[C@@]14C=Cc5cc(ccc5O4)[N+](=O)[O-]
Cc1c(c2cc(ccc2o1)N(C(=O)c3ccccc3)S(=O)(=O)c4cccs4)C(=O)OC
C=CCOc1ccccc1CNc2ccccc2F
C=CCOc1ccccc1CNc2cccc(c2)F
Cc1cccc(c1)NCc2ccccc2OCC=C
c1ccc(cc1)Cc2nc3c(cnn3c4ccccc4)c(=O)n2c5cccc6c5cccc6
CCCCOc1cccc(c1)C(=O)Nc2cc(ccc2F)[N+](=O)[O-]
CC(C)c1ccc(cc1)N(CC(=O)NCCc2ccc(cc2)OC)S(=O)(=O)C
CCOc1ccccc1N(CC(=O)NCCc2ccc(cc2)OC)S(=O)(=O)C
CCc1ccc(cc1)NC(=O)Cn2c3ccccc3nc2SCC
C[C@@H](C(=O)c1ccccc1)OC(=O)c2ccc(cc2)N3C(=O)c4cccc(c4C3=O)[N+](=O)[O-]
COc1ccc2ccc3c(c2c1)C=CC(O3)(c4ccccc4)c5ccccc5
COc1ccc(cc1)C2(C=Cc3c4ccccc4ccc3O2)c5ccc(cc5)OC
c1ccc(cc1)C2(C=Cc3c4ccccc4ccc3O2)c5ccccc5
COc1ccc(cc1)C2(C=Cc3ccc4ccccc4c3O2)c5ccc(cc5)OC
CC1(C=Cc2cc(c(cc2O1)OCc3ccc(cc3)[N+](=O)[O-])OCc4ccc(cc4)[N+](=O)[O-])C
c1cc(c(cc1Cl)Cl)Nc2nnc(s2)c3cc4cc(ccc4oc3=O)Cl
```

```
Cc1ccc(cc1)NC(=O)[C@@H](Cc2ccc(cc2)O)NC(=O)c3ccccc3NC(=O)c4ccc(cc4)OC
CCCCCCCOc1ccc(cc1)c2ccc(c(n2)Cl)C#N
CCOC(=O)c1c2cc(ccc2oc1c3ccccc3)NS(=O)(=O)c4ccc(cc4)Br
COc1ccc(cc1)C(=O)Nc2cccc(c2)/C=C/3\COc4ccccc4C3=O
CCCCCCCOc1ccc(cc1)c2nc(no2)CCC
Cc1ccc(cc1)S(=O)(=O)N(c2ccc3c(c2)c(c(o3)C)C(=O)C)C(=O)C4CCCCC4
CCOC(=O)c1c(oc2c1cc(cc2)N(C(=O)C3CCCCC3)S(=O)(=O)c4ccc(cc4)C)C
Cc1c(c2cc(ccc2o1)N(C(=O)C3CCCCC3)S(=O)(=O)c4ccccc4)C(=O)OC
CCc1ccc(cc1)S(=O)(=O)N(c2ccc3c(c2)c(c(o3)C)C(=O)OC)C(=O)C4CCCCC4
Cc1ccc(cc1)S(=O)(=O)N(c2ccc3c(c2)c(c(o3)C)C(=O)OC)C(=O)C4CCCCC4
Cc1ccc(c(c1)C)S(=O)(=O)N(c2ccc3c(c2)c(c(o3)C)C(=O)OC)C(=O)C4CCCCC4
Cc1c(c2cc(ccc2o1)N(C(=O)C3CCCCC3)S(=O)(=O)c4ccc(cc4)Cl)C(=O)OC
Cc1ccc(c(c1)C)S(=O)(=O)N(c2ccc3c(c2)c4c(o3)CCCC4)C(=O)C5CCCCC5
Cc1cc(c(cc1C)S(=O)(=O)N(c2ccc3c(c2)c4c(o3)CCCC4)C(=O)C5CCCCC5)C
CCOc1ccccc1N(CC(=O)Nc2ccccc2C(=O)[O-])S(=O)(=O)c3ccc(cc3)C
CCOc1ccc(cc1)S(=O)(=O)Nc2ccc(c(c2)Sc3nc4ccccc4s3)O
c1ccc(cc1)C2=Cc3ccccc3O[C@@H]2c4ccc(cc4)OCC[NH+]5CCCC5
Cc1ccc(cc1C)NCc2ccccc2OCC=C
COc1ccc(cc1)C2=Cc3ccccc3O[C@@H]2c4ccc(cc4)OCC[NH+]5CCCCC5
CC1(C=Cc2cc(c(cc2O1)[N+](=O)[O-])NC(=O)Cc3ccc(cc3)OC)C
COc1ccc(cc1)c2cn3cc(cc(c3n2)Cl)C(F)(F)F
COc1ccc(cc1)C2(C=Cc3c4ccccc4cc(c3O2)O)c5ccc(cc5)OC
CC(=O)Nc1ccc(cc1)OCc2cc(ccc2OC)/C=C/C(=O)c3ccccc3O
CC1=NC2=C([C@H](C1C(=O)OC3CCCC3)c4ccc(cc4)OCc5ccccc5)C(=O)C[C@H](C2)c6ccccc6
CC1=NC2=C([C@@H](C1C(=O)OC3CCCC3)c4ccc(cc4)OCc5ccccc5)C(=O)C[C@H](C2)c6ccccc6
CC1=NC2=C([C@@H](C1C(=O)OC3CCCC3)c4ccc(cc4)OCc5ccccc5)C(=O)C[C@@H](C2)c6ccccc6
c1ccc(c(c1)C(=O)N2CCN(CC2)c3ccc(cc3S(=O)(=O)N4CCOCC4)[N+](=O)[O-])Cl
CC1=NC2=C([C@H](C1C(=O)OCCOc3ccccc3)c4ccc(c(c4OC)OC)OC)C(=O)CC(C2)(C)C
CC1=NC2=C([C@@H](C1C(=O)OCCOc3ccccc3)c4ccc(c(c4OC)OC)OC)C(=O)CC(C2)(C)C
c1cc(cc(c1)S(=O)(=O)N2CC[NH+](CC2)Cc3ccc4c(c3)OCO4)C(F)(F)F
CC1=NC2=C([C@H](C1C(=O)OC)c3cc(c(c(c3)OC)[O-])[N+](=O)[O-])C(=O)C[C@H](C2)c4cccs4
```

```
CC1=NC2=C([C@@H](C1C(=O)OC)c3cc(c(c(c3)OC)[O-])[N+](=O)[O-])C(=O)C[C@H](C2)c4cccs4
CC1=NC2=C([C@H](C1C(=O)OC)c3cc(c(c(c3)OC)[O-])[N+](=O)[O-])C(=O)C[C@@H](C2)c4cccs4
CC1=NC2=C([C@@H](C1C(=O)OC)c3cc(c(c(c3)OC)[O-])[N+](=O)[O-])C(=O)C[C@@H](C2)c4cccs4
CCCCOC(=O)c1c(oc2c1cc(cc2)[N-]S(=O)(=O)c3ccc4c5c3cccc5C(=O)N4)C
CCCCOC(=O)c1c(oc2c1cc(c3c2cccc3)NS(=O)(=O)c4ccccc4)C
Cc1cccc(c1)C(=O)Nc2ccc(cc2)C(=O)/C=C/c3ccccc3OC
Cc1ccc(cc1)S(=O)(=O)N(CC(=O)Nc2ccccc2C(=O)[O-])c3cccc(c3C)Cl
COc1ccc(cc1)NC(=O)CN(c2ccc(cc2)C34CC5CC(C3)CC(C5)C4)S(=O)(=O)C
CC(=O)N1c2ccccc2NC3=C([C@@H]1c4cccc(c4)Br)C(=O)CC(C3)(C)C
CC(=O)N1c2ccccc2NC3=C([C@H]1c4cccc(c4)Br)C(=O)CC(C3)(C)C
Cc1ccc(cc1)S(=O)(=O)N2[C@@H](CC(=N2)c3ccc(cc3)Br)c4ccc(cc4)Br
Cc1ccc(cc1)S(=O)(=O)N2[C@H](CC(=N2)c3ccc(cc3)Br)c4ccc(cc4)Br
Cc1c(c2cc(ccc2o1)N(C(=O)c3ccncc3)S(=O)(=O)c4ccc(cc4)F)C(=O)C
CC1(C=Cc2cc(ccc2O1)/C(=N/OC(=O)Nc3cccc(c3)C(F)(F)F)/N)C
CC1(C=Cc2cc(ccc2O1)/C(=N/OC(=O)Nc3ccc(cc3)OC)/N)C
CC1=NC2=C([C@@H](C1C(=O)OC)c3cn(nc3c4ccccc4)Cc5ccc(cc5Cl)Cl)C(=O)CCC2
CC1=NC2=C([C@H](C1C(=O)OCCc3ccccc3)c4ccc5c(c4)OCO5)C(=O)C[C@@H](C2)c6ccccc6
c1ccc(cc1)[C@@H]2CC(=C(/C(=N/Cc3ccc4c(c3)OCO4)/C2)C(=S)Nc5ccccc5)[O-]
[H]/N=C(/Cc1[n+](c2ccccc2s1)CCCS(=O)(=O)[O-])\C=C/3\N(c4ccccc4S3)CCCS(=O)(=O)ON
Cc1cccc(c1)NC(=O)/C(=C/c2ccccc2OCc3ccccc3F)/C#N
COc1ccc2c(c1)ccc3c2C=CC(O3)(c4ccccc4)c5ccccc5
COc1cccc(c1OCc2ccccc2F)CNc3ccc(cc3)N4CCOCC4
Cc1cccc(c1)NCc2c3ccccc3ccc2OCc4ccccc4F
CCCCC(CCC(=O)Nc1ccccc1Cl)(C(=O)OCC)C(=O)OCC
Cc1cccc(c1C)NCc2ccc(c(c2)OC)OCc3ccccc3F
c1ccc(c(c1)CNc2ccc(cc2)F)OCc3ccc(cc3)[N+](=O)[O-]
CN1c2ccccc2-c3ccccc3[C@@]14C=Cc5cc(ccc5O4)/N=N/c6ccccc6
CN1c2ccccc2-c3ccccc3[C@]14C=Cc5cc(ccc5O4)/N=N/c6ccccc6
COc1ccc(cc1)C(=O)/C=C/c2ccc(cc2)NC(=O)c3ccc(cc3)F
c1ccc(c(c1)C(=O)Nc2ccc(cc2)C(=O)/C=C/c3ccc4c(c3)OCO4)F
CC(=O)Nc1ccc(cc1)OCc2cc(ccc2OC)/C=C/C(=O)c3ccccc3
```

```
COc1ccc(cc1OC)C[NH2+]C2CCCCC2
Cc1cccc(c1)NC(=O)C2=Cc3ccccc3OC2
Cc1ccc(cc1C)NC(=O)C2=Cc3ccccc3OC2
Cc1cc(cc(c1)NC(=O)C2=Cc3ccccc3OC2)C
Cc1cccc(c1)NC(=O)/C=C/C2=Cc3ccccc3OC2
Cc1ccc(cc1C)NC(=O)/C=C/C2=Cc3ccccc3OC2
c1ccc2c(c1)C=C(CO2)/C=C/C(=O)Nc3ccc(cc3)F
c1ccc2c(c1)C=C(CO2)/C=C/C(=O)Nc3ccccc3F
Cc1cc(cc(c1)NC(=O)/C=C/C2=Cc3ccccc3OC2)C
CCOc1ccccc1/C=C/C(=O)c2ccc(cc2)N3CCOCC3
CC(C)Oc1c(cccc1OC)/C=C/C(=O)c2ccc(cc2)N3CCOCC3
CCN(CC)c1ccc2c(c1)O[C@@]3(C=C2)C(=Cc4ccccc4O3)c5ccccc5
CCN(CC)c1ccc2c(c1)O[C@]3(C=C2)C(=Cc4ccccc4O3)c5ccccc5
Cn1c2c(c(=O)[nH]c1=O)n(c(n2)N3CC[NH+](CC3)C)Cc4ccc(cc4)[N+](=O)[O-]
Cn1c2c(c(=O)[nH]c1=O)n(c(n2)N3CC[NH+](CC3)C)CC(=O)c4ccc(cc4)Br
Cn1c2c(c(=O)[nH]c1=O)n(c(n2)N3CC[NH+](CC3)C)C[C@@H](COc4ccc(cc4)Br)O
Cn1c2c(c(=O)[nH]c1=O)n(c(n2)N3CC[NH+](CC3)C)C[C@H](COc4ccc(cc4)Br)O
COc1ccc2c3c1cccc3O[C@@]24C=Cc5c6ccccc6ccc5O4
COc1ccc2c3c1cccc3O[C@@]24C=Cc5ccccc5O4
COc1ccc2c3c1cccc3O[C@]24C=Cc5c6ccccc6ccc5O4
COc1ccc2c3c1cccc3O[C@]24C=Cc5ccccc5O4
CC1=NC2=C([C@H](C1C(=O)OC)c3cccc(c3)OCc4ccccc4)C(=O)CC(C2)(C)C
CC1=NC2=C([C@@H](C1C(=O)OC)c3cccc(c3)OCc4ccccc4)C(=O)CC(C2)(C)C
Cc1ccc(cc1)N[C@H]2C=C(Oc3c2cccc3)c4ccccc4
Cc1cccc(c1C)NC(=O)CSc2[nH]nc(n2)c3ccccc3O
c1cc(cc(c1)NC(=O)c2cn3cc(cc(c3n2)Cl)C(F)(F)F)C(F)(F)F
c1cc(ccc1NNC(=O)c2cn3cc(cc(c3n2)Cl)Cl)F
c1ccc(cc1)C2=Cc3ccccc3O[C@H]2c4ccc(cc4)O
CC1(C=Cc2c(ccc(c2O1)OC)OC)C
c1ccc2c(c1)C=C([C@@H](O2)c3ccc(cc3)OCC[NH+]4CCCCC4)c5ccc(cc5)O
c1ccc(cc1)COc2ccccc2/C=C/C(=O)c3cccc(c3)[N+](=O)[O-]
```

```
CCCCOC(=O)c1ccc(cc1)NC(=O)/C=C/C2=Cc3ccccc3O[C@H]2C
CC(C)(C)c1ccc(cc1)[C@@H]2C(=C(C(=O)N2CCc3ccccc3)O)C(=O)c4cc5ccccc5o4
CC(C)(C)c1ccc(cc1)[C@H]2C(=C(C(=O)N2CCc3ccccc3)O)C(=O)c4cc5ccccc5o4
CC1(CC2=C([C@@H](c3c(=O)[nH]c(nc3N2)SCc4ccccc4)c5ccc(c(c5)Cl)Cl)C(=O)C1)C
CC1(CC2=C([C@@H](c3c(=O)[nH]c(nc3N2)SCc4ccccc4)c5ccc(cc5)Br)C(=O)C1)C
CC1(CC2=C([C@@H](c3c(=O)[nH]c(nc3N2)SCc4ccccc4Cl)c5ccc(cc5)OCc6ccccc6)C(=O)C1)C
CC1(CC2=C([C@H](c3c(=O)[nH]c(nc3N2)SCc4ccccc4)c5ccc(c(c5)Cl)Cl)C(=O)C1)C
CC1(CC2=C([C@H](c3c(=O)[nH]c(nc3N2)SCc4ccccc4)c5ccc(cc5)Br)C(=O)C1)C
CC1(CC2=C([C@H](c3c(=O)[nH]c(nc3N2)SCc4ccccc4Cl)c5ccc(cc5)OCc6ccccc6)C(=O)C1)C
CCC(C)(C)c1ccc(c(c1)C(C)(C)CC)OCC(=O)Nc2c(nc3n2c(ccc3)C)c4ccccc4
CCCCCOc1ccc(cc1)[C@@H]2C(=C(C(=O)N2Cc3ccco3)[O-])C(=O)c4ccc5c(c4)C[C@@H](O5)C
CCCCCOc1ccc(cc1)[C@@H]2C(=C(C(=O)N2Cc3ccco3)[O-])C(=O)c4ccc5c(c4)C[C@H](O5)C
CCCCCOc1ccc(cc1)[C@H]2C(=C(C(=O)N2Cc3ccco3)[O-])C(=O)c4ccc5c(c4)C[C@@H](O5)C
CCCCCOc1ccc(cc1)[C@H]2C(=C(C(=O)N2Cc3ccco3)[O-])C(=O)c4ccc5c(c4)C[C@H](O5)C
CCCCCOc1ccc(cc1OC)[C@@H]2C(=C(C(=O)N2Cc3ccco3)[O-])C(=O)c4ccc(cc4)F
CCCCCOc1ccc(cc1OC)[C@H]2C(=C(C(=O)N2Cc3ccco3)[O-])C(=O)c4ccc(cc4)F
CCCCOc1ccc(cc1)C(=O)C2=C(C(=O)N([C@@H]2c3ccc(c(c3)OC)OC)c4nc(c(s4)C(=O)OC)C)O
CCCCOc1ccc(cc1)C(=O)C2=C(C(=O)N([C@@H]2c3ccc(cc3)F)c4nnc(s4)C)[O-]
CCCCOc1ccc(cc1)C(=O)C2=C(C(=O)N([C@H]2c3ccc(c(c3)OC)OC)c4nc(c(s4)C(=O)OC)C)O
CCCCOc1ccc(cc1)C(=O)C2=C(C(=O)N([C@H]2c3ccc(cc3)F)c4nnc(s4)C)[O-]
CCCCOc1ccc(cc1OC)[C@@H]2C(=C(C(=O)N2Cc3ccco3)O)C(=O)c4ccc(cc4)Br
CCCCOc1ccc(cc1OC)[C@@H]2C(=C(C(=O)N2Cc3ccco3)[O-])C(=O)c4ccc5c(c4)C[C@@H](O5)C
CCCCOc1ccc(cc1OC)[C@@H]2C(=C(C(=O)N2Cc3ccco3)[O-])C(=O)c4ccc5c(c4)C[C@H](O5)C
CCCCOc1ccc(cc1OC)[C@H]2C(=C(C(=O)N2Cc3ccco3)O)C(=O)c4ccc(cc4)Br
CCCCOc1ccc(cc1OC)[C@H]2C(=C(C(=O)N2Cc3ccco3)[O-])C(=O)c4ccc5c(c4)C[C@@H](O5)C
CCCCOc1ccc(cc1OC)[C@H]2C(=C(C(=O)N2Cc3ccco3)[O-])C(=O)c4ccc5c(c4)C[C@H](O5)C
CCCCOc1cccc(c1)C(=O)C2=C(C(=O)N([C@@H]2c3cccc(c3)O)Cc4ccncc4)[O-]
CCCCOc1cccc(c1)C(=O)C2=C(C(=O)N([C@H]2c3cccc(c3)O)Cc4ccncc4)[O-]
CCCOc1ccc(cc1)[C@@H]2C(=C(C(=O)N2Cc3cccnc3)[O-])C(=O)c4ccc(c(c4)OC)OC
CCCOc1ccc(cc1)[C@H]2C(=C(C(=O)N2Cc3cccnc3)[O-])C(=O)c4ccc(c(c4)OC)OC
CCOCCCN1[C@@H](C(=C(C1=O)[O-])C(=O)c2ccc(c(c2)C)OCC)c3ccc(cc3)C
```

```
CCOCCCN1[C@H](C(=C(C1=O)[O-])C(=O)c2ccc(c(c2)C)OCC)c3ccc(cc3)C
CCOc1ccc(cc1)C(=O)C2=C(C(=O)N([C@@H]2c3ccc(c(c3)OC)OC)Cc4ccco4)[O-]
CCOc1ccc(cc1)C(=O)C2=C(C(=O)N([C@@H]2c3ccc(c(c3)OC)OCCC(C)C)Cc4ccco4)[O-]
CCOc1ccc(cc1)C(=O)C2=C(C(=O)N([C@H]2c3ccc(c(c3)Cl)Cl)Cc4ccco4)[O-]
CCc1ccc(cc1)[C@@H]2C(=C(C(=O)N2c3nc4ccc(cc4s3)C)[O-])C(=O)c5ccc(cc5)C
CCc1ccc(cc1)[C@H]2C(=C(C(=O)N2c3nc4ccc(cc4s3)C)[O-])C(=O)c5ccc(cc5)C
CC(C)(C)c1ccc(cc1)C(=O)Nc2c(c3c(s2)CCCC3)C(=O)NCc4cccnc4
CCCCCCC(=O)N[C@@H](C(Cl)(Cl)Cl)NC(=S)Nc1cccc(c1)C(F)(F)F
CCCCCCC(=O)N[C@H](C(Cl)(Cl)Cl)NC(=S)Nc1cccc(c1)C(F)(F)F
CCCCCCOc1ccc(cc1)C(=O)Nc2c(c3c(s2)CCCC3)C(=O)NCc4cccnc4
CC(=O)Nc1ccc(cc1)OCc2cc(ccc2OC)/C=C/C(=O)c3ccc(cc3)OC
CC(C)OC(=O)CSc1c(c(cc(n1)c2ccccc2)c3ccc(cc3)Cl)C#N
c1ccc(cc1)C2=Cc3ccccc3O[C@H]2c4ccc(cc4)OCC[NH+]5CCCC5
CCn1c2ccccc2c3c1ccc(c3)NC(=O)c4ccccc4
CN(C)c1ccc(cc1)[C@@]2(C=Cc3c4ccc(cc4ccc3O2)OC)c5ccc(c6c5cccc6)N(C)C
CN(C)c1ccc(cc1)[C@]2(C=Cc3c4ccc(cc4ccc3O2)OC)c5ccc(c6c5cccc6)N(C)C
CC1(Cc2c(c3cc(ccc3o2)NS(=O)(=O)c4ccc5c(c4)C(=O)c6ccccc6C5=O)C(=O)C1)C
C[C@@H]1CCc2c(c3cc(ccc3o2)NS(=O)(=O)c4ccc5c(c4)C(=O)c6ccccc6C5=O)C1
C[C@H]1CCc2c(c3cc(ccc3o2)NS(=O)(=O)c4ccc5c(c4)C(=O)c6ccccc6C5=O)C1
Cn1c(=O)c2c3c(sc2nc1SCC(=O)Nc4ccc(cc4)S(=O)(=O)Nc5cc(nc(n5)OC)OC)CCCC3
c1ccc(cc1)N2[C@@H]3CS(=O)(=O)C[C@@H]3N(C2=S)c4ccc(cc4)Br
c1ccc(cc1)N2[C@@H]3CS(=O)(=O)C[C@H]3N(C2=S)c4ccc(cc4)Br
CC1=NC2=C([C@@H](C1C(=O)OC)c3ccc(cc3)OCc4ccccc4)C(=O)C[C@@H](C2)c5ccc(cc5)Cl
CC(C)(C)c1ccc(cc1)OC[C@@H](C[NH+]2CCc3ccccc3C2)O
CC(C)(C)c1ccc(cc1)OC[C@H](C[NH+]2CCc3ccccc3C2)O
Cc1c(sc(c1C#N)NC(=O)CSc2c(c(cc(n2)c3ccccc3)c4ccc(cc4)OC)C#N)C
Cc1cc(cc(c1)NC(=O)CSc2c(c(cc(n2)c3ccc(cc3)OC)c4ccc(cc4)OC)C#N)C
Cc1cc(cc(c1)NC(=O)CSc2c(c(cc(n2)c3ccc(cc3)OC)c4ccccc4)C#N)C
Cc1cc(cc(c1)NC(=O)CSc2c(c(cc(n2)c3ccccc3)c4ccc(cc4)OC)C#N)C
Cc1cc(cc2c1N[C@@H]([C@@H]3[C@@H]2[C@@H]([C@@H](C3)Sc4ccccc4[N+](=O)[O-])Cl)c5ccc(cc5)Br)[N+](=O)[O-]
Cc1cc(cc2c1N[C@@H]([C@H]3[C@@H]2[C@@H]([C@@H](C3)Sc4ccccc4[N+](=O)[O-])Cl)c5ccc(cc5)Br)[N+](=O)[O-]
```

```
Cc1cc(cc2c1N[C@H]([C@@H]3[C@@H]2[C@@H]([C@@H](C3)Sc4ccccc4[N+](=O)[O-])Cl)c5ccc(cc5)Br)[N+](=O)[O-]
Cc1cc(cc2c1N[C@H]([C@H]3[C@@H]2[C@@H]([C@@H](C3)Sc4ccccc4[N+](=O)[O-])Cl)c5ccc(cc5)Br)[N+](=O)[O-]
Cc1cc(ccc1NC(=S)NC(=O)c2ccc(cc2)I)[N+](=O)[O-]
Cc1cc2c3c(c1)[C@@H]4[C@@H](C[C@@H]([C@@H]4Cl)Sc5ccccc5[N+](=O)[O-])[C@@H](N3C[C@@H]6[C@@H]2C=CC6)C(=O)[O-]
Cc1cc2c3c(c1)[C@@H]4[C@H](C[C@@H]([C@@H]4Cl)Sc5ccccc5[N+](=O)[O-])[C@@H](N3C[C@@H]6[C@@H]2C=CC6)C(=O)[O-]
Cc1ccc(c(c1)C)NC(=O)c2ccc(cc2)Nc3nc4ccc(cc4c(n3)c5ccccc5)Br
COc1ccc2c(c1)C=C[C@H](O2)c3ccc(cc3)[N+](=O)[O-]
Cc1ccc(cc1)S(=O)(=O)N(CC(=O)NCCSc2ccccn2)c3cccc(c3)Cl
Cc1ccc(cc1)c2cc(c(c(n2)SCC(=O)N(C3CCCCC3)C4CCCCC4)C#N)c5ccccc5
Cc1ccc(cc1)c2cc(c(c(n2)SCC(=O)Nc3cccc(c3)C)C#N)c4ccc(cc4)OC
Cc1ccc(cc1)c2cc(c3c(c(sc3n2)C(=O)Nc4cc(ccc4C)C)N)c5ccc(c(c5)OC)OC
c1cc(sc1)S(=O)(=O)[N-]c2ccc(c(c2)Sc3[nH]ncn3)O
c1ccc(c(c1)C(=O)Nc2ccc(cc2)Cl)[N-]S(=O)(=O)c3cccc4c3nccc4
CCc1cccc(c1N(COCC)C(=O)Cn2c3ccccc3nc2COc4ccc(cc4)C)C
C[C@@H](C(=O)NC(c1ccccc1)c2ccccc2)Sc3nnc(n3C)C(c4ccccc4)c5ccccc5
C[C@H](C(=O)NC(c1ccccc1)c2ccccc2)Sc3nnc(n3C)C(c4ccccc4)c5ccccc5
Cc1c(cc(s1)[C@@H]2C3=C(CC(CC3=O)(C)C)NC(=C2C(=O)OC)C)COc4ccc(cc4)F
Cc1c(cc(s1)[C@H]2C3=C(CC(CC3=O)(C)C)NC(=C2C(=O)OC)C)COc4ccc(cc4)F
Cc1cc(c(c(c1)C)COc2ccc(cc2OC)[C@@H]3c4c(n[nH]c4OC(=C3C#N)N)C)C
Cc1cc(c(c(c1)C)COc2ccc(cc2OC)[C@H]3c4c(n[nH]c4OC(=C3C#N)N)C)C
C=CCOc1ccccc1CNc2ccc(cc2)F
Cc1ccc(c(c1)C)NC(=O)CSC2=NC3=C([C@H](C2C#N)c4cccs4)C(=O)CC(C3)(C)C
Cc1ccc(c(c1)C)NC(=O)CSC2=NC3=C([C@@H](C2C#N)c4cccs4)C(=O)CC(C3)(C)C
c1ccc(cc1)COc2ccccc2/C=C/C(=O)c3ccc(cc3)F
Cc1ccc(cc1)C(=O)C2=C(C(=O)N([C@H]2c3ccc(cc3)Br)c4ccc(cc4)O)[O-]
Cc1ccc(cc1)C(=O)C2=C(C(=O)N([C@H]2c3ccc(cc3)Cl)c4ccc(cc4)O)[O-]
Cc1ccc(cc1)C(=O)C2=C(C(=O)N([C@H]2c3ccc(cc3)OC)c4cccc(c4)C(F)(F)F)[O-]
Cc1ccc(cc1)C(=O)C2=C(C(=O)N([C@@H]2c3ccc(cc3)Br)c4ccc(cc4)O)[O-]
Cc1ccc(cc1)C(=O)C2=C(C(=O)N([C@@H]2c3ccc(cc3)Cl)c4ccc(cc4)O)[O-]
Cc1ccc(cc1)C(=O)C2=C(C(=O)N([C@@H]2c3ccc(cc3)OC)c4cccc(c4)C(F)(F)F)[O-]
CCCCc1ccc(cc1)NCc2ccc(cc2)OCc3ccccc3F
```

```
Cc1cccc(c1)NC(=O)CSc2c(c(cc(n2)c3ccccc3)c4ccc(c(c4)OC)OC)C#N
Cc1cccc(c1C)NC[C@@H](Cn2c3ccc(cc3c4c2ccc(c4)I)I)O
Cc1cccc(c1C)NC[C@H](Cn2c3ccc(cc3c4c2ccc(c4)I)I)O
Cc1cccc(c1NC(=O)[C@@H](C)Sc2c(c(cc(n2)c3ccccc3)c4ccc(cc4)C(C)(C)C)C#N)C
Cc1cccc(c1NC(=O)[C@H](C)Sc2c(c(cc(n2)c3ccccc3)c4ccc(cc4)C(C)(C)C)C#N)C
Cc1cccc(c1NC(=O)c2ccc(cc2)Nc3nc4ccc(cc4c(n3)c5ccccc5)Br)C
Cc1ccccc1NC(=O)c2c(nc(c(c2c3cccnc3)C#N)SCC(=O)c4ccc(cc4)Cl)C
Cc1ccccc1NC(=O)C2=C(NC(=C([C@@H]2c3ccc(cc3)OC)C#N)SCC(=O)c4cccs4)C
Cc1ccccc1NC(=O)C2=C(NC(=C([C@@H]2c3cccs3)C#N)SCC(=O)c4ccc(cc4)C5CCCCC5)C
Cn1c2ccccc2c(c1c3ccccc3)/N=N\c4nc5ccc(cc5n4C)Br
c1cc(ccc1NC(=O)c2cc(ccc2I)I)N3CCOCC3
c1cc(ccc1NC[C@@H](Cn2c3ccc(cc3c4c2ccc(c4)Cl)Cl)O)I
c1cc(ccc1NC[C@H](Cn2c3ccc(cc3c4c2ccc(c4)Cl)Cl)O)I
c1cc(cnc1)NC(=O)c2cc(ccc2I)I
c1cc2c(c(c1)I)N[C@@H]([C@@H]3[C@@H]2C=CC3)c4ccc(cc4Cl)Cl
c1cc2c(c(c1)I)N[C@H]([C@@H]3[C@@H]2C=CC3)c4ccc(cc4Cl)Cl
c1cc2c(c(c1)I)N[C@H]([C@H]3[C@@H]2C=CC3)c4ccc(cc4Cl)Cl
c1ccc(c(c1)[C@@H]2[C@@H]3C[C@@H]([C@@H]([C@@H]3c4cccc(c4N2)C(=O)[O-])Cl)Sc5ccccc5[N+](=O)[O-])[N+](=O)[O-]
c1ccc(c(c1)[C@@H]2[C@H]3C[C@@H]([C@@H]([C@@H]3c4cccc(c4N2)C(=O)[O-])Cl)Sc5ccccc5[N+](=O)[O-])[N+](=O)[O-]
c1ccc(c(c1)[C@H]2[C@@H]3C[C@@H]([C@@H]([C@@H]3c4cccc(c4N2)C(=O)[O-])Cl)Sc5ccccc5[N+](=O)[O-])[N+](=O)[O-]
c1ccc(c(c1)[C@H]2[C@H]3C[C@@H]([C@@H]([C@@H]3c4cccc(c4N2)C(=O)[O-])Cl)Sc5ccccc5[N+](=O)[O-])[N+](=O)[O-]
c1ccc(c(c1)[N+](=O)[O-])S[C@@H]2C[C@@H]3CNc4c(ccc(c4[C@@H]3[C@@H]2Cl)[N+](=O)[O-])C(=O)[O-]
c1ccc(c(c1)[N+](=O)[O-])S[C@@H]2C[C@@H]3[C@@H](Nc4c(cccc4C(F)(F)F)[C@@H]3[C@@H]2Cl)c5cccc(c5)[N+](=O)[O-]
c1cc(ccc1c2cnc([nH]2)SCC(=O)N(C3CCCCC3)C4CCCCC4)Cl
c1cc(ccc1c2cnc([nH]2)SCC(=O)Nc3c(c4c(s3)CCCC4)C#N)Cl
c1ccc(c(c1)[N+](=O)[O-])S[C@@H]2C[C@@H]3[C@@H]([C@@H]2Cl)c4cccc(c4N[C@@H]3C(=O)[O-])[N+](=O)[O-]
c1ccc(c(c1)[N+](=O)[O-])S[C@@H]2C[C@@H]3[C@@H]([C@@H]2Cl)c4cccc(c4N[C@@H]3c5ccc(cc5Cl)Cl)C(=O)[O-]
c1ccc(c(c1)[N+](=O)[O-])S[C@@H]2C[C@H]3CNc4c(ccc(c4[C@@H]3[C@@H]2Cl)[N+](=O)[O-])C(=O)[O-]
c1ccc(c(c1)[N+](=O)[O-])S[C@@H]2C[C@H]3[C@@H](Nc4c(cccc4C(F)(F)F)[C@@H]3[C@@H]2Cl)c5cccc(c5)[N+](=O)[O-]
c1ccc(c(c1)[N+](=O)[O-])S[C@@H]2C[C@H]3[C@@H]([C@@H]2Cl)c4cccc(c4N[C@@H]3C(=O)[O-])[N+](=O)[O-]
c1ccc(c(c1)[N+](=O)[O-])S[C@@H]2C[C@H]3[C@@H]([C@@H]2Cl)c4cccc(c4N[C@@H]3c5ccc(cc5Cl)Cl)C(=O)[O-]
```

```
c1ccc(c(c1)[N+](=O)[O-])S[C@H]2C[C@@H]3CNc4c(ccc(c4[C@@H]3[C@@H]2Cl)[N+](=O)[O-])C(=O)[O-]
c1ccc(c(c1)[N+](=O)[O-])S[C@H]2C[C@@H]3[C@@H](Nc4c(cccc4C(F)(F)F)[C@@H]3[C@@H]2Cl)c5cccc(c5)[N+](=O)[O-]
c1ccc(c(c1)[N+](=O)[O-])S[C@H]2C[C@@H]3[C@@H]([C@@H]2Cl)c4cccc(c4N[C@@H]3C(=O)[O-])[N+](=O)[O-]
c1ccc(c(c1)[N+](=O)[O-])S[C@H]2C[C@@H]3[C@@H]([C@@H]2Cl)c4cccc(c4N[C@@H]3c5ccc(cc5Cl)Cl)C(=O)[O-]
c1ccc(c(c1)[N+](=O)[O-])S[C@H]2C[C@H]3CNc4c(ccc(c4[C@@H]3[C@@H]2Cl)[N+](=O)[O-])C(=O)[O-]
c1ccc(c(c1)[N+](=O)[O-])S[C@H]2C[C@H]3[C@@H](Nc4c(cccc4C(F)(F)F)[C@@H]3[C@@H]2Cl)c5cccc(c5)[N+](=O)[O-]
c1ccc(c(c1)[N+](=O)[O-])S[C@H]2C[C@H]3[C@@H]([C@@H]2Cl)c4cccc(c4N[C@@H]3C(=O)[O-])[N+](=O)[O-]
c1ccc(c(c1)[N+](=O)[O-])S[C@H]2C[C@H]3[C@@H]([C@@H]2Cl)c4cccc(c4N[C@@H]3c5ccc(cc5Cl)Cl)C(=O)[O-]
c1ccc(cc1)C(=O)C2=C(C(=O)N([C@H]2c3ccccn3)CCc4c[nH]c5c4cccc5)[O-]
c1ccc(cc1)C(=O)C2=C(C(=O)N([C@H]2c3cccnc3)CCc4c[nH]c5c4cccc5)[O-]
c1ccc(cc1)C(=O)C2=C(C(=O)N([C@@H]2c3cccnc3)CCc4c[nH]c5c4cccc5)[O-]
c1ccc(cc1)N2[C@@H](c3c(n[nH]c3C2=O)c4ccc(cc4)Br)c5ccc(cc5)Br
c1ccc(cc1)N2[C@H](c3c(n[nH]c3C2=O)c4ccc(cc4)Br)c5ccc(cc5)Br
c1ccc(cc1)c2c3c([nH]n2)C(=O)N([C@@H]3c4ccc(cc4)Br)c5cccc(c5)C(F)(F)F
c1ccc(cc1)c2c3c([nH]n2)C(=O)N([C@H]3c4ccc(cc4)Br)c5cccc(c5)C(F)(F)F
c1ccc(cc1)c2c3cc(ccc3[nH]c(=O)c2C(=O)Nc4ccc(cc4C(=O)c5ccccc5)Br)Br
c1ccc2c(c1)[nH]c(n2)/C(=C/c3cc(cc(c3OCc4ccc(cc4Cl)Cl)Br)Br)/C#N
c1ccc2c(c1)[nH]c(n2)SCCCCCCCCCCSc3[nH]c4ccccc4n3
c1ccc(cc1)c2c3cc(ccc3nc(n2)Nc4ccc(cc4)C(=O)N5CCCCC5)Br
c1ccc(cc1)c2cc(nc(c2C#N)SCC(=O)NC3CCCCC3)c4ccccc4
c1ccc(cc1)c2cc(nc3c2c(c(s3)C(=O)Nc4cccc5c4cccc5)N)c6ccccc6
c1ccc2c(c1)C(=O)c3ccc(cc3C2=O)NC(=S)NC(=O)c4ccc(cc4)[N+](=O)[O-]
c1ccc2cc(ccc2c1)C3=NN4[C@@H](C3)c5cc(ccc5O[C@@H]4c6ccncc6)Br
c1ccc2cc(ccc2c1)C3=NN4[C@H](C3)c5cc(ccc5O[C@@H]4c6ccncc6)Br
Cc1cc(cc2c1OCCO2)NC(=O)c3ccccc3F
CC(C)Oc1ccccc1/C=C/C(=O)c2ccc(cc2)N3CCOCC3
Cc1ccc(cc1)NC(=O)CSc2[nH]nc(n2)c3ccccc3O
CCCCOC(=O)c1ccc(cc1)NC(=O)/C=C/C2=Cc3ccccc3O[C@@H]2C
Cc1ccc(cc1)C(=O)Nc2cccc(c2)/C=C/C(=O)c3ccc4c(c3)OCO4
CCOC(=O)c1ccc(cc1)NC(=O)/C=C/C2=Cc3ccccc3O[C@@H]2C
CCOc1ccc(cc1)NC(=O)/C=C/C2=Cc3ccccc3O[C@@H]2C
```

```
CCOc1ccc(cc1)NC(=O)/C=C/C2=Cc3ccccc3O[C@H]2C
C[C@@H]1C(=Cc2ccccc2O1)/C=C/C(=O)Nc3ccc(c(c3)Cl)OC
C[C@@H]1C(=Cc2ccccc2O1)/C=C/C(=O)Nc3ccc(cc3)C(=O)C
C[C@@H]1C(=Cc2ccccc2O1)/C=C/C(=O)Nc3ccc(cc3)C(=O)[O-]
C[C@@H]1C(=Cc2ccccc2O1)/C=C/C(=O)Nc3ccc(cc3)C(=O)OC
C[C@@H]1C(=Cc2ccccc2O1)/C=C/C(=O)Nc3ccc(cc3)F
C[C@@H]1C(=Cc2ccccc2O1)/C=C/C(=O)Nc3ccc(cc3)OC
C[C@@H]1C(=Cc2ccccc2O1)/C=C/C(=O)Nc3cccc(c3)C(=O)C
C[C@@H]1C(=Cc2ccccc2O1)/C=C/C(=O)Nc3cccc(c3)C(=O)[O-]
C[C@@H]1C(=Cc2ccccc2O1)/C=C/C(=O)Nc3ccccc3
C[C@@H]1C(=Cc2ccccc2O1)/C=C/C(=O)Nc3ccccc3F
C[C@H]1C(=Cc2ccccc2O1)/C=C/C(=O)Nc3ccc(c(c3)Cl)OC
C[C@H]1C(=Cc2ccccc2O1)/C=C/C(=O)Nc3ccc(cc3)C(=O)C
C[C@H]1C(=Cc2ccccc2O1)/C=C/C(=O)Nc3ccc(cc3)C(=O)[O-]
C[C@H]1C(=Cc2ccccc2O1)/C=C/C(=O)Nc3ccc(cc3)C(=O)OC
C[C@H]1C(=Cc2ccccc2O1)/C=C/C(=O)Nc3ccc(cc3)F
C[C@H]1C(=Cc2ccccc2O1)/C=C/C(=O)Nc3ccc(cc3)OC
C[C@H]1C(=Cc2ccccc2O1)/C=C/C(=O)Nc3cccc(c3)C(=O)C
C[C@H]1C(=Cc2ccccc2O1)/C=C/C(=O)Nc3cccc(c3)C(=O)[O-]
C[C@H]1C(=Cc2ccccc2O1)/C=C/C(=O)Nc3ccccc3
C[C@H]1C(=Cc2ccccc2O1)/C=C/C(=O)Nc3ccccc3F
Cc1cc(ccc1NC(=O)/C=C/C2=Cc3ccccc3O[C@@H]2C)[N+](=O)[O-]
Cc1cc(ccc1NC(=O)/C=C/C2=Cc3ccccc3O[C@H]2C)[N+](=O)[O-]
Cc1ccccc1NC(=O)/C=C/C2=Cc3ccccc3O[C@@H]2C
Cc1ccccc1NC(=O)/C=C/C2=Cc3ccccc3O[C@H]2C
CC(C)c1ccc(cc1[N+](=O)[O-])/C=C/2\[C@@H](Oc3ccccc3C2=O)c4ccccc4
CC(C)c1ccc(cc1[N+](=O)[O-])/C=C/2\[C@H](Oc3ccccc3C2=O)c4ccccc4
c1ccc(c(c1)C(=O)Nc2cccc(c2)C#N)OCc3ccc(cc3)F
c1ccc(cc1)COc2ccc(cc2)NC(=O)c3ccc(cc3F)F
c1ccc(cc1)[C@@H]2/C(=C\c3cccc(c3)[N+](=O)[O-])/C(=O)c4ccccc4O2
c1ccc(cc1)[C@H]2/C(=C\c3cccc(c3)[N+](=O)[O-])/C(=O)c4ccccc4O2
```

```
COc1ccc(cc1)[C@H]2N3[C@@H](CC(=N3)c4ccc(cc4)F)c5cc(ccc5O2)Br
COc1ccc(cc1)[C@]2(C=Cc3c(ccc4c3c5ccccc5o4)O2)c6ccccc6F
COc1ccc(cc1F)C(=O)/C=C/c2cccc(c2)NC(=O)c3ccccc3Cl
CC1(Cc2cccc(c2O1)OCc3ccc(cc3)C(=O)Nc4ccccc4F)C
CC1(Cc2cccc(c2O1)OCc3ccc(cc3)C(=O)Nc4cccc(c4)F)C
CC[NH+]1CCN(CC1)c2ccc(cc2F)C(=O)/C=C/c3ccccc3OCC
COc1cccc(c1OC)/C=C/C(=O)c2ccc(c(c2)F)N3CCOCC3
CCOc1ccccc1/C=C/C(=O)c2ccc(c(c2)F)N3CCOCC3
COc1ccccc1/C=C/C(=O)c2ccc(c(c2)F)N3CCOCC3
CCOc1cccc(c1OC)/C=C/C(=O)c2ccc(c(c2)F)N3CCOCC3
CC(C)Oc1c(cccc1OC)/C=C/C(=O)c2ccc(c(c2)F)N3CCOCC3
CC(C)Oc1c(cccc1OC)/C=C/C(=O)c2ccc(cc2)N(C)C
C=CCOc1ccccc1/C=C/C(=O)c2ccc(cc2)N3CCOCC3
CCOc1ccccc1/C=C/C(=O)c2cccc(c2)NC(=O)C
CCOc1cccc(c1OC)/C=C/C(=O)c2cccc(c2)NC(=O)C
CCCOc1c(cccc1OCC)/C=C/C(=O)c2cccc(c2)NC(=O)C
COc1ccc(cc1)C(=O)Nc2cccc(c2)/C=C/C(=O)c3ccc4c(c3)OCCO4
CCOc1ccc(cc1OC)NC(=O)c2ccccc2OCc3ccc(cc3)F
c1cc(cc(c1)NC(=O)c2cccc(c2)OCc3ccc(cc3)F)C#N
Cc1ccc(cc1C)OCCSc2nc3ccccc3n2CCOc4ccccc4
CCOc1cc(ccc1OC)[C@@H]2C(=C(N=c3n2c(=O)/c(=C/c4ccco4)/s3)C)C(=O)OCC
CCOc1cc(ccc1OC)[C@H]2C(=C(N=c3n2c(=O)/c(=C/c4ccco4)/s3)C)C(=O)OCC
CCOc1cc(ccc1OC)[C@@H]2C(=C(N=c3n2c(=O)/c(=C/c4ccc(c(c4)Br)O)/s3)C)C(=O)OCC
CCOc1cc(ccc1OC)[C@H]2C(=C(N=c3n2c(=O)/c(=C/c4ccc(c(c4)Br)O)/s3)C)C(=O)OCC
c1ccc(cc1)C(=O)C2=C(C(=O)N([C@H]2c3cccc(c3)F)CCc4c[nH]c5c4cccc5)[O-]
COc1ccc(cc1OC)[C@@H]2C(=C(C(=O)N2CCc3c[nH]c4c3cccc4)[O-])C(=O)c5ccccc5
COc1ccc(cc1OC)[C@H]2C(=C(C(=O)N2CCc3c[nH]c4c3cccc4)[O-])C(=O)c5ccccc5
CCOc1cc(ccc1OC)[C@@H]2C(=C(N=c3n2c(=O)/c(=C/c4ccc(cc4)N(C)C)/s3)C)C(=O)OCC
C[C@@]1(CC(=O)[C@@H]([C@@H]([C@@H]1C(=O)OCc2ccccc2)c3ccccc3)C(=O)OCc4ccccc4)O
CCOc1cc(ccc1OC)[C@H]2C(=C(N=c3n2c(=O)/c(=C/c4ccc(cc4)N(C)C)/s3)C)C(=O)OCC
C[C@]1(CC(=O)[C@@H]([C@@H]([C@@H]1C(=O)OCc2ccccc2)c3ccccc3)C(=O)OCc4ccccc4)O
```

CCOc1cc(ccc1OC)[C@@H]2C(=C(N=c3n2c(=O)/c(=C/c4ccc(cc4)C)/s3)C)C(=O)OCC
C[C@@]1(CC(=O)[C@H]([C@@H]([C@@H]1C(=O)OCc2ccccc2)c3ccccc3)C(=O)OCc4ccccc4)O
C[C@]1(CC(=O)[C@H]([C@@H]([C@@H]1C(=O)OCc2ccccc2)c3ccccc3)C(=O)OCc4ccccc4)O
CCOc1cc(ccc1OC)[C@H]2C(=C(N=c3n2c(=O)/c(=C/c4ccc(cc4)C)/s3)C)C(=O)OCC
Cc1ccc(cc1)[C@@H]2[C@@H](C(=O)C[C@@]([C@@H]2C(=O)OCc3ccccc3)(C)O)C(=O)OCc4ccccc4
CCOc1cc(ccc1OC)[C@H]2C(=C(N=c3n2c(=O)/c(=C/c4cn(c5c4cccc5)CC=C)/s3)C)C(=O)OCC
C[C@@]1(CC(=O)[C@H]([C@@H]([C@@H]1C(=O)OCc2ccccc2)c3ccc(cc3)O)C(=O)OCc4ccccc4)O
CCOc1cc(ccc1OC)[C@@H]2C(=C(N=c3n2c(=O)/c(=C/c4ccc5c(c4)OCCO5)/s3)C)C(=O)OCC
C[C@]1(CC(=O)[C@H]([C@@H]([C@@H]1C(=O)OCc2ccccc2)c3ccc(cc3)O)C(=O)OCc4ccccc4)O
CCOc1cc(ccc1OC)[C@H]2C(=C(N=c3n2c(=O)/c(=C/c4ccc5c(c4)OCCO5)/s3)C)C(=O)OCC
CCCCCCCCN1[C@@H](C(=C(C1=O)O)C(=O)C)c2ccc(c(c2)OC)O
CCOc1cc(ccc1OC)[C@@H]2C(=C(N=c3n2c(=O)/c(=C/c4ccc(c(c4)OC)C)/s3)C)C(=O)OCC
CCCCCCCCN1[C@H](C(=C(C1=O)O)C(=O)C)c2ccc(c(c2)OC)O
CCOc1cc(ccc1OC)[C@H]2C(=C(N=c3n2c(=O)/c(=C/c4ccc(c(c4)OC)C)/s3)C)C(=O)OCC
CCCCCCCCN1[C@@H](C(=C(C1=O)O)C(=O)C)c2ccccn2
CCOc1cc(ccc1OC)[C@@H]2C(=C(N=c3n2c(=O)/c(=C/c4cc(ccc4OC)Br)/s3)C)C(=O)OCC
CCOc1cc(ccc1OC)[C@H]2C(=C(N=c3n2c(=O)/c(=C/c4cc(ccc4OC)Br)/s3)C)C(=O)OCC
CCCCCCCCN1[C@H](C(=C(C1=O)O)C(=O)C)c2ccccn2
CCOc1cc(ccc1OC)[C@@H]2C(=C(N=c3n2c(=O)/c(=C/c4ccc(c(c4)Br)OC)/s3)C)C(=O)OCC
CCCCCCCCN1[C@@H](C(=C(C1=O)O)C(=O)C)c2ccc(cc2)O
CCOc1cc(ccc1OC)[C@H]2C(=C(N=c3n2c(=O)/c(=C/c4ccc(c(c4)Br)OC)/s3)C)C(=O)OCC
CCCCCCCCN1[C@H](C(=C(C1=O)O)C(=O)C)c2ccc(cc2)O
CCOc1cc(ccc1OC)[C@@H]2C(=C(N=c3n2c(=O)/c(=C/c4ccc(c(c4)OC)O)/s3)C)C(=O)OCC
CCCCCCCCN1[C@@H](C(=C(C1=O)O)C(=O)C)c2cccnc2
CCOc1cc(ccc1OC)[C@H]2C(=C(N=c3n2c(=O)/c(=C/c4ccc(c(c4)OC)O)/s3)C)C(=O)OCC
CCCCCCCCN1[C@H](C(=C(C1=O)O)C(=O)C)c2cccnc2
CCOc1cc(ccc1OC)[C@@H]2C(=C(N=c3n2c(=O)/c(=C\c4cccc(c4)OC)/s3)C)C(=O)OCC
CCCCCCCCN1[C@@H](C(=C(C1=O)O)C(=O)C)c2cccc(c2)O
CCOc1cc(ccc1OC)[C@H]2C(=C(N=c3n2c(=O)/c(=C\c4cccc(c4)OC)/s3)C)C(=O)OCC
CCCCCCCCN1[C@H](C(=C(C1=O)O)C(=O)C)c2cccc(c2)O
CCCCCCCCN1[C@@H](C(=C(C1=O)O)C(=O)c2ccccc2)c3cccnc3

```
CCCCCCCCN1[C@H](C(=C(C1=O)O)C(=O)c2ccccc2)c3cccnc3
[H]/N=C\1/N(C(=O)/C(=C/c2ccc(o2)c3cccc(c3)F)/S1)c4ccc(cc4Cl)Cl
COc1cc(cc(c1O)CC=C)/C=C/2\C(=O)N(/C(=N\c3ccccc3)/S2)C4CCCCC4
CC(=O)OC([C@@]12C=C[C@@H](O1)[C@@H]3[C@@H]2C(=O)N(C3=O)c4ccccc4C(=O)OC)OC(=O)C
CC(=O)OC([C@]12C=C[C@H](O1)[C@@H]3[C@@H]2C(=O)N(C3=O)c4ccccc4C(=O)OC)OC(=O)C
Cc1ccc(cc1)C(=O)C2=C(C(=O)N([C@H]2c3cccnc3)CCc4ccc(c(c4)OC)OC)[O-]
Cc1cc(c(n1c2cccnc2)C)/C=C\3/C(=O)N(/C(=N\c4ccc(cc4)C(=O)OC)/S3)C
COc1ccc(cc1)C(=O)C2=C(C(=O)N([C@@H]2c3ccccc3OC)CCc4c[nH]c5c4cccc5)[O-]
COc1ccc(cc1)C(=O)C2=C(C(=O)N([C@H]2c3ccccc3OC)CCc4c[nH]c5c4cccc5)[O-]
Cc1ccc(cc1)N2C(=O)/C(=C\c3cccn3c4ccc(cc4)S(=O)(=O)N)/C(=O)N2
CC\1=NN(C(=O)/C1=C\c2cccn2c3ccc(cc3)S(=O)(=O)N)c4ccc(cc4)C(=O)OC(C)C
Cc1ccc(cc1Cl)N2C(=O)/C(=C\c3cccn3c4ccc(cc4)S(=O)(=O)N)/C(=N2)C
COc1ccc(cc1)C(=O)C2=C(C(=O)N([C@@H]2c3ccccc3Cl)CCc4c[nH]c5c4cccc5)[O-]
COc1ccc(cc1)C(=O)C2=C(C(=O)N([C@H]2c3ccccc3Cl)CCc4c[nH]c5c4cccc5)[O-]
Cc1ccc(cc1Cl)N2C(=O)/C(=C\c3ccc(c(c3)OC)OCc4ccc(cc4)Cl)/C(=O)N2
COc1cc(ccc1OCC(=O)[O-])/C=C\2/C(=O)N(C(=O)S2)c3cccc(c3)Cl
COc1ccc(cc1)[C@@H]2C(=C(C(=O)N2CCc3c[nH]c4c3cccc4)[O-])C(=O)c5ccc(cc5)Cl
COc1ccc(cc1)[C@H]2C(=C(C(=O)N2CCc3c[nH]c4c3cccc4)[O-])C(=O)c5ccc(cc5)Cl
COC(=O)c1ccc(cc1)[C@@H]2C(=C(C(=O)N2CC[NH2+]CCO)O)C(=O)c3ccccc3
COC(=O)c1ccc(cc1)[C@H]2C(=C(C(=O)N2CC[NH2+]CCO)O)C(=O)c3ccccc3
CCCN1C(=O)/C(=C\c2ccc(c(c2)OCC)OCC(C)C)/SC1=Nc3ccccc3
c1ccc(cc1)C(=O)C2=C(C(=O)N([C@@H]2c3ccccc3Cl)CC[NH+]4CCOCC4)O
c1ccc(cc1)C(=O)C2=C(C(=O)N([C@H]2c3ccccc3Cl)CC[NH+]4CCOCC4)O
C[C@H]1CC[C@@]2([C@@H]3C[C@@H]4[C@@H]([C@@H]3CC(=O)[C@H]2C1)CC[C@@H]5[C@@H]4C[NH+]6C[C@H](CC[C@@H]6[C@@]5(C)O)C)C
C[C@H]1CC[C@@]2([C@H]3C[C@@H]4[C@@H]([C@@H]3CC(=O)[C@H]2C1)CC[C@@H]5[C@@H]4C[NH+]6C[C@H](CC[C@@H]6[C@@]5(C)O)C)C
C[C@H]1CC[C@@]2([C@@H]3C[C@H]4[C@@H]([C@@H]3CC(=O)[C@H]2C1)CC[C@@H]5[C@@H]4C[NH+]6C[C@H](CC[C@@H]6[C@@]5(C)O)C)C
C[C@H]1CC[C@@]2([C@H]3C[C@H]4[C@@H]([C@@H]3CC(=O)[C@H]2C1)CC[C@@H]5[C@@H]4C[NH+]6C[C@H](CC[C@@H]6[C@@]5(C)O)C)C
c1ccc(cc1)[C@H]2CC(=C(/C(=N/Cc3ccc4c(c3)OCO4)/C2)C(=S)Nc5ccccc5)[O-]
COC(=O)C1=C(C(=C([C@@]2([C@@H]1c3cccc4c3c2ccc4)[NH+]5CCCC5)C(=O)OC)C(=O)OC)C(=O)OC
COC(=O)C1=C(C(=C([C@]2([C@@H]1c3cccc4c3c2ccc4)[NH+]5CCCC5)C(=O)OC)C(=O)OC)C(=O)OC
COC(=O)C1=C(C(=C([C@@]2([C@H]1c3cccc4c3c2ccc4)[NH+]5CCCC5)C(=O)OC)C(=O)OC)C(=O)OC
```

```
COC(=O)C1=C(C(=C([C@]2([C@H]1c3cccc4c3c2ccc4)[NH+]5CCCC5)C(=O)OC)C(=O)OC)C(=O)OC
CC1=C(C2=C([C@@H]3N(c4ccccc4S3)[C@@H]([C@@]2(O1)c5ccccc5)C(=O)c6ccccc6)c7ccccc7)C(=O)C
CC1=C(C2=C([C@H]3N(c4ccccc4S3)[C@@H]([C@@]2(O1)c5ccccc5)C(=O)c6ccccc6)c7ccccc7)C(=O)C
c1ccc(cc1)C(=O)[C@@H]2[C@@]3(C(=C4c5ccccc5C(=O)c6c4cccc6)[C@]3([C@@H]7N2c8ccccc8S7)c9ccccc9)c1ccccc1
c1ccc(cc1)C(=O)[C@H]2[C@@]3(C(=C4c5ccccc5C(=O)c6c4cccc6)[C@@]3([C@@H]7N2c8ccccc8S7)c9ccccc9)c1ccccc1
c1ccc(cc1)C(=O)C2=CN3c4ccccc4S[C@@]([C@@H]3[C@@H]5N2c6ccccc6S5)(c7ccccc7)O
c1ccc(cc1)C(=O)C2=CN3c4ccccc4S[C@]([C@@H]3[C@@H]5N2c6ccccc6S5)(c7ccccc7)O
c1ccc(cc1)C(=O)C2=CN3c4ccccc4S[C@@]([C@H]3[C@@H]5N2c6ccccc6S5)(c7ccccc7)O
c1ccc(cc1)C(=O)C2=CN3c4ccccc4S[C@]([C@H]3[C@@H]5N2c6ccccc6S5)(c7ccccc7)O
CC1(N([C@H]([C@@H](N1C(=O)[C@@H]2CC(=NO2)c3ccccc3)c4ccccc4)c5ccccc5)C(=O)[C@@H]6CC(=NO6)c7ccccc7)C
CC1(N([C@H]([C@@H](N1C(=O)[C@H]2CC(=NO2)c3ccccc3)c4ccccc4)c5ccccc5)C(=O)[C@@H]6CC(=NO6)c7ccccc7)C
CC1(N([C@H]([C@@H](N1C(=O)[C@H]2CC(=NO2)c3ccccc3)c4ccccc4)c5ccccc5)C(=O)[C@H]6CC(=NO6)c7ccccc7)C
c1ccc(cc1)C(=C2C=C[C@@H]3[C@@H]2[C@@]4(n5c6ccccc6nc5[C@]3(S4)c7ccccc7)c8ccccc8)c9ccccc9
c1ccc(cc1)C(=C2C=C[C@H]3[C@@H]2[C@@]4(n5c6ccccc6nc5[C@]3(S4)c7ccccc7)c8ccccc8)c9ccccc9
c1ccc(cc1)C(=C2C=C[C@@H]3[C@H]2[C@@]4(n5c6ccccc6nc5[C@]3(S4)c7ccccc7)c8ccccc8)c9ccccc9
c1ccc(cc1)C(=C2C=C[C@H]3[C@H]2[C@@]4(n5c6ccccc6nc5[C@]3(S4)c7ccccc7)c8ccccc8)c9ccccc9
COC(=O)[C@H]1[C@@H]([C@@]2(n3c4ccccc4nc3[C@]1(S2)c5ccccc5)c6ccccc6)C(=O)OC
c1cc(ccc1COc2ccc(cc2)/C=C\3/C(=O)N(C(=O)S3)c4ccc(cc4)Cl)C(=O)[O-]
c1ccc(cc1)[C@@H]2[C@@H]([C@@]23c4cccc5c4c(ccc5)C3=O)C(=O)c6ccc(cc6)Br
c1ccc(cc1)Sc2ccc(o2)/C=C\3/C(=O)N(C(=O)S3)c4ccc(cc4)Cl
c1ccc(cc1)[C@H]2[C@@H]([C@@]23c4cccc5c4c(ccc5)C3=O)C(=O)c6ccc(cc6)Br
c1ccc(cc1)[C@@H]2[C@H]([C@@]23c4cccc5c4c(ccc5)C3=O)C(=O)c6ccc(cc6)Br
c1ccc(cc1)[C@H]2[C@H]([C@@]23c4cccc5c4c(ccc5)C3=O)C(=O)c6ccc(cc6)Br
COC(=O)[C@@H]1C(=C(S[C@]1(c2ccccc2)c3[nH]c4ccccc4n3)c5ccccc5)C(=O)OC
COc1ccc(cc1)N2C=C[C@@H]([C@H](S2(=O)=O)C(=O)c3ccccc3)c4ccccc4
c1ccc2c(c1)C(=O)c3cc4c5c(c3C2=O)cccc5C(=O)OC4=O
Cc1cccc(c1n2c(cc(c2C)/C=C\3/C(=O)N(C(=O)S3)c4ccc(cc4)Cl)C)C
CC1=C(S[C@]([C@H]1C(=O)OC)(c2ccccc2)c3[nH]c4ccccc4n3)c5ccccc5
c1ccc(c(c1)C(=O)c2ccc3c4c2cccc4C(=O)OC3=O)C(=O)[O-]
c1cc(ccc1NC(=S)c2c(nc(s2)N3CCOCC3)N4CCOCC4)Cl
COc1cc(ccc1C(=O)/C=C/c2ccc(cc2)OCc3ccccc3)OCc4ccccc4
```

```
c1ccc(cc1)COc2ccc(cc2)/C=C/C(=O)c3ccc(cc3O)OCc4ccccc4
c1ccc(cc1)/C(=C\C(=O)Nc2ccc(cc2)N3CCN(CC3)c4cccc(c4)Cl)/C(=O)[O-]
c1ccc(cc1)/C(=C\C(=O)Nc2ccc(cc2)N3CCN(CC3)c4ccc(cc4)Cl)/C(=O)[O-]
c1ccc(cc1)/C(=C\C(=O)Nc2ccc(cc2)N3CCN(CC3)c4cccc(c4Cl)Cl)/C(=O)[O-]
c1ccc(cc1)/C(=C\C(=O)Nc2ccc(cc2)N3CCN(CC3)c4ccc(cc4)F)/C(=O)[O-]
c1ccc(cc1)C[NH+]2CCN(CC2)c3ccc(cc3)NC(=O)/C=C(\c4ccccc4)/C(=O)[O-]
CCN1C(=O)/C(=C/c2ccc(c(c2)OC)OCC(=O)[O-])/SC1=Nc3ccc(cc3)OCC
c1cc(ccc1NC(=O)[C@@H]2CC=CC[C@@H]2C(=O)[O-])N3CCN(CC3)c4ccc(cc4)Cl
c1cc(ccc1NC(=O)[C@H]2CC=CC[C@@H]2C(=O)[O-])N3CCN(CC3)c4ccc(cc4)Cl
c1cc(ccc1NC(=O)[C@@H]2CC=CC[C@H]2C(=O)[O-])N3CCN(CC3)c4ccc(cc4)Cl
CCN\1C(=O)/C(=C/c2cc(c(c(c2)Cl)OCC=C)OCC)/S/C1=N/c3ccc(cc3)OCC
c1cc(ccc1NC(=O)[C@H]2CC=CC[C@H]2C(=O)[O-])N3CCN(CC3)c4ccc(cc4)Cl
CCN1C(=O)/C(=C/c2cc(c(c(c2)Cl)OCC(=O)[O-])OC)/SC1=Nc3ccc(cc3)OCC
c1cc(ccc1NC(=O)[C@@H]2CCCC[C@@H]2C(=O)[O-])N3CCN(CC3)c4ccc(cc4)Cl
c1cc(ccc1NC(=O)[C@H]2CCCC[C@@H]2C(=O)[O-])N3CCN(CC3)c4ccc(cc4)Cl
c1cc(ccc1NC(=O)[C@@H]2CCCC[C@H]2C(=O)[O-])N3CCN(CC3)c4ccc(cc4)Cl
c1cc(ccc1NC(=O)[C@H]2CCCC[C@H]2C(=O)[O-])N3CCN(CC3)c4ccc(cc4)Cl
Cc1ccc(cc1)C(=O)On2c3ccccc3nn2
c1cc(c(c(c1)Cl)Cl)N2CCN(CC2)c3ccc(cc3)NC(=O)C=CC(=O)[O-]
c1cc(ccc1NC(=O)/C=C\C(=O)[O-])N2CCN(CC2)c3ccc(c(c3)Cl)Cl
CCCN1C(=O)/C(=C/c2ccc(c(c2)[N+](=O)[O-])[O-])/SC1=Nc3ccc(cc3)OCC
CCCN1C(=O)/C(=C/c2ccc(cc2)OCC(=O)[O-])/SC1=Nc3ccc(cc3)OCC
CCOC(=O)c1ccc(cc1)/N=C\2/N(C(=O)/C(=C/c3ccc(c(c3)OC)OCC(=O)OC)/S2)C
c1ccc(c(c1)C(=O)N2CCN(CC2)c3ccc(cc3)F)NC(=O)[C@@H]4CCCC[C@@H]4C(=O)[O-]
c1ccc(c(c1)C(=O)N2CCN(CC2)c3ccc(cc3)F)NC(=O)[C@@H]4CCCC[C@H]4C(=O)[O-]
c1ccc(c(c1)C(=O)N2CCN(CC2)c3ccc(cc3)F)NC(=O)[C@H]4CCCC[C@H]4C(=O)[O-]
c1ccc(c(c1)C(=O)Nc2cccc(c2Cl)Cl)NC(=O)[C@H]3CC=CC[C@@H]3C(=O)[O-]
CN\1C(=O)/C(=C/c2cc(c(c(c2)Cl)OCC#C)OC)/S/C1=N/c3ccc(cc3)C(=O)OC
CCOc1ccc(cc1/C=C\2/C(=O)N(/C(=N\c3ccc(cc3)C(=O)OC)/S2)C)Br
Cc1cccc(c1C)n2c(cc(c2C)/C=C\3/C(=O)N(/C(=N\c4ccc(cc4)C(=O)OC)/S3)C)C
CCN(CC)c1ccc(c(c1)OCC)/C=C\2/C(=O)N(/C(=N\c3ccc(cc3)C(=O)OC)/S2)C
```

CCOc1cc(ccc1OCC(=O)OC)/C=C\2/C(=O)N(/C(=N\c3ccc(cc3)C(=O)OC)/S2)C
Cc1cc(c(n1c2cccc(c2)[N+](=O)[O-])C)/C=C\3/C(=O)N(/C(=N\c4ccc(cc4)C(=O)OC)/S3)C
Cc1cc(c(n1c2ccccc2F)C)/C=C\3/C(=O)N(/C(=N\c4ccc(cc4)C(=O)OC)/S3)C
Cc1cccc(c1)n2c(cc(c2C)/C=C\3/C(=O)N(/C(=N\c4ccc(cc4)C(=O)OC)/S3)C)C
Cc1cc(c(n1c2ccc(cc2)OC)C)/C=C\3/C(=O)N(/C(=N\c4ccc(cc4)C(=O)OC)/S3)C
Cc1cc(c(n1c2ccc(cc2)N(C)C)C)/C=C\3/C(=O)N(/C(=N\c4ccc(cc4)C(=O)OC)/S3)C
Cc1ccccc1n2c(cc(c2C)/C=C\3/C(=O)N(/C(=N\c4ccc(cc4)C(=O)OC)/S3)C)C
Cc1cc(c(n1c2ccc(cc2)F)C)/C=C\3/C(=O)N(/C(=N\c4ccc(cc4)C(=O)OC)/S3)C
CCc1ccc(cc1)n2c(cc(c2C)/C=C\3/C(=O)N(/C(=N\c4ccc(cc4)C(=O)OC)/S3)C)C
Cc1ccc(c(c1)C)n2c(cc(c2C)/C=C\3/C(=O)N(/C(=N\c4ccc(cc4)C(=O)OC)/S3)C)C
CCOc1ccc(cc1OC)/C=C\2/C(=O)N(/C(=N\c3ccc(cc3)C(=O)OCC)/S2)C
CCOc1cc(ccc1OCC#N)/C=C\2/C(=O)N(/C(=N\c3ccc(cc3)C(=O)OCC)/S2)C
CCOC(=O)c1ccc(cc1)/N=C\2/N(C(=O)/C(=C/c3ccc(c(c3)OC)OCC#N)/S2)C
CCOc1cc(cc(c1O)Cl)/C=C\2/C(=O)N(/C(=N\c3ccc(cc3)C(=O)OCC)/S2)C
CCOc1c(cc(cc1Cl)/C=C\2/C(=O)N(/C(=N\c3ccc(cc3)C(=O)OCC)/S2)C)Cl
CCOC(=O)COc1ccc(cc1OC)/C=C\2/C(=O)N(/C(=N\c3ccc(cc3)C(=O)OCC)/S2)C
CCOc1cccc(c1O)/C=C\2/C(=O)N(/C(=N\c3ccc(cc3)C(=O)OCC)/S2)C
CCOc1ccc(cc1/C=C\2/C(=O)N(/C(=N\c3ccc(cc3)C(=O)OCC)/S2)C)Br
CCN(CC)c1ccc(c(c1)OCC)/C=C\2/C(=O)N(/C(=N\c3ccc(cc3)C(=O)OCC)/S2)C
CCc1ccc(cc1)/N=C\2/NC(=O)/C(=C\c3cc(c(c(c3)Cl)OCC#N)OC)/S2
CCc1ccc(cc1)/N=C\2/NC(=O)/C(=C\c3ccc(c(c3)Br)OCC(=O)OCC)/S2
Cn1c2ccccc2nc1CNc3ccc(cc3)N4CCN(CC4)c5ccc(cc5)Cl
COc1ccccc1N2CCN(CC2)c3ccccc3NC(=O)c4ccccc4C(=O)[O-]
CN(C)C(=O)CN(c1cc2c3ccccc3oc2cc1OC)S(=O)(=O)c4ccccc4
COc1cc2c(cc1N(CC(=O)N3CCCCC3)S(=O)(=O)c4ccccc4)c5ccccc5o2
COc1ccc(cc1)CCNC(=O)CN(C2CCCCC2)S(=O)(=O)c3ccccc3
COc1ccc(cc1)CCNC(=O)CN(Cc2ccc(cc2)Cl)S(=O)(=O)c3ccccc3
c1ccc2cc(ccc2c1)S(=O)(=O)/N=C/3\C=C(C(=O)c4c3cccc4)Br
c1ccc(cc1)OC(=O)N(c2ccc(cc2)OC(=O)Oc3ccccc3)S(=O)(=O)c4ccccc4
C[C@@H]1CCc2c(c3cc(ccc3o2)NS(=O)(=O)c4ccc(cc4)Br)C1
C[C@H]1CCc2c(c3cc(ccc3o2)NS(=O)(=O)c4ccc(cc4)Br)C1

```
Cc1ccc(cc1)S(=O)(=O)Nc2cc3c4c(oc3c5c2cccc5)CC[C@@H](C4)C(C)(C)C
Cc1ccc(cc1)S(=O)(=O)Nc2cc3c4c(oc3c5c2cccc5)CC[C@H](C4)C(C)(C)C
CCOC(=O)c1c2cc(ccc2oc1c3ccccc3)NS(=O)(=O)c4cc(ccc4Cl)Cl
c1ccc2c(c1)C(=O)c3ccc(cc3C2=O)S(=O)(=O)Nc4ccc(cc4)Br
CCOc1cc(ccc1OCc2ccc(cc2)C(=O)[O-])/C=C\3/C(=O)N(C(=O)S3)Cc4ccccc4
c1cc2c(ccc3c2c(c1)C(=O)OC3=O)Br
CCOc1ccc(cc1)N2C(=O)/C(=C/c3ccccc3OCc4cccc(c4)F)/NC2=S
CCC(C)(C)[C@@H]1CCc2c(c3cc(c4ccccc4c3o2)NS(=O)(=O)c5ccc(cc5C)C)C1
CCC(C)(C)[C@H]1CCc2c(c3cc(c4ccccc4c3o2)NS(=O)(=O)c5ccc(cc5C)C)C1
Cc1ccc(c(c1)C)S(=O)(=O)Nc2cc3c(c(oc3c4c2cccc4)C)C(=O)OC(C)C
CCOc1cc(ccc1OCc2ccccc2C#N)/C=C\3/C(=O)N(/C(=N\c4ccc(cc4)C)/S3)C
CCc1ccc(cc1)S(=O)(=O)Nc2cc3c(c(oc3c4c2cccc4)C)C(=O)OC(C)C
CCOc1ccc(cc1OC)/C=C\2/C(=O)N(/C(=N\c3cccc(c3)C(F)(F)F)/S2)C
Cc1c(c2cc(c3ccccc3c2o1)NS(=O)(=O)c4ccc5ccccc5c4)C(=O)OC(C)C
c1ccc(cc1)N2C(=O)/C(=C/c3ccc(cc3)OCc4ccc(cc4)C(=O)[O-])/NC2=S
Cc1c(c2cc(c3ccccc3c2o1)NS(=O)(=O)c4cc(ccc4Cl)Cl)C(=O)OC(C)C
CCOc1ccc(cc1)N2C(=O)/C(=C/c3cc(c(c(c3)Br)OCC#C)OC)/NC2=S
Cc1ccc(c(c1)S(=O)(=O)Nc2cc3c(c(oc3c4c2cccc4)C)C(=O)OC(C)C)C
Cc1c(c2cc(ccc2o1)NS(=O)(=O)c3ccc4ccccc4c3)C(=O)OC(C)C
c1ccc(cc1)S(=O)(=O)Nc2cc(c(c3c2cccc3)O)Sc4nc5ccccc5s4
CCCN1C(=O)/C(=C/c2cc(c(c(c2)OCC)OCc3ccccc3C#N)CC=C)/SC1=O
c1ccc2c(c1)c(cc(c2O)Sc3nc4ccccc4s3)NS(=O)(=O)c5ccc(cc5)Cl
Cc1ccc(c(c1)C)S(=O)(=O)Nc2cc(c(c3c2cccc3)O)Sc4nc5ccccc5s4
CN1C(=O)/C(=C/c2cc(c(cc2Br)OCc3ccc(cc3)Cl)OC)/NC1=S
CCc1ccc(cc1)S(=O)(=O)Nc2ccc(c(c2)Sc3nc4ccccc4s3)O
CN1C(=O)/C(=C/c2ccc(c(c2)Br)OCc3ccc(cc3Cl)Cl)/NC1=S
c1ccc2c(c1)nc(s2)Sc3cc(ccc3O)[N-]S(=O)(=O)c4cc(ccc4Cl)Cl
Cc1c(c2cc(c3ccccc3c2o1)NS(=O)(=O)c4ccc(cc4)C(C)(C)C)C(=O)OCCOC
Cc1cc(c(c(c1)C)S(=O)(=O)Nc2cc3c(c(oc3c4c2cccc4)C)C(=O)OCCOC)C
CCc1ccc(cc1)S(=O)(=O)Nc2cc3c(c(oc3c4c2cccc4)C)C(=O)OCCOC
COc1cc(ccc1OCc2ccccc2C#N)/C=C\3/C(=O)N(C(=O)S3)CC(=O)N4CCOC
```

```
Cc1ccc(cc1)S(=O)(=O)Nc2cc3c(c(oc3c4c2cccc4)C)C(=O)OCCOC
Cc1c(c2cc(c3ccccc3c2o1)NS(=O)(=O)c4ccc(cc4)F)C(=O)OCCOC
CC1(Cc2c(c3cc(c4ccccc4c3o2)NS(=O)(=O)c5cc(ccc5Cl)Cl)C(=O)C1)C
CCOC(=O)C1=C(N=c2n(c(=O)/c(=C/c3cc(c(cc3[N+](=O)[O-])OC)OC)/s2)[C@@H]1c4cccc(c4)OC)c5ccccc5
COc1ccc2cc(ccc2c1S(=O)(=O)N3CCOCC3)S(=O)(=O)N4CCOCC4
CCOC(=O)C1=C(N=c2n(c(=O)/c(=C/c3cc(c(cc3[N+](=O)[O-])OC)OC)/s2)[C@H]1c4cccc(c4)OC)c5ccccc5
CCOC(=O)c1c2cc(c3ccccc3c2oc1c4ccccc4)NS(=O)(=O)c5cc(ccc5C)C
CCOc1ccc(cc1)S(=O)(=O)Nc2cc3c(c(oc3c4c2cccc4)C)C(=O)OC
CCOc1ccc(cc1)S(=O)(=O)Nc2cc3c4c(oc3c5c2cccc5)CC[C@@H](C4)C
CCOc1ccc(cc1)S(=O)(=O)Nc2cc3c4c(oc3c5c2cccc5)CC[C@H](C4)C
CCOC(=O)C1=C(N=c2n(c(=O)/c(=C/c3ccncc3)/s2)[C@@H]1c4cccc(c4)OC)c5ccccc5
Cc1ccc(cc1)C(=O)N(c2ccc3c(c2)c4c(o3)CCCC4)S(=O)(=O)c5ccc(cc5)C
CCOC(=O)C1=C(N=c2n(c(=O)/c(=C/c3ccncc3)/s2)[C@H]1c4cccc(c4)OC)c5ccccc5
C[C@@H]1CCc2c(c3cc(c4ccccc4c3o2)NS(=O)(=O)c5ccc(cc5)Br)C1
CCOC(=O)C1=C(N=c2n(c(=O)/c(=C/c3cccnc3)/s2)[C@@H]1c4cccc(c4)OC)c5ccccc5
C[C@H]1CCc2c(c3cc(c4ccccc4c3o2)NS(=O)(=O)c5ccc(cc5)Br)C1
CCOC(=O)C1=C(N=c2n(c(=O)/c(=C/c3cccnc3)/s2)[C@H]1c4cccc(c4)OC)c5ccccc5
Cc1ccc(cc1)C(=O)N(c2ccc3c(c2)c4c(o3)CCCC4)S(=O)(=O)c5ccc(cc5)F
c1cc(ccc1C(=O)N(c2ccc3c(c2)c4c(o3)CCCC4)S(=O)(=O)c5ccc(cc5)F)Cl
CCOC(=O)C1=C(N=c2n(c(=O)/c(=C/c3ccc(o3)N4CCOCC4)/s2)[C@@H]1c5cccc(c5)OC)c6ccccc6
Cc1cc(c(c(c1)C)S(=O)(=O)N(c2ccc3c(c2)c4c(o3)CCCC4)C(=O)c5ccccc5)C
CCc1ccc(cc1)S(=O)(=O)N(c2ccc3c(c2)c4c(o3)CCCC4)C(=O)c5ccccc5
COc1ccc(cc1)S(=O)(=O)N(c2ccc3c(c2)c4c(o3)CCCC4)C(=O)c5ccccc5
Cc1ccc(cc1)C(=O)N(c2ccc3c(c2)c4c(o3)CCCC4)S(=O)(=O)c5ccc(cc5)OC
CCOC(=O)C1=C(N=c2n(c(=O)/c(=C/c3ccc(o3)N4CCOCC4)/s2)[C@H]1c5cccc(c5)OC)c6ccccc6
COc1ccc(cc1)S(=O)(=O)N(c2ccc3c(c2)c4c(o3)CCCC4)C(=O)c5ccc(cc5)Cl
CCOC(=O)C1=C(N=c2n(c(=O)/c(=C\c3cc(n(c3C)c4cccnc4)C)/s2)[C@@H]1c5cccc(c5)OC)c6ccccc6
CCOc1ccc(cc1)S(=O)(=O)N(c2ccc3c(c2)c4c(o3)CCCC4)C(=O)c5ccccc5
CC(C)(C)c1ccc(cc1)S(=O)(=O)N(c2ccc3c(c2)c4c(o3)CCCC4)C(=O)c5ccccc5
Cc1ccc(cc1)C(=O)N(c2ccc3c(c2)c4c(o3)CCCC4)S(=O)(=O)c5cc(ccc5C)C
CCOC(=O)C1=C(N=c2n(c(=O)/c(=C\c3cc(n(c3C)c4cccnc4)C)/s2)[C@H]1c5cccc(c5)OC)c6ccccc6
```

```
Cc1ccc(c(c1)S(=O)(=O)N(c2ccc3c(c2)c4c(o3)CCCC4)C(=O)c5ccc(cc5)Cl)C
Cc1ccc(c(c1)S(=O)(=O)N(c2ccc3c(c2)c4c(o3)CCCC4)C(=O)c5ccccc5)C
CC(C)c1ccc(cc1)S(=O)(=O)N(c2ccc3c(c2)c4c(o3)CCCC4)C(=O)c5ccccc5
Cc1ccc(cc1)C(=O)N(c2ccc3c(c2)c4c(o3)CCCC4)S(=O)(=O)c5ccc(cc5)C(C)C
COc1ccc(cc1)NC2=C/C(=N\S(=O)(=O)c3ccc(cc3)Br)/c4ccccc4C2=O
CCOC(=O)C1=C(N=c2n(c(=O)/c(=C\c3ccccc3O)/s2)[C@@H]1c4cccc(c4)OC)c5ccccc5
COc1ccc(cc1)NC2=C/C(=N\S(=O)(=O)c3ccc(cc3)F)/c4ccccc4C2=O
CCOC(=O)C1=C(N=c2n(c(=O)/c(=C\c3ccccc3O)/s2)[C@H]1c4cccc(c4)OC)c5ccccc5
CCc1ccc(cc1)S(=O)(=O)/N=C/2\C=C(C(=O)c3c2cccc3)Nc4ccc(cc4)OC
COc1ccc(cc1)NC2=CC(=NS(=O)(=O)c3ccc(cc3)OC)c4ccccc4C2=O
CC(C)(C)c1ccc(cc1)S(=O)(=O)/N=C/2\C=C(C(=O)c3c2cccc3)Nc4ccc(cc4)OC
Cc1ccc(c(c1)S(=O)(=O)/N=C/2\C=C(C(=O)c3c2cccc3)Nc4ccc(cc4)OC)C
Cc1cc(c(cc1C)S(=O)(=O)/N=C/2\C=C(C(=O)c3c2cccc3)Nc4ccc(cc4)OC)C
Cc1ccc(c(c1)C)NC2=C/C(=N\S(=O)(=O)c3ccc(cc3)OC)/c4ccccc4C2=O
CCOc1ccc(cc1)NC2=C/C(=N\S(=O)(=O)c3ccc(cc3)C)/c4ccccc4C2=O
CCOc1ccc(cc1)NC2=C/C(=N\S(=O)(=O)c3ccc(cc3C)C)/c4ccccc4C2=O
CCOc1cc(ccc1OCc2ccccc2F)/C=C\3/C(=O)N(C(=O)S3)CC(=O)N4CCOCC4
CCc1ccc(cc1)S(=O)(=O)/N=C/2\C=C(C(=O)c3c2cccc3)Nc4ccc(cc4)OCC
CCOc1cc(cc(c1O)I)/C=C\2/C(=O)N(C(=O)S2)c3cccc(c3)Cl
CCOc1ccc(cc1)NC2=C/C(=N\S(=O)(=O)c3ccc(cc3)C(C)C)/c4ccccc4C2=O
Cc1ccc(cc1C)N2C(=O)/C(=C/c3cc(cc(c3OC)I)I)/NC2=S
CCOc1ccc(cc1)NC2=C/C(=N\S(=O)(=O)c3ccc(cc3)C(C)(C)C)/c4ccccc4C2=O
Cc1ccc(c(c1)C)S(=O)(=O)/N=C/2\C=C(C(=O)c3c2cccc3)Nc4ccccc4C(=O)OC
CCc1ccc(cc1)S(=O)(=O)/N=C/2\C=C(C(=O)c3c2cccc3)Nc4ccccc4C(=O)OC
CCOc1ccc(cc1)S(=O)(=O)/N=C/2\C=C(C(=O)c3c2cccc3)Nc4ccccc4C(=O)OC
Cc1ccc(c(c1)C)S(=O)(=O)/N=C/2\C=C(C(=O)c3c2cccc3)Sc4nc5ccccc5s4
Cc1ccc(c(c1)S(=O)(=O)/N=C/2\C=C(C(=O)c3c2cccc3)Sc4nc5ccccc5s4)C
c1ccc(cc1)S(=O)(=O)/N=C/2\C=C(C(=O)c3c2cccc3)Sc4nc5ccccc5s4
c1cc(ccc1N(C(=O)c2ccncc2)S(=O)(=O)c3ccc(cc3)Cl)OC(=O)c4ccncc4
Cc1ccc(cc1)C(=O)N(c2ccc3c(c2)c(c(o3)C)C(=O)OC)S(=O)(=O)c4ccc(cc4C)C
COC(=O)c1ccc(cc1)NC(=O)CSc2nc3ccc(cc3s2)NC(=O)CSc4nnnn4Cc5c
```

```
CCOC(=O)c1c(oc2c1cc(cc2)N(C(=O)c3ccccc3)S(=O)(=O)c4ccc(cc4C)C)C
Cc1ccccc1OCc2nnc(n2C)SCC(=O)Nc3ccc4c(c3)sc(n4)SCC(=O)Nc5ccc(cc5)C(=O)OC
Cc1ccc(c(c1)C)S(=O)(=O)N(c2ccc3c(c2)c4c(o3)CCCC4)C(=O)c5ccncc5
CCCC(=O)N(c1ccc2c(c1)c3c(o2)CCCC3)S(=O)(=O)c4ccc(cc4)C(C)C
CCC(C)(C)[C@@H]1CCc2c(c3cc(ccc3o2)NS(=O)(=O)c4ccc(cc4)C(C)C)C1
Cn1c(nnc1SCC(=O)Nc2ccc3c(c2)sc(n3)SCC(=O)Nc4ccc(cc4)C(=O)OC)c5ccc(cc5)N
CCC(C)(C)[C@H]1CCc2c(c3cc(ccc3o2)NS(=O)(=O)c4ccc(cc4)C(C)C)C1
Cc1ccc(cc1)C(=O)N(c2ccc3c(c2)c(c(o3)C)C(=O)C)S(=O)(=O)c4cc(ccc4C)C
Cc1ccc(c(c1)S(=O)(=O)N(c2ccc3c(c2)c(c(o3)C)C(=O)OC)C(=O)c4ccccc4)C
CCOC(=O)c1c(oc2c1cc(cc2)N(C(=O)c3ccccc3)S(=O)(=O)c4cc(ccc4C)C)C
Cc1ccc(cc1)C(=O)N(c2ccc3c(c2)c(c(o3)C)C(=O)OC)S(=O)(=O)c4cc(ccc4C)C
c1ccc(cc1)OCCNC(=O)CSc2nc3ccc(cc3s2)NC(=O)CSCC(=O)Nc4ccc5ccccc5c4
CCCC(=O)N(c1ccc2c(c1)c(c(o2)C)C(=O)OCC)S(=O)(=O)c3cc(ccc3C)C
CCCC(=O)N(c1ccc2c(c1)c(c(o2)C)C(=O)OC)S(=O)(=O)c3cc(ccc3C)C
CCOC(=O)c1ccc(cc1)N2C(=O)/C(=C\c3cc(c(cc3Br)OC)OC)/C(=N2)C(F)(F)F
Cc1ccc(c(c1)S(=O)(=O)N(c2ccc3c(c2)c4c(o3)CCCC4)C(=O)c5ccncc5)C
CCOC(=O)c1ccc(cc1)N2C(=O)C(=Cc3cc(c(c(c3)Br)O)OC)C(=N2)C(F)(F)F
CCOC(=O)c1c2cc(ccc2oc1c3ccccc3)N(C(=O)C)S(=O)(=O)c4ccc(cc4C)C
CCCC(=O)N(c1ccc2c(c1)c(c(o2)C)C(=O)OCC)S(=O)(=O)c3ccc(cc3C)C
Cc1ccc(c(c1)C)S(=O)(=O)N(c2ccc3c(c2)c(c(o3)C)C(=O)C)C(=O)c4ccncc4
c1cc(ccc1N2C(=O)/C(=C\c3ccc(o3)c4ccc(c(c4)Cl)Cl)/C(=N2)C(F)(F)F)S(=O)(=O)N
c1cc(ccc1N2C(=O)/C(=C\c3cc(c(c(c3)Br)O)Br)/C(=N2)C(F)(F)F)S(=O)(=O)N
c1cc(ccc1N(C(=O)c2ccncc2)S(=O)(=O)c3ccc(cc3)F)OC(=O)c4ccncc4
CCCC(=O)N(c1ccc2c(c1)c3c(o2)CCCC3)S(=O)(=O)c4ccc(cc4)Br
CCN\1C(=O)/C(=C/c2c(ccc3c2cc(cc3)OC)OC)/S/C1=N\c4ccc(c(c4)Cl)Br
Cc1cc(c(cc1C)S(=O)(=O)N(c2ccc3c(c2)c4c(o3)CCCC4)C(=O)c5ccncc5)C
CCCC(=O)N(c1ccc2c(c1)c3c(o2)CCCC3)S(=O)(=O)c4cc(c(cc4C)C)C
Cc1cc(c(cc1C)S(=O)(=O)N(c2ccc3c(c2)c(c(o3)C)C(=O)OC)C(=O)c4ccccc4)C
Cc1cc(c(cc1C)S(=O)(=O)N(c2ccc3c(c2)c(c(o3)C)C(=O)C)C(=O)c4ccccc4)C
Cc1ccc(cc1)C(=O)N(c2ccc3c(c2)c(c(o3)C)C(=O)C)S(=O)(=O)c4cc(c(cc4C)C)C
CCCC(=O)N(c1ccc2c(c1)c(c(o2)C)C(=O)OC)S(=O)(=O)c3cc(c(cc3C)C)C
```

```
CCCC(=O)N(c1ccc2c(c1)c(c(o2)C)C(=O)OCC)S(=O)(=O)c3cc(c(cc3C)C)C
COc1ccc(cc1)S(=O)(=O)Nc2cc(c(c3c2cccc3)O)Sc4nc5ccccc5s4
CCOc1cc(cc(c1OS(=O)(=O)c2ccc(cc2)C)I)C3C4=C(CC(CC4=O)(C)C)N(C5=C3C(=O)CC(C5)(C)C)Cc6ccccc6
COc1ccc(cc1)S(=O)(=O)N(c2ccc(cc2)OC(=O)c3ccncc3)C(=O)c4ccncc4
CC1=C([C@@H](n2c(=O)/c(=C\c3cccs3)/sc2=N1)c4ccc(cc4)F)C(=O)Nc5ccccc5
Cc1c(c2cc(ccc2o1)N(C(=O)c3ccccc3)S(=O)(=O)c4ccc(cc4)OC)C(=O)OC
Cc1ccc(cc1)C(=O)N(c2ccc3c(c2)c(c(o3)C)C(=O)OC)S(=O)(=O)c4ccc(cc4)OC
CC1=C([C@H](n2c(=O)/c(=C\c3cccs3)/sc2=N1)c4ccc(cc4)F)C(=O)Nc5ccccc5
CCOC(=O)c1c(oc2c1cc(cc2)N(C(=O)c3ccccc3)S(=O)(=O)c4ccc(cc4)OC)C
CCCC(=O)N(c1ccc2c(c1)c(c(o2)C)C(=O)OCC)S(=O)(=O)c3ccc(cc3)OC
CCOC(=O)C1=C(N=c2n(c(=O)/c(=C\c3ccccn3)/s2)[C@@H]1c4ccc(cc4)OC(C)C)C
Cc1c(c2cc(ccc2o1)N(C(=O)c3ccccc3)S(=O)(=O)c4ccc(cc4)F)C(=O)OC
Cc1ccc(cc1)C(=O)N(c2ccc3c(c2)c(c(o3)C)C(=O)OC)S(=O)(=O)c4ccc(cc4)F
CCOC(=O)C1=C(N=c2n(c(=O)c(=Cc3ccccn3)s2)[C@H]1c4ccc(cc4)OC(C)C)C
CCOC(=O)c1c(oc2c1cc(cc2)N(C(=O)c3ccc(cc3)C)S(=O)(=O)c4ccc(cc4)F)C
CCCC(=O)N(c1ccc2c(c1)c3c(o2)CCCC3)S(=O)(=O)c4c(cc(cc4C)C)C
CCOc1cc(ccc1OCc2cccc(c2)F)/C=C\3/C(=NN(C3=O)c4ccccc4)C
Cc1cc(c(c(c1)C)S(=O)(=O)N(c2ccc3c(c2)c4c(o3)CCCC4)C(=O)c5ccncc5)C
Cc1cc(c(c(c1)C)S(=O)(=O)N(c2ccc(cc2)OC(=O)c3ccccc3)C(=O)c4ccccc4)C
CCC(C)(C)[C@@H]1CCc2c(c3cc(ccc3o2)NS(=O)(=O)c4c(cc(cc4C)C)C)C1
CCC(C)(C)[C@H]1CCc2c(c3cc(ccc3o2)NS(=O)(=O)c4c(cc(cc4C)C)C)C1
CCOc1ccc(cc1)S(=O)(=O)N(c2ccc3c(c2)c(c(o3)C)C(=O)OC)C(=O)c4ccccc4
CCOc1ccc(cc1)S(=O)(=O)N(c2ccc3c(c2)c(c(o3)C)C(=O)C)C(=O)c4ccc(cc4)C
CCCC(=O)N(c1ccc2c(c1)c(c(o2)C)C(=O)OCC)S(=O)(=O)c3ccc(cc3)OCC
CCOc1cc(cc(c1OCC#C)I)/C=C/2\C(=O)NC(=S)N(C2=O)CC=C
CCCC(=O)N(c1ccc2c(c1)c3c(o2)CCCC3)S(=O)(=O)c4ccc(cc4)OCC
CCC(C)(C)[C@@H]1CCc2c(c3cc(ccc3o2)NS(=O)(=O)c4ccc(cc4)OCC)C1
CCC(C)(C)[C@H]1CCc2c(c3cc(ccc3o2)NS(=O)(=O)c4ccc(cc4)OCC)C1
Cc1ccc(cc1)C(=O)N(c2ccc3c(c2)c4c(o3)CCCC4)S(=O)(=O)c5cc(c(cc5C)C)C
Cc1ccc(cc1)C(=O)N(c2ccc3c(c2)c4c(o3)CCCC4)S(=O)(=O)c5cccs5
Cc1cc(c(c(c1)C)S(=O)(=O)N(c2ccc3c(c2)c(c(o3)C)C(=O)C)C(=O)c4ccccc4)C
```

```
Cc1cc(c(c(c1)C)S(=O)(=O)N(c2ccc3c(c2)c(c(o3)C)C(=O)OC)C(=O)c4ccccc4)C
Cc1ccc(cc1)C(=O)N(c2ccc3c(c2)c4c(o3)CCCC4)S(=O)(=O)c5ccccc5
CCCCC(=O)Nc1cccc(c1)NC(=O)c2cccc3c2cccc3Br
c1ccc(cc1)S(=O)(=O)N(c2ccc3c(c2)c4c(o3)CCCC4)C(=O)c5ccc(cc5)Cl
Cc1ccc(cc1)S(=O)(=O)N(c2ccc3c(c2)c4c(o3)CCCC4)C(=O)c5ccccc5
Cc1ccc(cc1)SC2=C/C(=N\S(=O)(=O)c3ccccc3)/c4ccccc4C2=O
Cc1ccc(cc1)SC2=C/C(=N\S(=O)(=O)c3ccc(cc3)Cl)/c4ccccc4C2=O
Cc1ccc(cc1)SC2=C/C(=N\S(=O)(=O)c3ccc(cc3)Br)/c4ccccc4C2=O
Cc1ccc(cc1)SC2=C/C(=N\S(=O)(=O)c3ccc(cc3)F)/c4ccccc4C2=O
Cc1cccc(c1)N2C(=O)/C(=C\c3ccc(o3)c4ccc(c(c4)C(=O)[O-])Cl)/C(=O)N2
Cc1ccc(cc1)SC2=C/C(=N\S(=O)(=O)c3c(cc(cc3C)C)C)/c4ccccc4C2=O
Cc1cccc(c1)N2C(=O)/C(=C\c3ccc(o3)c4ccc(cc4)S(=O)(=O)N)/C(=O)N2
CCc1ccc(cc1)S(=O)(=O)/N=C/2\C=C(C(=O)c3c2cccc3)Sc4ccc(cc4)C
Cc1ccc(cc1)SC2=C/C(=N\S(=O)(=O)c3ccc(cc3)OC)/c4ccccc4C2=O
CCOc1ccc(cc1)S(=O)(=O)/N=C/2\C=C(C(=O)c3c2cccc3)Sc4ccc(cc4)C
Cc1ccc(cc1)SC2=C/C(=N\S(=O)(=O)c3cc(ccc3C)C)/c4ccccc4C2=O
CCOc1cc(ccc1Oc2c(cnc(n2)Cl)F)/C=C/3\C(=O)N(C(=O)S3)CC(=O)Nc4ccccc4C
Cc1ccc(cc1)SC2=C/C(=N\S(=O)(=O)c3ccc(cc3)C(C)C)/c4ccccc4C2=O
Cc1ccc(cc1)SC2=C/C(=N\S(=O)(=O)c3cc(c(cc3C)C)C)/c4ccccc4C2=O
Cc1ccc(cc1)S(=O)(=O)/N=C/2\C=C(C(=O)c3c2cccc3)Nc4ccccc4OC
Cc1ccccc1NC(=O)CN2C(=O)/C(=C\c3cn(c4c3cccc4)C(C)C)/SC2=O
COc1ccccc1NC2=C/C(=N\S(=O)(=O)c3ccc(cc3)Cl)/c4ccccc4C2=O
COc1ccccc1NC2=C/C(=N\S(=O)(=O)c3ccc(cc3)Br)/c4ccccc4C2=O
Cc1cc(c(c(c1)C)S(=O)(=O)N=C2C=C(C(=O)c3c2cccc3)Nc4ccccc4OC)C
CCc1ccc(cc1)S(=O)(=O)/N=C/2\C=C(C(=O)c3c2cccc3)Nc4ccccc4OC
CCOc1ccc(cc1)S(=O)(=O)/N=C/2\C=C(C(=O)c3c2cccc3)Nc4ccccc4OC
Cc1ccccc1NC(=O)CN2C(=O)/C(=C\c3ccc4c(c3)n(c(=O)n4C)C)/SC2=O
CC(C)(C)c1ccc(cc1)S(=O)(=O)/N=C/2\C=C(C(=O)c3c2cccc3)Nc4ccccc4OC
Cc1ccccc1NC(=O)CN2C(=O)/C(=C\c3ccc(o3)c4ccc(c(c4)C(=O)[O-])Cl)/SC2=O
COc1ccccc1NC2=C/C(=N\S(=O)(=O)c3ccc4ccccc4c3)/c5ccccc5C2=O
Cc1ccc(cc1)NC(=O)CN2C(=O)/C(=C\c3cn(c4c3cccc4)C(C)C)/SC2=O
```

```
CC(C)c1ccc(cc1)S(=O)(=O)/N=C/2\C=C(C(=O)c3c2cccc3)Nc4ccccc4OC
Cc1cc(c(cc1C)S(=O)(=O)/N=C/2\C=C(C(=O)c3c2cccc3)Nc4ccccc4OC)C
COc1cc(ccc1Oc2ccc(cn2)[N+](=O)[O-])/C=C/3\C(=O)N(C(=O)S3)CC(=O)N4CCOCC4
CCCCc1ccc(cc1)NC2=C/C(=N\S(=O)(=O)c3ccc(cc3)C)/c4ccccc4C2=O
CCCCc1ccc(cc1)NC2=C/C(=N\S(=O)(=O)c3ccc(cc3)F)/c4ccccc4C2=O
c1ccc(cc1)NC(=O)CN2C(=O)/C(=C\c3ccc(o3)c4ccc(c(c4)C(=O)[O-])Cl)/SC2=O
CCOC(=O)c1ccc(cc1)NC2=C/C(=N\S(=O)(=O)c3ccc(cc3)F)/c4ccccc4C2=O
CCOC(=O)c1ccc(cc1)NC2=C/C(=N\S(=O)(=O)c3ccc(cc3C)C)/c4ccccc4C2=O
CCc1ccc(cc1)S(=O)(=O)/N=C/2\C=C(C(=O)c3c2cccc3)Nc4ccc(cc4)C(=O)OCC
CCOC(=O)c1ccc(cc1)NC2=C/C(=N\S(=O)(=O)c3cccs3)/c4ccccc4C2=O
Cc1ccc(cc1C)NC(=O)COc2ccc(cc2OC)/C=C/3\C(=O)N(C(=O)S3)CC(=O)N4CCOCC4
CCOC(=O)COc1ccc(cc1)/C=C\2/C(=O)N(C(=O)S2)CC(=O)N3CCOCC3
Cc1cc(c(c(c1)C)NC2=C/C(=N\S(=O)(=O)c3cccs3)/c4ccccc4C2=O)C
CCc1ccc(cc1)S(=O)(=O)/N=C/2\C=C(C(=O)c3c2cccc3)Nc4ccc(cc4)C(=O)OC
CCOc1c(cc(cc1I)I)/C=C/2\C(=O)NC(=O)S2
COc1ccc(cc1)S(=O)(=O)/N=C/2\C=C(C(=O)c3c2cccc3)Nc4ccc(cc4)C(=O)OC
Cc1ccc(c(c1)NC2=C/C(=N\S(=O)(=O)c3ccc(cc3)F)/c4ccccc4C2=O)C
CCOc1ccc(cc1)S(=O)(=O)/N=C/2\C=C(C(=O)c3c2cccc3)Nc4cc(ccc4C)C
Cc1ccc(c(c1)NC2=C/C(=N\S(=O)(=O)c3cccs3)/c4ccccc4C2=O)C
CCOc1ccc(cc1)S(=O)(=O)/N=C/2\C=C(C(=O)c3c2cccc3)Nc4cc(ccc4C)Cl
Cc1ccc(cc1C)NC2=C/C(=N\S(=O)(=O)c3ccc(cc3)OC)/c4ccccc4C2=O
CCOc1ccc(cc1)S(=O)(=O)/N=C/2\C=C(C(=O)c3c2cccc3)Nc4ccc(c(c4)C)C
COc1cc(ccc1OCc2ccncc2)/C=C/3\C(=O)N(C(=O)S3)Cc4ccc(cc4)F
Cc1ccccc1NC2=C/C(=N\S(=O)(=O)c3ccc(cc3)OC)/c4ccccc4C2=O
CCOc1ccc(cc1)S(=O)(=O)/N=C/2\C=C(C(=O)c3c2cccc3)Nc4ccccc4C
CC(C)c1ccc(cc1)S(=O)(=O)/N=C/2\C=C(C(=O)c3c2cccc3)Nc4ccc(cc4)NC(=O)C
c1ccc(cc1)C2=C(NC(=C3C=C(C(=O)C(=C3)Br)Br)N2)c4ccccc4
Cc1cc(c(c(c1)C)S(=O)(=O)N=C2C/C(=N\Cc3ccccc3)/C(=O)c4c2cccc4)C
COc1cc(ccc1OCc2ccncc2)/C=C/3\C(=O)N(C(=O)S3)Cc4ccccc4Cl
COc1ccccc1NC2=C/C(=N\S(=O)(=O)c3ccccc3)/c4ccccc4C2=O
Cc1cc(c(cc1C)[N+](=O)[O-])c2ccc(o2)/C=c\3/c(=O)n4c5ccccc5nc4s3
```

```
CCOC(=O)c1ccc(cc1)NC2=C/C(=N\S(=O)(=O)c3ccccc3)/c4ccccc4C2=O
CCc1ccc(cc1)S(=O)(=O)/N=C/2\C=C(C(=O)c3c2cccc3)Nc4ccc(cc4)C
CCOc1ccc(cc1)S(=O)(=O)/N=C/2\C=C(C(=O)c3c2cccc3)Nc4ccc(cc4)C
COc1cc(ccc1OCc2ccncc2)/C=C/3\C(=O)N(C(=O)S3)Cc4ccccc4
CCCc1c(c2cc(c3ccccc3c2o1)NS(=O)(=O)c4cccs4)C(=O)OCC
Cc1cc(c2c(c1)n3c(=O)/c(=C/c4ccc(c(c4)OC)OCc5ccc(cc5Cl)Cl)/sc3n2)C
CC[C@@H](C)Oc1ccc(cc1OCC)/C=C/2\C(=O)N(C(=O)S2)CC(=O)Nc3ccc(c(c3)C)C
Cc1ccc(c(c1)C)S(=O)(=O)Nc2ccc3c(c2)c(c(o3)C)C(=O)OCc4ccccc4
CC[C@H](C)Oc1ccc(cc1OCC)/C=C/2\C(=O)N(C(=O)S2)CC(=O)Nc3ccc(c(c3)C)C
CCCCCOC(=O)c1c(oc2c1cc(c3c2cccc3)NS(=O)(=O)c4cc(c(cc4C)C)C)C
CCCCOC(=O)c1c(oc2c1cc(c3c2cccc3)NS(=O)(=O)c4cc(c(cc4C)C)C)C
CN(C)c1cc(c(cc1[N+](=O)[O-])/C=N/NC(=O)Cn2c3ccccc3nc2c4c(non4)N)Cl
Cc1cc(c(cc1C)S(=O)(=O)Nc2ccc(cc2)NS(=O)(=O)c3cc(c(cc3C)C)C)C
CCCCCCCC(=O)N/N=C/c1ccc(c(c1)[N+](=O)[O-])Sc2nccn2C
CCOC(=O)c1c(oc2c1cc(cc2Cl)NS(=O)(=O)c3cc(c(cc3C)C)C)C
CC[C@@H](C)Oc1ccc(cc1OCC)/C=C/2\C(=O)N(C(=O)S2)CC(=O)Nc3ccc(cc3)F
Cc1c(c(c(c(c1[N+](=O)[O-])C)[N+](=O)[O-])C)[C@@H]2c3c(n[nH]c3OC(=C2C#N)N)C
CCOC(=O)c1c2cc(ccc2oc1c3ccccc3)NS(=O)(=O)c4cc(ccc4C)C
CC[C@H](C)Oc1ccc(cc1OCC)/C=C/2\C(=O)N(C(=O)S2)CC(=O)Nc3ccc(cc3)F
Cc1c(c(c(c(c1[N+](=O)[O-])C)[N+](=O)[O-])C)[C@H]2c3c(n[nH]c3OC(=C2C#N)N)C
Cc1c(c2cc(ccc2o1)NS(=O)(=O)c3cc(ccc3Cl)Cl)C(=O)OC
Cc1ccc2c(c1)nc([nH]2)/C(=C/c3cc(c(c(c3)Br)O)Br)/C#N
Cc1cc(c(c(c1)C)S(=O)(=O)Nc2cc3c(c(oc3c4c2cccc4)C(C)(C)C)C(=O)OC)C
Cc1ccc(cc1C)NC(=O)CN2C(=O)/C(=C\c3ccc(c(c3)OC)OCC=C)/SC2=O
COc1cc(cc(c1OCC#C)I)C=NNC(=O)Cn2c3ccccc3nc2c4c(non4)N
CC(C)(C)c1c(c2cc(c3ccccc3c2o1)NS(=O)(=O)c4ccc5ccccc5c4)C(=O)OC
CCOc1cc(ccc1OCC=C)/C=C/2\C(=O)N(C(=O)S2)CC(=O)Nc3ccc(c(c3)C)C
c1ccc2c(c1)[nH]c(n2)/C(=C/c3cc(ccc3OCc4ccc(cc4Cl)Cl)Br)/C#N
CCCc1c(c2cc(ccc2o1)NS(=O)(=O)c3ccc(cc3)OCC)C(=O)OCC
CCOc1cc(ccc1OCC(C)C)/C=C\2/C(=O)N(C(=O)S2)CC(=O)Nc3ccc(cc3)F
CCCc1c(c2cc(c3ccccc3c2o1)NS(=O)(=O)c4ccc(cc4)CC)C(=O)OCC
```

CC1=C([C@@H](C(=C(N1)SCC(=O)Nc2nc3ccc(cc3s2)Br)C#N)c4cccnc4)C(=O)C
CCCc1c(c2cc(c3ccccc3c2o1)NS(=O)(=O)c4ccc(cc4)C(C)(C)C)C(=O)OCC
CCCCOC(=O)c1c(oc2c1cc(cc2)NS(=O)(=O)c3cc(ccc3Cl)Cl)C
CCCCCOC(=O)c1c(oc2c1cc(cc2)NS(=O)(=O)c3ccc(cc3)Br)C
CCCCCOC(=O)c1c(oc2c1cc(cc2)NS(=O)(=O)c3cc(ccc3Cl)Cl)C
CC1=C([C@H](C(=C(N1)SCC(=O)Nc2nc3ccc(cc3s2)Br)C#N)c4cccnc4)C(=O)C
CCCCCOC(=O)c1c(oc2c1cc(cc2)NS(=O)(=O)c3cc(ccc3C)C)C
CCCCCOC(=O)c1c(oc2c1cc(cc2)NS(=O)(=O)c3ccc(cc3)CC)C
c1ccc(cc1)COc2c(cc(cc2I)/C=c/3\c(=O)n4c5ccccc5nc4s3)I
CCCCCOC(=O)c1c(oc2c1cc(cc2)NS(=O)(=O)c3ccc(cc3)OCC)C
C=CCOc1c(cc(cc1I)/C=c/2\c(=O)n3c4ccccc4nc3s2)I
CCCCCOC(=O)c1c(oc2c1cc(c3c2cccc3)NS(=O)(=O)c4ccc(cc4)F)C
c1ccc2c(c1)nc3n2c(=O)/c(=C/c4cc(c(c(c4)I)[O-])[N+](=O)[O-])/s3
CCCCOC(=O)c1c(oc2c1cc(c3c2cccc3)NS(=O)(=O)c4ccc(cc4)F)C
CCCCOC(=O)c1c(oc2c1cc(c3c2cccc3)NS(=O)(=O)c4c(cc(cc4C)C)C)C
Cc1ccc2c(c1C)nc3n2c(=O)/c(=C/c4ccccc4OCc5ccc(cc5Cl)Cl)/s3
CCN(CC)c1ccc(c(c1)OCc2ccc(cc2)Cl)/C=C(\C#N)/c3[nH]c4ccccc4n3
CCCCOC(=O)c1c(oc2c1cc(c3c2cccc3)NS(=O)(=O)c4ccc(cc4)C(C)(C)C)C
COc1cccc(c1OCc2ccc(cc2Cl)Cl)/C=N/c3c(nc4n3cccc4)c5ccco5
Cc1cc(c(cc1C)S(=O)(=O)/N=C/2\C=C(C(=O)c3c2cccc3)Br)C
CC1CCN(CC1)c2ccc(cc2[N+](=O)[O-])/C=N/c3c([nH+]c4n3cccc4)c5ccc(c(c5)OC)O
c1ccc2c(c1)nc3n2c(=O)/c(=C\c4ccc(o4)c5ccc(cc5[N+](=O)[O-])Cl)/s3
c1ccc(cc1)CN(Cc2ccccc2)S(=O)(=O)c3ccc4c5c3cccc5C(=O)S4
Cc1ccc(cc1)CN2C(=O)/C(=C/c3cc(c(c(c3)I)OCC#C)I)/SC2=O
COc1cc(ccc1OCc2cccc3c2cccc3)/C=c/4\c(=O)n5c6ccccc6nc5s4
Cc1c(c2cc(ccc2o1)N(C(=O)C3CCCCC3)S(=O)(=O)c4ccc(cc4)Cl)C(=O)C
Cc1ccc(cc1)CN2C(=O)/C(=C\c3c4ccccc4ccc3OCc5ccccc5)/SC2=O
COc1cc(cc(c1OCc2ccc(cc2Cl)Cl)I)/C=c/3\c(=O)n4c5ccccc5nc4s3
CCOC(=O)c1c(oc2c1cc(cc2)NS(=O)(=O)c3ccc4c(c3)C(=O)c5ccccc5C4=O)C
Cc1ccc(cc1)CN2C(=O)/C(=C/c3ccc(c(c3)OC)OCC(=O)Nc4ccccc4C)/SC2=O
COc1cc(cc(c1OCc2ccccc2)CC=C)/C=C\3/C(=O)N(C(=O)S3)Cc4c(cccc4Cl)F

```
CC(C)c1ccc(cc1)S(=O)(=O)Nc2ccc3c4c2C(=O)c5ccccc5-c4cc(=O)n3C
CCOC(=O)c1c(oc2c1cc(cc2)N(C(=O)C3CCCCC3)S(=O)(=O)c4ccc(cc4)OC)C
COc1cc(cc(c1OCc2ccccc2Cl)I)/C=c/3\c(=O)n4c5ccccc5nc4s3
Cc1c(c2cc(ccc2o1)NS(=O)(=O)c3ccc4c(c3)C(=O)c5ccccc5C4=O)C(=O)OC
c1ccc2c(c1)cccc2COc3cccc(c3)/C=c/4\c(=O)n5c6ccccc6nc5s4
CCOc1cc(cc(c1OCc2ccccc2Cl)Br)/C=C/3\C(=O)N(C(=O)S3)Cc4c(cccc4Cl)F
CCCCCOC(=O)c1c(oc2c1cc(cc2)NS(=O)(=O)c3ccc(cc3)Cl)C
COc1cc(cc(c1OCc2ccccc2Cl)Br)/C=c/3\c(=O)n4c5ccccc5nc4s3
CCCCCOC(=O)c1c(oc2c1cc(c3c2cccc3)NS(=O)(=O)c4ccc(cc4)OC)C
COc1cc(ccc1OCc2ccccc2Cl)/C=c/3\c(=O)n4c5ccccc5nc4s3
COc1cc(cc(c1OCc2ccc(cc2)Cl)I)/C=C/3\C(=O)N(C(=O)S3)Cc4c(cccc4Cl)F
CC1=C([C@@H](C(=C(N1)SCc2cccc(c2)[N+](=O)[O-])C#N)c3ccc(cc3)Cl)C(=O)C
CCCc1c(c2cc(ccc2o1)NS(=O)(=O)c3cc(ccc3Cl)Cl)C(=O)OCC
CCOc1cc(cc(c1OCc2cccc3c2cccc3)Br)/C=C/4\C(=O)N(C(=O)S4)Cc5c(cccc5Cl)F
CCCc1c(c2cc(ccc2o1)NS(=O)(=O)c3ccc4ccccc4c3)C(=O)OCC
CCCc1c(c2cc(c3ccccc3c2o1)NS(=O)(=O)c4ccc5ccccc5c4)C(=O)OCC
Cc1cccc(c1)NC(=O)COc2ccc(cc2OC)/C=C/3\C(=O)N(C(=O)S3)Cc4c(cccc4Cl)F
CC1=C([C@@H](C(=C(N1)SCC(=O)OCc2ccccc2)C#N)c3ccccc3Cl)C(=O)Nc4ccccc4
Cc1c(c2cc(ccc2o1)NS(=O)(=O)c3cc(ccc3Cl)Cl)C(=O)OCCOC
CC1=C([C@H](C(=C(N1)SCC(=O)OCc2ccccc2)C#N)c3ccccc3Cl)C(=O)Nc4ccccc4
COc1cc(cc(c1OCc2ccc(cc2)F)Br)/C=C/3\C(=O)N(C(=O)S3)Cc4cc5c(cc4Cl)OCO5
CCOC(=O)c1c2cc(ccc2oc1c3ccccc3)NS(=O)(=O)c4ccc(cc4)OC
C[C@@H]1CCc2c(c3cc(c4ccccc4c3o2)NS(=O)(=O)c5ccc(cc5)C(C)(C)C)C1
CC1=C([C@@H](C(=C(N1)SCC(=O)c2ccc(cc2)Cl)C#N)c3ccccc3Cl)C(=O)C
C[C@H]1CCc2c(c3cc(c4ccccc4c3o2)NS(=O)(=O)c5ccc(cc5)C(C)(C)C)C1
COC(=O)c1ccc(cc1)NC2=CC(=NS(=O)(=O)c3ccc(cc3)F)c4ccccc4C2=O
Cc1ccc(cc1)S(=O)(=O)Nc2ccc3c4c2C(=O)c5ccccc5-c4cc(=O)n3C
CC1=C([C@H](C(=C(N1)SCC(=O)c2ccc(cc2)Cl)C#N)c3ccccc3Cl)C(=O)C
CCOC(=O)c1c2cc(c3ccccc3c2oc1c4ccccc4)NS(=O)(=O)c5cccs5
Cc1cc(c(cc1C)S(=O)(=O)Nc2cc3c4c(oc3c5c2cccc5)CCCC4=O)C
Cc1cc(c(cc1C)S(=O)(=O)Nc2cc3c4c(oc3c5c2cccc5)CC(CC4=O)(C)C)C
```

```
COc1cc(cc(c1OCc2ccc(cc2)Cl)Br)/C=C/3\C(=O)N(C(=O)S3)Cc4cc5c(cc4Cl)OCO5
Cc1cc(c(cc1C)S(=O)(=O)Nc2cc3c4c(oc3c5c2cccc5)CC[C@@H](C4)C(C)(C)C)C
Cc1cc(c(cc1C)S(=O)(=O)Nc2cc3c4c(oc3c5c2cccc5)CC[C@H](C4)C(C)(C)C)C
CCOc1cc(c(cc1OC)/C=c/2\c(=O)n3c4ccccc4nc3s2)Br
Cc1c(c2cc(ccc2o1)NS(=O)(=O)c3ccc(cc3)OC)C(=O)OCc4ccccc4
Cc1c(c2cc(c3ccccc3c2o1)[N-]S(=O)(=O)c4ccc(cc4)F)C(=O)OCc5ccccc5
c1cc2c(cc(c3c2c(c1)C(=O)S3)S(=O)(=O)N4CCOCC4)S(=O)(=O)N5CCOCC5
CCOc1ccc(cc1)S(=O)(=O)Nc2cc3c(c(oc3c4c2cccc4)C(C)(C)C)C(=O)OC
Cc1ccc2c(c1C)nc3n2c(=O)/c(=C\c4cn(nc4c5ccccc5)Cc6ccc(cc6Cl)Cl)/s3
CC(C)(C)c1c(c2cc(c3ccccc3c2o1)NS(=O)(=O)c4ccc(cc4)F)C(=O)OC
COc1cc(ccc1OCc2cc3c(cc2Cl)OCO3)/C=c\4/c(=O)n5c6ccccc6nc5s4
Cc1cc(c(cc1C)S(=O)(=O)N(c2ccc3c(c2)cc(o3)C)C(=O)c4ccccc4)C
CCCC(=O)N(c1ccc2c(c1)c(c(o2)C)C(=O)C)S(=O)(=O)c3ccc(cc3C)F
CCCC(=O)N(c1ccc2c(c1)c(c(o2)C)C(=O)OCC)S(=O)(=O)c3ccc(cc3C)F
CCOC(=O)c1c(oc2c1cc(cc2)N(C(=O)C)S(=O)(=O)c3ccc(cc3C)F)C
c1ccc(cc1)n2cc(c(n2)c3ccc(cc3)Cl)/C=c\4/c(=O)n5c6ccccc6nc5s4
Cc1c(c2cc(c3ccccc3c2o1)NS(=O)(=O)c4cccc5c4nccc5)C(=O)OCCOC
Cc1cc(ccc1S(=O)(=O)Nc2ccc(cc2)NS(=O)(=O)c3ccc(cc3C)F)F
Cc1cc(ccc1S(=O)(=O)Nc2cc3c(c(oc3c4c2cccc4)C)C(=O)OCCOC)F
Cc1ccc(s1)[C@@H]2C3=C(CC(CC3=O)(C)C)NC(=C2C(=O)OCCc4ccccc4)C
Cc1ccc(c(c1)N(CC(=O)NCCc2ccc(c(c2)OC)OC)S(=O)(=O)C)OC
Cc1ccc(s1)[C@H]2C3=C(CC(CC3=O)(C)C)NC(=C2C(=O)OCCc4ccccc4)C
CCOc1ccc(cc1)NC(=O)CN(c2ccc(c(c2)Cl)OC)S(=O)(=O)c3ccc(cc3)C
Cc1cc(ccc1S(=O)(=O)Nc2cc3c(c(oc3c4c2cccc4)C)C(=O)OC)F
CC[C@@H](C)OC(=O)C1[C@H](C2=C(CC(CC2=O)(C)C)N=C1C)c3ccc(c(c3)OCC)OC(=O)C
Cc1cc(ccc1S(=O)(=O)Nc2cc(c(c3c2cccc3)O)SCC(=O)[O-])F
Cc1ccc(c(c1)C)Sc2cc(c3ccccc3c2O)NS(=O)(=O)c4ccccc4
CC[C@H](C)OC(=O)C1[C@H](C2=C(CC(CC2=O)(C)C)N=C1C)c3ccc(c(c3)OCC)OC(=O)C
Cc1ccc(cc1)S(=O)(=O)Nc2cc(c(c3c2cccc3)O)Sc4ccc(cc4C)C
c1ccc(cc1)S(=O)(=O)Nc2cc(c(c3c2cccc3)O)Sc4ccc(cc4)Cl
CC[C@@H](C)OC(=O)C1[C@@H](C2=C(CC(CC2=O)(C)C)N=C1C)c3ccc(c(c3)OCC)OC(=O)C
```

```
Cc1cc(cc(c1C)S(=O)(=O)Nc2ccc(c(c2)Sc3nc4ccccc4s3)O)C(C)(C)C
CC[C@H](C)OC(=O)C1[C@@H](C2=C(CC(CC2=O)(C)C)N=C1C)c3ccc(c(c3)OCC)OC(=O)C
CCOC(=O)c1c2cc(ccc2oc1c3ccccc3)NS(=O)(=O)c4c(c(cc(c4C)C)C)C
COc1ccc(cc1)N(C(=O)c2ccc(cc2)Cl)S(=O)(=O)c3cccc4c3nccc4
COc1ccc(cc1)N(C(=O)c2ccccc2Cl)S(=O)(=O)c3cccc4c3nccc4
Cc1c(c2cc(c3ccccc3c2o1)NS(=O)(=O)c4ccc(cc4)C(=O)[O-])C(=O)OC
Cc1c(c2cc(ccc2o1)NS(=O)(=O)c3ccc(cc3)C(=O)[O-])C(=O)OCc4ccccc4
CCOC(=O)c1c(oc2c1cc(c3c2cccc3)NS(=O)(=O)c4ccc(cc4)C(=O)[O-])C
CCC(C)(C)[C@@H]1CCc2c(c3cc(ccc3o2)NS(=O)(=O)c4ccc(cc4)C(=O)[O-])C1
CCC(C)(C)[C@H]1CCc2c(c3cc(ccc3o2)NS(=O)(=O)c4ccc(cc4)C(=O)[O-])C1
Cc1c(c2cc(c3ccccc3c2o1)NS(=O)(=O)c4ccc(cc4)C(=O)[O-])C(=O)OCCOC
CCCc1c(c2cc(c3ccccc3c2o1)NS(=O)(=O)c4ccc(cc4)C(=O)[O-])C(=O)OCC
c1cc(cc(c1)S(=O)(=O)Nc2cc(c(c(c2)Br)O)Br)C(=O)[O-]
CCCOC(=O)C1[C@H](C2=C(CCCC2=O)N=C1C)c3cc(ccc3OC)Br
CC(=O)Oc1c2ccccc2c(cc1SCC(=O)[O-])NS(=O)(=O)c3ccc(cc3)F
CCCOC(=O)C1[C@@H](C2=C(CCCC2=O)N=C1C)c3cc(ccc3OC)Br
CC(=O)N(c1ccc(cc1)OC(=O)C)S(=O)(=O)c2cc(ccc2Br)Br
CCCCc1ccc(cc1)NC2=C/C(=N/c3ccc(cc3)CCCC)/c4ccccc4C2=O
CC(C)Sc1c2c(cc(n1)c3cc4c(c(n3)SC(C)C)CCCC4)CCCC2
c1ccc(cc1)n2c(nnn2)Sc3cc(c4ccccc4c3O)NS(=O)(=O)c5ccccc5
CC1=CC(=O)O[C@H]2[C@H]1C=CC(=C2)OCCCCCCCCOC3=C[C@H]4[C@H](C=C3)C(=CC(=O)O4)C
CC(C)(C)c1ccc(cc1)S(=O)(=O)Nc2cc(c(c3c2cccc3)O)SCc4ccco4
CC1=CC(=O)O[C@H]2[C@H]1C=CC(=C2)OCCCCCCCCOC3=C[C@@H]4[C@H](C=C3)C(=CC(=O)O4)C
CCN1c2ccc(c3c2c(ccc3)C1=O)S(=O)(=O)N[C@@H](Cc4cc5ccccc5[nH]4)C(=O)[O-]
CC1=CC(=O)O[C@H]2[C@H]1C=CC(=C2)OCCCCCCCCOC3=C[C@H]4[C@@H](C=C3)C(=CC(=O)O4)C
CCN1c2ccc(c3c2c(ccc3)C1=O)S(=O)(=O)N[C@H](Cc4cc5ccccc5[nH]4)C(=O)[O-]
Cc1cc(c(c(c1)C)S(=O)(=O)NCCN(CCO)S(=O)(=O)c2c(cc(cc2C)C)C)C
CC1=CC(=O)O[C@H]2[C@H]1C=CC(=C2)OCCCCCCCCOC3=C[C@@H]4[C@@H](C=C3)C(=CC(=O)O4)C
CC1=CC(=O)O[C@H]2[C@H]1C=CC(=C2)OCCCCCCOC3=C[C@H]4[C@H](C=C3)C(=CC(=O)O4)C
c1cc(cc(c1)N2CCN(CC2)S(=O)(=O)c3ccc4c5c3cccc5C(=O)N4)C(F)(F)F
CC1=CC(=O)O[C@H]2[C@H]1C=CC(=C2)OCCCCCCOC3=C[C@@H]4[C@H](C=C3)C(=CC(=O)O4)C
```

```
c1ccc(c(c1)N2CCN(CC2)S(=O)(=O)c3ccc4c5c3cccc5C(=O)N4)Cl
Cc1c(c(=O)n(n1C)c2ccccc2)Nc3ccc4c5c3C(=O)c6ccccc6-c5cc(=O)n4C
CC1=CC(=O)O[C@H]2[C@H]1C=CC(=C2)OCCCCCCOC3=C[C@H]4[C@@H](C=C3)C(=CC(=O)O4)C
CC1(CC2=C(C(C3=C(O2)CC(CC3=O)(C)C)c4ccc(cc4)OC[C@@H](CN5C(=O)c6ccccc6S5(=O)=O)O)C(=O)C1)C
CC1=CC(=O)O[C@H]2[C@H]1C=CC(=C2)OCCCCCCOC3=C[C@@H]4[C@@H](C=C3)C(=CC(=O)O4)C
CC1(CC2=C(C(C3=C(O2)CC(CC3=O)(C)C)c4ccc(cc4)OC[C@H](CN5C(=O)c6ccccc6S5(=O)=O)O)C(=O)C1)C
CC1=NC2=C([C@H](C1C(=O)OCCc3ccccc3)c4ccc(cc4)C(=O)OC)C(=O)CC(C2)(C)C
CC1=C[C@H]2N(O[C@@H]1[C@@H]3[C@@H](C(=O)N3N2C(=O)OCc4ccccc4)NC(=O)Cc5ccccc5)C(=O)c6ccccc6
CC1=C[C@@H]2N(O[C@H]1[C@@H]3[C@@H](C(=O)N3N2C(=O)OCc4ccccc4)NC(=O)Cc5ccccc5)C(=O)c6ccccc6
CC1=NC2=C([C@@H](C1C(=O)OCCc3ccccc3)c4ccc(cc4)C(=O)OC)C(=O)CC(C2)(C)C
CC1(O[C@@H]2[C@H]3[C@@H]4[C@@H](C(=O)N4N([C@@H]([C@@H]2O1)ON3C(=O)OCc5ccccc5)C(=O)OC(C)(C)C)NC(=O)Cc6ccccc6)C
CC1=NC2=C([C@H](C1C(=O)OCCOC(C)C)c3ccccc3Br)C(=O)CC(C2)(C)C
CC1(O[C@H]2[C@H]3[C@@H]4[C@@H](C(=O)N4N([C@@H]([C@@H]2O1)ON3C(=O)OCc5ccccc5)C(=O)OC(C)(C)C)NC(=O)Cc6ccccc6)C
CC1=C([C@@H](n2c(cc(=O)[nH]c2=S)N1)c3ccccc3[N+](=O)[O-])C(=O)OC[C@@H]4CCCO4
COc1ccccc1OC2=CC(=O)OC2=O
CC1=C([C@H](n2c(cc(=O)[nH]c2=S)N1)c3ccccc3[N+](=O)[O-])C(=O)OC[C@@H]4CCCO4
CC1=NC2=C([C@@H](C1C(=O)OCCOC(C)C)c3ccccc3Br)C(=O)CC(C2)(C)C
CC1=C([C@@H](n2c(cc(=O)[nH]c2=S)N1)c3ccccc3[N+](=O)[O-])C(=O)OC[C@H]4CCCO4
Cn1c(c(c(=O)n(c1=O)C)C(=C(C#N)C#N)C#N)/N=C/N(C)C
CC1=C([C@H](n2c(cc(=O)[nH]c2=S)N1)c3ccccc3[N+](=O)[O-])C(=O)OC[C@H]4CCCO4
COc1ccc(cc1)c2cc(=O)c3c(cc(c(c3o2)c4cc(ccc4OC)c5cc(=O)c6c(o5)cc(cc6OC)OC)OC)OC
CCOc1cccc(c1)[C@@H]2C3=C(CCCC3=O)NC(=C2C(=O)OCCc4ccccc4)C
CC1=C([C@@H](c2c([nH]c(nc2=O)N)N1)c3cccc(c3)[N+](=O)[O-])C(=O)OC[C@@H]4CCCO4
CC1=C([C@H](c2c([nH]c(nc2=O)N)N1)c3cccc(c3)[N+](=O)[O-])C(=O)OC[C@@H]4CCCO4
CCOc1cccc(c1)[C@H]2C3=C(CCCC3=O)NC(=C2C(=O)OCCc4ccccc4)C
CC1=C([C@@H](c2c([nH]c(nc2=O)N)N1)c3cccc(c3)[N+](=O)[O-])C(=O)OC[C@H]4CCCO4
CC1=C([C@H](c2c([nH]c(nc2=O)N)N1)c3cccc(c3)[N+](=O)[O-])C(=O)OC[C@H]4CCCO4
Cc1ccccc1[C@@H]2C3=C(CC(CC3=O)(C)C)NC(=C2C(=O)OCc4ccc(cc4)OC)C
CC1(c2ccccc2N(C3=C(C(=C(C#N)C#N)N[C@@H]31)C#N)C)C
c1ccc2c(c1)n(c3[n+]2nc-4c(c3)-c5cccc6c5c4ccc6)Cc7ccc(cc7)C(=O)[O-]
CC1(c2ccccc2N(C3=C(C(=C(C#N)C#N)N[C@H]31)C#N)C)C
```

Cc1ccccc1[C@H]2C3=C(CC(CC3=O)(C)C)NC(=C2C(=O)OCc4ccc(cc4)OC)C
c1ccc2c(c1)n(c3[n+]2nc-4c(c3)-c5cccc6c5c4ccc6)CCNC(=O)CCCC(=O)NCCn7c8ccccc8[n+]9c7cc-1c(n9)-c2cccc3c2c1ccc3
COc1cc(cc(c1)OC)C(=O)NCCNC(=O)Cn2c3ccccc3[n+]4c2cc-5c(n4)-c6cccc7c6c5ccc7
CC1=NC(=C([C@H](C1C(=O)OCCOC)c2ccccc2[N+](=O)[O-])C(=O)OCCSC(C)C)C
CC1=NC(=C([C@@H](C1C(=O)OCCOC)c2ccccc2[N+](=O)[O-])C(=O)OCCSC(C)C)C
CC1=NC(=C([C@H](C1C(=O)OCCOC)c2ccccc2[N+](=O)[O-])C(=O)OCCSC)C
CC1=NC(=C([C@@H](C1C(=O)OCCOC)c2ccccc2[N+](=O)[O-])C(=O)OCCSC)C
CCOc1ccccc1[C@@H]2C3=C(CC(CC3=O)(C)C)NC(=C2C(=O)OCCOc4ccccc4)C
CC1=NC(=C([C@H](C1C(=O)OCCSC)c2cccc(c2)[N+](=O)[O-])C(=O)OCC=C)C
CC1=NC(=C([C@@H](C1C(=O)OCCSC)c2cccc(c2)[N+](=O)[O-])C(=O)OCC=C)C
CC1=NC(=C([C@@H](C1C(=O)OCCSC)c2ccccc2[N+](=O)[O-])C(=O)OCCSC)C
CCOc1ccccc1[C@H]2C3=C(CC(CC3=O)(C)C)NC(=C2C(=O)OCCOc4ccccc4)C
CC1=NC(=C([C@H](C1C(=O)OCCSC)c2cccc(c2)[N+](=O)[O-])C(=O)OCCSC)C
CCSCCOC(=O)C1[C@H](C(=C(N=C1C)C)C(=O)OCC=C)c2cccc(c2)[N+](=O)[O-]
Cc1ccc2c(c1)c(=O)c(co2)[C@@H]3C4=C(CC(CC4=O)(C)C)NC(=C3C(=O)OC(C)C)C
CCSCCOC(=O)C1[C@@H](C(=C(N=C1C)C)C(=O)OCC=C)c2cccc(c2)[N+](=O)[O-]
CC1=NC(=C([C@H](C1C(=O)OC)c2cccc(c2)[N+](=O)[O-])C(=O)OCCOc3ccc(cc3)NC(=O)C)C
Cc1ccc2c(c1)c(=O)c(co2)[C@H]3C4=C(CC(CC4=O)(C)C)NC(=C3C(=O)OC(C)C)C
CC1=NC(=C([C@@H](C1C(=O)OC)c2cccc(c2)[N+](=O)[O-])C(=O)OCCOc3ccc(cc3)NC(=O)C)C
CC1=NC(=C([C@H](C1C(=O)OCC=C)c2cccc(c2)[N+](=O)[O-])C(=O)OC[C@@H]3CCCO3)C
CC1=NC(=C([C@@H](C1C(=O)OCC=C)c2cccc(c2)[N+](=O)[O-])C(=O)OC[C@@H]3CCCO3)C
CC1=NC2=C([C@H](C1C(=O)OCCc3ccccc3)c4cccc(c4)F)C(=O)CC(C2)(C)C
CC1=NC(=C([C@H](C1C(=O)OCC=C)c2cccc(c2)[N+](=O)[O-])C(=O)OC[C@H]3CCCO3)C
CC1=NC(=C([C@@H](C1C(=O)OCC=C)c2cccc(c2)[N+](=O)[O-])C(=O)OC[C@H]3CCCO3)C
CC1=NC2=C([C@@H](C1C(=O)OCCc3ccccc3)c4cccc(c4)F)C(=O)CC(C2)(C)C
CC1=NC(=C([C@H](C1C(=O)OCCSC)c2cccc(c2)[N+](=O)[O-])C(=O)OC(C)C)C
CC1=NC(=C([C@@H](C1C(=O)OCCSC)c2cccc(c2)[N+](=O)[O-])C(=O)OC(C)C)C
CCOC(=O)C1[C@H](C(=C(N=C1C)C)C(=O)OCCNC(=O)c2cccnc2)c3cccc(c3)[N+](=O)[O-]
CC1=NC2=C([C@H](C1C(=O)OCCOc3ccccc3)c4cccc(c4)Br)C(=O)CCC2
CCOC(=O)C1[C@@H](C(=C(N=C1C)C)C(=O)OCCNC(=O)c2cccnc2)c3cccc(c3)[N+](=O)[O-]
CC1=NC(=C([C@H](C1C(=O)OCCOC)c2ccccc2[N+](=O)[O-])C(=O)OCCOc3ccc(cc3)NC(=O)C)C

```
CC1=NC(=C([C@@H](C1C(=O)OCCOC)c2ccccc2[N+](=O)[O-])C(=O)OCCOc3ccc(cc3)NC(=O)C)C
CC1=NC(=C([C@H](C1C(=O)OCCOC)c2cccc(c2)[N+](=O)[O-])C(=O)OCCOc3ccc(cc3)NC(=O)C)C
CC1=NC2=C([C@@H](C1C(=O)OCCOc3ccccc3)c4cccc(c4)Br)C(=O)CCC2
CC1=NC(=C([C@@H](C1C(=O)OCCOC)c2cccc(c2)[N+](=O)[O-])C(=O)OCCOc3ccc(cc3)NC(=O)C)C
CC1=NC(=C([C@H](C1C(=O)OC)c2cccc(c2)[N+](=O)[O-])C(=O)OCC[NH+]3CCN(CC3)C(=O)c4ccco4)C
CCOc1cccc(c1)[C@@H]2C3=C(CC(CC3=O)(C)C)NC(=C2C(=O)OCCOc4ccccc4)C
CC1=NC(=C([C@H](C1C(=O)OCCOc2ccc(cc2)NC(=O)C)c3cccc(c3)[N+](=O)[O-])C(=O)OCCOc4ccc(cc4)NC(=O)C)C
CC1=NC(=C([C@H](C1C(=O)OCCOc2ccc(cc2)NC(=O)C)c3ccccc3[N+](=O)[O-])C(=O)OC(C)C)C
CCOc1cccc(c1)[C@H]2C3=C(CC(CC3=O)(C)C)NC(=C2C(=O)OCCOc4ccccc4)C
CC1=NC(=C([C@@H](C1C(=O)OCCOc2ccc(cc2)NC(=O)C)c3ccccc3[N+](=O)[O-])C(=O)OC(C)C)C
CCOC(=O)C1[C@H](C(=C(N=C1C)C)C(=O)OCCOc2ccc(cc2)NC(=O)C)c3ccccc3[N+](=O)[O-]
Cc1cccc(c1)[C@@H]2C3=C(CC(CC3=O)(C)C)NC(=C2C(=O)OCCOc4ccccc4)C
COc1ccc(cc1)N2C(=O)[C@H]3[C@@H]([NH2+][C@@]4([C@@H]3C2=O)c5ccccc5N(C4=O)Cc6ccccc6F)CCSC
CCOC(=O)C1[C@@H](C(=C(N=C1C)C)C(=O)OCCOc2ccc(cc2)NC(=O)C)c3ccccc3[N+](=O)[O-]
CCOC(=O)C1[C@H](C(=C(N=C1C)C)C(=O)OCCNC(=O)c2ccccc2O)c3cccc(c3)[N+](=O)[O-]
Cc1cccc(c1)[C@H]2C3=C(CC(CC3=O)(C)C)NC(=C2C(=O)OCCOc4ccccc4)C
COc1ccc(cc1)N2C(=O)[C@H]3[C@H]([NH2+][C@@]4([C@@H]3C2=O)c5ccccc5N(C4=O)Cc6ccccc6F)CCSC
CCOC(=O)C1[C@@H](C(=C(N=C1C)C)C(=O)OCCNC(=O)c2ccccc2O)c3cccc(c3)[N+](=O)[O-]
CC1=NC(=C([C@H](C1C(=O)OCCNC(=O)c2ccccc2O)c3cccc(c3)[N+](=O)[O-])C(=O)OCCOc4ccc(cc4)NC(=O)C)C
CC1=NC2=C([C@H](C1C(=O)OCCOC(C)C)c3cc(ccc3OC)Br)C(=O)CCC2
CC1=NC(=C([C@@H](C1C(=O)OCCNC(=O)c2ccccc2O)c3cccc(c3)[N+](=O)[O-])C(=O)OCCOc4ccc(cc4)NC(=O)C)C
CC1=NC(=C([C@H](C1C(=O)OC)c2ccc(s2)[N+](=O)[O-])C(=O)OCCOc3ccc(cc3)NC(=O)C)C
CC1=NC(=C([C@@H](C1C(=O)OC)c2ccc(s2)[N+](=O)[O-])C(=O)OCCOc3ccc(cc3)NC(=O)C)C
Cc1c(c(n(n1)c2ccccc2)O)C(c3ccc(o3)c4ccc(cc4)Br)c5c(nn(c5O)c6ccccc6)C
CCOC(=O)C1[C@H](C(=C(N=C1C)C)C(=O)OCC[NH+]2CCN(CC2)S(=O)(=O)c3ccccc3)c4cccc(c4)[N+](=O)[O-]
CC1=NC2=C([C@@H](C1C(=O)OCCOC(C)C)c3cc(ccc3OC)Br)C(=O)CCC2
CCOC(=O)C1[C@@H](C(=C(N=C1C)C)C(=O)OCC[NH+]2CCN(CC2)S(=O)(=O)c3ccccc3)c4cccc(c4)[N+](=O)[O-]
CC1=NC(=C([C@H](C1C(=O)OCCNC(=O)c2ccccc2O)c3cccc(c3)[N+](=O)[O-])C(=O)OC(C)C)C
CC1=NC2=C([C@H](C1C(=O)OC)c3cccc(c3)OCc4ccccc4)C(=O)C[C@H](C2)c5cccs5
CC1=NC(=C([C@@H](C1C(=O)OCCNC(=O)c2ccccc2O)c3cccc(c3)[N+](=O)[O-])C(=O)OC(C)C)C
CCOC(=O)C1[C@H](C(=C(N=C1C)C)C(=O)OCCNC(=O)c2ccccc2O)c3ccc(s3)[N+](=O)[O-]
```

```
CCOC(=O)C1[C@@H](C(=C(N=C1C)C)C(=O)OCCNC(=O)c2ccccc2O)c3ccc(s3)[N+](=O)[O-]
CC[C@@H]1[C@H]2[C@@H](C(=O)N(C2=O)c3cccc(c3)Cl)[C@@]4([NH2+]1)c5ccccc5N(C4=O)Cc6cccc7c6cccc7
CC1=NC2=C([C@@H](C1C(=O)OC)c3cccc(c3)OCc4ccccc4)C(=O)C[C@H](C2)c5cccs5
CC1=NC(=C([C@H](C1C(=O)OCCOc2ccc(cc2)NC(=O)C)c3cccc(c3)[N+](=O)[O-])C(=O)OC[C@@H]4CCCO4)C
CC[C@H]1[C@H]2[C@@H](C(=O)N(C2=O)c3cccc(c3)Cl)[C@@]4([NH2+]1)c5ccccc5N(C4=O)Cc6cccc7c6cccc7
CC1=NC(=C([C@@H](C1C(=O)OCCOc2ccc(cc2)NC(=O)C)c3cccc(c3)[N+](=O)[O-])C(=O)OC[C@@H]4CCCO4)C
CC1=NC2=C([C@H](C1C(=O)OC)c3cccc(c3)OCc4ccccc4)C(=O)C[C@@H](C2)c5cccs5
CC1=NC(=C([C@H](C1C(=O)OCCOc2ccc(cc2)NC(=O)C)c3cccc(c3)[N+](=O)[O-])C(=O)OC[C@H]4CCCO4)C
CC1=NC(=C([C@@H](C1C(=O)OCCOc2ccc(cc2)NC(=O)C)c3cccc(c3)[N+](=O)[O-])C(=O)OC[C@H]4CCCO4)C
CC1=NC(=C([C@H](C1C(=O)OC)c2cccc(c2)[N+](=O)[O-])C(=O)OCCNC(=O)c3ccc(cc3)O)C
CC1=NC2=C([C@@H](C1C(=O)OC)c3cccc(c3)OCc4ccccc4)C(=O)C[C@@H](C2)c5cccs5
CC1=NC(=C([C@@H](C1C(=O)OC)c2cccc(c2)[N+](=O)[O-])C(=O)OCCNC(=O)c3ccc(cc3)O)C
CC1=NC(=C([C@H](C1C(=O)OCCOC(=O)c2ccc(cc2)NC(=O)C)c3cccc(c3)[N+](=O)[O-])C(=O)OCCOC(=O)c4ccc(cc4)NC(=O)C)C
CC1=NC(=C([C@H](C1C(=O)OCCNC(=O)c2ccccc2O)c3cccc(c3)[N+](=O)[O-])C(=O)OCCNC(=O)c4ccccc4O)C
CC1=NC(=C([C@H](C1C(=O)OCCNC(=O)c2cccnc2)c3cccc(c3)[N+](=O)[O-])C(=O)OCCNC(=O)c4ccccc4O)C
CC1=NC2=C([C@H](C1C(=O)OCc3ccccc3)c4ccccc4Br)C(=O)CCC2
CC1=NC(=C([C@@H](C1C(=O)OCCNC(=O)c2cccnc2)c3cccc(c3)[N+](=O)[O-])C(=O)OCCNC(=O)c4ccccc4O)C
CC1=NC(=C([C@H](C1C(=O)OCC[NH+](C)Cc2ccccc2)c3cccc(c3)[N+](=O)[O-])C(=O)OCCOC(=O)c4ccc(cc4)NC(=O)C)C
CC1=NC(=C([C@@H](C1C(=O)OCC[NH+](C)Cc2ccccc2)c3cccc(c3)[N+](=O)[O-])C(=O)OCCOC(=O)c4ccc(cc4)NC(=O)C)C
CC1=NC2=C([C@@H](C1C(=O)OCc3ccccc3)c4ccccc4Br)C(=O)CCC2
CC1=NC(=C([C@H](C1C(=O)OCCOC(=O)c2ccc(cc2)NC(=O)C)c3cccc(c3)[N+](=O)[O-])C(=O)OC[C@@H]4CCCO4)C
CSCC[C@@H]1[C@H]2[C@@H](C(=O)N(C2=O)Cc3ccccc3)[C@@]4([NH2+]1)c5ccccc5N(C4=O)Cc6cccc7c6cccc7
CC1=NC(=C([C@@H](C1C(=O)OCCOC(=O)c2ccc(cc2)NC(=O)C)c3cccc(c3)[N+](=O)[O-])C(=O)OC[C@@H]4CCCO4)C
CSCC[C@H]1[C@H]2[C@@H](C(=O)N(C2=O)Cc3ccccc3)[C@@]4([NH2+]1)c5ccccc5N(C4=O)Cc6cccc7c6cccc7
CCOc1cccc(c1)[C@@H]2C3=C(CC(CC3=O)(C)C)NC(=C2C(=O)OCc4ccccc4)C
CC1=NC(=C([C@H](C1C(=O)OCCOC(=O)c2ccc(cc2)NC(=O)C)c3cccc(c3)[N+](=O)[O-])C(=O)OC[C@H]4CCCO4)C
CC1=NC(=C([C@@H](C1C(=O)OCCOC(=O)c2ccc(cc2)NC(=O)C)c3cccc(c3)[N+](=O)[O-])C(=O)OC[C@H]4CCCO4)C
CCOc1cccc(c1)[C@H]2C3=C(CC(CC3=O)(C)C)NC(=C2C(=O)OCc4ccccc4)C
CC1=NC(=C([C@H](C1C(=O)OC)c2cccc(c2)[N+](=O)[O-])C(=O)OCCOC(=O)c3ccc(cc3)NC(=O)C)C
CC1=NC(=C([C@@H](C1C(=O)OC)c2cccc(c2)[N+](=O)[O-])C(=O)OCCOC(=O)c3ccc(cc3)NC(=O)C)C
CC1=NC(=C([C@@H](C1C(=O)OCCNC(=O)c2ccc(cc2)O)c3cccc(c3)[N+](=O)[O-])C(=O)OCCNC(=O)c4ccccc4O)C
```

```
CC1=NC(=C([C@H](C1C(=O)OCCNC(=O)c2ccccc2O)c3cccc(c3)[N+](=O)[O-])C(=O)OCCOC(=O)c4ccc(cc4)NC(=O)C)C
CC1=NC(=C([C@@H](C1C(=O)OCCNC(=O)c2ccccc2O)c3cccc(c3)[N+](=O)[O-])C(=O)OCCOC(=O)c4ccc(cc4)NC(=O)C)C
Cc1ccc(cc1)S(=O)(=O)N2CC[NH+](CC2)CCOC(=O)C3[C@H](C(=C(N=C3C)C)C(=O)OC)c4cccc(c4)[N+](=O)[O-]
Cc1ccccc1NC(=O)C2=C(NC(=C([C@@H]2c3ccco3)C#N)SCC(=O)Nc4ccc(cc4)Cl)C
Cc1ccc(cc1)NC(=O)CN2c3ccccc3[C@@]4(C2=O)[C@H]5[C@@H]([C@@H]([NH2+]4)CCSC)C(=O)N(C5=O)Cc6ccccc6
Cc1ccc(cc1)S(=O)(=O)N2CC[NH+](CC2)CCOC(=O)C3[C@@H](C(=C(N=C3C)C)C(=O)OC)c4cccc(c4)[N+](=O)[O-]
Cc1ccc(cc1)NC(=O)CN2c3ccccc3[C@]4(C2=O)[C@H]5[C@@H]([C@@H]([NH2+]4)CCSC)C(=O)N(C5=O)Cc6ccccc6
CC1=NC(=C([C@@H](C1C(=O)OCCOc2ccc(cc2)NC(=O)C)c3cccc(c3)[N+](=O)[O-])C(=O)OCCOC(=O)c4ccc(cc4)NC(=O)C)C
CCCc1c(nc(s1)N(c2ccc(cc2)OC)C(=O)C3CCCC3)c4ccc(cc4)OC
Cc1ccccc1NC(=O)C2=C(NC(=C([C@H]2c3ccco3)C#N)SCC(=O)Nc4ccc(cc4)Cl)C
CCOC(=O)C1[C@H](C(=C(N=C1C)C)C(=O)OCC[NH+]2CCN(CC2)S(=O)(=O)c3ccc(cc3)OC)c4cccc(c4)[N+](=O)[O-]
CCOC(=O)C1[C@@H](C(=C(N=C1C)C)C(=O)OCC[NH+]2CCN(CC2)S(=O)(=O)c3ccc(cc3)OC)c4cccc(c4)[N+](=O)[O-]
CC[C@@H]1[C@H]2[C@@H](C(=O)N(C2=O)Cc3ccccc3)[C@@]4([NH2+]1)c5ccccc5N(C4=O)Cc6ccccc6F
CC1=NC(=C([C@H](C1C(=O)OC)c2cccc(c2)[N+](=O)[O-])C(=O)OCC[NH+]3CC[NH+](CC3)C)C
CC[C@H]1[C@H]2[C@@H](C(=O)N(C2=O)Cc3ccccc3)[C@@]4([NH2+]1)c5ccccc5N(C4=O)Cc6ccccc6F
Cc1ccc(cc1)C(=O)CSC2=NC(=C([C@H](C2C#N)c3ccco3)C(=O)Nc4ccccc4C)C
CC1=NC(=C([C@@H](C1C(=O)OC)c2cccc(c2)[N+](=O)[O-])C(=O)OCC[NH+]3CC[NH+](CC3)C)C
CCOC(=O)C1[C@H](C(=C(N=C1C)C)C(=O)OCC[NH+]2CC[NH+](CC2)C)c3cccc(c3)[N+](=O)[O-]
Cc1ccc(cc1)C(=O)CSC2=NC(=C([C@@H](C2C#N)c3ccco3)C(=O)Nc4ccccc4C)C
CSCC[C@@H]1[C@H]2[C@@H](C(=O)N(C2=O)Cc3ccccc3)[C@@]4([NH2+]1)c5ccccc5N(C4=O)CC=C
CCOC(=O)C1[C@@H](C(=C(N=C1C)C)C(=O)OCC[NH+]2CC[NH+](CC2)C)c3cccc(c3)[N+](=O)[O-]
CCOC(=O)C1[C@H](C(=C(N=C1C)C)C(=O)OCC[NH+]2CCN(CC2)S(=O)(=O)c3cccc(c3)Cl)c4cccc(c4)[N+](=O)[O-]
CSCC[C@H]1[C@H]2[C@@H](C(=O)N(C2=O)Cc3ccccc3)[C@@]4([NH2+]1)c5ccccc5N(C4=O)CC=C
CCOC(=O)C1[C@@H](C(=C(N=C1C)C)C(=O)OCC[NH+]2CCN(CC2)S(=O)(=O)c3cccc(c3)Cl)c4cccc(c4)[N+](=O)[O-]
CC1=NC(=C([C@H](C1C(=O)OCCNC(=O)c2cccnc2)c3cccc(c3)[N+](=O)[O-])C(=O)OCC[NH+](C)C)C
CC1=NC(=C([C@@H](C1C(=O)OCCNC(=O)c2cccnc2)c3cccc(c3)[N+](=O)[O-])C(=O)OCC[NH+](C)C)C
CC1=NC(=C([C@H](C1C(=O)OCCOc2ccc(cc2)NC(=O)C)c3cccc(c3)[N+](=O)[O-])C(=O)OC/C=C/c4ccccc4)C
CC1=NC(=C([C@@H](C1C(=O)OCCOc2ccc(cc2)NC(=O)C)c3cccc(c3)[N+](=O)[O-])C(=O)OC/C=C/c4ccccc4)C
CC1=NC2=C([C@H](C1C(=O)OC)c3ccc(cc3)C(F)(F)F)C(=O)C[C@H](C2)c4cccs4
CC1=NC(=C([C@H](C1C(=O)OCCNC(=O)c2ccccc2O)c3ccccc3[N+](=O)[O-])C(=O)OC[C@@H]4CCCO4)C
CC1=NC(=C([C@@H](C1C(=O)OCCNC(=O)c2ccccc2O)c3ccccc3[N+](=O)[O-])C(=O)OC[C@@H]4CCCO4)C
```

```
CC1=NC(=C([C@H](C1C(=O)OCCNC(=O)c2ccccc2O)c3ccccc3[N+](=O)[O-])C(=O)OC[C@H]4CCCO4)C
CC1=NC2=C([C@@H](C1C(=O)OC)c3ccc(cc3)C(F)(F)F)C(=O)C[C@H](C2)c4cccs4
CC[C@@H]1[C@H]2[C@@H](C(=O)N(C2=O)c3ccc(cc3)Cl)[C@@]4([NH2+]1)c5ccccc5N(C4=O)CC=C
CC1=NC(=C([C@@H](C1C(=O)OCCNC(=O)c2ccccc2O)c3ccccc3[N+](=O)[O-])C(=O)OC[C@H]4CCCO4)C
CC[C@H]1[C@H]2[C@@H](C(=O)N(C2=O)c3ccc(cc3)Cl)[C@@]4([NH2+]1)c5ccccc5N(C4=O)CC=C
CC1=NC(=C([C@H](C1C(=O)OCCNC(=O)c2cccnc2)c3ccccc3[N+](=O)[O-])C(=O)OCCNC(=O)c4ccccc4O)C
CC1=NC2=C([C@H](C1C(=O)OC)c3ccc(cc3)C(F)(F)F)C(=O)C[C@@H](C2)c4cccs4
CC1=NC(=C([C@@H](C1C(=O)OCCNC(=O)c2cccnc2)c3ccccc3[N+](=O)[O-])C(=O)OCCNC(=O)c4ccccc4O)C
CC1=NC(=C([C@H](C1C(=O)OCCNC(=O)c2ccccc2O)c3ccccc3[N+](=O)[O-])C(=O)OCC[NH+](C)C)C
CC1=NC(=C([C@@H](C1C(=O)OCCNC(=O)c2ccccc2O)c3ccccc3[N+](=O)[O-])C(=O)OCC[NH+](C)C)C
CC1=NC2=C([C@@H](C1C(=O)OC)c3ccc(cc3)C(F)(F)F)C(=O)C[C@@H](C2)c4cccs4
CC1=NC(=C([C@@H](C1C(=O)OCCNC(=O)c2ccccc2O)c3cccc(c3)[N+](=O)[O-])C(=O)OC/C=C/c4ccccc4)C
CC1=NC(=C([C@H](C1C(=O)OCCOC(=O)c2ccc(cc2)NC(=O)C)c3ccccc3[N+](=O)[O-])C(=O)OC[C@@H]4CCCO4)C
CC1=NC(=C([C@@H](C1C(=O)OCCOC(=O)c2ccc(cc2)NC(=O)C)c3ccccc3[N+](=O)[O-])C(=O)OC[C@@H]4CCCO4)C
CC1=NC(=C([C@@H](C1C(=O)OCCOC(=O)c2ccc(cc2)NC(=O)C)c3ccccc3[N+](=O)[O-])C(=O)OC[C@H]4CCCO4)C
CC1=NC(=C([C@H](C1C(=O)OC)c2ccccc2[N+](=O)[O-])C(=O)OCCNC(=O)c3cccnc3)C
CC1=NC(=C([C@@H](C1C(=O)OC)c2ccccc2[N+](=O)[O-])C(=O)OCCNC(=O)c3cccnc3)C
CC1=NC(=C([C@H](C1C(=O)OC)c2cccc(c2)[N+](=O)[O-])C(=O)OCC[NH+]3CCN(CC3)S(=O)(=O)c4ccccc4Cl)C
CCCOCCOC(=O)C1[C@H](C2=C(CC(CC2=O)(C)C)N=C1C)c3ccc(cc3)N(CC)CC
CC1=NC(=C([C@@H](C1C(=O)OC)c2cccc(c2)[N+](=O)[O-])C(=O)OCC[NH+]3CCN(CC3)S(=O)(=O)c4ccccc4Cl)C
CCCOCCOC(=O)C1[C@@H](C2=C(CC(CC2=O)(C)C)N=C1C)c3ccc(cc3)N(CC)CC
CC1=NC(=C([C@H](C1C(=O)OC)c2ccccc2[N+](=O)[O-])C(=O)OCC[NH+]3CCN(CC3)S(=O)(=O)c4ccc(cc4)Cl)C
CC1=NC(=C([C@@H](C1C(=O)OC)c2ccccc2[N+](=O)[O-])C(=O)OCC[NH+]3CCN(CC3)S(=O)(=O)c4ccc(cc4)Cl)C
CC1=NC2=C([C@H](C1C(=O)OCCc3ccccc3)c4ccc(cc4)OC(=O)C)C(=O)CC(C2)(C)C
CC1=NC(=C([C@H](C1C(=O)OC)c2ccc(c(c2)[N+](=O)[O-])Cl)C(=O)OC[C@@H]3CCCO3)C
CC1=NC2=C([C@@H](C1C(=O)OCCc3ccccc3)c4ccc(cc4)OC(=O)C)C(=O)CC(C2)(C)C
CC1=NC(=C([C@@H](C1C(=O)OC)c2ccc(c(c2)[N+](=O)[O-])Cl)C(=O)OC[C@@H]3CCCO3)C
CC1=NC(=C([C@H](C1C(=O)OC)c2ccc(c(c2)[N+](=O)[O-])Cl)C(=O)OC[C@H]3CCCO3)C
CC1=NC(=C([C@@H](C1C(=O)OC)c2ccc(c(c2)[N+](=O)[O-])Cl)C(=O)OC[C@H]3CCCO3)C
CCCOCCOC(=O)C1[C@H](C2=C(CCCC2=O)N=C1C)c3cccc(c3)Br
CC1=NC(=C([C@H](C1C(=O)OC)c2ccccc2C(F)(F)F)C(=O)OC[C@@H]3CCCO3)C
```

```
CC1=NC(=C([C@@H](C1C(=O)OC)c2ccccc2C(F)(F)F)C(=O)OC[C@@H]3CCCO3)C
CC1=NC(=C([C@H](C1C(=O)OC)c2ccccc2C(F)(F)F)C(=O)OC[C@H]3CCCO3)C
CCCOCCOC(=O)C1[C@@H](C2=C(CCCC2=O)N=C1C)c3cccc(c3)Br
CC1=NC(=C([C@@H](C1C(=O)OC)c2ccccc2C(F)(F)F)C(=O)OC[C@H]3CCCO3)C
CC(C)C[C@@H]1[C@H]2[C@@H](C(=O)N(C2=O)c3ccccc3)[C@@]4([NH2+]1)c5ccccc5N(C4=O)CC=C
Cc1cc2c(cc1C)[nH]c3c2c[n+](c4c3cccc4)CC(=O)NCCCCCCCCNC(=O)C[n+]5cc6c7cc(c(cc7[nH]c6c8c5cccc8)C)C
CC(C)C[C@H]1[C@H]2[C@@H](C(=O)N(C2=O)c3ccccc3)[C@@]4([NH2+]1)c5ccccc5N(C4=O)CC=C
CCCOCCOC(=O)C1[C@H](C2=C(CC(CC2=O)(C)C)N=C1C)c3ccc(c(c3)F)F
CC1=C([C@@H](n2cnc(c2N1)C(=O)N)c3ccccc3[N+](=O)[O-])C(=O)OC[C@@H]4CCCO4
CCCOCCOC(=O)C1[C@@H](C2=C(CC(CC2=O)(C)C)N=C1C)c3ccc(c(c3)F)F
CC1=C([C@H](n2cnc(c2N1)C(=O)N)c3ccccc3[N+](=O)[O-])C(=O)OC[C@@H]4CCCO4
CC1=C([C@@H](n2cnc(c2N1)C(=O)N)c3ccccc3[N+](=O)[O-])C(=O)OC[C@H]4CCCO4
CCCOCCOC(=O)C1[C@H](C2=C(CC(CC2=O)(C)C)N=C1C)c3ccc(cc3)Br
CC1=C([C@H](n2cnc(c2N1)C(=O)N)c3ccccc3[N+](=O)[O-])C(=O)OC[C@H]4CCCO4
c1ccc(cc1)c2csc3[n+]2cc(c(c3)c4cccc(c4)[N+](=O)[O-])c5cccc(c5)[N+](=O)[O-]
c1ccc2c(c1)[n+]3cc(c(cc3s2)c4cccc(c4)[N+](=O)[O-])c5cccc(c5)[N+](=O)[O-]
CCCOCCOC(=O)C1[C@@H](C2=C(CC(CC2=O)(C)C)N=C1C)c3ccc(cc3)Br
CCCOCCOC(=O)C1[C@H](C2=C(CC(CC2=O)(C)C)N=C1C)c3ccc(c(c3)Cl)Cl
CCCCc1nc2ccccc2n1Cc3ccc(cc3)c4ccccc4c5nnn(n5)C(c6ccccc6)(c7ccccc7)c8ccccc8
c1ccc(cc1)C(c2ccccc2)(c3ccccc3)n4nc(nn4)c5ccccc5c6ccc(cc6)Cn7cnc8c7cc(c(c8)Cl)Cl
CCCOCCOC(=O)C1[C@@H](C2=C(CC(CC2=O)(C)C)N=C1C)c3ccc(c(c3)Cl)Cl
CCc1cc2n(c3ccccc3[n+]2nc1CC)CCCC#CC#CCCCn4c5ccccc5[n+]6c4cc(c(n6)CC)CC
CCCOCCOC(=O)C1[C@H](C2=C(CCCC2=O)N=C1C)c3ccccc3OCc4ccccc4
c1ccc2c(c1)n(c3[n+]2nc-4c(c3)-c5cccc6c5c4ccc6)CCCC#CC#CCCCn7c8ccccc8[n+]9c7cc-1c(n9)-c2cccc3c2c1ccc3
CC1=NC(=C([C@H](C1C(=O)OC)c2cccc(c2)Br)C(=O)OC[C@@H]3CCCO3)C
CCCOCCOC(=O)C1[C@@H](C2=C(CCCC2=O)N=C1C)c3ccccc3OCc4ccccc4
CC1=NC(=C([C@@H](C1C(=O)OC)c2cccc(c2)Br)C(=O)OC[C@@H]3CCCO3)C
CC1=NC(=C([C@H](C1C(=O)OC)c2cccc(c2)Br)C(=O)OC[C@H]3CCCO3)C
CCCOCCOC(=O)C1[C@H](C2=C(CC(CC2=O)(C)C)N=C1C)c3cc4c(cc3Br)OCO4
CC1=NC(=C([C@@H](C1C(=O)OC)c2cccc(c2)Br)C(=O)OC[C@H]3CCCO3)C
CCCOCCOC(=O)C1[C@@H](C2=C(CC(CC2=O)(C)C)N=C1C)c3cc4c(cc3Br)OCO4
```

```
CCCOCCOC(=O)C1[C@H](C2=C(CC(CC2=O)(C)C)N=C1C)c3ccccc3Cl
CCC(C)(C)c1ccc(c(c1)C(C)(C)CC)OCC(=O)Nc2cccc(c2)c3cn4cccc(c4n3)C
CCCOCCOC(=O)C1[C@@H](C2=C(CC(CC2=O)(C)C)N=C1C)c3ccccc3Cl
CC1=NC2=C([C@H](C1C(=O)OCCOC)c3cc(c(cc3Br)OC)OC)C(=O)CCC2
c1ccc(cc1)c2ccc(cc2)S(=O)(=O)Nc3cccc(c3)c4cn5ccsc5n4
CC1=NC2=C([C@@H](C1C(=O)OCCOC)c3cc(c(cc3Br)OC)OC)C(=O)CCC2
CCCCOc1ccc(cc1)S(=O)(=O)Nc2cccc(c2)c3cn4ccsc4n3
CCCOCCOC(=O)C1[C@H](C2=C(CC(CC2=O)(C)C)N=C1C)c3cccc(c3OC)OC
c1ccc(cc1)c2c(n3ccccc3n2)N(S(=O)(=O)c4ccc(cc4)Br)S(=O)(=O)c5ccc(cc5)Br
CCCOCCOC(=O)C1[C@@H](C2=C(CC(CC2=O)(C)C)N=C1C)c3cccc(c3OC)OC
c1ccc(cc1)c2ccc(cc2)S(=O)(=O)N(c3c(nc4n3cccc4)c5ccccc5)S(=O)(=O)c6ccc(cc6)c7ccccc7
CCC(C)(C)c1ccc(c(c1)C(C)(C)CC)OCC(=O)Nc2cccc(c2)c3csc(n3)C
CC[C@@H]1[C@H]2[C@@H](C(=O)N(C2=O)c3ccc(cc3)Br)[C@@]4([NH2+]1)c5ccccc5N(C4=O)CC=C
Cn1c2c(c(=O)n(c1=O)C)n(c(n2)NN=Cc3ccc(cc3OC)OC)CCO
CCC(C)(C)c1ccc(c(c1)C(C)(C)CC)OCC(=O)Nc2cccc(c2)c3cn4ccccc4n3
CC[C@H]1[C@H]2[C@@H](C(=O)N(C2=O)c3ccc(cc3)Br)[C@@]4([NH2+]1)c5ccccc5N(C4=O)CC=C
CC(C)(C)c1ccc(cc1)S(=O)(=O)N2CCS[C@@H]2c3cccc(c3)OCc4ccccc4
CC(C)(C)c1ccc(cc1)S(=O)(=O)N2CCS[C@H]2c3cccc(c3)OCc4ccccc4
Cc1ccc(cc1)NC(=O)CN2c3ccccc3[C@@]4(C2=O)[C@H]5[C@@H]([C@@H]([NH2+]4)CC(C)C)C(=O)N(C5=O)c6ccccc6
Cc1ccc(cc1)NC(=O)CN2c3ccccc3[C@]4(C2=O)[C@H]5[C@@H]([C@@H]([NH2+]4)CC(C)C)C(=O)N(C5=O)c6ccccc6
c1ccc(cc1)c2ccc(cc2)S(=O)(=O)Nc3cccc(c3)c4cn5cccnc5n4
CC1=NC2=C([C@H](C1C(=O)OC)c3ccc(c(c3)OC)OC(=O)C)C(=O)C[C@H](C2)c4cccs4
Cc1ccc(cc1)NC(=O)CN2c3ccccc3[C@@]4(C2=O)[C@H]5[C@@H]([C@@H]([NH2+]4)C)C(=O)N(C5=O)c6ccc(cc6)Br
C[C@@H](c1ccccc1)N2[C@H]([C@H](C2=O)N3[C@@H]4C[C@H]5CC[C@@]4(C5(C)C)CS3(=O)=O)/C=C/c6ccccc6
Cc1ccc(cc1)NC(=O)CN2c3ccccc3[C@]4(C2=O)[C@H]5[C@@H]([C@@H]([NH2+]4)C)C(=O)N(C5=O)c6ccc(cc6)Br
CC1=NC2=C([C@@H](C1C(=O)OC)c3ccc(c(c3)OC)OC(=O)C)C(=O)C[C@H](C2)c4cccs4
CC1([C@@H]2CC[C@]13CS(=O)(=O)N([C@@H]3C2)[C@@H]4[C@@H](N(C4=O)Cc5ccccc5)/C=C/c6ccccc6)C
Cc1c(c(n(n1)c2ccccc2)O)C(c3ccc(o3)c4ccc(cc4Cl)Cl)c5c(nn(c5O)c6ccccc6)C
CC1=NC2=C([C@H](C1C(=O)OC)c3ccc(c(c3)OC)OC(=O)C)C(=O)C[C@@H](C2)c4cccs4
CC1=NC2=C([C@@H](C1C(=O)OC)c3ccc(c(c3)OC)OC(=O)C)C(=O)C[C@@H](C2)c4cccs4
c1ccc(cc1)C(CC(=O)Nc2ccc(cc2)NC(=O)C34CC5CC(C3)CC(C5)C4)c6ccccc6
```

```
Cc1ccc(cc1)S(=O)(=O)N(c2cccc(c2)C)[C@@H]3[C@@H](C(=O)N3c4cccc(c4)C)Oc5ccccc5
Cc1ccc(cc1)S(=O)(=O)N(c2cccc(c2)C)[C@H]3[C@@H](C(=O)N3c4cccc(c4)C)Oc5ccccc5
Cc1ccc(cc1)S(=O)(=O)N(c2ccccc2)[C@@H]3[C@@H](C(=O)N3c4ccccc4)Oc5ccccc5
CCCOCCOC(=O)C1[C@H](C2=C(CC(CC2=O)(C)C)N=C1C)c3ccc(cc3)C(=O)OC
Cc1ccc(cc1)S(=O)(=O)N(c2ccccc2)[C@H]3[C@@H](C(=O)N3c4ccccc4)Oc5ccccc5
CCCOCCOC(=O)C1[C@@H](C2=C(CC(CC2=O)(C)C)N=C1C)c3ccc(cc3)C(=O)OC
COc1ccc(cc1)N2[C@@H]([C@@H](C2=O)Oc3ccccc3)N(c4ccc(cc4)OC)C(=O)COc5ccccc5
CCCCCCCCCCCCCCNS(=O)(=O)c1ccc-2c(c1)Cc3c2cccc3
COc1ccc(cc1)N2[C@H]([C@@H](C2=O)Oc3ccccc3)N(c4ccc(cc4)OC)C(=O)COc5ccccc5
Cc1ccc(s1)[C@@H]2C(C(=NC(=C2C(=O)Nc3ccccc3OC)C)SCC(=O)Nc4ccccc4)C#N
COc1ccc(cc1)N2[C@@H]([C@H](C2=O)Oc3ccccc3)N(c4ccc(cc4)OC)C(=O)COc5ccccc5
COc1ccc(cc1)N2[C@H]([C@H](C2=O)Oc3ccccc3)N(c4ccc(cc4)OC)C(=O)COc5ccccc5
Cc1ccc(s1)[C@H]2C(C(=NC(=C2C(=O)Nc3ccccc3OC)C)SCC(=O)Nc4ccccc4)C#N
Cc1cccc(c1)N2[C@@H]([C@@H](C2=O)Oc3ccccc3)N(c4cccc(c4)C)C(=O)COc5ccccc5
Cc1ccc(s1)[C@@H]2C(C(=NC(=C2C(=O)Nc3ccccc3OC)C)SCC(=O)Nc4ccc(cc4)Br)C#N
Cc1cccc(c1)N2[C@H]([C@@H](C2=O)Oc3ccccc3)N(c4cccc(c4)C)C(=O)COc5ccccc5
Cc1ccc(s1)[C@H]2C(C(=NC(=C2C(=O)Nc3ccccc3OC)C)SCC(=O)Nc4ccc(cc4)Br)C#N
Cc1cccc(c1)N2[C@@H]([C@H](C2=O)Oc3ccccc3)N(c4cccc(c4)C)C(=O)COc5ccccc5
Cn1c2c(c(=O)n(c1=O)C)n(c(n2)Oc3ccc(cc3C4CCCC4)Cl)Cc5cccc(c5)Cl
Cc1cccc(c1)N2[C@H]([C@H](C2=O)Oc3ccccc3)N(c4cccc(c4)C)C(=O)COc5ccccc5
CC1=N[C@H]([NH2+][C@H](C1C(=O)C)c2ccc(c(c2)Cl)Cl)c3ccc(c(c3)Cl)Cl
CC1=N[C@H]([NH2+][C@@H](C1C(=O)C)c2ccc(c(c2)Cl)Cl)c3ccc(c(c3)Cl)Cl
CC1(OC[C@@H](O1)[C@@H]2[C@@H]([C@@H]3[C@@H](O2)OC(O3)(C)C)N4[C@@H]([C@@H](C4=O)Oc5ccccc5)/C=C/c6ccccc6)C
CCCCCCCCCCOc1ccc(cc1)c2nnc(n2CC=C)SCC(=O)Nc3ccccc3C
CC1=N[C@@H]([NH2+][C@H](C1C(=O)C)c2ccc(c(c2)Cl)Cl)c3ccc(c(c3)Cl)Cl
CC1(OC[C@H](O1)[C@@H]2[C@@H]([C@@H]3[C@@H](O2)OC(O3)(C)C)N4[C@@H]([C@@H](C4=O)Oc5ccccc5)/C=C/c6ccccc6)C
CC1=N[C@@H]([NH2+][C@@H](C1C(=O)C)c2ccc(c(c2)Cl)Cl)c3ccc(c(c3)Cl)Cl
CC1(OC[C@@H](O1)[C@H]2[C@@H]([C@@H]3[C@@H](O2)OC(O3)(C)C)N4[C@@H]([C@@H](C4=O)Oc5ccccc5)/C=C/c6ccccc6)C
CC1(OC[C@H](O1)[C@H]2[C@@H]([C@@H]3[C@@H](O2)OC(O3)(C)C)N4[C@@H]([C@@H](C4=O)Oc5ccccc5)/C=C/c6ccccc6)C
CCOC(=O)[C@@H]1C[NH+](CC[C@@]12c3c(c4ccccc4[nH]3)CCN2C(=O)C(c5ccccc5)c6ccccc6)Cc7ccccc7
CCOc1cc(ccc1O)[C@H]2C3=C(C[C@@H](CC3=O)c4cccs4)NC(=C2C(=O)OCC)C
```

```
CCOc1cc(ccc1O)[C@@H]2C3=C(C[C@H](CC3=O)c4cccs4)NC(=C2C(=O)OCC)C
CCOc1cc(ccc1O)[C@H]2C3=C(C[C@H](CC3=O)c4cccs4)NC(=C2C(=O)OCC)C
c1cc(ccc1C(=O)[C@@H](CSc2ccc(cc2)Cl)n3cncn3)Br
c1cc(ccc1C(=O)[C@H](CSc2ccc(cc2)Cl)n3cncn3)Br
CCO[P@@](=S)(NC(=S)NNc1nc2ccccc2o1)Oc3ccccc3Cl
CCO[P@](=S)(NC(=S)NNc1nc2ccccc2o1)Oc3ccccc3Cl
CCCOP(=S)(OCCC)SCCCSc1nn2c(nnc2s1)c3ccccc3
CCCCOc1ccc(cc1)c2c3c([nH]n2)OC(=C([C@@]34c5ccccc5N(C4=O)CC(=O)OC)C#N)N
CCCCOc1ccc(cc1)c2c3c([nH]n2)OC(=C([C@]34c5ccccc5N(C4=O)CC(=O)OC)C#N)N
CCOc1cc(cc(c1[O-])[N+](=O)[O-])[C@@H]2c3c(n[nH]c3OC(=C2C#N)N)c4ccccc4
CCOc1cc(cc(c1[O-])[N+](=O)[O-])[C@H]2c3c(n[nH]c3OC(=C2C#N)N)c4ccccc4
CCOc1ccc(cc1)c2c3c([nH]n2)OC(=C([C@@]34c5ccccc5N(C4=O)CC(=O)[O-])C#N)N
CCOc1ccc(cc1)c2c3c([nH]n2)OC(=C([C@]34c5ccccc5N(C4=O)CC(=O)[O-])C#N)N
Cc1ccc(cc1)S[C@@H](c2ccccc2)[C@@H](Cn3cncn3)C(=O)c4ccc(cc4)Br
Cc1ccc(cc1)S[C@H](c2ccccc2)[C@@H](Cn3cncn3)C(=O)c4ccc(cc4)Br
Cc1ccc(cc1)S[C@@H](c2ccccc2)[C@H](Cn3cncn3)C(=O)c4ccc(cc4)Br
Cc1ccc(cc1)S[C@H](c2ccccc2)[C@H](Cn3cncn3)C(=O)c4ccc(cc4)Br
CC1=NC2=C([C@H](C1C(=O)OCC(C)C)c3cccc(c3)[N+](=O)[O-])C(=O)C[C@H](C2)c4cccs4
CC1=NC2=C([C@@H](C1C(=O)OCC(C)C)c3cccc(c3)[N+](=O)[O-])C(=O)C[C@H](C2)c4cccs4
Cc1ccc(cc1)S(=O)(=O)Oc2cc(ccc2[C@@]34CCCC[C@@H]3C5(CCCC5)c6ccc(cc6O4)OC)OC
Cc1ccc(cc1)S(=O)(=O)Oc2cc(ccc2[C@]34CCCC[C@@H]3C5(CCCC5)c6ccc(cc6O4)OC)OC
Cc1ccc(cc1)S(=O)(=O)Oc2cc(ccc2[C@@]34CCCC[C@H]3C5(CCCC5)c6ccc(cc6O4)OC)OC
CC1=NC2=C([C@H](C1C(=O)OCC(C)C)c3cccc(c3)[N+](=O)[O-])C(=O)C[C@@H](C2)c4cccs4
Cc1ccc(cc1)S(=O)(=O)Oc2cc(ccc2[C@]34CCCC[C@H]3C5(CCCC5)c6ccc(cc6O4)OC)OC
CC1=NC2=C([C@@H](C1C(=O)OCC(C)C)c3cccc(c3)[N+](=O)[O-])C(=O)C[C@@H](C2)c4cccs4
CC1=NC2=C([C@H](C1C(=O)OCC(C)C)c3cc(c(c(c3)OC)[O-])[N+](=O)[O-])C(=O)C[C@H](C2)c4cccs4
CC1=NC2=C([C@@H](C1C(=O)OCC(C)C)c3cc(c(c(c3)OC)[O-])[N+](=O)[O-])C(=O)C[C@H](C2)c4cccs4
CC1=NC2=C([C@H](C1C(=O)OCC(C)C)c3cc(c(c(c3)OC)[O-])[N+](=O)[O-])C(=O)C[C@@H](C2)c4cccs4
CC1=NC2=C([C@@H](C1C(=O)OCC(C)C)c3cc(c(c(c3)OC)[O-])[N+](=O)[O-])C(=O)C[C@@H](C2)c4cccs4
Cc1cc(nc2c1cccc2)SCCC(=O)Nc3ccc(cc3)c4nc5ccccc5s4
CCSCCOC(=O)C1[C@H](C2=C(C[C@H](CC2=O)c3cccs3)N=C1C)c4cc(c(c(c4)OC)[O-])[N+](=O)[O-]
```

```
CCSCCOC(=O)C1[C@@H](C2=C(C[C@H](CC2=O)c3cccs3)N=C1C)c4cc(c(c(c4)OC)[O-])[N+](=O)[O-]
COc1ccc(cc1)c2csc(n2)N3CC[NH+](CC3)C(c4ccccc4)c5ccccc5
CCSCCOC(=O)C1[C@H](C2=C(C[C@@H](CC2=O)c3cccs3)N=C1C)c4cc(c(c(c4)OC)[O-])[N+](=O)[O-]
Cc1nc(cs1)CSc2nc3c(c(cs3)c4ccc(cc4)Cl)c(=O)n2CC=C
CCSCCOC(=O)C1[C@@H](C2=C(C[C@@H](CC2=O)c3cccs3)N=C1C)c4cc(c(c(c4)OC)[O-])[N+](=O)[O-]
CC1=NC2=C([C@H](C1C(=O)OC3CCCC3)c4cccc(c4)F)C(=O)C[C@H](C2)c5cccs5
CC1=NC2=C([C@@H](C1C(=O)OC3CCCC3)c4cccc(c4)F)C(=O)C[C@H](C2)c5cccs5
CC(=O)c1cccc(c1)NC(=O)CSc2nc3c(c4c(s3)CCCC4)c(=O)n2CC=C
CC1=NC2=C([C@H](C1C(=O)OC3CCCC3)c4cccc(c4)F)C(=O)C[C@@H](C2)c5cccs5
CC1=NC2=C([C@@H](C1C(=O)OC3CCCC3)c4cccc(c4)F)C(=O)C[C@@H](C2)c5cccs5
CC(C)Oc1ccccc1NC(=O)c2ccccc2OCC(=O)NCCOc3ccccc3
CC1=NC2=C([C@H](C1C(=O)OC[C@@H]3CCCO3)c4ccc(c(c4OC)OC)OC)C(=O)C[C@H](C2)c5cccs5
COc1ccc(cc1)OCCNC(=O)c2ccccc2OCC(=O)NCCc3ccccc3
CC1=NC2=C([C@@H](C1C(=O)OC[C@@H]3CCCO3)c4ccc(c(c4OC)OC)OC)C(=O)C[C@H](C2)c5cccs5
c1ccc(cc1)OCCNC(=O)c2ccccc2NC(=O)[C@@H]3CCCN3S(=O)(=O)c4ccccc4
CC1=NC2=C([C@H](C1C(=O)OC[C@@H]3CCCO3)c4ccc(c(c4OC)OC)OC)C(=O)C[C@@H](C2)c5cccs5
c1ccc(cc1)OCCNC(=O)c2ccccc2NC(=O)[C@H]3CCCN3S(=O)(=O)c4ccccc4
CC(=O)Nc1ccc(cc1)NC(=O)COc2ccccc2C(=O)NCCOc3ccc(cc3)OC
CC1=NC2=C([C@@H](C1C(=O)OC[C@@H]3CCCO3)c4ccc(c(c4OC)OC)OC)C(=O)C[C@@H](C2)c5cccs5
CCOCCOC(=O)C1[C@H](C2=C(C[C@H](CC2=O)c3cccs3)N=C1C)c4ccsc4
CCOCCOC(=O)C1[C@@H](C2=C(C[C@H](CC2=O)c3cccs3)N=C1C)c4ccsc4
CCOCCOC(=O)C1[C@H](C2=C(C[C@@H](CC2=O)c3cccs3)N=C1C)c4ccsc4
CCOCCOC(=O)C1[C@@H](C2=C(C[C@@H](CC2=O)c3cccs3)N=C1C)c4ccsc4
CCOC(=O)C1[C@H](C2=C(C[C@H](CC2=O)c3cccs3)N=C1C)c4cc(ccc4F)Br
CCOC(=O)C1[C@@H](C2=C(C[C@H](CC2=O)c3cccs3)N=C1C)c4cc(ccc4F)Br
CCOC(=O)C1[C@H](C2=C(C[C@@H](CC2=O)c3cccs3)N=C1C)c4cc(ccc4F)Br
CCOC(=O)C1[C@@H](C2=C(C[C@@H](CC2=O)c3cccs3)N=C1C)c4cc(ccc4F)Br
CCOC(=O)C1[C@H](C2=C(C[C@H](CC2=O)c3cccs3)N=C1C)c4coc5ccccc5c4=O
c1ccc(cc1)S(=O)(=O)N(CC(=O)Nc2ccc(cc2)S(=O)(=O)N3CCOCC3)c4ccc(c(c4)C(F)(F)F)Cl
CCOC(=O)C1[C@@H](C2=C(C[C@H](CC2=O)c3cccs3)N=C1C)c4coc5ccccc5c4=O
c1ccc(cc1)S(=O)(=O)N(CC(=O)N/N=C/c2cc3c(cc2Br)OCO3)c4ccc(c(c4)C(F)(F)F)Cl
```

```
CCOC(=O)C1[C@H](C2=C(C[C@@H](CC2=O)c3cccs3)N=C1C)c4coc5ccccc5c4=O
CN(C)c1ccc(cc1Br)/C=N/NC(=O)CN(c2ccc(c(c2)C(F)(F)F)Cl)S(=O)(=O)c3ccccc3
CCOC(=O)C1[C@@H](C2=C(C[C@@H](CC2=O)c3cccs3)N=C1C)c4coc5ccccc5c4=O
C/C(=N\NC(=O)CN(c1ccc(c(c1)C(F)(F)F)Cl)S(=O)(=O)c2ccccc2)/c3ccc4c(c3)OCCO4
COC(=O)COc1ccc(cc1)/C=N/NC(=O)CN(c2ccc(c(c2)C(F)(F)F)Cl)S(=O)(=O)c3ccccc3
CC1=NC2=C([C@H](C1C(=O)OC3CCCC3)c4cccc(c4)OC)C(=O)C[C@H](C2)c5cccs5
CC(C)(C)OC(=O)COc1ccc(cc1)/C=N/NC(=O)CN(c2ccc(c(c2)C(F)(F)F)Cl)S(=O)(=O)c3ccccc3
c1ccc(cc1)S(=O)(=O)N(CC(=O)N/N=C/c2ccc(cc2)OCC(=O)N3CCCCC3)c4ccc(c(c4)C(F)(F)F)Cl
CC1=NC2=C([C@@H](C1C(=O)OC3CCCC3)c4cccc(c4)OC)C(=O)C[C@H](C2)c5cccs5
c1ccc(cc1)S(=O)(=O)N(CC(=O)N/N=C/c2ccc(cc2)OCC(=O)N3CCOCC3)c4ccc(c(c4)C(F)(F)F)Cl
CC1=NC2=C([C@H](C1C(=O)OC3CCCC3)c4cccc(c4)OC)C(=O)C[C@@H](C2)c5cccs5
c1ccc(cc1)S(=O)(=O)N(CC(=O)N/N=C/c2ccc(cc2)OC3CSC3)c4ccc(c(c4)C(F)(F)F)Cl
COc1cc(ccc1OCC=C)/C=N/NC(=O)CN(c2ccc(c(c2)C(F)(F)F)Cl)S(=O)(=O)c3ccccc3
CC1=NC2=C([C@@H](C1C(=O)OC3CCCC3)c4cccc(c4)OC)C(=O)C[C@@H](C2)c5cccs5
C/C(=N\NC(=O)CN(c1ccc(c(c1)C(F)(F)F)Cl)S(=O)(=O)c2ccccc2)/c3ccc(cc3)NC(=O)C
C/C(=N\NC(=O)CN(c1ccc(c(c1)C(F)(F)F)Cl)S(=O)(=O)c2ccccc2)/c3cccc(c3)NC(=O)C
CCOC(=O)c1ccc(cc1)c2ccc(o2)/C=N/NC(=O)CN(c3ccc(c(c3)C(F)(F)F)Cl)S(=O)(=O)c4ccccc4
CC1=NC2=C([C@H](C1C(=O)OCC(C)C)c3ccccc3F)C(=O)C[C@H](C2)c4cccs4
CCOc1ccccc1N(CC(=O)N/N=C/c2ccc3c4c2cccc4CC3)S(=O)(=O)c5ccccc5
CCOc1ccccc1N(CC(=O)N/N=C/c2ccc(cc2)OCC(=O)N3CCCCC3)S(=O)(=O)c4ccccc4
CC1=NC2=C([C@@H](C1C(=O)OCC(C)C)c3ccccc3F)C(=O)C[C@H](C2)c4cccs4
CCOc1ccccc1N(CC(=O)N/N=C/c2ccc(cc2)OCC=C)S(=O)(=O)c3ccccc3
CS(=O)(=O)N(CC(=O)N/N=C/c1ccc(cc1)OCC(=O)N2CCOCC2)c3ccccc3Cl
CC1=NC2=C([C@H](C1C(=O)OCC(C)C)c3ccccc3F)C(=O)C[C@@H](C2)c4cccs4
C/C(=N\NC(=O)CN(c1ccccc1Br)S(=O)(=O)C)/c2ccc3c(c2)OCCO3
C/C(=N\NC(=O)CN(c1ccccc1Br)S(=O)(=O)C)/c2cccs2
C/C(=N\NC(=O)CN(c1ccccc1Br)S(=O)(=O)C)/c2ccc(cc2)NC(=O)C
CC1=NC2=C([C@@H](C1C(=O)OCC(C)C)c3ccccc3F)C(=O)C[C@@H](C2)c4cccs4
C/C(=N\NC(=O)CN(c1ccccc1Br)S(=O)(=O)C)/c2cccc(c2)NC(=O)c3ccccc3
Cc1ccc(cc1)S(=O)(=O)N(CC(=O)N/N=C/c2ccc(cc2)OCC#N)c3ccccc3F
CCSCCOC(=O)C1[C@H](C2=C(C[C@H](CC2=O)c3cccs3)N=C1C)c4cc(c(c(c4)OC)OC)OC
```

```
Cc1ccc(cc1)S(=O)(=O)N(CC(=O)N/N=C(\C)/c2cccc(c2)NC(=O)c3ccccc3)c4ccccc4F
Cc1ccc(cc1)S(=O)(=O)N(CC(=O)N/N=C(\C)/c2ccc3c(c2)OCCO3)c4ccc(c(c4)OC)OC
CCSCCOC(=O)C1[C@@H](C2=C(C[C@H](CC2=O)c3cccs3)N=C1C)c4cc(c(c(c4)OC)OC)OC
Cc1ccc(cc1)S(=O)(=O)N(CC(=O)N/N=C/c2cccc(c2)OCC=C)c3ccc(c(c3)OC)OC
CCSCCOC(=O)C1[C@H](C2=C(C[C@@H](CC2=O)c3cccs3)N=C1C)c4cc(c(c(c4)OC)OC)OC
CCOc1ccc(cc1OCC)/C=N/NC(=O)CN(c2ccc(c(c2)OC)OC)S(=O)(=O)c3ccc(cc3)C
CCSCCOC(=O)C1[C@@H](C2=C(C[C@@H](CC2=O)c3cccs3)N=C1C)c4cc(c(c(c4)OC)OC)OC
CCOCCOC(=O)C1[C@H](C2=C(C[C@H](CC2=O)c3cccs3)N=C1C)c4cccc(c4Cl)Cl
Cc1ccc(cc1)S(=O)(=O)N(CC(=O)N/N=C(\C)/c2cccc(c2)NC(=O)c3cccc(c3)Cl)c4ccc(c(c4)OC)OC
CCOCCOC(=O)C1[C@@H](C2=C(C[C@H](CC2=O)c3cccs3)N=C1C)c4cccc(c4Cl)Cl
CCOCCOC(=O)C1[C@H](C2=C(C[C@@H](CC2=O)c3cccs3)N=C1C)c4cccc(c4Cl)Cl
CCOCCOC(=O)C1[C@@H](C2=C(C[C@@H](CC2=O)c3cccs3)N=C1C)c4cccc(c4Cl)Cl
CC1=NC2=C([C@H](C1C(=O)OCCOC)c3ccc(c(c3OC)OC)OC)C(=O)C[C@H](C2)c4cccs4
CC1=NC2=C([C@@H](C1C(=O)OCCOC)c3ccc(c(c3OC)OC)OC)C(=O)C[C@H](C2)c4cccs4
CC1=NC2=C([C@H](C1C(=O)OCCOC)c3ccc(c(c3OC)OC)OC)C(=O)C[C@@H](C2)c4cccs4
CC1=NC2=C([C@@H](C1C(=O)OCCOC)c3ccc(c(c3OC)OC)OC)C(=O)C[C@@H](C2)c4cccs4
CCSCCOC(=O)C1[C@H](C2=C(C[C@H](CC2=O)c3cccs3)N=C1C)c4cccc(c4)Cl
CCSCCOC(=O)C1[C@@H](C2=C(C[C@H](CC2=O)c3cccs3)N=C1C)c4cccc(c4)Cl
CCSCCOC(=O)C1[C@H](C2=C(C[C@@H](CC2=O)c3cccs3)N=C1C)c4cccc(c4)Cl
CCSCCOC(=O)C1[C@@H](C2=C(C[C@@H](CC2=O)c3cccs3)N=C1C)c4cccc(c4)Cl
CC1=NC2=C([C@H](C1C(=O)OC3CCCC3)c4ccc(cc4)C(F)(F)F)C(=O)C[C@H](C2)c5cccs5
CC1=NC2=C([C@@H](C1C(=O)OC3CCCC3)c4ccc(cc4)C(F)(F)F)C(=O)C[C@H](C2)c5cccs5
CC1=NC2=C([C@H](C1C(=O)OC3CCCC3)c4ccc(cc4)C(F)(F)F)C(=O)C[C@@H](C2)c5cccs5
CC1=NC2=C([C@@H](C1C(=O)OC3CCCC3)c4ccc(cc4)C(F)(F)F)C(=O)C[C@@H](C2)c5cccs5
CC1=NC2=C([C@H](C1C(=O)OC3CCCC3)c4cc(ccc4F)Br)C(=O)C[C@H](C2)c5cccs5
CC1=NC2=C([C@@H](C1C(=O)OC3CCCC3)c4cc(ccc4F)Br)C(=O)C[C@H](C2)c5cccs5
CC1=NC2=C([C@H](C1C(=O)OC3CCCC3)c4cc(ccc4F)Br)C(=O)C[C@@H](C2)c5cccs5
CC1=NC2=C([C@H](C1C(=O)OC3CCCC3)c4cc(c(c(c4)OC)OC)OC)C(=O)C[C@H](C2)c5ccccc5OC
CC1=NC2=C([C@@H](C1C(=O)OC3CCCC3)c4cc(c(c(c4)OC)OC)OC)C(=O)C[C@H](C2)c5ccccc5OC
CC1=NC2=C([C@H](C1C(=O)OC3CCCC3)c4cc(c(c(c4)OC)OC)OC)C(=O)C[C@@H](C2)c5ccccc5OC
CC1=NC2=C([C@@H](C1C(=O)OC3CCCC3)c4cc(c(c(c4)OC)OC)OC)C(=O)C[C@@H](C2)c5ccccc5OC
```

```
CCSCCOC(=O)C1[C@H](C2=C(C[C@H](CC2=O)c3cccs3)N=C1C)c4ccc(cc4)C(F)(F)F
CCSCCOC(=O)C1[C@@H](C2=C(C[C@H](CC2=O)c3cccs3)N=C1C)c4ccc(cc4)C(F)(F)F
CCSCCOC(=O)C1[C@H](C2=C(C[C@@H](CC2=O)c3cccs3)N=C1C)c4ccc(cc4)C(F)(F)F
CCSCCOC(=O)C1[C@@H](C2=C(C[C@@H](CC2=O)c3cccs3)N=C1C)c4ccc(cc4)C(F)(F)F
CCSCCOC(=O)C1[C@H](C2=C(C[C@H](CC2=O)c3cccs3)N=C1C)c4cc(ccc4F)Br
CCSCCOC(=O)C1[C@@H](C2=C(C[C@H](CC2=O)c3cccs3)N=C1C)c4cc(ccc4F)Br
CCSCCOC(=O)C1[C@H](C2=C(C[C@@H](CC2=O)c3cccs3)N=C1C)c4cc(ccc4F)Br
CCSCCOC(=O)C1[C@@H](C2=C(C[C@@H](CC2=O)c3cccs3)N=C1C)c4cc(ccc4F)Br
CC1=NC2=C([C@H](C1C(=O)OC3CCCC3)c4cc(c(cc4OC)OC)OC)C(=O)C[C@H](C2)c5cccs5
CC1=NC2=C([C@@H](C1C(=O)OC3CCCC3)c4cc(c(cc4OC)OC)OC)C(=O)C[C@H](C2)c5cccs5
CC1=NC2=C([C@H](C1C(=O)OC3CCCC3)c4cc(c(cc4OC)OC)OC)C(=O)C[C@@H](C2)c5cccs5
CC1=NC2=C([C@@H](C1C(=O)OC3CCCC3)c4cc(c(cc4OC)OC)OC)C(=O)C[C@@H](C2)c5cccs5
CC1=NC2=C([C@H](C1C(=O)OCC(C)C)c3cc(c(c(c3)OC)OC)OC)C(=O)C[C@H](C2)c4cccs4
CC1=NC2=C([C@@H](C1C(=O)OCC(C)C)c3cc(c(c(c3)OC)OC)OC)C(=O)C[C@H](C2)c4cccs4
CC1=NC2=C([C@H](C1C(=O)OCC(C)C)c3cc(c(c(c3)OC)OC)OC)C(=O)C[C@@H](C2)c4cccs4
CC1=NC2=C([C@@H](C1C(=O)OCC(C)C)c3cc(c(c(c3)OC)OC)OC)C(=O)C[C@@H](C2)c4cccs4
Cn1c2c(c(=O)n(c1=O)C)n3c(n2)N(N=C(C3)c4ccc(cc4)Br)c5ccccc5
Cn1c2c(c(=O)n(c1=O)C)n3c(n2)N(N=C(C3)c4ccc(cc4)Br)CCO
CC1=NC2=C([C@H](C1C(=O)OC)c3ccc(c(c3)Br)OC)C(=O)C[C@H](C2)c4ccccc4OC
CC1=NC2=C([C@@H](C1C(=O)OC)c3ccc(c(c3)Br)OC)C(=O)C[C@H](C2)c4ccccc4OC
CC1=NC2=C([C@H](C1C(=O)OC)c3ccc(c(c3)Br)OC)C(=O)C[C@@H](C2)c4ccccc4OC
CC1=NC2=C([C@@H](C1C(=O)OC)c3ccc(c(c3)Br)OC)C(=O)C[C@@H](C2)c4ccccc4OC
CC1=NC2=C([C@H](C1C(=O)OC(C)C)c3ccc(cc3)OC(=O)C)C(=O)C[C@H](C2)c4ccccc4OC
CC1=NC2=C([C@@H](C1C(=O)OC(C)C)c3ccc(cc3)OC(=O)C)C(=O)C[C@H](C2)c4ccccc4OC
CC1=NC2=C([C@H](C1C(=O)OC(C)C)c3ccc(cc3)OC(=O)C)C(=O)C[C@@H](C2)c4ccccc4OC
CC1=NC2=C([C@@H](C1C(=O)OC(C)C)c3ccc(cc3)OC(=O)C)C(=O)C[C@@H](C2)c4ccccc4OC
CCSCCOC(=O)C1[C@H](C2=C(C[C@H](CC2=O)c3ccccc3OC)N=C1C)c4cc(c(c(c4)OC)OC)OC
CCSCCOC(=O)C1[C@@H](C2=C(C[C@H](CC2=O)c3ccccc3OC)N=C1C)c4cc(c(c(c4)OC)OC)OC
CCSCCOC(=O)C1[C@H](C2=C(C[C@@H](CC2=O)c3ccccc3OC)N=C1C)c4cc(c(c(c4)OC)OC)OC
CCSCCOC(=O)C1[C@@H](C2=C(C[C@@H](CC2=O)c3ccccc3OC)N=C1C)c4cc(c(c(c4)OC)OC)OC
Cn1c2c(nc1N(CC(=O)Nc3ccccc3C(=O)OC)/N=C/c4ccccc4)n(c(=O)n(c2=O)C)C
```

```
CC1(CC2=C([C@H](C3=C(CC(CC3=N2)(C)C)[O-])c4ccc(cc4)OCc5ccc(cc5)Cl)C(=O)C1)C
CCN1c2cc(c(cc2N[C@@H]3[C@@H]1OC(=C3C(=O)OC4CCC(CC4)C(C)(C)C)C)F)N5CCOCC5
CCN1c2cc(c(cc2N[C@H]3[C@@H]1OC(=C3C(=O)OC4CCC(CC4)C(C)(C)C)C)F)N5CCOCC5
CCN1c2cc(c(cc2N[C@@H]3[C@@H]1OC(=C3C(=O)OCc4ccco4)C)F)N5CCOCC5
CCN1c2cc(c(cc2N[C@H]3[C@@H]1OC(=C3C(=O)OCc4ccco4)C)F)N5CCOCC5
CCC/C(=N\CCCCCC/N=C(/C1=C(CCCC1=O)[O-])\CCC)/C2=C(CCCC2=O)[O-]
Cc1ccc(cc1/N=C/2\c3cccc(c3C(=C2C(=O)C)O)[N+](=O)[O-])Nc4ccc(cc4[N+](=O)[O-])[N+](=O)[O-]
Cc1ccc(cc1)OCCSc2nc3ccccc3n2CCOc4ccccc4OC
c1cc(sc1)C2=NN3[C@@H](C2)c4cc(ccc4O[C@@H]3c5cc(ccc5O)Br)Cl
c1cc(sc1)C2=NN3[C@H](C2)c4cc(ccc4O[C@@H]3c5cc(ccc5O)Br)Cl
c1cc(sc1)C2=NN3[C@@H](C2)c4cc(ccc4O[C@@H]3C(=O)c5ccc(cc5)Br)Br
c1cc(sc1)C2=NN3[C@H](C2)c4cc(ccc4O[C@@H]3C(=O)c5ccc(cc5)Br)Br
c1cc(sc1)C2=NN3[C@@H](C2)c4cc(ccc4O[C@@H]3c5ccc(cc5)Cl)Br
c1cc(sc1)C2=NN3[C@H](C2)c4cc(ccc4O[C@@H]3c5ccc(cc5)Cl)Br
c1cc(sc1)C2=NN3[C@@H](C2)c4cc(ccc4O[C@@H]3c5ccc(cc5)Br)Br
c1cc(sc1)C2=NN3[C@H](C2)c4cc(ccc4O[C@@H]3c5ccc(cc5)Br)Br
c1cc(sc1)C2=NN3[C@@H](C2)c4cc(ccc4O[C@@H]3c5ccc(cc5Cl)Cl)Br
c1cc(sc1)C2=NN3[C@H](C2)c4cc(ccc4O[C@@H]3c5ccc(cc5Cl)Cl)Br
c1cc(cc(c1)[N+](=O)[O-])[C@@H]2N3[C@@H](CC(=N3)c4cccs4)c5cc(ccc5O2)Br
c1cc(cc(c1)[N+](=O)[O-])[C@H]2N3[C@@H](CC(=N3)c4cccs4)c5cc(ccc5O2)Br
Cc1c(c2cc(ccc2o1)N(C(=O)c3ccccc3)S(=O)(=O)c4ccc5ccccc5c4)C(=O)C
Cc1c(c2cc(ccc2o1)N(C(=O)Oc3ccccc3)S(=O)(=O)c4ccc5ccccc5c4)C(=O)C
CCOC(=O)c1c(oc2c1cc(cc2)N(C(=O)c3ccncc3)S(=O)(=O)c4ccc(cc4)Cl)C
c1ccc(cc1)c2c3ccccc3nc(n2)N4[C@@H](CC(=N4)c5ccc(cc5)Br)c6ccc(cc6)F
c1ccc(cc1)c2c3ccccc3nc(n2)N4[C@H](CC(=N4)c5ccc(cc5)Br)c6ccc(cc6)F
Cc1c(c2cc(ccc2o1)N(C(=O)c3ccccc3)S(=O)(=O)c4ccccc4)C(=O)C
Cc1c(c2cc(ccc2o1)N(C(=O)c3ccccc3Cl)S(=O)(=O)c4ccccc4)C(=O)C
Cc1c(c2cc(ccc2o1)N(C(=O)/C=C/c3ccccc3)S(=O)(=O)c4ccc(cc4)Cl)C(=O)C
CCc1ccc(cc1)S(=O)(=O)N(c2ccc3c(c2)c(c(o3)C)C(=O)C)C(=O)c4ccc(cc4)Cl
CCc1ccc(cc1)S(=O)(=O)N(c2ccc3c(c2)c(c(o3)C)C(=O)C)C(=O)Oc4ccccc4
CCc1ccc(cc1)S(=O)(=O)N(c2ccc3c(c2)c(c(o3)C)C(=O)C)C(=O)/C=C/c4ccccc4
```

```
CCOC(=O)c1c(oc2c1cc(cc2)N(C(=O)c3ccc(cc3)Cl)S(=O)(=O)c4ccccc4)C
CCOC(=O)c1c(oc2c1cc(cc2)N(C(=O)c3ccccc3Cl)S(=O)(=O)c4ccccc4)C
c1cc(ccc1N2CC[NH+](CC2)C[C@@H](Cn3c4ccc(cc4c5c3ccc(c5)Br)Br)O)Cl
c1cc(ccc1N2CC[NH+](CC2)C[C@H](Cn3c4ccc(cc4c5c3ccc(c5)Br)Br)O)Cl
C[NH+]1CCCC1=C2c3cc(cc(c3-c4c2cc(cc4[N+](=O)[O-])[N+](=O)[O-])[N+](=O)[O-])[N+](=O)[O-]
c1ccc(c(c1)[C@@H]2C(=C(C(=O)N2c3cccc(c3)C(F)(F)F)O)C(=O)c4ccc(cc4)Br)Cl
c1ccc(c(c1)[C@H]2C(=C(C(=O)N2c3cccc(c3)C(F)(F)F)O)C(=O)c4ccc(cc4)Br)Cl
COc1ccccc1[C@@H]2C(=C(C(=O)N2c3cccc(c3)C(F)(F)F)O)C(=O)c4ccc(cc4)Br
COc1ccccc1[C@H]2C(=C(C(=O)N2c3cccc(c3)C(F)(F)F)O)C(=O)c4ccc(cc4)Br
c1ccc(cc1)c2c3cc(ccc3nc(n2)N4[C@@H](CC(=N4)c5ccc(cc5)Br)c6ccc(cc6)Br)Cl
c1ccc(cc1)c2c3cc(ccc3nc(n2)N4[C@H](CC(=N4)c5ccc(cc5)Br)c6ccc(cc6)Br)Cl
COc1ccc(cc1)C2=NN([C@@H](C2)c3ccc(cc3)Br)c4nc5ccccc5c(n4)c6ccccc6
COc1ccc(cc1)C2=NN([C@H](C2)c3ccc(cc3)Br)c4nc5ccccc5c(n4)c6ccccc6
COc1cc(cc(c1OCc2cccc(c2)Br)I)/C=C\3/C(=O)OC(=N3)c4ccc(cc4)F
Cc1ccccc1NC(=O)CSc2c(c(cc(n2)c3ccccc3)c4ccccc4)C#N
COC(=O)c1ccc(cc1)NC(=O)c2c(c3c(cc(nc3s2)c4ccccc4)c5ccccc5)N
Cc1ccc(cc1)c2cc(c(c(n2)SCC(=O)Nc3ccc(c(c3)C)C)C#N)c4ccc(cc4)OC
CCOC(=O)c1c2c(sc1NC(=O)CSc3c(c(cc(n3)c4ccc(cc4)C)c5ccccc5)C#N)CCCC2
CCOc1cc(cc(c1OCC)OCC)C(=O)Nc2ccc(cc2)c3nc4ccccc4s3
c1ccc(cc1)c2cc(nc(c2C#N)SCC(=O)Nc3ccccc3Cl)c4ccccc4
Cc1ccc(cc1)c2cc(c(c(n2)SCC(=O)Nc3ccccc3c4ccccc4)C#N)c5ccc(cc5)OC
CCCOc1ccc(cc1)C(=O)C2=C(C(=O)N([C@@H]2c3ccccc3)Cc4ccccc4)[O-]
CCCOc1ccc(cc1)C(=O)C2=C(C(=O)N([C@H]2c3ccccc3)Cc4ccccc4)[O-]
Cc1ccc(cc1)c2cc(c(c(n2)SCC(=O)Nc3cccc(c3)Cl)C#N)c4ccccc4
Cc1ccc(cc1)c2cc(c3c(c(sc3n2)C(=O)Nc4cc(ccc4Cl)C(F)(F)F)N)c5ccccc5
CC[NH+](CC)CCCN1[C@@H](C(=C(C1=O)O)C(=O)c2ccc(cc2)OCC)c3ccc(cc3)C(C)C
CC[NH+](CC)CCCN1[C@H](C(=C(C1=O)O)C(=O)c2ccc(cc2)OCC)c3ccc(cc3)C(C)C
COc1ccc(cc1)c2cc(nc3c2c(c(s3)C(=O)Nc4ccc(cc4)Br)N)c5ccccc5
Cc1cccn2c1nc3c(c2=O)cc(c([n+]3Cc4ccncc4)N)C(=O)NCc5cccnc5
Cc1cccn2c1nc3c(c2=O)cc(c(=[NH2+])n3Cc4ccncc4)C(=O)NCC[NH+]5CCOCC5
c1ccc(cc1)c2cc(nc3c2c(c(s3)C(=O)Nc4cc(ccc4Cl)C(F)(F)F)N)c5ccccc5
```

```
Cc1c(sc(c1C#N)NC(=O)c2c(c3c(cc(nc3s2)c4ccccc4)c5ccc(cc5)OC)N)C
CC(=C)COc1c(cc(cc1I)/C=C\2/C(=O)OC(=N2)c3ccc4ccccc4c3)I
c1ccc2c(c1)c(=O)n(c(n2)/C=C/c3cccc(c3)C(F)(F)F)c4cccc(c4)C(F)(F)F
Cc1ccc(cc1)C[n+]2c(c(cc3c2nc4ccccn4c3=O)C(=O)NCc5cccnc5)N
COc1cc(cc(c1OCc2cccc(c2)Br)Cl)/C=C\3/C(=O)OC(=N3)c4cc(ccc4Cl)I
Cc1cccc(c1C)O[C@@H](C)C(=O)Nc2nnc(s2)SCc3ccc(cc3Cl)Cl
Cc1cccc(c1C)O[C@H](C)C(=O)Nc2nnc(s2)SCc3ccc(cc3Cl)Cl
c1ccc(cc1)c2cc(nc(c2C#N)SCC(=O)Nc3ccc(cc3)Br)c4ccccc4
Cc1c(sc(c1C#N)NC(=O)CSc2c(c(cc(n2)c3ccccc3)c4ccccc4)C#N)C
CC(C)(C)c1ccc(cc1)OCC(=O)Nc2nnc(s2)SCc3ccc(cc3Cl)Cl
Cc1ccc(cc1)c2cc(c(c(n2)SCC(=O)Nc3ccccc3c4ccccc4)C#N)c5ccccc5
Cc1ccc(cc1)c2cc(c(c(n2)SCC(=O)Nc3ccccc3Cl)C#N)c4ccccc4
Cc1ccc(cc1)c2cc(c3c(c(sc3n2)C(=O)Nc4ccc(cc4)Br)N)c5ccc(cc5)OC
Cc1ccc(c(c1)C)NC(=O)CSc2c(c(cc(n2)c3ccccc3)c4ccc(cc4)C(C)(C)C)C#N
CC(C)(C)c1ccc(cc1)c2cc(nc(c2C#N)SCC(=O)Nc3ccccc3)c4ccccc4
COc1ccc(cc1)c2cc(nc3c2c(c(s3)C(=O)Nc4c(cc(cc4Cl)Cl)Cl)N)c5ccccc5
c1ccc2c(c1)cccc2CC(=O)Nc3nnc(s3)SCc4cccc5c4cccc5
Cc1c2ccccc2oc1C(=O)Nc3nnc(s3)SCc4ccc(cc4Cl)Cl
Cc1ccc(c(c1)C)N2C(=O)c3ccccc3/C(=C\Nc4c(n(n(c4=O)c5ccccc5)C)C)/C2=O
CCCCCCOc1ccc(cc1)C(=O)Nc2nnc(s2)SCc3ccc(cc3Cl)Cl
CCCCCCOc1ccc(cc1)C(=O)Nc2nnc(s2)SCc3ccccc3F
CC1(Cc2c(c3cc(ccc3o2)NS(=O)(=O)c4cc(ccc4Cl)Cl)C(=O)C1)C
Cc1c(c2cc(ccc2o1)N(C(=O)c3ccncc3)S(=O)(=O)c4ccc(cc4)Cl)C(=O)OC
Cc1c(c2cc(ccc2o1)N(C(=O)c3cccc(c3)[N+](=O)[O-])S(=O)(=O)c4ccc(cc4)Cl)C(=O)OC
CC1([C@@H]([C@@H]1C(=O)Nc2nnc(s2)SCc3ccc(cc3)F)C=C(Cl)Cl)C
Cc1ccc(cc1)S(=O)(=O)N(c2ccc3c(c2)c(c(o3)C)C(=O)OC)C(=O)/C=C/c4ccccc4
Cc1ccc(cc1)S(=O)(=O)N(c2ccc3c(c2)c(c(o3)C)C(=O)OC)C(=O)Oc4ccccc4
CCc1ccc(cc1)S(=O)(=O)N(c2ccc3c(c2)c(c(o3)C)C(=O)OC)C(=O)c4ccc(cc4)C
Cc1c(c2cc(ccc2o1)N(C(=O)C)S(=O)(=O)c3ccc(cc3)C(C)(C)C)C(=O)OC
CC1([C@H]([C@H]1C(=O)Nc2nnc(s2)SCc3ccc(cc3)F)C=C(Cl)Cl)C
Cc1ccc2c(c1)sc(c2Cl)C(=O)Nc3nnc(s3)SCc4ccc(cc4)F
```

c1ccc(c(c1)C(=O)N2[C@@H](CC(=N2)c3ccc(cc3)Br)c4ccc(cc4)F)Cl
c1ccc(c(c1)C(=O)N2[C@H](CC(=N2)c3ccc(cc3)Br)c4ccc(cc4)F)Cl
Cc1ccc2c(c1)sc(c2Cl)C(=O)Nc3nnc(s3)SCc4ccc(cc4)Cl
COc1ccccc1[C@H]2CC(=NN2C(=O)c3ccc(cc3Cl)Cl)c4c(c5cc(ccc5[nH]c4=O)Br)c6ccccc6
COc1ccc(cc1)C2=NN([C@@H](C2)c3ccc(cc3)F)c4nc5ccc(cc5c(n4)c6ccccc6)Cl
COc1ccc(cc1)C2=NN([C@H](C2)c3ccc(cc3)F)c4nc5ccc(cc5c(n4)c6ccccc6)Cl
c1ccc(c(c1)C(=O)NN2C(=O)/C(=C\c3cccs3)/SC2=S)Br
c1ccc(cc1)c2c3cc(ccc3[nH]c(=O)c2C4=NN([C@@H](C4)c5cccc(c5)[N+](=O)[O-])C(=O)c6ccc(cc6Cl)Cl)Br
c1ccc(cc1)c2c3cc(ccc3[nH]c(=O)c2C4=NN([C@H](C4)c5cccc(c5)[N+](=O)[O-])C(=O)c6ccc(cc6Cl)Cl)Br
CCCCCCCCCCn1c2ccccc2nc1CCCO
CCCCCCCn1c2ccccc2nc1CCCO
CCCCCCCCCn1c2ccccc2nc1CCCO
CCCCCCCCCCCn1c2ccccc2nc1CCCO
CCCCCCCCCCCCCCCCCn1c2ccccc2nc1CCCO
CCCCCCCCCCCCCCCCCCn1c2ccccc2nc1c3ccccc3
c1cc(ccc1[C@@H]2CC(=NN2C(=O)c3cc(c(c(c3)[N+](=O)[O-])N4CCOCC4)[N+](=O)[O-])c5ccc(cc5)Br)Br
c1cc(ccc1[C@H]2CC(=NN2C(=O)c3cc(c(c(c3)[N+](=O)[O-])N4CCOCC4)[N+](=O)[O-])c5ccc(cc5)Br)Br
COc1ccc(cc1)C2=NN([C@@H](C2)c3cccc(c3)[N+](=O)[O-])C(=O)c4cc(c(c(c4)[N+](=O)[O-])N5CCOCC5)[N+](=O)[O-]
COc1ccc(cc1)C2=NN([C@H](C2)c3cccc(c3)[N+](=O)[O-])C(=O)c4cc(c(c(c4)[N+](=O)[O-])N5CCOCC5)[N+](=O)[O-]
CC1=NC2=C([C@H](C1C(=O)OC)c3ccc(c(c3)OC)O)C(=O)C[C@H](C2)c4ccc(cc4)Cl
CC1=NC2=C([C@@H](C1C(=O)OC)c3ccc(c(c3)OC)O)C(=O)C[C@H](C2)c4ccc(cc4)Cl
CC1=NC2=C([C@H](C1C(=O)OC)c3ccc(c(c3)OC)O)C(=O)C[C@@H](C2)c4ccc(cc4)Cl
CC1=NC2=C([C@@H](C1C(=O)OC)c3ccc(c(c3)OC)O)C(=O)C[C@@H](C2)c4ccc(cc4)Cl
CCCCCCCc1ccc(nc1)c2ccc(cc2)O
CC1=NC2=C([C@H](C1C(=O)OC)c3cccc(c3)OC)C(=O)C[C@H](C2)c4ccc(cc4)OC
CC1=NC2=C([C@@H](C1C(=O)OC)c3cccc(c3)OC)C(=O)C[C@H](C2)c4ccc(cc4)OC
CC1=NC2=C([C@H](C1C(=O)OC)c3cccc(c3)OC)C(=O)C[C@@H](C2)c4ccc(cc4)OC
CC1=NC2=C([C@@H](C1C(=O)OC)c3cccc(c3)OC)C(=O)C[C@@H](C2)c4ccc(cc4)OC
CC1=NC2=C([C@H](C1C(=O)OC)c3ccccc3OCc4ccccc4)C(=O)C[C@H](C2)c5ccc(cc5)Cl
CC1=NC2=C([C@@H](C1C(=O)OC)c3ccccc3OCc4ccccc4)C(=O)C[C@H](C2)c5ccc(cc5)Cl
CC1=NC2=C([C@H](C1C(=O)OC)c3ccccc3OCc4ccccc4)C(=O)C[C@@H](C2)c5ccc(cc5)Cl

```
CC1=NC2=C([C@@H](C1C(=O)OC)c3ccccc3OCc4ccccc4)C(=O)C[C@@H](C2)c5ccc(cc5)Cl
CC(C)(C)c1ccc(cc1)OCC(=O)Nc2nnc(s2)SCc3ccccc3Cl
CC1=NC2=C([C@H](C1C(=O)OCCc3ccccc3)c4ccc5c(c4)OCO5)C(=O)C[C@H](C2)c6ccccc6
CC1=NC2=C([C@@H](C1C(=O)OCCc3ccccc3)c4ccc5c(c4)OCO5)C(=O)C[C@H](C2)c6ccccc6
Cc1ccc(cc1)CSc2nnc(s2)NC(=O)c3c(c4ccc(cc4s3)C)Cl
CC1=NC2=C([C@@H](C1C(=O)OCCc3ccccc3)c4ccc5c(c4)OCO5)C(=O)C[C@@H](C2)c6ccccc6
CCCCCCOc1ccc(cc1)C(=O)Nc2nnc(s2)SCc3ccc(cc3)C
CC1=NC2=C([C@H](C1C(=O)OC3CCCCC3)c4ccc(cc4)OC(=O)C)C(=O)C[C@H](C2)c5ccccc5
CC1=NC2=C([C@@H](C1C(=O)OC3CCCCC3)c4ccc(cc4)OC(=O)C)C(=O)C[C@H](C2)c5ccccc5
Cc1ccc(cc1)CSc2nnc(s2)NC(=O)COc3ccc(cc3)C(C)(C)C
CC1=NC2=C([C@H](C1C(=O)OC3CCCCC3)c4ccc(cc4)OC(=O)C)C(=O)C[C@@H](C2)c5ccccc5
CC1=NC2=C([C@@H](C1C(=O)OC3CCCCC3)c4ccc(cc4)OC(=O)C)C(=O)C[C@@H](C2)c5ccccc5
CC1=NC2=C([C@H](C1C(=O)OC)c3ccc(cc3)[N+](=O)[O-])C(=O)C[C@H](C2)c4ccc(cc4)Cl
CC1=NC2=C([C@@H](C1C(=O)OC)c3ccc(cc3)[N+](=O)[O-])C(=O)C[C@H](C2)c4ccc(cc4)Cl
CC1=NC2=C([C@H](C1C(=O)OC)c3ccc(cc3)[N+](=O)[O-])C(=O)C[C@@H](C2)c4ccc(cc4)Cl
Cc1ccc(cc1)C(=O)C2=C(C(=O)N([C@@H]2c3ccc(cc3)C(C)(C)C)Cc4ccccc4)[O-]
CC1=NC2=C([C@@H](C1C(=O)OC)c3ccc(cc3)[N+](=O)[O-])C(=O)C[C@@H](C2)c4ccc(cc4)Cl
Cc1ccc(cc1)C(=O)C2=C(C(=O)N([C@H]2c3ccc(cc3)C(C)(C)C)Cc4ccccc4)[O-]
Cc1ccc(cc1)C(=O)C2=C(C(=O)N([C@@H]2c3cccc(c3)Oc4ccccc4)Cc5ccccc5)[O-]
CC\1(c2ccc3ccccc3c2N(/C1=C/C=C/C4=[N+](c5ccc6ccccc6c5C4(C)C)C)C)C
Cc1ccc(cc1)C(=O)C2=C(C(=O)N([C@H]2c3cccc(c3)Oc4ccccc4)Cc5ccccc5)[O-]
CCCCCCCCCCCCCCCn1c2c(c(=N)c3c1CCC3)CCC2
CCCOc1ccc(cc1)C(=O)C2=C(C(=O)N([C@@H]2c3ccc(cc3)N(C)C)CCc4ccccc4)[O-]
CCCOc1ccc(cc1)C(=O)C2=C(C(=O)N([C@H]2c3ccc(cc3)N(C)C)CCc4ccccc4)[O-]
COc1ccc(cc1)C(=O)C2=C(C(=O)N([C@@H]2c3cc(c(c(c3)OC)OC)OC)CCc4ccccc4)[O-]
CCOc1ccc(cc1)/N=N/[C@@H]2C(=NN(C2=O)C(=S)N)C
c1ccc2c(c1)-c3ccccc3C2(c4ccc(cc4)N5C(=O)C=CC5=O)c6ccc(cc6)N7C(=O)C=CC7=O
COc1ccc(cc1)C(=O)C2=C(C(=O)N([C@H]2c3cc(c(c(c3)OC)OC)OC)CCc4ccccc4)[O-]
c1ccc(cc1)c2csc(n2)N3C(=O)/C(=N\Nc4c(cc(cc4Br)Br)Br)/C(=N3)c5ccccc5
c1ccc(cc1)C\2=NN(C(=O)/C2=N/Nc3ccc(c(c3)[N+](=O)[O-])Cl)c4ccccc4
Cc1ccc(nc1)NC(=O)C2[C@H](C3=C(C[C@@H](CC3=O)c4ccccc4)N=C2C)c5cnn(c5C)C
```

```
Cc1cc(c(cc1C2(c3ccccc3C(=O)O2)c4cc(c(cc4C)OC(=O)CCC(=O)OC)C(C)C)C(C)C)OC(=O)CCC(=O)OC
CCOc1ccc(cc1)C(=O)C2=C(C(=O)N([C@@H]2c3ccc(cc3)C(C)(C)C)CCc4ccccc4)[O-]
Cc1ccc(cc1)c2csc(n2)N3C(=O)/C(=C/c4ccc(cc4)N(C)C)/C(=N3)c5ccccc5
CCOc1ccc(cc1)C(=O)C2=C(C(=O)N([C@H]2c3ccc(cc3)C(C)(C)C)CCc4ccccc4)[O-]
COc1ccccc1NC(=O)c2cc(ccc2I)I
CCCCOc1cc(c(cc1OC)[C@@H]2C3C(=NC4=C2C(=O)CCC4)CCCC3=O)Br
CCOc1ccc(cc1)C(=O)C2=C(C(=O)N([C@@H]2c3ccncc3)CCc4ccccc4)[O-]
COc1ccc(cc1)/C=C\C(=O)Nc2ccc3c(c2)nc(o3)c4ccc(c(c4)Br)OC
CCCCOc1ccc(cc1)[C@@H]2C(=C(C(=O)N2CCc3ccccc3)[O-])C(=O)c4ccc(cc4)OCC
CC1=C([C@@H](C2=C(N1)CC(CC2=O)(C)C)c3cccc(c3)F)C(=O)Nc4nc5ccccc5s4
CCCCOc1ccc(cc1)[C@H]2C(=C(C(=O)N2CCc3ccccc3)[O-])C(=O)c4ccc(cc4)OCC
CCOc1ccc(cc1)C(=O)C2=C(C(=O)N([C@@H]2c3ccc(cc3)OCc4ccccc4)CCc5ccccc5)O
COc1ccc(cc1Br)c2nc3cc(ccc3o2)NC(=O)c4ccc(o4)c5ccc(cc5)Cl
CC1=C([C@H](C2=C(N1)CC(CC2=O)(C)C)c3cccc(c3)F)C(=O)Nc4nc5ccccc5s4
CCOc1ccc(cc1)C(=O)C2=C(C(=O)N([C@H]2c3ccc(cc3)OCc4ccccc4)CCc5ccccc5)O
Cc1cc(c2c(c1)nc(o2)c3ccc(cc3)NC(=O)c4cccc(c4)OCc5ccccc5)C
CCOc1ccc(cc1)C(=O)C2=C(C(=O)N([C@@H]2c3ccc(c(c3)OC)OCc4ccccc4)CCc5ccccc5)O
CCOc1ccc(cc1)C(=O)C2=C(C(=O)N([C@H]2c3ccc(c(c3)OC)OCc4ccccc4)CCc5ccccc5)O
CCOc1ccc(cc1)C(=O)C2=C(C(=O)N([C@@H]2c3ccc(cc3)C(C)C)CCc4ccccc4)[O-]
CCOc1cc(cc(c1O)Br)[C@@H]2C3=C(C[C@@H](CC3=O)c4ccc(cc4)Cl)NC(=C2C(=O)OCCOC)C
CC1=C([C@@H](C2=C(N1)C[C@@H](CC2=O)c3ccccc3)c4cccc(c4)OC)C(=O)Nc5ccccn5
CCOc1cc(cc(c1O)Br)[C@H]2C3=C(C[C@@H](CC3=O)c4ccc(cc4)Cl)NC(=C2C(=O)OCCOC)C
CCOc1ccc(cc1)C(=O)C2=C(C(=O)N([C@H]2c3ccc(cc3)C(C)C)CCc4ccccc4)[O-]
CCOc1cc(cc(c1O)Br)[C@@H]2C3=C(C[C@H](CC3=O)c4ccc(cc4)Cl)NC(=C2C(=O)OCCOC)C
CCOc1cc(cc(c1O)Br)[C@H]2C3=C(C[C@H](CC3=O)c4ccc(cc4)Cl)NC(=C2C(=O)OCCOC)C
CCCOc1ccc(cc1)C(=O)C2=C(C(=O)N([C@@H]2c3ccc(cc3)CC)CCc4ccccc4)[O-]
CC1=NC2=C([C@H](C1C(=O)OC)c3cc(c(c(c3)Br)O)OC)C(=O)C[C@H](C2)c4ccc(cc4)OC
CC1=NC2=C([C@@H](C1C(=O)OC)c3cc(c(c(c3)Br)O)OC)C(=O)C[C@H](C2)c4ccc(cc4)OC
CC1=C([C@H](C2=C(N1)C[C@@H](CC2=O)c3ccccc3)c4cccc(c4)OC)C(=O)Nc5ccccn5
CC1=NC2=C([C@H](C1C(=O)OC)c3cc(c(c(c3)Br)O)OC)C(=O)C[C@@H](C2)c4ccc(cc4)OC
CC1=NC2=C([C@@H](C1C(=O)OC)c3cc(c(c(c3)Br)O)OC)C(=O)C[C@@H](C2)c4ccc(cc4)OC
```

```
CCCOc1ccc(cc1)C(=O)C2=C(C(=O)N([C@H]2c3ccc(cc3)CC)CCc4ccccc4)[O-]
CC1=C([C@@H](C2=C(N1)C[C@H](CC2=O)c3ccccc3)c4cccc(c4)OC)C(=O)Nc5ccccn5
CCCCN(CCCC)S(=O)(=O)c1ccc-2c(c1)C(=O)c3c2ccc(c3)S(=O)(=O)N(CCCC)CCCC
C[C@]12CC[C@](C1(C)C)(OC2=O)C(=O)Nc3ccc(cc3OC)c4ccc(c(c4)OC)NC(=O)[C@]56CC[C@](C5(C)C)(C(=O)O6)C
CC1=C([C@H](C2=C(N1)C[C@H](CC2=O)c3ccccc3)c4cccc(c4)OC)C(=O)Nc5ccccn5
C[C@]12CC[C@](C1(C)C)(OC2=O)C(=O)Nc3ccc(cc3OC)c4ccc(c(c4)OC)NC(=O)[C@@]56CC[C@@](C5(C)C)(C(=O)O6)C
CCOC(=O)C1[C@@H](C(=C(N=C1C)C)C(=O)OCC)c2cc(ccc2Br)OC
C[C@]12CC[C@](C1(C)C)(CC2=O)C(=O)Nc3ccc(cc3OC)c4ccc(c(c4)OC)NC(=O)[C@]56CC[C@](C5(C)C)(C(=O)C6)C
C[C@]12CC[C@](C1(C)C)(CC2=O)C(=O)Nc3ccc(cc3OC)c4ccc(c(c4)OC)NC(=O)[C@@]56CC[C@@](C5(C)C)(C(=O)C6)C
CC1=C([C@@H]([C@@H]2C(=N1)CC(CC2=O)(C)C)c3ccc(s3)Br)C(=O)Nc4ccccn4
C[C@]12CC[C@](C1(C)C)(CC2=O)C(=O)NCCCCCNC(=O)[C@]34CC[C@](C3(C)C)(C(=O)C4)C
C[C@]12CC[C@](C1(C)C)(CC2=O)C(=O)NCCCCCNC(=O)[C@@]34CC[C@@](C3(C)C)(C(=O)C4)C
CC1=C([C@H](C2=C(N1)CC(CC2=O)(C)C)c3ccc(s3)Br)C(=O)Nc4ccccn4
Cc1cc2c3c(c1)[C@H]4[C@@H](C[C@@H]([C@@H]4Cl)Sc5ccccc5[N+](=O)[O-])[C@@H](N3C[C@@H]6[C@@H]2C=CC6)C(=O)[O-]
CCOc1ccc2c(c1)sc(n2)N3[C@@H](C(=C(C3=O)O)C(=O)c4ccc(cc4)OC)c5ccc(c(c5)OC)OCc6ccccc6
Cc1cc2c3c(c1)[C@H]4[C@H](C[C@@H]([C@@H]4Cl)Sc5ccccc5[N+](=O)[O-])[C@@H](N3C[C@@H]6[C@@H]2C=CC6)C(=O)[O-]
CCOc1ccc2c(c1)sc(n2)N3[C@H](C(=C(C3=O)O)C(=O)c4ccc(cc4)OC)c5ccc(c(c5)OC)OCc6ccccc6
c1ccc(cc1)c2cc(nc3c2c(c(s3)C(=O)Nc4ccc(cc4Br)Br)N)c5ccccc5
C[C@@]12CC[C@@H](CC1=CC[C@@H]3[C@@H]2CC[C@@]4([C@@H]3C/C(=C\c5ccc(cc5)C(F)(F)F)/[C@@H]4O)C)O
Cc1ccc(cc1)c2cc(c(c(n2)SCC(=O)Nc3ccc(cc3)C(=O)OC)C#N)c4ccc(cc4)OC
COc1ccc(cc1)c2cc(nc3c2c(c(s3)C(=O)Nc4cc(cc(c4)Cl)Cl)N)c5ccccc5
CC1=NC2=C([C@H](C1C(=O)Nc3ccccn3)c4ccc(o4)COc5cccc(c5)C(F)(F)F)C(=O)CC(C2)(C)C
C[C@]12CC[C@@H](CC1=CC[C@@H]3[C@@H]2CC[C@@]4([C@@H]3C/C(=C\c5ccc(cc5)C(F)(F)F)/[C@@H]4O)C)O
CCOC(=O)c1ccc(cc1)NC(=O)c2c(c3c(cc(nc3s2)c4ccccc4)c5ccc(cc5)OC)N
Cc1c(c(no1)c2ccccc2Cl)C(=O)Nc3cc4c5ccccc5oc4cc3OC
C[C@@]12CC[C@H](CC1=CC[C@@H]3[C@@H]2CC[C@@]4([C@@H]3C/C(=C\c5ccc(cc5)C(F)(F)F)/[C@@H]4O)C)O
Cc1c(c(no1)c2c(cccc2Cl)Cl)C(=O)Nc3cc4c5ccccc5oc4cc3OC
C[C@]12CC[C@H](CC1=CC[C@@H]3[C@@H]2CC[C@@]4([C@@H]3C/C(=C\c5ccc(cc5)C(F)(F)F)/[C@@H]4O)C)O
C[C@@]12[C@@H]([C@@H](c3ccccc3O1)n4c(=O)/c(=C\c5ccc(cc5)OC(F)F)/sc4=N2)C(=O)OC
CC1=C([C@H](C2=C(N1)CC(CC2=O)(C)C)c3ccc(o3)COc4cccc(c4)C(F)(F)F)C(=O)Nc5ccccn5
C[C@@]12[C@H]([C@@H](c3ccccc3O1)n4c(=O)/c(=C\c5ccc(cc5)OC(F)F)/sc4=N2)C(=O)OC
```

```
c1cc(ccc1C#Cc2ccc(cc2)F)c3c(nc4cc(ccc4n3)C(=O)[O-])c5ccc(cc5)C#Cc6ccc(cc6)F
CC1=C([C@@H](C2=C(N1)CCCC2=O)c3ccc4c(c3)c5ccccc5n4C)C(=O)Nc6ccc(cn6)Br
Cc1ccc(c(c1)OCc2nc3c4c5c(sc4ncn3n2)C([NH2+]C(C5)(C)C)(C)C)C(C)C
CCOC(=O)C1=C(N=c2n(c(=O)/c(=C\c3ccc[nH]3)/s2)[C@H]1c4ccc(cc4)OC)C
C/C(=N\OCc1nc2c3c4c(sc3ncn2n1)CCCC4)/c5ccc(cc5)Br
COc1ccc(c(c1OC)OC)/C=C/C(=O)c2ccc(cc2)I
CC1(Cc2c3c4nc(nn4cnc3sc2C([NH2+]1)(C)C)c5ccc(cc5Cl)Cl)C
CC1=C([C@H](C2=C(N1)CCCC2=O)c3ccc4c(c3)c5ccccc5n4C)C(=O)Nc6ccc(cn6)Br
COc1cc(cc(c1OC)OC)/C=C/C(=O)c2ccc(cc2)I
C[C@@H](C(=O)NNC(=O)CSc1nc2ccccc2o1)NC(=O)c3ccccc3NC(=O)c4ccc(cc4)OC
C[C@H](C(=O)NNC(=O)CSc1nc2ccccc2o1)NC(=O)c3ccccc3NC(=O)c4ccc(cc4)OC
CCOC(=O)c1ccc(cc1)NC(=O)c2c(c3c(cc(nc3s2)c4ccc(cc4)C)c5ccc(cc5)OC)N
Cc1ccc(cc1)c2cc(c3c(c(sc3n2)C(=O)Nc4ccc(cc4)C(=O)OC)N)c5ccc(cc5)OC
c1cc(cc(c1)I)C(=O)O/N=C/2\CCCc3c2sc4c3c(=O)n5c(n4)CCCCC5
c1cc(ccc1[C@@H]2CC(=NN2C(=O)CCC(=O)Nc3ccc(cc3)Cl)c4ccc(cc4)Br)Br
c1cc(ccc1[C@H]2CC(=NN2C(=O)CCC(=O)Nc3ccc(cc3)Cl)c4ccc(cc4)Br)Br
CC1=C([C@@H](C2=C(N1)C[C@@H](CC2=O)c3ccccc3)c4ccc(o4)c5cccc(c5)Cl)C(=O)Nc6ccccn6
COc1ccc(cc1OC)c2cc(nc(c2C#N)SCC(=O)Nc3ccc(cc3)Cl)c4ccccc4
Cc1ccc(cc1)c2cc(nc(c2C#N)SCC(=O)Nc3ccc(cc3)Br)c4ccccc4
Cc1ccc(cc1)c2cc(nc(c2C#N)SCC(=O)Nc3ccccc3Cl)c4ccccc4
COc1ccc(cc1)c2cc(nc(c2C#N)SCC(=O)Nc3ccc(cc3)Cl)c4ccccc4
CC1=C([C@H](C2=C(N1)C[C@@H](CC2=O)c3ccccc3)c4ccc(o4)c5cccc(c5)Cl)C(=O)Nc6ccccn6
CCOc1ccc(cc1)c2cc(c(c(n2)SCC(=O)Nc3c(cc(cc3Cl)Cl)Cl)C#N)c4ccccc4
C[C@@H](c1ccccc1)NC(=O)c2cc3nc(cc(n3n2)C(F)(F)F)c4ccc(c(c4)Cl)Cl
C[C@H](c1ccccc1)NC(=O)c2cc3nc(cc(n3n2)C(F)(F)F)c4ccc(c(c4)Cl)Cl
CCOc1cccc(c1)C(=O)C2=C(C(=O)N([C@@H]2c3cccc(c3)O)Cc4ccncc4)[O-]
c1cc(oc1)c2cc(n3c(n2)c(c(n3)C(=O)Nc4ccc(cc4)OC(F)(F)F)Cl)C(F)(F)F
CC1=NC2=C([C@H](C1C(=O)Nc3ccccn3)c4ccc(o4)c5cccc(c5)Cl)C(=O)C[C@@H](C2)c6ccccc6
COc1ccc(cc1OC)c2cc(n3c(n2)c(c(n3)C(=O)N4CC[NH+](CC4)Cc5ccccc5)Cl)C(F)(F)F
CC1=C([C@H](C2=C(N1)C[C@H](CC2=O)c3ccccc3)c4ccc(o4)c5cccc(c5)Cl)C(=O)Nc6ccccn6
Cc1ccc(c(c1)C(=O)C2=C(C(=O)N([C@@H]2c3ccc(cc3)C(C)C)c4nnc(s4)C)[O-])C
```

CC1=NC2=C([C@H](C1C(=O)Nc3ccc(cn3)Br)c4ccc(o4)COc5ccc(cc5)C#N)C(=O)CCC2
Cc1ccc(c(c1)C(=O)C2=C(C(=O)N([C@@H]2c3ccc(cc3)C(C)(C)C)c4nnc(s4)C)[O-])C
CCOC(=O)c1c2c(sc1NC(=O)c3c(c4c(cc(nc4s3)c5ccc(cc5)Cl)c6ccccc6)N)CCCC2
CCOC(=O)c1c2c(sc1NC(=O)CSc3c(c(cc(n3)c4ccc(cc4)Cl)c5ccccc5)C#N)CCCC2
Cc1ccc(c(c1)C(=O)C2=C(C(=O)N([C@H]2c3ccc(cc3)C(C)(C)C)c4nnc(s4)C)[O-])C
CC1=NC2=C([C@@H](C1C(=O)Nc3ccc(cn3)Br)c4ccc(o4)COc5ccc(cc5)C#N)C(=O)CCC2
Cc1ccc(c(c1)C(=O)C2=C(C(=O)N([C@H]2c3cccc(c3)Cl)CCCn4cc[nH+]c4)[O-])C
CC1(C2=C(c3cc(ccc3N1C(=O)c4ccccc4)OC)C5(C(=C(S2)C(=O)OC)C(=O)OC)SC(=C(S5)C(=O)OC)C(=O)OC)C
Cc1cc(c2c(c1)C3=C(C(N2C(=O)Cc4ccccc4)(C)C)SC(=C(C35SC(=C(S5)C(=O)OC)C(=O)OC)C(=O)OC)C(=O)OC)C
Cc1ccc(cc1C)NC(=S)N[C@@H](C(Cl)(Cl)Cl)NC(=O)c2cccc(c2)Br
Cc1c(cccc1Cl)N/N=C/2\C(=NN(C2=O)c3nc(cs3)c4ccccc4)C
Cc1ccc(cc1C)NC(=S)N[C@H](C(Cl)(Cl)Cl)NC(=O)c2cccc(c2)Br
Cc1ccc(cc1C)NC(=S)N[C@@H](C(Cl)(Cl)Cl)NC(=O)c2ccc(cc2Cl)Cl
CCOC(=O)[C@@H]1[C@@H](CC2=C(C1=O)[C@@H](C(=C(N2)C)C(=O)OCC)c3ccc(o3)COc4ccc(cc4)OC)c5ccccc5
Cc1ccc(cc1C)NC(=S)N[C@H](C(Cl)(Cl)Cl)NC(=O)c2ccc(cc2Cl)Cl
Cc1cc(c(c(c1)Br)NC(=S)N[C@@H](C(Cl)(Cl)Cl)NC(=O)c2cccc(c2)Cl)Br
Cc1cc(c(c(c1)Br)NC(=S)N[C@H](C(Cl)(Cl)Cl)NC(=O)c2cccc(c2)Cl)Br
CCCCCC(=O)N[C@@H](C(Cl)(Cl)Cl)NC(=S)Nc1ccc(cc1)C(=O)C
CCCCCC(=O)N[C@H](C(Cl)(Cl)Cl)NC(=S)Nc1ccc(cc1)C(=O)C
CCOC(=O)[C@@H]1[C@H](CC2=C(C1=O)[C@@H](C(=C(N2)C)C(=O)OCC)c3ccc(o3)COc4ccc(cc4)OC)c5ccccc5
Cc1ccc(cc1)N2C(=O)/C(=C\Nc3c(cc(cn3)Br)Br)/C(=N2)C
Cc1ccc(cc1)N2C(=O)/C(=C/Nc3ccc(cc3OC)S(=O)(=O)Nc4ccccc4C)/C(=N2)C
COc1cc(cc(c1OC)OC)C(=O)N[C@@H](C(Cl)(Cl)Cl)NC(=S)Nc2ccc(cc2)C(=O)OC
CCOC(=O)[C@H]1[C@H](CC2=C(C1=O)[C@@H](C(=C(N2)C)C(=O)OCC)c3ccc(o3)COc4ccc(cc4)OC)c5ccccc5
COc1cc(cc(c1OC)OC)C(=O)N[C@H](C(Cl)(Cl)Cl)NC(=S)Nc2ccc(cc2)C(=O)OC
Cc1ccc(cc1C)N2C(=O)/C(=C\Nc3ccc(cc3OC)S(=O)(=O)Nc4ccccc4)/C(=N2)C
c1ccc(cc1)C(c2ccccc2)c3nc(c(o3)NCc4cccc(c4)Oc5ccccc5)S(=O)(=O)c6ccccc6
Cc1cccnc1NC(=O)C2=C(NC3=C([C@@H]2c4ccc(c(c4)COc5ccc(cc5)Cl)OC)C(=O)CCC3)C
CCOc1ccc(cc1OC)C(NC(=O)c2cccc(c2)Cl)NC(=O)c3cccc(c3)Cl
c1cc(cc(c1)I)C(=O)N[C@@H](C(Cl)(Cl)Cl)NC(=S)Nc2cccc(c2)C(F)(F)F
Cc1cccnc1NC(=O)C2[C@@H](C3=C(CCCC3=O)N=C2C)c4ccc(c(c4)COc5ccc(cc5)Cl)OC

```
c1cc(cc(c1)I)C(=O)N[C@H](C(Cl)(Cl)Cl)NC(=S)Nc2cccc(c2)C(F)(F)F
Cc1ccc(cc1)NS(=O)(=O)c2ccc(c(c2)OC)N/C=C/3\C(=NN(C3=O)c4ccc(cc4)Cl)C
Cc1ccccc1NS(=O)(=O)c2ccc(c(c2)OC)N/C=C/3\C(=NN(C3=O)c4ccc(cc4)Cl)C
CCOc1cc(cc(c1O)I)/C=C/2\C(=NN(C2=O)c3ccc(c(c3)Cl)Cl)C
CCCCOc1ccc(cc1)C(NC(=O)c2ccccc2F)NC(=O)c3ccccc3F
CCCNC(=O)[C@@]12CC[C@@]([C@@H]1Br)(C2(C)C)C(Br)Br
CC1=NC2=C([C@H](C1C(=O)Nc3ccc(cn3)Br)c4ccc(cc4OC)OC)C(=O)C[C@H](C2)c5ccccc5
c1ccc(cc1)[C@@H]2CC(=NN2C(=[NH2+])S[C@@H]3CC(=O)N(C3=O)c4cc(cc(c4)Cl)Cl)c5ccccc5
c1ccc(cc1)[C@H]2CC(=NN2C(=[NH2+])S[C@@H]3CC(=O)N(C3=O)c4cc(cc(c4)Cl)Cl)c5ccccc5
C[C@]12C[C@H](CC(C1)(C)C)N(C2)C(=O)[C@@]34CC[C@@]([C@@H]3Br)(C4(C)C)C(Br)Br
c1ccc(cc1)[C@@H]2CC(=NN2C(=[NH2+])S[C@@H]3CC(=O)N(C3=O)c4ccc(c(c4)Cl)Cl)c5ccccc5
c1ccc(cc1)[C@H]2CC(=NN2C(=[NH2+])S[C@@H]3CC(=O)N(C3=O)c4ccc(c(c4)Cl)Cl)c5ccccc5
CC1=C([C@H](C2=C(N1)C[C@@H](CC2=O)c3ccccc3)c4ccc(cc4OC)OC)C(=O)Nc5ccc(cn5)Br
C[C@@]12C[C@@H](CC(C1)(C)C)N(C2)C(=O)[C@@]34CC[C@@]([C@@H]3Br)(C4(C)C)C(Br)Br
CCCCCCOc1ccc(cc1)N2C(=O)C[C@@H](C2=O)SC(=[NH2+])N3[C@@H](CC(=N3)c4ccccc4)c5ccccc5
Cc1ccc(cc1)C2=NN([C@@H](C2)c3ccc(cc3)Cl)C(=[NH2+])S[C@@H]4CC(=O)N(C4=O)c5cccc(c5)Cl
Cc1ccc(cc1)C2=NN([C@H](C2)c3ccc(cc3)Cl)C(=[NH2+])S[C@@H]4CC(=O)N(C4=O)c5cccc(c5)Cl
CC1=C([C@@H](C2=C(N1)C[C@H](CC2=O)c3ccccc3)c4ccc(cc4OC)OC)C(=O)Nc5ccc(cn5)Br
Cc1ccc(cc1)C2=NN([C@@H](C2)c3ccc(cc3)Cl)C(=[NH2+])S[C@@H]4CC(=O)N(C4=O)c5ccc(cc5)F
CC1([C@@]2(CC[C@]1([C@@H]2Br)C(=O)NCc3ccc(cc3)OC)C(Br)Br)C
Cc1ccc(cc1)C2=NN([C@@H](C2)c3ccc(cc3)Cl)C(=[NH2+])S[C@@H]4CC(=O)N(C4=O)c5ccc(cc5)Br
Cc1ccc(cc1)C2=NN([C@H](C2)c3ccc(cc3)Cl)C(=[NH2+])S[C@@H]4CC(=O)N(C4=O)c5ccc(cc5)Br
CC1([C@@]2(CC[C@]1([C@@H]2Br)C(=O)Nc3nc4c(s3)CCCC4)C(Br)Br)C
Cc1ccc(cc1)C2=NN([C@@H](C2)c3ccc(cc3)Cl)C(=[NH2+])S[C@@H]4CC(=O)N(C4=O)c5cc(cc(c5)Cl)Cl
Cc1ccc(cc1)C2=NN([C@H](C2)c3ccc(cc3)Cl)C(=[NH2+])S[C@@H]4CC(=O)N(C4=O)c5cc(cc(c5)Cl)Cl
CC1([C@]2(CC[C@@]1([C@@H]2Br)C(=O)Nc3c(cc(cn3)Cl)Cl)C(Br)Br)C
CC1=C([C@H](C2=C(N1)C[C@H](CC2=O)c3ccccc3)c4ccc(cc4OC)OC)C(=O)Nc5ccc(cn5)Br
Cc1ccc(cc1)C2=NN([C@@H](C2)c3ccc(cc3)Cl)C(=[NH2+])S[C@@H]4CC(=O)N(C4=O)c5cccc(c5)Br
Cc1ccc(cc1)C2=NN([C@H](C2)c3ccc(cc3)Cl)C(=[NH2+])S[C@@H]4CC(=O)N(C4=O)c5cccc(c5)Br
CC1([C@@]2(CC[C@]1([C@@H]2Br)C(=O)Nc3c(cc(cn3)Cl)Cl)C(Br)Br)C
Cc1ccc(cc1)C2=NN([C@@H](C2)c3ccc(cc3)Cl)C(=[NH2+])S[C@@H]4CC(=O)N(C4=O)c5ccc(c(c5)Cl)Cl
```

```
Cc1ccc(cc1)C2=NN([C@H](C2)c3ccc(cc3)Cl)C(=[NH2+])S[C@@H]4CC(=O)N(C4=O)c5ccc(c(c5)Cl)Cl
CC1([C@]2(CC[C@@]1([C@@H]2Br)C(=O)Nc3ccc(cc3)SC(F)F)C(Br)Br)C
CCCOc1cc(c(cc1OC)[C@@H]2C(C(=NC(=C2C(=O)OCC)C)C)C(=O)OCC)Br
CC1([C@@]2(CC[C@]1([C@@H]2Br)C(=O)Nc3ccc(cc3)SC(F)F)C(Br)Br)C
c1ccc(cc1)Cn2cc(c3c2cccc3)/C=C\c4nc5ccccc5c(=O)n4c6ccccc6
Cc1ccc2c(c1)c(cc(n2)c3ccc(o3)c4ccc(c(c4)C(F)(F)F)Cl)C(=O)[O-]
Cc1cccc(c1)n2c(nc3ccccc3c2=O)/C=C/c4c(n(c5c4cc(cc5)OC)C)C
CCN1c2ccccc2/C(=C/c3nc4ccccc4c(=O)n3c5ccc(cc5)OC)/C1=O
Cc1ccc2c(c1)/C(=C/c3nc4ccccc4c(=O)n3c5ccc(cc5)OC)/C(=O)N2C
Cc1cc2c(cc1C)N(C(C3=C2C4(C(=C(S3)C(=O)OC)C(=O)OC)SC(=C(S4)C(=O)OC)C(=O)OC)(C)C)C(=O)c5cccc(c5)F
CCOC(=O)C1=C(C2[C@H]3[C@@H](C1([C@@H]4[C@@H]2C(=O)N(C4=O)c5ccc(cc5)C(C)C)C)C(=O)N(C3=O)c6ccc(cc6)C(C)C)C
CCOC(=O)C1=C(C2[C@@H]3[C@@H](C1([C@H]4[C@@H]2C(=O)N(C4=O)c5ccc(cc5)C(C)C)C)C(=O)N(C3=O)c6ccc(cc6)C(C)C)C
CCOC(=O)[C@@H]1[C@@H](CC2=C(C1=O)[C@@H](C(=C(N2)C)C(=O)OCC)c3ccc4c(c3)c5ccccc5n4C)c6ccccc6
CCOC(=O)C1=C(C2[C@@H]3[C@@H](C1([C@@H]4[C@@H]2C(=O)N(C4=O)c5ccc(c(c5)Cl)C)C)C(=O)N(C3=O)c6ccc(c(c6)Cl)C)C
CCOC(=O)C1=C(C2[C@H]3[C@@H](C1([C@@H]4[C@@H]2C(=O)N(C4=O)c5ccc(c(c5)Cl)C)C)C(=O)N(C3=O)c6ccc(c(c6)Cl)C)C
CCOC(=O)C1=C(C2[C@@H]3[C@@H](C1([C@H]4[C@@H]2C(=O)N(C4=O)c5ccc(c(c5)Cl)C)C)C(=O)N(C3=O)c6ccc(c(c6)Cl)C)C
CCOC(=O)C1=C(C2[C@H]3[C@H](C1([C@@H]4[C@@H]2C(=O)N(C4=O)c5ccc(c(c5)Cl)C)C)C(=O)N(C3=O)c6ccc(c(c6)Cl)C)C
CCCCCCCCCCSc1nc([nH]n1)N
CCOC(=O)[C@H]1[C@@H](CC2=C(C1=O)[C@@H](C(=C(N2)C)C(=O)OCC)c3ccc4c(c3)c5ccccc5n4C)c6ccccc6
CCOC(=O)[C@@H]1[C@H](CC2=C(C1=O)[C@@H](C(=C(N2)C)C(=O)OCC)c3ccc4c(c3)c5ccccc5n4C)c6ccccc6
Cc1cccc(c1)[C@@H]2c3c(=O)[nH]c(nc3NC4=C2C(=O)CC(C4)(C)C)SCc5ccccc5Cl
Cc1cccc(c1)[C@H]2c3c(=O)[nH]c(nc3NC4=C2C(=O)CC(C4)(C)C)SCc5ccccc5Cl
CCOC(=O)C1=C(N=c2n(c(=O)/c(=C/c3cc(n(c3c4ccccc4)c5ccc(cc5)Br)c6ccccc6)/s2)[C@@H]1c7ccccc7Cl)C
CCOC(=O)C1=C(N=c2n(c(=O)/c(=C/c3cc(n(c3c4ccccc4)c5ccc(cc5)Br)c6ccccc6)/s2)[C@H]1c7ccccc7Cl)C
CCOC(=O)C1=C(N=c2n(c(=O)/c(=C/c3cc(n(c3C)c4ccc(cc4)Br)C)/s2)[C@@H]1c5ccccc5Cl)C
CCOC(=O)[C@H]1[C@H](CC2=C(C1=O)[C@@H](C(=C(N2)C)C(=O)OCC)c3ccc4c(c3)c5ccccc5n4C)c6ccccc6
CCOC(=O)C1=C(N=c2n(c(=O)/c(=C/c3cc(n(c3C)c4ccc(cc4)Br)C)/s2)[C@H]1c5ccccc5Cl)C
COc1ccccc1OCCn2c3ccccc3nc2SCCOc4ccccc4OC
c1ccc(cc1)c2c(nc([nH]2)c3c4ccccc4c(c5c3cccc5)Cl)c6ccc(cc6)C(=O)c7ccccc7
CC1=NC2=C([C@H](C1C(=O)Nc3ccc(cn3)Br)c4cccc(c4)F)C(=O)C[C@H](C2)c5ccccc5
Cc1cccc(c1)C(=O)Nc2ccc3c(c2)nc(o3)c4cc(ccc4Cl)Br
```

```
Cc1cc2c(cc1C)oc(n2)c3cccc(c3)NC(=O)c4cc(c(c(c4)OC)OC)OC
CC1=C([C@H](C2=C(N1)C[C@@H](CC2=O)c3ccccc3)c4cccc(c4)F)C(=O)Nc5ccc(cn5)Br
COc1ccc(cc1OC)C(=O)Oc2ccc(cc2)C(=O)/C=C/c3ccccc3Cl
c1ccc(c(c1)COc2ccc(cc2)c3coc4cc(cc(c4c3=O)OCc5ccccc5Cl)OCc6ccccc6Cl)Cl
c1cc(ccc1c2c[nH]nc2c3ccc(cc3O)OCc4ccc(cc4Cl)Cl)Cl
CC1=NC2=C([C@H](C1C(=O)Nc3ccc(cn3)Br)c4cccc(c4)F)C(=O)C[C@@H](C2)c5ccccc5
CCOC(=O)C1=C(N=c2n(c(=O)/c(=C/c3ccc(o3)c4ccc(cc4)Br)/s2)[C@@H]1c5ccc(c(c5)OC)OC)C
CCOC(=O)C1=C(N=c2n(c(=O)/c(=C/c3ccc(o3)c4ccc(cc4)Br)/s2)[C@H]1c5ccc(c(c5)OC)OC)C
CCOC(=O)C1=C(N=c2n(c(=O)/c(=C\c3ccc(o3)c4ccc(cc4Cl)[N+](=O)[O-])/s2)[C@@H]1c5ccc(cc5)N(C)C)C
CCOC(=O)C1=C(N=c2n(c(=O)/c(=C\c3ccc(o3)c4ccc(cc4Cl)[N+](=O)[O-])/s2)[C@H]1c5ccc(cc5)N(C)C)C
CCOC(=O)C1=C(N=c2n(c(=O)/c(=C\c3ccc(c(c3)OC)OCc4cccc5c4cccc5)/s2)[C@@H]1c6ccc(cc6)N(C)C)C
CCOC(=O)C1=C(N=c2n(c(=O)/c(=C\c3ccc(c(c3)OC)OCc4cccc5c4cccc5)/s2)[C@H]1c6ccc(cc6)N(C)C)C
CCOC(=O)C1=C(N=c2n(c(=O)/c(=C\c3ccc(o3)c4ccc(cc4)Cl)/s2)[C@@H]1c5ccc6c(c5)OCO6)C
CC1=NC2=C([C@@H](C1C(=O)Nc3ccc(cn3)Br)c4cccc(c4)F)C(=O)C[C@@H](C2)c5ccccc5
CCOC(=O)C1=C(N=c2n(c(=O)/c(=C\c3ccc(o3)c4ccc(cc4)Cl)/s2)[C@H]1c5ccc6c(c5)OCO6)C
CCOC(=O)C1=C(N=c2n(c(=O)/c(=C\c3ccc(cc3)OCc4cccc5c4cccc5)/s2)[C@@H]1c6ccc(cc6)Cl)C
CCOC(=O)C1=C(N=c2n(c(=O)/c(=C\c3ccc(cc3)OCc4cccc5c4cccc5)/s2)[C@H]1c6ccc(cc6)Cl)C
Cc1ccc(cc1)COc2c(cc(cc2Br)/C=C(\C#N)/c3[nH]c4ccc(cc4n3)C)Br
c1ccc(cc1)COc2c(cc(cc2Cl)Cl)/C=C(\C#N)/c3[nH]c4ccccc4n3
CCN(CC)c1ccc(cc1)/C=c\2/c(=O)n3c(=NC(=C([C@H]3c4ccc(c(c4)OC)OC)C(=O)OCC)C)s2
CCOC(=O)[C@@H]1[C@@H](CC2=C(C1=O)[C@@H](C(=C(N2)C)C(=O)OCC)c3cnc4ccccc4n3)c5ccccc5
CCOC(=O)[C@H]1[C@@H](CC2=C(C1=O)[C@@H](C(=C(N2)C)C(=O)OCC)c3cnc4ccccc4n3)c5ccccc5
c1ccc2c(c1)CC[NH+](C2)Cc3ccc(cc3)C(=O)Nc4ccc(cc4)I
CCCCOc1ccc(cc1)C(=O)N2CCN(CC2)C(=O)c3ccc(cc3)OCCCC
CCOC(=O)[C@@H]1[C@H](CC2=C(C1=O)[C@@H](C(=C(N2)C)C(=O)OCC)c3cnc4ccccc4n3)c5ccccc5
CC(C)(C)n\1c(cs/c1=N\c2ccccc2)c3ccc(cc3)S(=O)(=O)N4CCCCCC4
CCOC(=O)[C@H]1[C@H](CC2=C(C1=O)[C@@H](C(=C(N2)C)C(=O)OCC)c3cnc4ccccc4n3)c5ccccc5
CC1CCN(CC1)S(=O)(=O)c2ccc(cc2)c3cs/c(=N\c4ccccc4)/n3C(C)(C)C
c1ccc(cc1)/[NH+]=c\2/n(c(cs2)c3ccc(cc3)S(=O)(=O)N4CCCC4)CCN5CCOCC5
CN(C)S(=O)(=O)c1ccc(cc1)c2cs/c(=N\c3ccccc3)/n2CC[NH+]4CCOCC4
COc1cc(ncn1)[N-]S(=O)(=O)c2ccc(cc2)NC(=O)c3c(c(n[nH]3)OC)[N+](=O)[O-]
```

```
Cc1c(cn(n1)C)[C@@H]2C3=C(CC(CC3=O)(C)C)NC(=C2C(=O)Nc4ccc(cn4)Br)C
CC1CCN(CC1)S(=O)(=O)c2ccc(cc2)c3cs/c(=N\c4ccccc4)/n3CC[NH+]5CCOCC5
c1ccc(cc1)/N=c\2/n(c(cs2)c3ccc(cc3)S(=O)(=O)N4CCCCCC4)CCC[NH+]5CCOCC5
Cc1c(cn(n1)C)[C@H]2C3=C(CC(CC3=O)(C)C)NC(=C2C(=O)Nc4ccc(cn4)Br)C
CN(C)S(=O)(=O)c1ccc(cc1)c2cs/c(=N\c3ccccc3)/n2C4CCCCC4
Cc1ccc2c(c1)C(=C3SC(=C(S3)C(=O)OC)C(=O)OC)C(=S)C(N2C(=O)CSc4nc5ccccc5s4)(C)C
CC1CCN(CC1)S(=O)(=O)c2ccc(cc2)c3cs/c(=N\c4ccccc4)/n3C5CCCCC5
Cc1ccc2c(c1)N(C(C(=S)C2=C3SC(=C(S3)C(=O)OC)C(=O)OC)(C)C)C(=O)CSc4nc5ccccc5s4
CCOC(=O)C1=C(SC(=C2c3cc(c(cc3N(C(C2=S)(C)C)C(=O)C(C)C)C)C)S1)C(=O)OCC
Cc1cc(n(n1)CC(=O)N2c3ccc(cc3C(=CC2(C)C)C)C(c4ccccc4)(c5ccccc5)c6ccccc6)C
CCCCCn\1c(cs/c1=N\c2ccccc2)c3ccc(cc3)S(=O)(=O)N4CCCC4
Cc1cc2c(cc1C)N(C(C(=S)C2=C3SC(=C(S3)C(=O)OC)C(=O)OC)(C)C)C(=O)COc4nc5ccccc5s4
Cc1ccnc(c1)NC(=O)C2[C@@H](C3=C(C[C@H](CC3=O)c4ccccc4)N=C2C)c5ccccc5C(F)(F)F
CCCCCn\1c(cs/c1=N\c2ccccc2)c3ccc(cc3)S(=O)(=O)N(CC)CC
c1ccc(cc1)Cn\2c(cs/c2=N\c3ccccc3)c4ccc(cc4)S(=O)(=O)N5CCCCCC5
Cc1ccnc(c1)NC(=O)C2=C(NC3=C([C@@H]2c4ccccc4C(F)(F)F)C(=O)C[C@H](C3)c5ccccc5)C
CN(C)S(=O)(=O)c1ccc(cc1)c2cs/c(=N\c3ccccc3)/n2Cc4ccccc4
Cc1ccc-2c(c1)N(C(c3c2c(=S)ss3)(C)C)C(=O)c4ccc(cc4Cl)Cl
CC1CCN(CC1)S(=O)(=O)c2ccc(cc2)c3cs/c(=N\c4ccccc4)/n3CCc5ccccc5
Cc1ccc2c(c1)N(C(C(=S)C2=C3SC(=C(S3)C(=O)OC)C(=O)OC)(C)C)C(=O)c4ccc(cc4Cl)Cl
COCCCn\1c(cs/c1=N\c2ccccc2)c3ccc(cc3)S(=O)(=O)N4CCCC4
CCCCCCCCCCCCCOC(=O)c1ccc(cc1)N2C(=O)[C@H]3[C@@H](C2=O)C4(c5ccccc5C3c6c4cccc6)Br
c1ccc(cc1)/N=c\2/n(c(cs2)c3ccc(cc3)S(=O)(=O)N4CCCCC4)c5ccccc5
CCCCCCCCCCCCCOC(=O)c1ccc(cc1)N2C(=O)[C@@H]3[C@@H](C2=O)C4(c5ccccc5C3c6c4cccc6)Br
c1ccc(cc1)/N=c\2/n(c(cs2)c3ccc(cc3)S(=O)(=O)N4CCCC4)c5ccccc5
CCCCCCCCCCCCCOC(=O)c1ccc(cc1)N2C(=O)[C@H]3[C@H](C2=O)C4(c5ccccc5C3c6c4cccc6)Br
CCOC(=O)COc1c(cc(cc1I)Br)[C@@H]2C3C(=NC4=C2C(=O)CCC4)CCCC3=O
CCCCCCCCCCCCCOC(=O)c1ccc(cc1)N2C(=O)[C@@H]3[C@H](C2=O)C4(c5ccccc5C3c6c4cccc6)Br
CCN(CC)S(=O)(=O)c1ccc(cc1)c2cs/c(=N\c3ccccc3)/n2c4ccccc4
CCN(CC)S(=O)(=O)c1ccc(cc1)c2cs/c(=N\c3ccccc3)/n2CCOC
c1ccc(cc1)CC(=O)Nc2c(c3c(s2)CCCC3)C(=O)Nc4cccc(c4)Cl
```

```
c1ccc2c(c1)nc(n2CC[NH+]3CCOCC3)NC(=O)C45C[C@H]6C[C@@H](C4)CC(C6)(C5)n7cnc(n7)Br
c1ccc(cc1)CC(=O)Nc2c(c3c(s2)CCCCC3)C(=O)Nc4cccc(c4)Cl
CCCn\1c(cs/c1=N\c2ccccc2)c3ccc(cc3)S(=O)(=O)N4CCCCCC4
CCCn\1c(cs/c1=N\c2ccccc2)c3ccc(cc3)S(=O)(=O)N4CCC(CC4)C
COc1cc(ccc1N/C=C/2\C(=O)NC(=O)N(C2=O)c3cccc(c3)Cl)SC#N
COc1c(c([n-]n1)C(=O)Nc2nc(cs2)c3ccc(c(c3)Cl)Cl)[N+](=O)[O-]
Cc1ccnc(c1)NC(=O)C2[C@H](C3=C(C[C@H](CC3=O)c4ccccc4)N=C2C)c5ccc(c(c5)COc6c(c(cc(c6F)F)F)F)OC
CCOC(=O)c1c2c(sc1N/C=C/3\C(=O)NC(=O)N(C3=O)c4ccc(cc4)C)CCCC2
Cc1c(sc(n1)N2[C@H](C(=C(C2=O)[O-])C(=O)c3ccc(cc3)OC)c4ccccc4)C(=O)C
Cc1ccnc(c1)NC(=O)C2=C(NC3=C([C@H]2c4ccc(c(c4)COc5c(c(cc(c5F)F)F)F)OC)C(=O)C[C@@H](C3)c6ccccc6)C
Cc1ccnc(c1)NC(=O)C2=C(NC3=C([C@@H]2c4ccc(c(c4)COc5c(c(cc(c5F)F)F)F)OC)C(=O)C[C@H](C3)c6ccccc6)C
CC\1=NN(C(=O)/C1=C/Nc2ccc(c(c2)C(=O)OC)Cl)c3c(cc(cc3Cl)Cl)Cl
CCOC(=O)c1cc(ccc1Cl)N/C=C/2\C(=NN(C2=O)c3c(cc(cc3Cl)Cl)Cl)C
CCCCOC(=O)c1cc(ccc1Cl)N/C=C/2\C(=NN(C2=O)c3c(cc(cc3Cl)Cl)Cl)C
Cc1ccnc(c1)NC(=O)C2[C@@H](C3=C(C[C@@H](CC3=O)c4ccccc4)N=C2C)c5ccc(c(c5)COc6c(c(cc(c6F)F)F)F)OC
CCCCOc1ccc(cc1OC)[C@@H]2C3C(=NC4=C2C(=O)CC(C4)(C)C)CC(CC3=O)(C)C
c1ccc(cc1)c2ccc(cc2)C(=O)N3[C@@](CC(=N3)C(F)(F)F)(c4ccc(cc4)Br)O
c1ccc(cc1)c2ccc(cc2)C(=O)N3[C@](CC(=N3)C(F)(F)F)(c4ccc(cc4)Br)O
Cc1cc(ccc1OC(C)C)C(=O)C2=C(C(=O)N([C@@H]2c3ccc(cc3)Cl)CCC[NH+](C)C)O
CC1=NC2=C([C@H](C1C(=O)Nc3ccccn3)c4ccc(c(c4)COc5ccc(cc5)C#N)OC)C(=O)C[C@H](C2)c6ccccc6
Cc1cc(ccc1OC(C)C)C(=O)C2=C(C(=O)N([C@H]2c3ccc(cc3)Cl)CCC[NH+](C)C)O
CC(C)CCOc1ccc(cc1OC)[C@@H]2C(=C(C(=O)N2CCC[NH+](C)C)O)C(=O)c3ccco3
c1cc(ccc1Cc2ccncc2)NC(=O)c3cc(ccc3I)I
CC(C)CCOc1ccc(cc1OC)[C@H]2C(=C(C(=O)N2CCC[NH+](C)C)O)C(=O)c3ccco3
CC1=C([C@H](C2=C(N1)C[C@@H](CC2=O)c3ccccc3)c4ccc(c(c4)COc5ccc(cc5)C#N)OC)C(=O)Nc6ccccn6
c1ccc(cc1)COc2ccc(cc2)c3c4c(nc(c3C#N)SCC(=O)OCc5ccccc5)CCCC4
COCCCN1[C@@H](C(=C(C1=O)O)C(=O)c2ccco2)c3ccc(c(c3)OC)OCC=C
CCCCc1ccc(cc1)C(=O)CSc2c(c(c3c(n2)CCCC3)c4ccc(cc4)OCc5ccccc5)C#N
COCCCN1[C@H](C(=C(C1=O)O)C(=O)c2ccco2)c3ccc(c(c3)OC)OCC=C
Cc1ccccc1NC(=O)C2=C(NC(=C([C@@H]2c3cccs3)C#N)SCC(=O)c4ccc(cc4)F)C
Cc1cc(ccc1OC(C)C)C(=O)C2=C(C(=O)N([C@@H]2c3ccncc3)CCC[NH+](C)C)O
```

```
CC1=C([C@@H](C2=C(N1)C[C@H](CC2=O)c3ccccc3)c4ccc(c(c4)COc5ccc(cc5)C#N)OC)C(=O)Nc6ccccn6
Cc1cc(ccc1OC(C)C)C(=O)C2=C(C(=O)N([C@H]2c3ccncc3)CCC[NH+](C)C)O
Cc1cc(ccc1OC(C)C)C(=O)C2=C(C(=O)N([C@@H]2c3cccs3)CCC[NH+](C)C)O
Cc1ccccc1NC(=O)C2=C(NC(=C([C@H]2c3cccs3)C#N)SCC(=O)c4ccc(cc4)F)C
Cc1cc(ccc1OC(C)C)C(=O)C2=C(C(=O)N([C@H]2c3cccs3)CCC[NH+](C)C)O
CCOC(=O)CSC1=NC(=C([C@H](C1C#N)c2cccs2)C(=O)Nc3ccccc3C)C
CCOC(=O)CSC1=NC(=C([C@@H](C1C#N)c2cccs2)C(=O)Nc3ccccc3C)C
CC1=C([C@@H](C(=C(N1)SC)C#N)c2ccccc2Cl)C(=O)Nc3ccccc3OC
CC1=C([C@H](C(=C(N1)SC)C#N)c2ccccc2Cl)C(=O)Nc3ccccc3OC
Cc1ccc(c(c1)[N+](=O)[O-])OCc2cc(ccc2OC)[C@@H]3C4=C(CCCC4=O)NC(=C3C(=O)Nc5ccc(cn5)Br)C
CCCCOc1ccc(cc1)C(=O)C2=C(C(=O)N([C@@H]2c3ccc(cc3)Br)c4nnc(s4)C)O
CCCCc1ccc(cc1)C(=O)CSC2=NC(=C([C@H](C2C#N)c3cccs3)C(=O)Nc4ccccc4OC)C
CCCCOc1ccc(cc1)C(=O)C2=C(C(=O)N([C@H]2c3ccc(cc3)Br)c4nnc(s4)C)O
Cc1ccc(c(c1)[N+](=O)[O-])OCc2cc(ccc2OC)[C@H]3C4=C(CCCC4=O)NC(=C3C(=O)Nc5ccc(cn5)Br)C
CCCCc1ccc(cc1)C(=O)CSC2=NC(=C([C@@H](C2C#N)c3cccs3)C(=O)Nc4ccccc4OC)C
CC1=C([C@@H](C2=C(N1)CC(CC2=O)(C)C)c3ccc(cc3)SC)C(=O)Nc4ccccn4
CCOc1ccc(cc1)NC(=O)c2c(c3c(cc(nc3s2)c4ccc(cc4)Cl)C(F)(F)F)N
CCCSC1=NC(=C([C@H](C1C#N)c2ccccc2Cl)C(=O)Nc3ccc(cc3)Cl)C
CCCSC1=NC(=C([C@@H](C1C#N)c2ccccc2Cl)C(=O)Nc3ccc(cc3)Cl)C
COc1ccc(cc1)CCN([C@H]2CC(=O)N(C2=O)c3ccccc3)C(=O)CCC(=O)[O-]
CCOc1ccc(cc1C)C(=O)C2=C(C(=O)N([C@H]2c3ccc(cc3)C)CCc4ccc(cc4)OC)[O-]
Cc1ccc(nc1)NC(=O)C2[C@H](C3=C(C[C@H](CC3=O)c4ccccc4)N=C2C)c5ccc(c(c5)COc6ccccc6C(=O)N)OC
CCCCCCCCCCCCCOC(=O)c1ccccc1N2C(=O)[C@@H]3[C@@H](C2=O)C4c5ccccc5C3c6c4cccc6
CCCCCCCCCCCCCOC(=O)c1ccccc1N2C(=O)[C@H]3[C@H](C2=O)C4c5ccccc5C3c6c4cccc6
CCCCCCCCCCCCCOC(=O)c1ccccc1N2C(=O)[C@@H]3[C@H](C2=O)C4c5ccccc5C3c6c4cccc6
Cc1ccc(nc1)NC(=O)C2=C(NC3=C([C@H]2c4ccc(c(c4)COc5ccccc5C(=O)N)OC)C(=O)C[C@@H](C3)c6ccccc6)C
Cc1cc(cc(c1)N2C(=O)/C(=C/c3ccc(c(c3)OC)OCc4ccc(cc4Cl)Cl)/C(=O)NC2=O)C
Cc1ccc(nc1)NC(=O)C2=C(NC3=C([C@@H]2c4ccc(c(c4)COc5ccccc5C(=O)N)OC)C(=O)C[C@H](C3)c6ccccc6)C
c1ccc(cc1)c2cc(c(n2c3ccc(cc3)Cl)c4ccccc4)C=C5C(=O)N(C(=S)N(C5=O)c6ccccc6)c7ccccc7
Cc1ccc(cc1)N2C(=O)/C(=C\c3cc(n(c3c4ccccc4)c5ccc(cc5)Cl)c6ccccc6)/C(=O)NC2=S
Cc1ccc(nc1)NC(=O)C2=C(NC3=C([C@H]2c4ccc(c(c4)COc5ccccc5C(=O)N)OC)C(=O)C[C@H](C3)c6ccccc6)C
```

```
Cc1cc(ccc1C(=O)C2=C(C(=O)N([C@@H]2c3ccc(cc3)Br)CCC[NH+](C)C)O)OC(C)C
Cc1cc(ccc1C(=O)C2=C(C(=O)N([C@H]2c3ccc(cc3)Br)CCC[NH+](C)C)O)OC(C)C
c1ccc(cc1)c2cc(=O)[nH]c(n2)SCC(=O)Nc3ccccc3Oc4ccccc4
Cc1cc(ccc1OCc2ccccc2)C(=O)C3=C(C(=O)N([C@@H]3c4cccc(c4)Br)CCC[NH+](C)C)O
CCOc1cc(cc(c1OCc2ccccc2)Cl)[C@H]3C4C(=NC5=C3C(=O)CCC5)CCCC4=O
Cc1cc(ccc1OCc2ccccc2)C(=O)C3=C(C(=O)N([C@H]3c4cccc(c4)Br)CCC[NH+](C)C)O
COCCN1[C@@H](C(=C(C1=O)[O-])C(=O)c2ccc(cc2)OCC=C)c3ccc(c(c3)Cl)Cl
COCCN1[C@H](C(=C(C1=O)[O-])C(=O)c2ccc(cc2)OCC=C)c3ccc(c(c3)Cl)Cl
CC1=C([C@@H](NC(=O)N1)c2cccc(c2)Br)C(=O)OCc3ccc(cc3)OC
CCOc1cc(ccc1O)[C@@H]2C(=C(C(=O)N2CCOC)[O-])C(=O)c3ccc(cc3)OCC=C
CCCOc1ccc(cc1OC)[C@@H]2C3=C(C[C@@H](CC3=O)c4ccccc4)NC(=C2C(=O)OCC)C
CCCOc1ccc(cc1OC)[C@H]2C3=C(C[C@@H](CC3=O)c4ccccc4)NC(=C2C(=O)OCC)C
CCOc1cc(ccc1O)[C@H]2C(=C(C(=O)N2CCOC)[O-])C(=O)c3ccc(cc3)OCC=C
CCCOc1ccc(cc1OC)[C@@H]2C3=C(C[C@H](CC3=O)c4ccccc4)NC(=C2C(=O)OCC)C
COc1ccc(cc1F)C(=O)C2=C(C(=O)N([C@@H]2c3cccc(c3)Oc4ccccc4)CC[NH+]5CCOCC5)O
CCCOc1ccc(cc1OC)[C@H]2C3=C(C[C@H](CC3=O)c4ccccc4)NC(=C2C(=O)OCC)C
COc1ccc(cc1F)C(=O)C2=C(C(=O)N([C@H]2c3cccc(c3)Oc4ccccc4)CC[NH+]5CCOCC5)O
CC[NH+](CC)CCCN1[C@@H](C(=C(C1=O)O)C(=O)c2ccc(cc2)OCc3ccc(cc3)C)c4ccc(cc4)O
Cc1cccnc1NC(=O)C2=C(NC3=C([C@@H]2c4ccc(c(c4)COc5cccc(c5Cl)Cl)OC)C(=O)CCC3)C
CC[NH+](CC)CCCN1[C@H](C(=C(C1=O)O)C(=O)c2ccc(cc2)OCc3ccc(cc3)C)c4ccc(cc4)O
C[NH+](C)CCN1[C@@H](C(=C(C1=O)O)C(=O)c2ccc(cc2)OC)c3ccc(cc3)OCc4ccccc4
Cc1ccc2c(c1)C(=C3SC(=C(S3)C(=O)OC)C(=O)OC)C(=S)C(N2C(=O)COc4ccc(cc4)Cl)(C)C
C[NH+](C)CCN1[C@H](C(=C(C1=O)O)C(=O)c2ccc(cc2)OC)c3ccc(cc3)OCc4ccccc4
Cc1cccnc1NC(=O)C2=C(NC3=C([C@H]2c4ccc(c(c4)COc5cccc(c5Cl)Cl)OC)C(=O)CCC3)C
Cc1cc2c(cc1C)N(C(C(=S)C2=C3SC(=C(S3)C(=O)OC)C(=O)OC)(C)C)C(=O)c4ccc5c(c4)OCO5
COc1cccc(c1)OCCSc2nc(c(n2c3ccccc3)c4ccccc4)c5ccccc5
Cc1ccc(nc1)NC(=O)C2=C(NC3=C([C@@H]2c4ccc(c(c4)CSc5nc(cc(n5)C(F)F)C)OC)C(=O)CC(C3)(C)C)C
Cc1ccc(nc1)NC(=O)C2=C(NC3=C([C@H]2c4ccc(c(c4)CSc5nc(cc(n5)C(F)F)C)OC)C(=O)CC(C3)(C)C)C
Cc1ccc(cc1)C(=O)N2c3ccc(cc3[C@@](CC2(C)C)(C)c4ccc(cc4)F)C(c5ccccc5)(c6ccccc6)c7ccccc7
Cc1ccc(cc1)C(=O)N2c3ccc(cc3[C@](CC2(C)C)(C)c4ccc(cc4)F)C(c5ccccc5)(c6ccccc6)c7ccccc7
CC(C)Oc1ccc(cc1)c2nc3n(n2)c(=O)/c(=C\c4ccc(cc4OC)OC)/s3
```

```
CC1=C([C@@H](C2=C(N1)CC(CC2=O)(C)C)c3ccccn3)C(=O)Nc4ccc(cn4)Br
c1cc(ccc1c2cc(n3c(n2)c(cn3)C(=O)Nc4cc(cc(c4)Oc5ccc6c(c5)CCC6)[N+](=O)[O-])C(F)(F)F)Br
CCOc1ccc(cc1)NC(=O)CSc2c(c(cc(n2)c3ccc(cc3)C)C(F)(F)F)C#N
CCOc1cc(ccc1O)[C@@H]2C(=C(C(=O)N2CC[NH+]3CCOCC3)O)C(=O)c4ccc(cc4C)OCc5ccccc5
CCOc1cc(ccc1O)[C@H]2C(=C(C(=O)N2CC[NH+]3CCOCC3)O)C(=O)c4ccc(cc4C)OCc5ccccc5
CC1=NC2=C([C@@H](C1C(=O)Nc3ccc(cn3)Br)c4ccccn4)C(=O)CC(C2)(C)C
Cc1cc(ccc1C(=O)C2=C(C(=O)N([C@@H]2c3ccc(cc3)C(C)(C)C)CC[NH+]4CCOCC4)O)OCc5ccccc5
CC(C)(C)c1ccc(cc1)[C@@H]2C(=C(C(=O)N2CCCOC)[O-])C(=O)c3ccc(cc3)OCC=C
CC(C)(C)c1ccc(cc1)[C@H]2C(=C(C(=O)N2CCCOC)[O-])C(=O)c3ccc(cc3)OCC=C
CCOC(=O)C1=C(N=c2n(c(=O)/c(=C\c3ccc(o3)c4cc(ccc4OC)[N+](=O)[O-])/s2)[C@@H]1c5ccccc5)C
CCOC(=O)C1=C(N=c2n(c(=O)/c(=C\c3ccc(o3)c4cc(ccc4OC)[N+](=O)[O-])/s2)[C@H]1c5ccccc5)C
CCOC(=O)C1=C(N=c2n(c(=O)/c(=C\c3ccc(o3)c4cccc(c4)C(F)(F)F)/s2)[C@@H]1c5ccccc5)C
CCOC(=O)C1=C(N=c2n(c(=O)/c(=C\c3ccc(o3)c4cccc(c4)C(F)(F)F)/s2)[C@H]1c5ccccc5)C
COCCCN1[C@@H](C(=C(C1=O)[O-])C(=O)c2ccc(cc2)OCC=C)c3ccc(cc3)OC
CCOC(=O)C1=C(N=c2n(c(=O)/c(=C\c3ccc(o3)c4cccc(c4)Cl)/s2)[C@@H]1c5ccc(c(c5)OC)OC)C
COCCCN1[C@H](C(=C(C1=O)[O-])C(=O)c2ccc(cc2)OCC=C)c3ccc(cc3)OC
CCOC(=O)C1=C(N=c2n(c(=O)/c(=C\c3ccc(o3)c4cccc(c4)Cl)/s2)[C@H]1c5ccc(c(c5)OC)OC)C
CCOC(=O)C1=C(N=c2n(c(=O)/c(=C\c3ccc(o3)c4cccc(c4)C(F)(F)F)/s2)[C@@H]1c5ccc(c(c5)OC)OC)C
CCOC(=O)C1=C(N=c2n(c(=O)/c(=C\c3ccc(o3)c4cccc(c4)C(F)(F)F)/s2)[C@H]1c5ccc(c(c5)OC)OC)C
CCOC(=O)C1=C(N=c2n(c(=O)/c(=C\c3ccc(o3)c4cccc(c4)Cl)/s2)[C@@H]1c5ccc6c(c5)OCO6)C
CCOC(=O)C1=C(N=c2n(c(=O)/c(=C\c3ccc(o3)c4cccc(c4)Cl)/s2)[C@H]1c5ccc6c(c5)OCO6)C
CC[NH+](CC)CCN1[C@@H](C(=C(C1=O)O)C(=O)c2ccc(c(c2)F)OC)c3ccc(cc3)OC
CCOC(=O)C1=C(N=c2n(c(=O)/c(=C\c3ccc(o3)c4cccc(c4)C(F)(F)F)/s2)[C@@H]1c5ccc6c(c5)OCO6)C
Cc1ccnc(c1)NC(=O)C2=C(NC3=C([C@@H]2c4ccc5c(c4)c6ccccc6n5C)C(=O)C[C@@H](C3)c7ccccc7)C
CCOC(=O)C1=C(N=c2n(c(=O)/c(=C\c3ccc(o3)c4cccc(c4)C(F)(F)F)/s2)[C@H]1c5ccc6c(c5)OCO6)C
CCOC(=O)C1=C(N=c2n(c(=O)/c(=C\c3ccc(o3)c4cccc(c4)C(F)(F)F)/s2)[C@@H]1c5ccc(cc5)Cl)C
CC[NH+](CC)CCN1[C@H](C(=C(C1=O)O)C(=O)c2ccc(c(c2)F)OC)c3ccc(cc3)OC
CCOC(=O)C1=C(N=c2n(c(=O)/c(=C\c3ccc(o3)c4cccc(c4)C(F)(F)F)/s2)[C@H]1c5ccc(cc5)Cl)C
CCOC(=O)C1=C(N=c2n(c(=O)/c(=C\c3c[nH]c4c3cccc4)/s2)[C@@H]1c5cc(ccc5OC)OC)C
CCOC(=O)C1=C(N=c2n(c(=O)/c(=C\c3c[nH]c4c3cccc4)/s2)[C@H]1c5cc(ccc5OC)OC)C
COc1ccc(cc1F)C(=O)C2=C(C(=O)N([C@H]2c3ccc(cc3)OCc4ccccc4)CCCn5ccnc5)O
```

```
Cc1ccnc(c1)NC(=O)C2[C@@H](C3=C(C[C@H](CC3=O)c4ccccc4)N=C2C)c5ccc6c(c5)c7ccccc7n6C
CCOC(=O)C1=C(N=c2n(c(=O)/c(=C\c3ccc(o3)c4cccc(c4)C(F)(F)F)/s2)[C@@H]1c5cc(ccc5OC)OC)C
CCOC(=O)C1=C(N=c2n(c(=O)/c(=C\c3ccc(o3)c4cccc(c4)C(F)(F)F)/s2)[C@H]1c5cc(ccc5OC)OC)C
CCOC(=O)C1=C(N=c2n(c(=O)/c(=C\c3ccc(o3)c4cccc(c4)Cl)/s2)[C@@H]1c5cccs5)C
CCOC(=O)C1=C(N=c2n(c(=O)/c(=C\c3ccc(o3)c4cccc(c4)Cl)/s2)[C@H]1c5cccs5)C
Cc1ccnc(c1)NC(=O)C2[C@H](C3=C(C[C@@H](CC3=O)c4ccccc4)N=C2C)c5ccc6c(c5)c7ccccc7n6C
CCOC(=O)C1=C(N=c2n(c(=O)/c(=C\c3ccc(o3)c4ccc(c(c4)Cl)Cl)/s2)[C@@H]1c5c6ccccc6ccc5OC)C
CCOC(=O)C1=C(N=c2n(c(=O)/c(=C\c3ccc(o3)c4ccc(c(c4)Cl)Cl)/s2)[C@H]1c5c6ccccc6ccc5OC)C
C[NH+](C)CCCN1[C@@H](C(=C(C1=O)O)C(=O)c2ccc(c(c2)OC)OC)c3cc(c(c(c3)Br)O)OC
c1ccc(cc1)C2=C/C(=C\c3ccc(o3)c4ccc(c(c4)Cl)Cl)/C(=O)N2c5cccc(c5)Cl
CCOc1ccc(cc1)N2C(=C/C(=C\c3ccc(o3)c4ccc(c(c4)Cl)Cl)/C2=O)c5ccccc5
CCOC(=O)C1=C(N=c2n(c(=O)/c(=C\c3cc(n(c3C)c4ccc(cc4)F)C)/s2)[C@@H]1c5ccccc5)C
CCOC(=O)C1=C(N=c2n(c(=O)/c(=C\c3cc(n(c3C)c4ccc(cc4)F)C)/s2)[C@H]1c5ccccc5)C
CCOC(=O)C1=C(N=c2n(c(=O)/c(=C\c3cc(n(c3C)c4ccc(cc4)Cl)C)/s2)[C@@H]1c5ccc(cc5)N(C)C)C
C[NH+](C)CCCN1[C@H](C(=C(C1=O)O)C(=O)c2ccc(c(c2)OC)OC)c3cc(c(c(c3)Br)O)OC
CCOC(=O)C1=C(N=c2n(c(=O)/c(=C\c3cc(n(c3C)c4ccc(cc4)Cl)C)/s2)[C@H]1c5ccc(cc5)N(C)C)C
CCOC(=O)C1=C(N=c2n(c(=O)/c(=C\c3cc(n(c3C)c4ccc(cc4)F)C)/s2)[C@@H]1c5ccc(cc5)N(C)C)C
CCOc1cc(c(cc1OCC(=O)[O-])Br)[C@@H]2C3C(=NC4=C2C(=O)CC(C4)(C)C)CC(CC3=O)(C)C
CCOC(=O)C1=C(N=c2n(c(=O)/c(=C\c3cc(n(c3C)c4ccc(cc4)F)C)/s2)[C@H]1c5ccc(cc5)N(C)C)C
COCCCN1[C@@H](C(=C(C1=O)O)C(=O)c2ccc(c(c2)F)OC)c3ccc(c(c3)OC)OCc4ccccc4
CCOC(=O)C1=C(N=c2n(c(=O)/c(=C\c3cc(n(c3C)c4ccc(cc4)Br)C)/s2)[C@@H]1c5ccc(cc5)N(C)C)C
CCOC(=O)C1=C(N=c2n(c(=O)/c(=C\c3cc(n(c3C)c4ccc(cc4)Br)C)/s2)[C@H]1c5ccc(cc5)N(C)C)C
CCOC(=O)C1=C(N=c2n(c(=O)/c(=C\c3cc(n(c3C)c4ccc(cc4)Cl)C)/s2)[C@@H]1c5ccc6c(c5)OCO6)C
COCCCN1[C@H](C(=C(C1=O)O)C(=O)c2ccc(c(c2)F)OC)c3ccc(c(c3)OC)OCc4ccccc4
CCOC(=O)C1=C(N=c2n(c(=O)/c(=C\c3cc(n(c3C)c4ccc(cc4)Cl)C)/s2)[C@H]1c5ccc6c(c5)OCO6)C
Cc1cccnc1NC(=O)C2=C(NC3=C([C@@H]2c4c(nn(c4Cl)c5ccccc5)C)C(=O)C[C@@H](C3)c6ccccc6)C
CCOC(=O)C1=C(N=c2n(c(=O)/c(=C\c3cc(n(c3C)c4ccc(cc4)c5ccccc5)C)/s2)[C@@H]1c6ccc7c(c6)OCO7)C
CCOC(=O)C1=C(N=c2n(c(=O)/c(=C\c3cc(n(c3C)c4ccc(cc4)c5ccccc5)C)/s2)[C@H]1c6ccc7c(c6)OCO7)C
c1cc(c(cc1Cl)Cl)C(=O)Nc2c(c3c(s2)CCCC3)C(=O)N[C@@H]4CCS(=O)(=O)C4
CCOC(=O)C1=C(N=c2n(c(=O)/c(=C\c3cc(n(c3C)c4ccc(cc4)C)C)/s2)[C@@H]1c5ccc(c(c5)OC)OC)C
c1cc(c(cc1Cl)Cl)C(=O)Nc2c(c3c(s2)CCCC3)C(=O)N[C@H]4CCS(=O)(=O)C4
```

```
CCOC(=O)C1=C(N=c2n(c(=O)/c(=C\c3cc(n(c3C)c4ccc(cc4)C)C)/s2)[C@H]1c5ccc(c(c5)OC)OC)C
CCOC(=O)C1=C(N=c2n(c(=O)/c(=C\c3cc(n(c3C)c4ccc(cc4)Br)C)/s2)[C@@H]1c5ccc(c(c5)OC)OC)C
CCOC(=O)C1=C(N=c2n(c(=O)/c(=C\c3cc(n(c3C)c4ccc(cc4)Br)C)/s2)[C@H]1c5ccc(c(c5)OC)OC)C
Cc1ccccc1OCC(=O)Nc2c(c3c(s2)CCCC3)C(=O)N[C@@H]4CCS(=O)(=O)C4
CCOC(=O)C1=C(N=c2n(c(=O)/c(=C\c3ccc(o3)c4ccc(cc4Br)C)/s2)[C@@H]1c5cc(ccc5OC)OC)C
CCOC(=O)C1=C(N=c2n(c(=O)/c(=C\c3ccc(o3)c4ccc(cc4Br)C)/s2)[C@H]1c5cc(ccc5OC)OC)C
Cc1ccccc1OCC(=O)Nc2c(c3c(s2)CCCC3)C(=O)N[C@H]4CCS(=O)(=O)C4
COCCCN1[C@@H](C(=C(C1=O)[O-])C(=O)c2ccc(cc2)OCC=C)c3ccc(cc3)F
Cc1ccc2c(c1)c(c(n2C[C@H](Cn3cnc(c3c4ccccc4)c5ccccc5)O)c6ccccc6)c7ccccc7
COCCCN1[C@H](C(=C(C1=O)[O-])C(=O)c2ccc(cc2)OCC=C)c3ccc(cc3)F
Cc1cccnc1NC(=O)C2[C@@H](C3=C(CCCC3=O)N=C2C)c4ccc(c(c4)COC(=O)C)OC
Cc1ccc(c(c1)NC(=O)CSc2nc3c(c4c(s3)CCCC4)c(=O)n2c5cccc6c5cccc6)C
Cn1cc(c(n1)C(=O)Nc2nc3ccccc3n2CC[NH+]4CCOCC4)I
c1ccc2c(c1)cccc2n3c(=O)c4c5c(sc4nc3SCC(=O)c6cc(sc6Cl)Cl)CCCC5
Cc1ccc(cc1)NC(=O)CSc2nc3c(c4c(s3)CCC4)c(=O)n2c5ccccc5
CC1=C([C@@H]([C@@H]2C(=N1)CC(CC2=O)(C)C)c3ccc(cc3)SC)C(=O)Nc4ccc(cn4)Br
CC(C)(C)c1cc(cc(c1O)C(C)(C)C)n2c3ccccc3nc2c4cccc(c4)Br
CC(C)(C)c1cc(cc(c1O)C(C)(C)C)n2c3cc(c(cc3nc2c4ccc(cc4)N(C)C)OC)OC
CC1=NC2=C([C@@H](C1C(=O)Nc3ccc(cn3)Br)c4ccc(cc4)SC)C(=O)CC(C2)(C)C
Cc1ccnc(c1)NC(=O)C2[C@H](C3=C(CCCC3=O)N=C2C)c4ccc(c(c4)OC)OCC(=O)N
Cc1ccc(cc1)SCc2cc(ccc2OC)[C@@H]3C4=C(CCCC4=O)NC(=C3C(=O)Nc5c(cccn5)C)C
Cc1cc2c(cc1C)N(C(C(=S)C2=C3SC(=C(S3)C(=O)OC)C(=O)OC)(C)C)C(=O)CCC4CCCCC4
Cc1ccc(cc1)SCc2cc(ccc2OC)[C@H]3C4=C(CCCC4=O)NC(=C3C(=O)Nc5c(cccn5)C)C
Cc1ccc2c(c1)N(C(C(=S)C2=C3SC(=C(S3)C(=O)OC)C(=O)OC)(C)C)C(=O)CCC4CCCCC4
Cc1ccc2c(c1)-c3c(ssc3=S)C(N2C(=O)c4ccc(cc4)C(F)(F)F)(C)C
CCOc1cc(cc(c1OCC(=O)[O-])Cl)[C@@H]2C3C(=NC4=C2C(=O)CCC4)CCCC3=O
Cc1cc2c(cc1C)N(C(C(=S)C2=C3SC(=C(S3)C(=O)OC)C(=O)OC)(C)C)C(=O)c4ccc(cc4Cl)Cl
CCOc1cccc2c1N(C(C(=S)C2=C3SC(=C(S3)C(=O)OC)C(=O)OC)(C)C)C(=O)c4ccc(cc4Cl)Cl
Cc1ccc(cc1)/C=C/C(=O)N2c3ccc(cc3C(=C4SC(=C(S4)C(=O)OC)C(=O)OC)C(=S)C2(C)C)OC
CC1=NC2=C([C@H](C1C(=O)OC3CCCCC3)c4cccc(c4Cl)Cl)C(=O)C[C@H](C2)c5ccccc5
COc1cc(cc(c1OC)Br)[C@H]2C3=C(CCCC3=O)N=C4C2=C(CCC4)[O-]
```

CC1=NC2=C([C@@H](C1C(=O)OC3CCCCC3)c4cccc(c4Cl)Cl)C(=O)C[C@H](C2)c5ccccc5
CC1=NC2=C([C@H](C1C(=O)OC3CCCCC3)c4cccc(c4Cl)Cl)C(=O)C[C@@H](C2)c5ccccc5
CC1=NC2=C([C@@H](C1C(=O)OC3CCCCC3)c4cccc(c4Cl)Cl)C(=O)C[C@@H](C2)c5ccccc5
CCOC(=O)C1[C@H](C2=C(C[C@H](CC2=O)c3ccc(cc3)OC)N=C1C)c4ccc(c(c4)OC)O
CCOC(=O)C1[C@@H](C2=C(C[C@H](CC2=O)c3ccc(cc3)OC)N=C1C)c4ccc(c(c4)OC)O
Cc1cc(ccc1Cl)OCc2ccc(o2)[C@@H]3C4=C(CC(CC4=O)(C)C)NC(=C3C(=O)OC)C
CCOC(=O)C1[C@H](C2=C(C[C@@H](CC2=O)c3ccc(cc3)OC)N=C1C)c4ccc(c(c4)OC)O
CCOC(=O)C1[C@@H](C2=C(C[C@@H](CC2=O)c3ccc(cc3)OC)N=C1C)c4ccc(c(c4)OC)O
Cc1cc(ccc1Cl)OCc2ccc(o2)[C@H]3C4=C(CC(CC4=O)(C)C)NC(=C3C(=O)OC)C
CC1=NC2=C([C@H](C1C(=O)OC(C)C)c3ccc(cc3)OCc4ccccc4)C(=O)C[C@H](C2)c5ccc(cc5)OC
CC1=NC2=C([C@@H](C1C(=O)OC(C)C)c3ccc(cc3)OCc4ccccc4)C(=O)C[C@H](C2)c5ccc(cc5)OC
CC1=NC2=C([C@H](C1C(=O)OC(C)C)c3ccc(cc3)OCc4ccccc4)C(=O)C[C@@H](C2)c5ccc(cc5)OC
CC1=NC2=C([C@@H](C1C(=O)OC(C)C)c3ccc(cc3)OCc4ccccc4)C(=O)C[C@@H](C2)c5ccc(cc5)OC
CCCOc1ccc(cc1OC)[C@@H]2C3=C(C[C@@H](CC3=O)c4ccccc4)NC(=C2C(=O)OCCOC)C
Cc1c(cc(s1)[C@@H]2C3=C(CCCC3=O)NC(=C2C(=O)OC)C)COc4ccc(cc4)Br
CCCOc1ccc(cc1OC)[C@H]2C3=C(C[C@@H](CC3=O)c4ccccc4)NC(=C2C(=O)OCCOC)C
CCCOc1ccc(cc1OC)[C@@H]2C3=C(C[C@H](CC3=O)c4ccccc4)NC(=C2C(=O)OCCOC)C
CCCOc1ccc(cc1OC)[C@H]2C3=C(C[C@H](CC3=O)c4ccccc4)NC(=C2C(=O)OCCOC)C
CC1=NC2=C([C@H](C1C(=O)OCCOC)c3ccc(cc3)OC(=O)C)C(=O)C[C@H](C2)c4ccc(cc4)Cl
CC1=NC2=C([C@@H](C1C(=O)OCCOC)c3ccc(cc3)OC(=O)C)C(=O)C[C@H](C2)c4ccc(cc4)Cl
Cc1c(cc(s1)[C@@H]2C3=C(CCCC3=O)NC(=C2C(=O)OC)C)COc4ccc(cc4)Cl
CC1=NC2=C([C@H](C1C(=O)OCCOC)c3ccc(cc3)OC(=O)C)C(=O)C[C@@H](C2)c4ccc(cc4)Cl
CC1=NC2=C([C@@H](C1C(=O)OCCOC)c3ccc(cc3)OC(=O)C)C(=O)C[C@@H](C2)c4ccc(cc4)Cl
CCOc1ccc(cc1)[C@@H]2C3=C(C[C@@H](CC3=O)c4ccc(cc4)OC)NC(=C2C(=O)OC)C
CCOc1ccc(cc1)[C@H]2C3=C(C[C@@H](CC3=O)c4ccc(cc4)OC)NC(=C2C(=O)OC)C
CCOc1ccc(cc1)[C@@H]2C3=C(C[C@H](CC3=O)c4ccc(cc4)OC)NC(=C2C(=O)OC)C
CCOc1ccc(cc1)[C@H]2C3=C(C[C@H](CC3=O)c4ccc(cc4)OC)NC(=C2C(=O)OC)C
Cc1c(cc(s1)[C@H]2C3=C(CCCC3=O)NC(=C2C(=O)OC)C)COc4ccc(cc4)Cl
Cc1ccc(cc1)[C@@H]2C3=C(C[C@@H](CC3=O)c4ccc(cc4)Cl)NC(=C2C(=O)OCCOC)C
Cc1ccc(cc1)[C@H]2C3=C(C[C@@H](CC3=O)c4ccc(cc4)Cl)NC(=C2C(=O)OCCOC)C
Cc1ccc(cc1)[C@@H]2C3=C(C[C@H](CC3=O)c4ccc(cc4)Cl)NC(=C2C(=O)OCCOC)C

Cc1ccc(cc1)[C@H]2C3=C(C[C@H](CC3=O)c4ccc(cc4)Cl)NC(=C2C(=O)OCCOC)C
CC1=NC2=C([C@H](C1C(=O)OCCOC)c3ccccc3F)C(=O)C[C@H](C2)c4ccc(cc4)Cl
CC1=NC2=C([C@@H](C1C(=O)OCCOC)c3ccccc3F)C(=O)C[C@H](C2)c4ccc(cc4)Cl
CCOC(=O)C1[C@H](C2=C(CC(CC2=O)(C)C)N=C1C)c3cc(c(s3)C)COc4ccc(cc4)Br
CC1=NC2=C([C@H](C1C(=O)OCCOC)c3ccccc3F)C(=O)C[C@@H](C2)c4ccc(cc4)Cl
CC1=NC2=C([C@@H](C1C(=O)OCCOC)c3ccccc3F)C(=O)C[C@@H](C2)c4ccc(cc4)Cl
CC1=NC2=C([C@H](C1C(=O)OC(C)C)c3ccccc3OC(C)C)C(=O)C[C@H](C2)c4ccc(cc4)OC
CC1=NC2=C([C@@H](C1C(=O)OC(C)C)c3ccccc3OC(C)C)C(=O)C[C@H](C2)c4ccc(cc4)OC
CCOC(=O)C1[C@@H](C2=C(CC(CC2=O)(C)C)N=C1C)c3cc(c(s3)C)COc4ccc(cc4)Br
CC1=NC2=C([C@H](C1C(=O)OC(C)C)c3ccccc3OC(C)C)C(=O)C[C@@H](C2)c4ccc(cc4)OC
CC1=NC2=C([C@@H](C1C(=O)OC(C)C)c3ccccc3OC(C)C)C(=O)C[C@@H](C2)c4ccc(cc4)OC
CCc1ccc(cc1)[C@@H]2C3=C(C[C@@H](CC3=O)c4ccccc4)NC(=C2C(=O)OCCOc5ccccc5)C
CCc1ccc(cc1)[C@H]2C3=C(C[C@@H](CC3=O)c4ccccc4)NC(=C2C(=O)OCCOc5ccccc5)C
CCOC(=O)C1[C@H](C2=C(CC(CC2=O)(C)C)N=C1C)c3cc(c(s3)C)COc4ccc(cc4)Cl
CCc1ccc(cc1)[C@@H]2C3=C(C[C@H](CC3=O)c4ccccc4)NC(=C2C(=O)OCCOc5ccccc5)C
CCc1ccc(cc1)[C@H]2C3=C(C[C@H](CC3=O)c4ccccc4)NC(=C2C(=O)OCCOc5ccccc5)C
CC1=NC2=C([C@@H](C1C(=O)OCC(C)C)c3cc(c(c(c3)Br)O)OC)C(=O)C[C@H](C2)c4ccc(cc4)Cl
CC1=NC2=C([C@H](C1C(=O)OCC(C)C)c3cc(c(c(c3)Br)O)OC)C(=O)C[C@@H](C2)c4ccc(cc4)Cl
CCOC(=O)C1[C@@H](C2=C(CC(CC2=O)(C)C)N=C1C)c3cc(c(s3)C)COc4ccc(cc4)Cl
CC1=NC2=C([C@H](C1C(=O)OCCOc3ccccc3)c4ccccc4OC(C)C)C(=O)C[C@H](C2)c5ccccc5
CC1=NC2=C([C@@H](C1C(=O)OCCOc3ccccc3)c4ccccc4OC(C)C)C(=O)C[C@H](C2)c5ccccc5
CC1=NC2=C([C@H](C1C(=O)OCCOc3ccccc3)c4ccccc4OC(C)C)C(=O)C[C@@H](C2)c5ccccc5
CC1=NC2=C([C@@H](C1C(=O)OCCOc3ccccc3)c4ccccc4OC(C)C)C(=O)C[C@@H](C2)c5ccccc5
CCOC(=O)C1[C@H](C2=C(C[C@H](CC2=O)c3ccccc3)N=C1C)c4cccc5c4cccc5
CCOC(=O)C1[C@@H](C2=C(C[C@H](CC2=O)c3ccccc3)N=C1C)c4cccc5c4cccc5
CCOC(=O)C1[C@H](C2=C(CCCC2=O)N=C1C)c3cc(c(s3)C)COc4ccc(cc4)Cl
CCOC(=O)C1[C@H](C2=C(C[C@@H](CC2=O)c3ccccc3)N=C1C)c4cccc5c4cccc5
CCOC(=O)C1[C@@H](C2=C(C[C@@H](CC2=O)c3ccccc3)N=C1C)c4cccc5c4cccc5
CC1=NC2=C([C@H](C1C(=O)OCCOC)c3ccccc3OC)C(=O)C[C@H](C2)c4ccc(cc4)OC
CC1=NC2=C([C@@H](C1C(=O)OCCOC)c3ccccc3OC)C(=O)C[C@H](C2)c4ccc(cc4)OC
CC1=NC2=C([C@H](C1C(=O)OCCOC)c3ccccc3OC)C(=O)C[C@@H](C2)c4ccc(cc4)OC

```
CCOC(=O)C1[C@@H](C2=C(CCCC2=O)N=C1C)c3cc(c(s3)C)COc4ccc(cc4)Cl
CC1=NC2=C([C@@H](C1C(=O)OCCOC)c3ccccc3OC)C(=O)C[C@@H](C2)c4ccc(cc4)OC
CC1=NC2=C([C@H](C1C(=O)OCC(C)C)c3cccc4c3cccc4)C(=O)C[C@H](C2)c5ccccc5
CCOc1ccc(cc1)C(=O)C2=C(C(=O)N([C@@H]2c3ccc(cc3)Br)Cc4ccco4)[O-]
[H]/N=C\1/[C@H](C2=CCCC[C@H]2[C@H](C1(C#N)C#N)c3ccc(c(c3)OC)OCc4c(cc(cc4C)C)C)C#N
CC1=NC2=C([C@@H](C1C(=O)OCC(C)C)c3cccc4c3cccc4)C(=O)C[C@H](C2)c5ccccc5
CC1=NC2=C([C@H](C1C(=O)OCC(C)C)c3cccc4c3cccc4)C(=O)C[C@@H](C2)c5ccccc5
[H]/N=C/1\[C@@H](C2=CCCC[C@H]2[C@@H](C1(C#N)C#N)c3ccc(c(c3)OC)OCc4c(cc(cc4C)C)C)C#N
CC1=NC2=C([C@@H](C1C(=O)OCC(C)C)c3cccc4c3cccc4)C(=O)C[C@@H](C2)c5ccccc5
CC1=NC2=C([C@H](C1C(=O)OCCc3ccccc3)c4cccc(c4OC)OC)C(=O)C[C@H](C2)c5ccc(cc5)Cl
[H]/N=C/1\[C@H](C2=CCCC[C@@H]2[C@H](C1(C#N)C#N)c3ccc(c(c3)OC)OCc4c(cc(cc4C)C)C)C#N
CC1=NC2=C([C@@H](C1C(=O)OCCc3ccccc3)c4cccc(c4OC)OC)C(=O)C[C@H](C2)c5ccc(cc5)Cl
CC1=NC2=C([C@H](C1C(=O)OCCc3ccccc3)c4cccc(c4OC)OC)C(=O)C[C@@H](C2)c5ccc(cc5)Cl
CC1=NC2=C([C@@H](C1C(=O)OCCc3ccccc3)c4cccc(c4OC)OC)C(=O)C[C@@H](C2)c5ccc(cc5)Cl
CC[C@@H](C)OC(=O)C1[C@H](C2=C(C[C@H](CC2=O)c3ccc(cc3)OC)N=C1C)c4ccc(c(c4)OCC)O
[H]/N=C/1\[C@H](C2=CCCC[C@@H]2[C@@H](C1(C#N)C#N)c3ccc(c(c3)OC)OCc4c(cc(cc4C)C)C)C#N
CC[C@H](C)OC(=O)C1[C@H](C2=C(C[C@H](CC2=O)c3ccc(cc3)OC)N=C1C)c4ccc(c(c4)OCC)O
CC[C@@H](C)OC(=O)C1[C@@H](C2=C(C[C@H](CC2=O)c3ccc(cc3)OC)N=C1C)c4ccc(c(c4)OCC)O
CC[C@H](C)OC(=O)C1[C@@H](C2=C(C[C@H](CC2=O)c3ccc(cc3)OC)N=C1C)c4ccc(c(c4)OCC)O
Cc1c(cc(s1)[C@@H]2C3=C(CC(CC3=O)(C)C)NC(=C2C(=O)OC)C)COc4ccc(cc4)Br
CCCOc1ccc(cc1OC)[C@@H]2C3C(=NC(=C2C(=O)OCc4ccccc4)C)C[C@@H](CC3=O)c5ccccc5
CCCOc1ccc(cc1OC)[C@H]2C3C(=NC(=C2C(=O)OCc4ccccc4)C)C[C@@H](CC3=O)c5ccccc5
Cc1c(cc(s1)[C@H]2C3=C(CC(CC3=O)(C)C)NC(=C2C(=O)OC)C)COc4ccc(cc4)Br
CCCOc1ccc(cc1OC)[C@@H]2C3C(=NC(=C2C(=O)OCc4ccccc4)C)C[C@H](CC3=O)c5ccccc5
CCCOc1ccc(cc1OC)[C@H]2C3C(=NC(=C2C(=O)OCc4ccccc4)C)C[C@H](CC3=O)c5ccccc5
CCOc1cc(cc(c1O)Br)[C@@H]2C3C(=NC(=C2C(=O)OCC(C)C)C)C[C@@H](CC3=O)c4ccc(cc4)Cl
CCOc1cc(cc(c1O)Br)[C@H]2C3C(=NC(=C2C(=O)OCC(C)C)C)C[C@@H](CC3=O)c4ccc(cc4)Cl
CCOc1cc(cc(c1O)Br)[C@@H]2C3C(=NC(=C2C(=O)OCC(C)C)C)C[C@H](CC3=O)c4ccc(cc4)Cl
CCCCOc1ccc(cc1)[C@@H]2C(=C(C(=O)N2Cc3ccco3)O)C(=O)c4cccs4
CCOc1cc(cc(c1O)Br)[C@H]2C3C(=NC(=C2C(=O)OCC(C)C)C)C[C@H](CC3=O)c4ccc(cc4)Cl
[H]/N=C\1/[C@H](C2=CCCC[C@H]2[C@H](C1(C#N)C#N)c3c(cc(c(c3C)COc4ccc(cc4)Cl)C)C)C#N
```

```
CCCCOc1ccc(cc1)[C@H]2C(=C(C(=O)N2Cc3ccco3)O)C(=O)c4cccs4
c1cc2c(cc1NC(=O)c3ccc(o3)Br)nc(o2)c4cc(c(c(c4)Br)OC(=O)c5ccc(o5)Br)Br
CCCCOc1ccc(cc1)[C@@H]2C(=C(C(=O)N2Cc3ccco3)[O-])C(=O)c4ccc(o4)C
[H]/N=C\1/[C@H](C2=CCCC[C@H]2[C@@H](C1(C#N)C#N)c3c(cc(c(c3C)COc4ccc(cc4)Cl)C)C)C#N
CCCCOc1ccc(cc1)[C@H]2C(=C(C(=O)N2Cc3ccco3)[O-])C(=O)c4ccc(o4)C
[H]/N=C/1\[C@H](C2=CCCC[C@@H]2[C@H](C1(C#N)C#N)c3c(cc(c(c3C)COc4ccc(cc4)Cl)C)C)C#N
CCCCOc1ccc(cc1)[C@@H]2C(=C(C(=O)N2Cc3ccco3)[O-])C(=O)c4ccc(cc4)Cl
[H]/N=C\1/[C@H](C2=CCCC[C@@H]2[C@@H](C1(C#N)C#N)c3c(cc(c(c3C)COc4ccc(cc4)Cl)C)C)C#N
CCCCOc1ccc(cc1)[C@H]2C(=C(C(=O)N2Cc3ccco3)[O-])C(=O)c4ccc(cc4)Cl
CCCCOc1ccc(cc1)[C@@H]2C(=C(C(=O)N2C[C@@H]3CCCO3)[O-])C(=O)c4ccc(cc4)F
c1ccc2c(c1)c(c(c(=O)o2)C(c3cc4c(cc3[N+](=O)[O-])OCO4)c5c(c6ccccc6oc5=O)[O-])[O-]
CCCCOc1ccc(cc1)[C@H]2C(=C(C(=O)N2C[C@@H]3CCCO3)[O-])C(=O)c4ccc(cc4)F
CCCCOc1ccc(cc1)[C@@H]2C(=C(C(=O)N2C[C@H]3CCCO3)[O-])C(=O)c4ccc(cc4)F
CCCCOc1ccc(cc1)[C@H]2C(=C(C(=O)N2C[C@H]3CCCO3)[O-])C(=O)c4ccc(cc4)F
CCOC(=O)C1=C(N=c2n(c(=O)/c(=C/c3cc(n(c3C)c4ccc(cc4)C)C)/s2)[C@H]1c5ccc(cc5)C)C
Cc1cc(c(cc1COc2ccc(cc2)Cl)[C@@H]3C4=C(CCCC4=O)NC(=C3C(=O)OC)C)C
CCOC(=O)C1=C(N=c2n(c(=O)/c(=C\c3ccc(o3)c4cc(ccc4Cl)Cl)/s2)[C@@H]1c5ccc(cc5)Cl)C
CCOC(=O)C1=C(N=c2n(c(=O)/c(=C\c3ccc(o3)c4cc(ccc4Cl)Cl)/s2)[C@H]1c5ccc(cc5)Cl)C
CCOC(=O)C1=C(N=c2n(c(=O)/c(=C\c3ccc(o3)c4cccc(c4)Cl)/s2)[C@@H]1c5ccc(cc5)Cl)C
CCOC(=O)C1=C(N=c2n(c(=O)/c(=C\c3ccc(o3)c4cccc(c4)Cl)/s2)[C@H]1c5ccc(cc5)Cl)C
c1ccc(cc1)COc2ccc(cc2)[C@H]3C(=C(C(=O)N3Cc4ccco4)[O-])C(=O)c5ccc(cc5)Cl
CCOC(=O)C1=C(N=c2n(c(=O)/c(=C\c3cccs3)/s2)[C@@H]1c4ccc(cc4)Cl)C
CCOC(=O)C1=C(N=c2n(c(=O)/c(=C\c3cccs3)/s2)[C@H]1c4ccc(cc4)Cl)C
Cc1cc(c(cc1COc2ccc(cc2)Cl)[C@H]3C4=C(CCCC4=O)NC(=C3C(=O)OC)C)C
c1ccc(cc1)COc2ccc(cc2)[C@H]3C(=C(C(=O)N3Cc4ccco4)O)C(=O)c5ccc(cc5)Br
Cc1c(c(n(n1)c2ccc(cc2)I)O)/C=C\3/C(=NN(C3=O)c4ccc(cc4)I)C
Cc1ccc(cc1[N+](=O)[O-])S(=O)(=O)N2CC[NH+](CC2)CCN3C(=O)c4cccc5c4c(ccc5)C3=O
c1cc2cccc3c2c(c1)C(=O)N(C3=O)CCCC(=O)Nc4ccc(cc4)OC(F)F
Cc1cc(c(cc1COc2ccc(cc2)Br)[C@@H]3C4=C(CCCC4=O)NC(=C3C(=O)OC)C)C
c1ccc(cc1)N2C(=O)/C(=C/Nc3ccc(cc3)C(=O)N4CCOCC4)/C(=O)NC2=O
COc1cc(ccc1N/C=C/2\C(=O)NC(=O)N(C2=O)c3ccc(cc3)Cl)SC#N
```

```
c1ccc(cc1)CC[C@@H]2CC=C3[C@@H](C2)[C@@]4(c5cc(ccc5NC4=O)Br)C(C(=C3C#N)N)(C#N)C#N
Cc1cc(c(cc1COc2ccc(cc2)Br)[C@H]3C4=C(CCCC4=O)NC(=C3C(=O)OC)C)C
c1ccc(cc1)CC[C@H]2CC=C3[C@@H](C2)[C@@]4(c5cc(ccc5NC4=O)Br)C(C(=C3C#N)N)(C#N)C#N
c1ccc(cc1)CC[C@@H]2CC=C3[C@H](C2)[C@@]4(c5cc(ccc5NC4=O)Br)C(C(=C3C#N)N)(C#N)C#N
c1ccc(cc1)COc2ccc(cc2)[C@@H]3C(=C(C(=O)N3C[C@@H]4CCCO4)O)C(=O)c5ccc(cc5)Br
c1ccc(cc1)CC[C@H]2CC=C3[C@H](C2)[C@@]4(c5cc(ccc5NC4=O)Br)C(C(=C3C#N)N)(C#N)C#N
Cc1cc(c2c(c1)nc(o2)c3ccc(cc3O)NC(=O)c4cc(ccc4I)I)C
c1ccc(cc1)COc2ccc(cc2)[C@H]3C(=C(C(=O)N3C[C@@H]4CCCO4)O)C(=O)c5ccc(cc5)Br
COc1ccc(cc1Br)c2csc(n2)NC(=O)c3ccc4c(c3)OCCO4
CCOC(=O)C1[C@H](C2=C(CCCC2=O)N=C1C)c3ccc(c(c3)OC)OCc4c(cc(cc4C)C)C
c1ccc(cc1)COc2ccc(cc2)[C@@H]3C(=C(C(=O)N3C[C@H]4CCCO4)O)C(=O)c5ccc(cc5)Br
c1ccc(cc1)COc2ccc(cc2)[C@H]3C(=C(C(=O)N3C[C@H]4CCCO4)O)C(=O)c5ccc(cc5)Br
CC1(CC2=C([C@@H](Nc3c2c4ccccc4cc3)c5cc(cc(c5OCC=C)I)I)C(=O)C1)C
CCOC(=O)C1[C@@H](C2=C(CCCC2=O)N=C1C)c3ccc(c(c3)OC)OCc4c(cc(cc4C)C)C
CC1(CC2=C([C@H](Nc3c2c4ccccc4cc3)c5cc(cc(c5OCC=C)I)I)C(=O)C1)C
CC(=O)Oc1cc(c(cc1OC)/C=c\2/c(=O)n3c4ccccc4nc3s2)Br
CCOC(=O)C1[C@H](C2=C(CCCC2=O)N=C1C)c3cc(c(s3)C)COc4ccc(cc4)F
Cc1ccc2c(c1C)nc3n2c(=O)/c(=C\c4cc(ccc4OCc5c(cccc5Cl)F)Cl)/s3
COc1cccc(c1C(=O)N2CCN(CC2)C(=O)c3cc(c(c(c3)OC)OC)OC)OC
CCOC(=O)C1[C@@H](C2=C(CCCC2=O)N=C1C)c3cc(c(s3)C)COc4ccc(cc4)F
[H]/N=C/1\[C@@H](C2=CCCC[C@H]2[C@H](C1(C#N)C#N)c3cc(c(cc3C)C)COc4ccc(cc4)Br)C#N
CCOC(=O)C1=c2n(c(=O)/c(=C\c3ccccc3Cl)/s2)C(=C([C@@H]1c4cccc(c4)Cl)C#N)N
[H]/N=C/1\[C@@H](C2=CCCC[C@H]2[C@@H](C1(C#N)C#N)c3cc(c(cc3C)C)COc4ccc(cc4)Br)C#N
CCOC(=O)C1=c2n(c(=O)/c(=C\c3ccccc3Cl)/s2)C(=C([C@H]1c4cccc(c4)Cl)C#N)N
[H]/N=C/1\[C@H](C2=CCCC[C@@H]2[C@H](C1(C#N)C#N)c3cc(c(cc3C)C)COc4ccc(cc4)Br)C#N
[H]/N=C/1\[C@H](C2=CCCC[C@@H]2[C@@H](C1(C#N)C#N)c3cc(c(cc3C)C)COc4ccc(cc4)Br)C#N
CCOC(=O)C1[C@H](C2=C(CC(CC2=O)(C)C)N=C1C)c3cc(c(s3)C)COc4ccc(cc4)F
c1cc(c(cc1[N+](=O)[O-])NC(=O)c2cc(cc(c2)[N+](=O)[O-])C(=O)Nc3cc(ccc3Cl)[N+](=O)[O-])Cl
COc1ccc(c(c1)[N+](=O)[O-])NC(=O)c2cc(cc(c2)[N+](=O)[O-])C(=O)Nc3ccc(cc3[N+](=O)[O-])OC
CCOC(=O)C1[C@@H](C2=C(CC(CC2=O)(C)C)N=C1C)c3cc(c(s3)C)COc4ccc(cc4)F
CCCCN(CCCC)S(=O)(=O)c1ccc-2c(c1)C(c3c2ccc(c3)S(=O)(=O)N(CCCC)CCCC)O
```

CCCOc1ccc(cc1)[C@@H]2C(=C(C(=O)N2Cc3ccco3)[O-])C(=O)c4ccc(cc4)C
Cc1c(c(=O)n(n1C)c2ccccc2)NC(=O)[C@@H](CCSC)NC(=O)c3ccccc3NC(=O)c4ccc(cc4)OC
Cc1c(c(=O)n(n1C)c2ccccc2)NC(=O)[C@H](CCSC)NC(=O)c3ccccc3NC(=O)c4ccc(cc4)OC
CCCOc1ccc(cc1)[C@H]2C(=C(C(=O)N2Cc3ccco3)[O-])C(=O)c4ccc(cc4)C
Cc1cc(c(cc1COc2ccc(cc2)Br)[C@@H]3C4=C(CC(CC4=O)(C)C)NC(=C3C(=O)OC)C)C
c1ccc(c(c1)[C@@H]2[C@@H]3C[C@@H]([C@@H]([C@@H]3c4cc(ccc4N2)Cl)Cl)Sc5ccccc5[N+](=O)[O-])Br
c1ccc(c(c1)[C@H]2[C@@H]3C[C@@H]([C@@H]([C@@H]3c4cc(ccc4N2)Cl)Cl)Sc5ccccc5[N+](=O)[O-])Br
c1ccc(c(c1)[C@@H]2[C@H]3C[C@@H]([C@@H]([C@@H]3c4cc(ccc4N2)Cl)Cl)Sc5ccccc5[N+](=O)[O-])Br
c1ccc(c(c1)[C@H]2[C@H]3C[C@@H]([C@@H]([C@@H]3c4cc(ccc4N2)Cl)Cl)Sc5ccccc5[N+](=O)[O-])Br
Cc1cc(c(cc1COc2ccc(cc2)Br)[C@H]3C4=C(CC(CC4=O)(C)C)NC(=C3C(=O)OC)C)C
Cc1cc(ccc1OCC=C)C(=O)C2=C(C(=O)N([C@@H]2c3ccc(cc3)OCc4ccccc4)CC[NH+](C)C)O
CCOC(=O)C1[C@H](C2=C(CC(CC2=O)(C)C)N=C1C)c3ccc(c(c3)OC)OCc4c(cc(cc4C)C)C
Cc1cc(ccc1OCC=C)C(=O)C2=C(C(=O)N([C@H]2c3ccc(cc3)OCc4ccccc4)CC[NH+](C)C)O
c1ccc(cc1)COc2ccc(cc2)[C@@H]3C(=C(C(=O)N3CC[NH+]4CCOCC4)O)C(=O)c5cccs5
c1ccc(c(c1)[N+](=O)[O-])S[C@@H]2C[C@@H]3[C@@H](Nc4c(ccc(c4[C@@H]3[C@@H]2Cl)[N+](=O)[O-])C(=O)[O-
])c5ccc(cc5)[N+](=O)[O-]
c1ccc(c(c1)[N+](=O)[O-])S[C@H]2C[C@@H]3[C@@H](Nc4c(ccc(c4[C@@H]3[C@@H]2Cl)[N+](=O)[O-])C(=O)[O-])c5ccc(cc5)[N+](=C
]
CCOC(=O)C1[C@@H](C2=C(CC(CC2=O)(C)C)N=C1C)c3ccc(c(c3)OC)OCc4c(cc(cc4C)C)C
c1ccc(c(c1)[N+](=O)[O-])S[C@@H]2C[C@H]3[C@@H](Nc4c(ccc(c4[C@@H]3[C@@H]2Cl)[N+](=O)[O-])C(=O)[O-])c5ccc(cc5)[N+](=C
]
c1ccc(cc1)COc2ccc(cc2)[C@H]3C(=C(C(=O)N3CC[NH+]4CCOCC4)O)C(=O)c5cccs5
c1ccc(c(c1)[N+](=O)[O-])S[C@H]2C[C@H]3[C@@H](Nc4c(ccc(c4[C@@H]3[C@@H]2Cl)[N+](=O)[O-])C(=O)[O-])c5ccc(cc5)[N+](=O)
Cc1ccc(o1)C(=O)C2=C(C(=O)N([C@@H]2c3ccc(cc3)OCc4ccccc4)CC[NH+]5CCOCC5)O
Cc1cc(c(c(c1)C)COc2ccc(cc2OC)[C@@H]3C4=C(CC(CC4=O)(C)C)NC(=C3C(=O)OC)C)C
Cc1ccc(o1)C(=O)C2=C(C(=O)N([C@H]2c3ccc(cc3)OCc4ccccc4)CC[NH+]5CCOCC5)O
c1ccc(cc1)COc2ccc(cc2)[C@@H]3C(=C(C(=O)N3CC[NH+]4CCOCC4)O)C(=O)c5ccc(cc5)Cl
Cc1cc(c(c(c1)C)COc2ccc(cc2OC)[C@H]3C4=C(CC(CC4=O)(C)C)NC(=C3C(=O)OC)C)C
c1ccc(cc1)COc2ccc(cc2)[C@H]3C(=C(C(=O)N3CC[NH+]4CCOCC4)O)C(=O)c5ccc(cc5)Cl
Cc1cc(c(cc1COc2ccc(cc2)Cl)[C@@H]3C4=C(CC(CC4=O)(C)C)NC(=C3C(=O)OC)C)C
CCCCOC(=O)c1cc(cc\2c1-c3c(cc(cc3[N+](=O)[O-])[N+](=O)[O-])/C2=C\c4ccco4)[N+](=O)[O-]
CCCCOC(=O)c1cc(cc2c1-c3c(cc(cc3[N+](=O)[O-])[N+](=O)[O-])C2=Cc4ccccc4O)[N+](=O)[O-]

CC(C)CCOc1ccc(cc1OC)[C@@H]2C(=C(C(=O)N2CC[NH+]3CCOCC3)O)C(=O)c4cccs4
Cc1cc(c(cc1COc2ccc(cc2)Cl)[C@H]3C4=C(CC(CC4=O)(C)C)NC(=C3C(=O)OC)C)C
CC(C)CCOc1ccc(cc1OC)[C@H]2C(=C(C(=O)N2CC[NH+]3CCOCC3)O)C(=O)c4cccs4
c1ccc(cc1)CN(c2ccccc2)S(=O)(=O)c3ccc-4c(c3)Cc5c4ccc(c5)S(=O)(=O)N(Cc6ccccc6)c7ccccc7
c1ccc(cc1)CN(c2ccccc2)S(=O)(=O)c3ccc-4c(c3)C(=O)c5c4ccc(c5)S(=O)(=O)N(Cc6ccccc6)c7ccccc7
Cc1ccc(o1)C(=O)C2=C(C(=O)N([C@@H]2c3ccc(c(c3)OC)OCCC(C)C)CC[NH+]4CCOCC4)O
Cc1ccc(cc1)S(=O)(=O)N(C[C@@H](Cn2c3ccc(cc3c4c2ccc(c4)Br)Br)O)c5ccc(cc5)NC(=O)C
Cc1cc(c(c(c1)C)COc2ccc(cc2OC)[C@@H]3C4=C(CCCC4=O)NC(=C3C(=O)OC)C)C
Cc1ccc(o1)C(=O)C2=C(C(=O)N([C@H]2c3ccc(c(c3)OC)OCCC(C)C)CC[NH+]4CCOCC4)O
Cc1ccc(cc1)S(=O)(=O)N(C[C@H](Cn2c3ccc(cc3c4c2ccc(c4)Br)Br)O)c5ccc(cc5)NC(=O)C
Cc1ccc(cc1)S(=O)(=O)N(C[C@@H]2CCCO2)C[C@@H](Cn3c4ccc(cc4c5c3ccc(c5)Br)Br)O
CCOc1ccc(cc1)C(=O)C2=C(C(=O)N([C@@H]2c3ccc(cc3)OCC=C)CC[NH+]4CCOCC4)O
Cc1ccc(cc1)S(=O)(=O)N(C[C@H]2CCCO2)C[C@@H](Cn3c4ccc(cc4c5c3ccc(c5)Br)Br)O
CCOc1ccc(cc1)C(=O)C2=C(C(=O)N([C@H]2c3ccc(cc3)OCC=C)CC[NH+]4CCOCC4)O
Cc1cc(c(c(c1)C)COc2ccc(cc2OC)[C@H]3C4=C(CCCC4=O)NC(=C3C(=O)OC)C)C
CCCOc1ccc(cc1)[C@@H]2C(=C(C(=O)N2CCC[NH+]3CCOCC3)O)C(=O)c4ccc(cc4)Br
CCCOc1ccc(cc1)[C@H]2C(=C(C(=O)N2CCC[NH+]3CCOCC3)O)C(=O)c4ccc(cc4)Br
Cc1c(c(=O)n([nH]1)c2ccccc2)[C@@H](c3ccc(cc3)OC(=O)C)c4c(nn(c4O)c5ccccc5)C
CC(C)CCOc1ccc(cc1OC)[C@@H]2C(=C(C(=O)N2CCC[NH+]3CCOCC3)O)C(=O)c4ccc(cc4)Cl
CCCN(CCC)C(=O)[C@@H](C(C)C)NC(=O)c1ccccc1NC(=O)c2ccc(cc2)OC
CCCN(CCC)C(=O)[C@H](C(C)C)NC(=O)c1ccccc1NC(=O)c2ccc(cc2)OC
CC(C)CCOc1ccc(cc1OC)[C@H]2C(=C(C(=O)N2CCC[NH+]3CCOCC3)O)C(=O)c4ccc(cc4)Cl
c1ccc(cc1)COc2ccc(cc2)[C@@H]3C(=C(C(=O)N3CCC[NH+]4CCOCC4)O)C(=O)c5ccc(cc5)Br
c1ccc(cc1)COc2ccc(cc2)[C@H]3C(=C(C(=O)N3CCC[NH+]4CCOCC4)O)C(=O)c5ccc(cc5)Br
CCOC(=O)C1[C@H](C2=C(CCCC2=O)N=C1C)c3cn(nc3c4ccccc4)Cc5ccc(cc5Cl)Cl
CCOC(=O)C1[C@@H](C2=C(CCCC2=O)N=C1C)c3cn(nc3c4ccccc4)Cc5ccc(cc5Cl)Cl
CC(C)[C@@H](C(=O)Nc1ccccc1C(=O)OC)NC(=O)c2ccccc2NC(=O)c3ccc(cc3)OC
CC(C)[C@H](C(=O)Nc1ccccc1C(=O)OC)NC(=O)c2ccccc2NC(=O)c3ccc(cc3)OC
Cc1ccc(cc1)OCC(=O)NNC(=O)[C@@H](C(C)C)NC(=O)c2ccccc2NC(=O)c3ccc(cc3)OC
CC1=NC2=C([C@H](C1C(=O)OC)c3cn(nc3c4ccccc4)Cc5ccc(cc5Cl)Cl)C(=O)CCC2
Cc1ccc(cc1)OCC(=O)NNC(=O)[C@H](C(C)C)NC(=O)c2ccccc2NC(=O)c3ccc(cc3)OC

```
CC[C@@H](C)[C@@H](C(=O)NCC(C)C)NC(=O)c1ccccc1NC(=O)c2ccc(cc2)OC
CC[C@H](C)[C@@H](C(=O)NCC(C)C)NC(=O)c1ccccc1NC(=O)c2ccc(cc2)OC
CC[C@@H](C)[C@H](C(=O)NCC(C)C)NC(=O)c1ccccc1NC(=O)c2ccc(cc2)OC
CC[C@H](C)[C@H](C(=O)NCC(C)C)NC(=O)c1ccccc1NC(=O)c2ccc(cc2)OC
CCCCCCCNC(=O)[C@@H](Cc1ccc(cc1)O)NC(=O)c2ccccc2NC(=O)c3ccc(cc3)OC
CCCCCCCNC(=O)[C@H](Cc1ccc(cc1)O)NC(=O)c2ccccc2NC(=O)c3ccc(cc3)OC
COc1ccccc1N2CCN(CC2)C(=O)CCN3C(=O)c4cccc5c4c(ccc5)C3=O
CCCCCCCCNc1cc(c(c2n1c3ccccc3n2)C#N)CCC
Cc1ccc(cc1)Cn2c3c(cn2)c(ncn3)N4CC[NH+](CC4)C(c5ccccc5)c6ccccc6
c1cc(ccc1N(c2c(nc3c(n2)non3)N(c4ccc(cc4)[N+](=O)[O-])[N+](=O)[O-])[N+](=O)[O-])[N+](=O)[O-]
c1cc(ccc1[C@@H]2C[C@@H](n3c(c(c(n3)C(=O)NCCCn4ccnc4)Cl)N2)C(F)(F)F)Br
c1cc(ccc1Cn2c3c(cn2)c(ncn3)N4CC[NH+](CC4)Cc5ccc(cc5)Cl)Cl
c1ccc(c(c1)Nc2c(nc3c(n2)non3)Nc4ccccc4[N+](=O)[O-])[N+](=O)[O-]
c1cc(ccc1Cn2c3c(cn2)c(ncn3)N4CC[NH+](CC4)CCOc5ccc(cc5)Cl)Cl
c1cc(ccc1[C@H]2C[C@@H](n3c(c(c(n3)C(=O)NCCCn4ccnc4)Cl)N2)C(F)(F)F)Br
CCOC(=O)C1[C@H](C(=C(N=C1C)C)C(=O)OCC)c2cccc(c2)I
CC(C)c1cc(n2c3ccccc3nc2c1C#N)N4CC[NH+](CC4)Cc5ccc(cc5)Cl
c1cc(ccc1[C@@H]2C[C@H](n3c(c(c(n3)C(=O)NCCCn4ccnc4)Cl)N2)C(F)(F)F)Br
CC\1=NN(C(=O)/C1=C\Nc2ccc(cc2)S(=O)(=O)[N-]c3cc(nc(n3)OC)OC)c4ccc(cc4)I
CCOC(=O)c1c2c(sc1NC(=O)c3ccc(cc3)N/C=C\4/C(=O)NC(=O)N(C4=O)c5ccccc5)CCCC2
c1cc(ccc1[C@H]2C[C@H](n3c(c(c(n3)C(=O)NCCCn4ccnc4)Cl)N2)C(F)(F)F)Br
c1ccc(c(c1)C(=O)N2[C@@H](CC(=N2)c3ccc(cc3)Br)c4ccc(cc4)Br)Br
c1ccc(c(c1)C(=O)N2[C@H](CC(=N2)c3ccc(cc3)Br)c4ccc(cc4)Br)Br
CCOC(=O)C1[C@H](C2=C(CC(CC2=O)(C)C)N=C1C)c3ccc(c(c3)COc4ccc(cc4)Cl)OC
COc1ccc(cc1)[C@@H]2CC(=NN2C(=O)CCC(=O)Nc3ccc(cc3)Cl)c4ccc(cc4)Br
COc1ccc(cc1)[C@H]2CC(=NN2C(=O)CCC(=O)Nc3ccc(cc3)Cl)c4ccc(cc4)Br
Cc1cc(c(n1c2cccc(c2)C(F)(F)F)C)C(=O)CSc3nc4c(c(c(s4)C)c5ccccc5)c(=O)n3c6ccccc6
CCOC(=O)C1[C@@H](C2=C(CC(CC2=O)(C)C)N=C1C)c3ccc(c(c3)COc4ccc(cc4)Cl)OC
c1ccc(c(c1)C(=O)N2[C@@H](CC(=N2)c3ccc(cc3)Br)c4ccc(cc4)Br)Cl
c1ccc(c(c1)C(=O)N2[C@H](CC(=N2)c3ccc(cc3)Br)c4ccc(cc4)Br)Cl
CCOC(=O)C1[C@H](C2=C(CC(CC2=O)(C)C)N=C1C)c3cn(nc3c4ccccc4)Cc5ccc(cc5)Br
```

```
Cc1c(sc(c1[C@@H](c2cccnc2)[NH+]3CCN(CC3)c4cccc[nH+]4)NC(=O)c5ccco5)C
Cc1c(sc(c1[C@H](c2cccnc2)[NH+]3CCN(CC3)c4cccc[nH+]4)NC(=O)c5ccco5)C
CCOC(=O)C1[C@@H](C2=C(CC(CC2=O)(C)C)N=C1C)c3cn(nc3c4ccccc4)Cc5ccc(cc5)Br
Cc1c(sc(c1[C@H](c2ccc(cc2)OC(F)F)[NH+]3CCOCC3)NC(=O)c4ccco4)C
COc1ccccc1NC(=O)CCC(=O)N2[C@@H](CC(=N2)c3ccc(cc3)Br)c4ccc(cc4)Br
COc1ccccc1NC(=O)CCC(=O)N2[C@H](CC(=N2)c3ccc(cc3)Br)c4ccc(cc4)Br
CCOC(=O)C[NH+]1[C@@H](c2cc(ccc2N[C@@H]1c3ccccc3OC)Br)c4ccccc4
CC1=NC2=C([C@H](C1C(=O)OC)c3ccc(c(c3)COc4ccc(cc4)Cl)OC)C(=O)CC(C2)(C)C
Cc1c(sc(c1[C@@H](c2ccc(cc2)Cl)[NH+]3CCOCC3)NC(=O)c4ccco4)C
Cc1c(sc(c1[C@H](c2ccc(cc2)Cl)[NH+]3CCOCC3)NC(=O)c4ccco4)C
c1cc(cc(c1)Br)N2C(=O)/C(=C\c3cc(cc(c3O)I)Br)/C(=O)NC2=S
Cc1cc(ccc1N2C(=O)/C(=C\c3cc(c(c(c3)I)OCc4ccc(cc4)Cl)OC)/C(=O)NC2=S)Br
Cc1cc(ccc1N2C(=O)/C(=C\c3cc(cc(c3Cl)Cl)Cl)/C(=O)NC2=S)Br
CC1=NC2=C([C@@H](C1C(=O)OC)c3ccc(c(c3)COc4ccc(cc4)Cl)OC)C(=O)CC(C2)(C)C
Cc1cc(c(n1c2c(c3c(s2)CCCC3)C#N)C)/C=c\4/c(=O)n5c(=NC(=C([C@@H]5c6c7ccccc7ccc6OC)C(=O)C)C)s4
Cc1cc(c(n1c2c(c3c(s2)CCCC3)C#N)C)/C=c\4/c(=O)n5c(=NC(=C([C@H]5c6c7ccccc7ccc6OC)C(=O)C)C)s4
Cc1c(sc(c1[C@@H](c2cccc(c2)Cl)[NH+]3CCOCC3)NC(=O)c4ccco4)C
c1cc(cc(c1)Br)N2C(=O)/C(=C\c3cc(c(cc3O)O)Br)/C(=O)NC2=S
Cc1c(sc(c1[C@H](c2cccc(c2)Cl)[NH+]3CCOCC3)NC(=O)c4ccco4)C
CCOc1cccc(c1)N2C(=O)/C(=C/c3cc(n(c3C)c4ccc(c(c4)C)C)C)/C(=NC2=S)[O-]
Cc1c(sc(c1[C@@H](c2cccc(c2)Br)[NH+]3CCOCC3)NC(=O)c4ccco4)C
CCOC(=O)C1[C@H](C2=C(CCCC2=O)N=C1C)c3ccc(c(c3)COc4ccc(cc4)Cl)OC
CCOc1cccc(c1)N2C(=O)/C(=C/c3cc(n(c3C)C4CCCCC4)C)/C(=NC2=S)[O-]
Cc1c(sc(c1[C@H](c2cccc(c2)Br)[NH+]3CCOCC3)NC(=O)c4ccco4)C
CC(=O)Nc1c(c2c(s1)CCCC2)[C@@H](c3ccc(cc3)OC(F)F)[NH+]4CCOCC4
CC(=O)Nc1ccc(cc1)OS(=O)(=O)c2cccc3c2C(=O)c4ccccc4C3=O
CCOC(=O)C1[C@@H](C2=C(CCCC2=O)N=C1C)c3ccc(c(c3)COc4ccc(cc4)Cl)OC
CC(=O)Nc1c(c2c(s1)CCCC2)[C@H](c3ccc(cc3)OC(F)F)[NH+]4CCOCC4
CCCCCCC1=NN([C@@](C1)(C(F)(F)F)O)C(=O)c2ccc(cc2)Br
CC(=O)Nc1c(c2c(s1)CCCC2)[C@H](c3ccc(cc3)C(F)(F)F)[NH+]4CCOCC4
CCCCCCC1=NN([C@](C1)(C(F)(F)F)O)C(=O)c2ccc(cc2)Br
```

```
CCOC(=O)C1[C@H](C2=C(CC(CC2=O)(C)C)N=C1C)c3ccc(o3)COc4ccc(cc4)Br
CC(=O)Nc1c(c2c(s1)CCCC2)[C@@H](c3cccc(c3)Br)[NH+]4CCOCC4
CC(=O)Nc1c(c2c(s1)CCCC2)[C@H](c3cccc(c3)Br)[NH+]4CCOCC4
CCOC(=O)c1c2c(sc1N(Cc3ccccc3O)C(=O)CO/N=C(\C)/c4cccs4)CCCC2
CCOC(=O)C1[C@@H](C2=C(CC(CC2=O)(C)C)N=C1C)c3ccc(o3)COc4ccc(cc4)Br
c1ccc(cc1)c2cc(nc(c2C#N)SCC(=O)N(c3ccccc3)c4ccccc4)c5ccccc5
Cc1cccc(c1OC(=O)CSc2c(c(cc(n2)c3ccccc3)c4ccccc4)C#N)C
CC1=NC2=C([C@H](C1C(=O)OC)c3ccc(o3)COc4ccc(cc4)Br)C(=O)CCC2
C[C@]12CC[C@](C1(C)C)(CC2=O)C(=O)Nc3ccc(cc3)Cc4ccc(cc4)NC(=O)[C@]56CC[C@](C5(C)C)(C(=O)C6)C
C[C@]12CC[C@](C1(C)C)(CC2=O)C(=O)Nc3ccc(cc3)Cc4ccc(cc4)NC(=O)[C@@]56CC[C@@](C5(C)C)(C(=O)C6)C
CC1([C@]2(CC[C@@]1([C@@H]2Br)C(=O)N3CCN(CC3)c4ccc(cc4)F)C(Br)Br)C
CC1=NC2=C([C@@H](C1C(=O)OC)c3ccc(o3)COc4ccc(cc4)Br)C(=O)CCC2
CC1([C@@]2(CC[C@]1([C@@H]2Br)C(=O)N3CCN(CC3)c4ccc(cc4)F)C(Br)Br)C
Cc1ccc(c(c1)NC(=O)CSc2c(c(cc(n2)c3ccccc3)c4ccccc4)C#N)C
c1cc(cc(c1)Cl)N2[C@@H]3CS(=O)(=O)C[C@@H]3N(C2=O)c4ccc(cc4)Br
CC1=NC2=C([C@H](C1C(=O)OC)c3ccc(c(c3)COc4ccccc4Br)OC)C(=O)CCC2
c1cc(cc(c1)Cl)N2[C@@H]3CS(=O)(=O)C[C@H]3N(C2=O)c4ccc(cc4)Br
COc1ccc(cc1)c2cc3cc(c(cc3c(n2)Cc4cccc5c4cccc5)OC)OC
c1cc(ccc1N2[C@@H]3CS(=O)(=O)C[C@@H]3N(C2=O)c4ccc(c(c4)Cl)Cl)Br
CCCCCCc1cc2cc(c(=O)oc2cc1O)c3cn4c5ccc(cc5sc4n3)OC
c1cc(ccc1N2[C@@H]3CS(=O)(=O)C[C@H]3N(C2=O)c4ccc(c(c4)Cl)Cl)Br
Cc1ccc2c(c1)sc(n2)N3[C@@H](C(=C(C3=O)[O-])C(=O)c4ccc(c(c4)C)OC)c5cccc(c5)Br
CC1=NC2=C([C@@H](C1C(=O)OC)c3ccc(c(c3)COc4ccccc4Br)OC)C(=O)CCC2
Cc1ccc(cc1)c2csc(n2)c\3cc4cc(ccc4o/c3=[NH+]\O)Br
Cc1ccc2c(c1)sc(n2)N3[C@H](C(=C(C3=O)[O-])C(=O)c4ccc(c(c4)C)OC)c5cccc(c5)Br
CC1=NC2=C([C@H](C1C(=O)OC)c3cn(nc3c4ccccc4)Cc5ccc(cc5)Br)C(=O)CC(C2)(C)C
CCc1ccc2c(c1)sc(n2)N3[C@@H](C(=C(C3=O)[O-])C(=O)c4ccc(cc4)Cl)c5ccc(cc5)C(C)(C)C
CCc1ccc2c(c1)sc(n2)N3[C@H](C(=C(C3=O)[O-])C(=O)c4ccc(cc4)Cl)c5ccc(cc5)C(C)(C)C
CC1=NC2=C([C@@H](C1C(=O)OC)c3cn(nc3c4ccccc4)Cc5ccc(cc5)Br)C(=O)CC(C2)(C)C
CCc1ccc2c(c1)sc(n2)N3[C@@H](C(=C(C3=O)[O-])C(=O)c4ccc(cc4)Cl)c5ccc(cc5)Br
CCc1ccc2c(c1)sc(n2)N3[C@H](C(=C(C3=O)[O-])C(=O)c4ccc(cc4)Cl)c5ccc(cc5)Br
```

```
CCc1ccc2c(c1)sc(n2)N3[C@@H](C(=C(C3=O)[O-])C(=O)c4ccc(cc4)F)c5ccc(cc5)Br
CC1=NC2=C([C@H](C1C(=O)OC)c3ccc(o3)COc4ccc(cc4)Br)C(=O)CC(C2)(C)C
CCOC(=O)c1c2c(sc1N/C=C\3/C(=NN(C3=O)c4ccc(cc4)I)C)CCCC2
CCc1ccc2c(c1)sc(n2)N3[C@H](C(=C(C3=O)[O-])C(=O)c4ccc(cc4)F)c5ccc(cc5)Br
Cc1cc(cc(c1)NC(=O)CSc2c(c(cc(n2)c3ccccc3)c4ccccc4)C#N)C
Cc1ccc2ccccc2c1NC(=O)CSc3c(c(cc(n3)c4ccccc4)c5ccccc5)C#N
CC1=NC2=C([C@@H](C1C(=O)OC)c3ccc(o3)COc4ccc(cc4)Br)C(=O)CC(C2)(C)C
Cc1cc(cc(c1)NC(=O)c2c(c3c(cc(nc3s2)c4ccccc4)c5ccccc5)N)C
Cc1ccc(c(c1)Br)NC(=O)CSc2nnc(o2)c3ccccc3O
CCc1ccc2c(c1)sc(n2)N3[C@@H](C(=C(C3=O)[O-])C(=O)c4ccccc4)c5ccc(cc5)Br
[H]/N=C/1\[C@@H](C2=CCCC[C@H]2[C@H](C1(C#N)C#N)c3ccc(c(c3)OC)COc4ccc(cc4)F)C#N
Cc1cccc(c1C)NC(=O)CSc2c(c(cc(n2)c3ccccc3)c4ccccc4)C#N
CCc1ccc2c(c1)sc(n2)N3[C@H](C(=C(C3=O)[O-])C(=O)c4ccccc4)c5ccc(cc5)Br
[H]/N=C/1\[C@@H](C2=CCCC[C@H]2[C@@H](C1(C#N)C#N)c3ccc(c(c3)OC)COc4ccc(cc4)F)C#N
CCOC(=O)CCSc1c(c(cc(n1)c2ccc(cc2)C)c3ccc(cc3)OC)C#N
[H]/N=C/1\[C@H](C2=CCCC[C@@H]2[C@H](C1(C#N)C#N)c3ccc(c(c3)OC)COc4ccc(cc4)F)C#N
Cc1ccc(cc1)c2cc(c(c(n2)SCC(=O)Nc3cc(ccc3C)C)C#N)c4ccc(c(c4)OC)OC
[H]/N=C/1\[C@H](C2=CCCC[C@@H]2[C@@H](C1(C#N)C#N)c3ccc(c(c3)OC)COc4ccc(cc4)F)C#N
Cc1ccc(cc1)c2cc(c3c(c(sc3n2)C(=O)Nc4cc(ccc4C)C)N)c5ccc(cc5)OC
Cc1ccc(cc1)c2cc(c(c(n2)SCC(=O)Nc3ccc(cc3C)C)C#N)c4ccc(c(c4)OC)OC
Cc1ccc(cc1)c2cc(c(c(n2)SCC(=O)Nc3c(cccc3C)C)C#N)c4ccc(c(c4)OC)OC
Cc1ccc(cc1)c2cc(c(c(n2)SCC(=O)Nc3cccc(c3C)C)C#N)c4ccc(c(c4)OC)OC
CCOC(=O)C1[C@H](C2=C(CCCC2=O)N=C1C)c3ccc(c(c3)COc4ccccc4Br)OC
c1ccc(c(c1)c2nnc(o2)SCC(=O)Nc3ccccc3Br)Br
Cc1ccc(cc1)c2cc(c(c(n2)SCC(=O)Nc3cc(ccc3C)C)C#N)c4ccccc4
CCOC(=O)C1[C@@H](C2=C(CCCC2=O)N=C1C)c3ccc(c(c3)COc4ccccc4Br)OC
Cc1ccc(cc1)c2cc(c3c(c(sc3n2)C(=O)Nc4ccccc4C)N)c5ccccc5
CC1=NC2=C([C@H](C1C(=O)OC)c3ccc(c(c3)COc4ccc(cc4)Cl)OC)C(=O)CCC2
Cc1ccc(cc1)c2cc(c3c(c(sc3n2)C(=O)Nc4ccc(cc4C)C)N)c5ccc(cc5)OC
Cc1c(sc(n1)N2[C@@H](C(=C(C2=O)O)C(=O)c3cc4ccccc4o3)c5ccccc5)C(=O)C
Cc1ccc(cc1)c2cc(nc(c2C#N)SCC(=O)Nc3ccccc3OC)c4ccccc4
```

```
Cc1ccc(cc1)c2cc(nc(c2C#N)SCC(=O)Nc3ccccc3C)c4ccccc4
Cc1ccc(cc1)c2cc(nc(c2C#N)SCC(=O)Nc3ccc(c(c3)C)C)c4ccccc4
CC1=NC2=C([C@@H](C1C(=O)OC)c3ccc(c(c3)COc4ccc(cc4)Cl)OC)C(=O)CCC2
Cc1ccc(cc1)c2cc(nc(c2C#N)SCC(=O)Nc3cccc(c3C)C)c4ccccc4
Cc1ccc(cc1)c2cc(nc(c2C#N)SCC(=O)Nc3ccccc3C(F)(F)F)c4ccccc4
Cc1ccc(cc1)c2cc(nc(c2C#N)SCC(=O)Nc3cccc(c3)C)c4ccccc4
Cc1cccc(c1)NC(=O)CSc2c(c(cc(n2)c3ccccc3)c4ccc(cc4)C(C)(C)C)C#N
Cc1c(sc(n1)N2[C@H](C(=C(C2=O)O)C(=O)c3cc4ccccc4o3)c5ccc(cc5)F)C(=O)C
CC1=NC2=C([C@H](C1C(=O)OC)c3cn(nc3c4ccccc4)Cc5ccc(cc5)Br)C(=O)CCC2
Cc1ccc(cc1)c2cc(nc3c2c(c(s3)C(=O)Nc4cccc(c4C)C)N)c5ccccc5
Cc1ccc(cc1)c2cc(nc(c2C#N)SCC(=O)Nc3cc(ccc3C)C)c4ccccc4
Cc1cccc(c1C)NC(=O)CSc2c(c(cc(n2)c3ccccc3)c4ccc(cc4)C(C)(C)C)C#N
Cc1ccc(cc1)c2cc(nc(c2C#N)SCC(=O)Nc3ccccc3c4ccccc4)c5ccccc5
CC(C)(C)c1ccc(cc1)c2cc(nc(c2C#N)SCC(=O)Nc3ccc(cc3)Oc4ccccc4)c5ccccc5
CC(C)(C)c1ccc(cc1)c2cc(nc(c2C#N)SCC(=O)Nc3ccc(cc3)Cl)c4ccccc4
Cc1ccccc1NC(=O)CSc2c(c(cc(n2)c3ccccc3)c4ccc(cc4)C(C)(C)C)C#N
CC1=NC2=C([C@@H](C1C(=O)OC)c3cn(nc3c4ccccc4)Cc5ccc(cc5)Br)C(=O)CCC2
Cc1ccc(cc1)NC(=O)CSc2c(c(cc(n2)c3ccccc3)c4ccc(cc4)C(C)(C)C)C#N
Cc1ccc(c(c1)NC(=O)CSc2c(c(cc(n2)c3ccccc3)c4ccc(cc4)C(C)(C)C)C#N)C
CCc1ccccc1NC(=O)CSc2c(c(cc(n2)c3ccccc3)c4ccc(cc4)C)C#N
Cc1c(sc(n1)N2[C@@H](C(=C(C2=O)O)C(=O)c3cc4ccccc4o3)c5cccs5)C(=O)C
CCc1ccccc1NC(=O)CSc2c(c(cc(n2)c3ccc(cc3)Cl)c4ccc(cc4)OC)C#N
Cc1ccc(cc1)c2cc(nc(c2C#N)SCC(=O)c3ccc(cc3)c4ccccc4)c5ccccc5
CCOC(=O)c1c(c(sc1NC(=O)CSc2c(c(cc(n2)c3ccccc3)c4ccc(cc4)C)C#N)C(=O)C)C
CC1=NC2=C([C@H](C1C(=O)OC)c3ccc(c(c3)COc4ccccc4Br)OC)C(=O)CC(C2)(C)C
Cc1ccc(cc1)c2cc(nc(c2C#N)SCC(=O)Nc3ccc(cc3)OC)c4ccccc4
CCCCCOc1ccc(cc1OC)[C@@H]2C(=C(C(=O)N2c3nc(c(s3)C(=O)C)C)O)C(=O)c4cc5ccccc5o4
CC(C)(C)c1ccc(cc1)c2cc(nc(c2C#N)SCC(=O)Nc3ccc(cc3)OC)c4ccccc4
CC(C)(C)c1ccc(cc1)c2cc(nc(c2C#N)SCC(=O)Nc3ccccc3OC)c4ccccc4
CCOC(=O)c1c(c(sc1NC(=O)CSc2c(c(cc(n2)c3ccccc3)c4ccc(cc4)C(C)(C)C)C#N)C)C
CCCCCOc1ccc(cc1OC)[C@H]2C(=C(C(=O)N2c3nc(c(s3)C(=O)C)C)O)C(=O)c4cc5ccccc5o4
```

```
Cc1ccc(cc1)c2cc(nc(c2C#N)SCC(=O)N(c3ccccc3)c4ccccc4)c5ccccc5
CCOC(=O)c1c(c(sc1NC(=O)CSc2c(c(cc(n2)c3ccccc3)c4ccc(cc4)C)C#N)C)C
CCOc1ccc(cc1OC)[C@@H]2C(=C(C(=O)N2c3nc(c(s3)C(=O)C)C)O)C(=O)c4cc5ccccc5o4
CC1=NC2=C([C@@H](C1C(=O)OC)c3ccc(c(c3)COc4ccccc4Br)OC)C(=O)CC(C2)(C)C
Cc1ccc(cc1)c2cc(nc(c2C#N)SCC(=O)Nc3cccc4c3cccc4)c5ccccc5
CCOc1cc(ccc1O)[C@@H]2C(=C(C(=O)N2c3nc(c(s3)C(=O)C)C)O)C(=O)c4cc5ccccc5o4
CCOC(=O)CCCSc1c(c(cc(n1)c2ccccc2)c3ccc(cc3)OC)C#N
CCOc1cc(ccc1O)[C@H]2C(=C(C(=O)N2c3nc(c(s3)C(=O)C)C)O)C(=O)c4cc5ccccc5o4
Cc1ccc(c(c1)NC(=O)CSc2c(c(cc(n2)c3ccccc3)c4ccc(cc4)OC)C#N)C
Cc1c(sc(n1)N2[C@@H](C(=C(C2=O)O)C(=O)c3cc4ccccc4o3)c5cc(c(c(c5)OC)OC)OC)C(=O)C
CC1=NC2=C([C@H](C1C(=O)OC)c3ccc(c(c3)OC)COc4ccc(cc4)F)C(=O)CC(C2)(C)C
Cc1ccc(c(c1)NC(=O)c2c(c3c(cc(nc3s2)c4ccccc4)c5ccc(cc5)OC)N)C
CCOC(=O)CSc1c(c(cc(n1)c2ccc(cc2)Br)c3ccccc3)C#N
Cc1c(sc(n1)N2[C@H](C(=C(C2=O)O)C(=O)c3cc4ccccc4o3)c5cc(c(c(c5)OC)OC)OC)C(=O)C
CCOc1ccc(cc1)c2cc(c(c(n2)SCC(=O)Nc3ccc(cc3)OCC)C#N)c4ccccc4
COc1ccc(cc1)c2cc(nc3c2c(c(s3)C(=O)Nc4cccc5c4cccc5)N)c6ccccc6
CCCCOc1ccc(cc1)[C@@H]2C(=C(C(=O)N2c3nc(c(s3)C(=O)C)C)O)C(=O)c4cc5ccccc5o4
CCc1ccccc1NC(=O)c2c(c3c(cc(nc3s2)c4ccccc4)c5ccc(cc5)OC)N
COc1ccc(cc1)c2cc(nc(c2C#N)SCc3ccccc3)c4ccc(cc4)OC
Cc1ccc(cc1)CSc2c(c(cc(n2)c3ccc(cc3)OC)c4ccc(cc4)OC)C#N
CC1=NC2=C([C@@H](C1C(=O)OC)c3ccc(c(c3)OC)COc4ccc(cc4)F)C(=O)CC(C2)(C)C
CCCCOc1ccc(cc1)[C@H]2C(=C(C(=O)N2c3nc(c(s3)C(=O)C)C)O)C(=O)c4cc5ccccc5o4
CC(C)(C)c1ccc(cc1)CSc2c(c(cc(n2)c3ccc(cc3)OC)c4ccc(cc4)OC)C#N
CCOC(=O)C(C(=O)OCC)Sc1c(c(cc(n1)c2ccc(cc2)OC)c3ccc(cc3)OC)C#N
COc1ccc(cc1)c2cc(nc(c2C#N)SCC(=O)Oc3cccc(c3)OC)c4ccc(cc4)OC
Cc1c(sc(n1)N2[C@@H](C(=C(C2=O)O)C(=O)c3cc4ccccc4o3)c5ccc(cc5)OCC=C)C(=O)C
COc1ccc(cc1)c2cc(nc(c2C#N)SCC(=O)OC3CCCC3)c4ccccc4
Cc1c(sc(n1)N2[C@H](C(=C(C2=O)O)C(=O)c3cc4ccccc4o3)c5ccc(cc5)OCC=C)C(=O)C
Cc1cc(cc(c1)NC(=O)c2c(c3c(cc(nc3s2)c4ccccc4)c5ccc(cc5)OC)N)C
COc1ccc(cc1)c2cc(nc(c2C#N)SCC(=O)Nc3ccccc3C(F)(F)F)c4ccccc4
CCOC(=O)C1[C@H](C2=C(CC(CC2=O)(C)C)N=C1C)c3ccc(c(c3)OC)COc4ccc(cc4)OC
```

```
COc1ccc(cc1)c2cc(nc(c2C#N)SCC(=O)Nc3ccc(cc3)Oc4ccccc4)c5ccccc5
Cc1c(sc(n1)N2[C@@H](C(=C(C2=O)O)C(=O)c3cc4ccccc4o3)c5ccc(cc5)OCc6ccccc6)C(=O)C
CCOC(=O)C1[C@@H](C2=C(CC(CC2=O)(C)C)N=C1C)c3ccc(c(c3)OC)COc4ccc(cc4)OC
Cc1cc(ccc1NC(=O)CSc2nc3c(c4ccccc4[nH]3)nn2)I
COc1ccc(cc1NC(=O)CN(C2CCCCC2)C(=O)c3c(c4ccccc4s3)Cl)Cl
CCc1ccccc1NC(=O)c2c(c3c(cc(nc3s2)c4ccccc4)c5ccc(cc5)Cl)N
CCOC(=O)C1[C@H](C2=C(CCCC2=O)N=C1C)c3ccc(c(c3)OC)COc4ccc(cc4)F
Cc1c(sc(n1)N2[C@@H](C(=C(C2=O)O)C(=O)c3cc4ccccc4o3)c5cc(c(c(c5)Br)O)OC)C(=O)C
Cc1c(sc(n1)N2[C@H](C(=C(C2=O)O)C(=O)c3cc4ccccc4o3)c5cc(c(c(c5)Br)O)OC)C(=O)C
CCOC(=O)C1[C@@H](C2=C(CCCC2=O)N=C1C)c3ccc(c(c3)OC)COc4ccc(cc4)F
c1cc(c(cc1Cl)Cl)/C=C/2\CCCC/C(=C/c3ccc(cc3Cl)Cl)/C2=O
Cc1ccc(cc1)c2cc(nc(c2C#N)SCC(=O)c3ccccc3)c4ccccc4
Cc1c(sc(n1)N2[C@@H](C(=C(C2=O)O)C(=O)c3cc4ccccc4o3)c5ccc(cc5)C(C)C)C(=O)C
Cc1ccc(cc1)c2cc(nc(c2C#N)SCC(=O)Nc3c(ccc4c3cccc4)C)c5ccccc5
Cc1ccc(cc1)c2cc(nc(c2C#N)SCC(=O)Nc3cc(cc(c3)C)C)c4ccccc4
Cc1cc(cc(c1)NC(=O)CSc2c(c(cc(n2)c3ccccc3)c4ccc(cc4)C(C)(C)C)C#N)C
CC(C)(C)c1ccc(cc1)c2cc(nc(c2C#N)SCC(=O)Nc3cc(ccc3OC)OC)c4ccccc4
Cc1c(sc(n1)N2[C@H](C(=C(C2=O)O)C(=O)c3cc4ccccc4o3)c5ccc(cc5)C(C)C)C(=O)C
CCOC(=O)c1c(c(sc1NC(=O)CSc2c(c(cc(n2)c3ccccc3)c4ccccc4Cl)C#N)C(=O)C)C
CC(C)(C)c1ccc(cc1)c2cc(nc(c2C#N)SCC(=O)c3ccccc3)c4ccccc4
Cc1ccc(c(c1)NC(=O)c2c(c3c(cc(nc3s2)c4ccccc4)c5ccc(c(c5)OC)OC)N)C
Cc1ccc(cc1)CSc2c(c(cc(n2)c3ccccc3)c4ccc(cc4)C(C)(C)C)C#N
Cc1ccc(c(c1)NC(=O)CSc2c(c(cc(n2)c3ccccc3)c4ccc(c(c4)OC)OC)C#N)C
CCOC(=O)C1[C@H](C2=C(CCCC2=O)N=C1C)c3ccc(c(c3)OC)COc4ccc(cc4)OC
COc1ccc(cc1OC)c2cc(nc(c2C#N)SCC(=O)Nc3ccccc3C(F)(F)F)c4ccccc4
c1ccc(cc1)CSc2nnc(s2)N3[C@@H](C(=C(C3=O)O)C(=O)c4cc5ccccc5o4)c6ccccc6
c1ccc(cc1)CSc2nnc(s2)N3[C@H](C(=C(C3=O)O)C(=O)c4cc5ccccc5o4)c6ccccc6
CCOC(=O)C1[C@@H](C2=C(CCCC2=O)N=C1C)c3ccc(c(c3)OC)COc4ccc(cc4)OC
c1ccc(cc1)CSc2nnc(s2)N3[C@@H](C(=C(C3=O)O)C(=O)c4cc5ccccc5o4)c6cccc(c6)Br
c1ccc(cc1)CSc2nnc(s2)N3[C@H](C(=C(C3=O)O)C(=O)c4cc5ccccc5o4)c6cccc(c6)Br
CCCCCCCSc1nc2c(n1CC(=C)C)c(=O)[nH]c(=O)n2C
```

```
CCOC(=O)C1[C@H](C2=C(CC(CC2=O)(C)C)N=C1C)c3ccc(c(c3)OC)COc4ccc(cc4)F
CCCCCCCCSc1nc2c(n1CC(=C)C)c(=O)[nH]c(=O)n2C
c1ccc(cc1)/C=C/C(=O)Nc2cc3c(cc2)-c4c(cc(cc4)NC(=O)/C=C/c5ccccc5)S3(=O)=O
c1ccc(cc1)CSc2nnc(s2)N3[C@@H](C(=C(C3=O)O)C(=O)c4cc5ccccc5o4)c6ccc(cc6)O
CCOC(=O)C1[C@@H](C2=C(CC(CC2=O)(C)C)N=C1C)c3ccc(c(c3)OC)COc4ccc(cc4)F
c1ccc(cc1)CSc2nnc(s2)N3[C@H](C(=C(C3=O)O)C(=O)c4cc5ccccc5o4)c6ccc(cc6)O
CCCCCOc1ccc(cc1OC)[C@@H]2C(=C(C(=O)N2c3nnc(s3)SCc4ccccc4)O)C(=O)c5cc6ccccc6o5
CCOC(=O)C1[C@H](C2=C(CC(CC2=O)(C)C)N=C1C)c3ccc(c(c3)COc4ccccc4Br)OC
Cc1ccc2cc(c(nc2c1)Cl)[C@@H]3CC(=NN3C(=O)CCC(=O)[O-])c4ccc(cc4)OC
CCCCCOc1ccc(cc1OC)[C@H]2C(=C(C(=O)N2c3nnc(s3)SCc4ccccc4)O)C(=O)c5cc6ccccc6o5
Cc1ccc2cc(c(nc2c1)Cl)[C@H]3CC(=NN3C(=O)CCC(=O)[O-])c4ccc(cc4)OC
CCOC(=O)C1[C@@H](C2=C(CC(CC2=O)(C)C)N=C1C)c3ccc(c(c3)COc4ccccc4Br)OC
CCOC(=O)C1[C@H](C2=C(CC(CC2=O)(C)C)N=C1C)c3ccc(o3)COc4cccc(c4)C(F)(F)F
CCOc1cc(ccc1O)[C@@H]2C(=C(C(=O)N2c3nnc(s3)SCc4ccccc4)O)C(=O)c5cc6ccccc6o5
Cc1cccc(c1)N2CCN(CC2)C3=NC(=O)/C(=C\c4ccc(o4)c5ccc(cc5)[N+](=O)[O-])/S3
CCOc1cc(ccc1O)[C@H]2C(=C(C(=O)N2c3nnc(s3)SCc4ccccc4)O)C(=O)c5cc6ccccc6o5
CCOC(=O)C1[C@@H](C2=C(CC(CC2=O)(C)C)N=C1C)c3ccc(o3)COc4cccc(c4)C(F)(F)F
Cc1ccc(cc1)N2CCN(CC2)C3=NC(=O)/C(=C\c4ccc(o4)c5ccc(cc5)[N+](=O)[O-])/S3
c1ccc(cc1)C[NH+]2CCN(CC2)C3=NC(=O)/C(=C\c4ccc(o4)c5ccc(cc5)[N+](=O)[O-])/S3
CCc1ccc(cc1)[C@@H]2C(=C(C(=O)N2c3nnc(s3)SCc4ccccc4)O)C(=O)c5cc6ccccc6o5
Cc1ccc(cc1)N2CCN(CC2)C3=NC(=O)/C(=C\c4ccc(o4)c5cc(ccc5Cl)Cl)/S3
CCOC(=O)C1[C@H](C2=C(CC(CC2=O)(C)C)N=C1C)c3ccc(c(c3)COc4ccc(cc4Br)Cl)OC
c1ccc(c(c1)c2nnc(o2)SCC(=O)Nc3ccc(cc3)[N+](=O)[O-])Br
CCc1ccc(cc1)[C@H]2C(=C(C(=O)N2c3nnc(s3)SCc4ccccc4)O)C(=O)c5cc6ccccc6o5
COc1cc(cc(c1OC)OC)[C@@H]2C(=C(C(=O)N2c3nnc(s3)SCc4ccccc4)O)C(=O)c5cc6ccccc6o5
CCOC(=O)C1[C@@H](C2=C(CC(CC2=O)(C)C)N=C1C)c3ccc(c(c3)COc4ccc(cc4Br)Cl)OC
COc1cc(cc(c1OC)OC)[C@H]2C(=C(C(=O)N2c3nnc(s3)SCc4ccccc4)O)C(=O)c5cc6ccccc6o5
C=CCOc1ccc(cc1)[C@@H]2C(=C(C(=O)N2c3nnc(s3)SCc4ccccc4)O)C(=O)c5cc6ccccc6o5
CCn1c(=O)/c(=C\C=C\2/C(c3cc(ccc3N2C)c4ccccc4)(C)C)/sc1=C5C(=O)c6ccccc6C5=O
CC1=NC2=C([C@H](C1C(=O)OC)c3ccc(c(c3)COc4ccccc4Cl)OC)C(=O)CC(C2)(C)C
C=CCOc1ccc(cc1)[C@H]2C(=C(C(=O)N2c3nnc(s3)SCc4ccccc4)O)C(=O)c5cc6ccccc6o5
```

```
c1ccc(cc1)COc2ccc(cc2)[C@@H]3C(=C(C(=O)N3c4nnc(s4)SCc5ccccc5)O)C(=O)c6cc7ccccc7o6
CC1=C([C@H](n2c(=O)/c(=C\C=C\c3ccccc3)/sc2=N1)c4ccccc4Cl)C(=O)OC
c1ccc(cc1)COc2ccc(cc2)[C@H]3C(=C(C(=O)N3c4nnc(s4)SCc5ccccc5)O)C(=O)c6cc7ccccc7o6
c1ccc(cc1)CNC(=O)/C(=C\c2ccc(o2)c3ccccc3Cl)/NC(=O)c4ccccc4
CC(C)CCOc1ccc(cc1OC)[C@@H]2C(=C(C(=O)N2c3nnc(s3)SCc4ccccc4)O)C(=O)c5cc6ccccc6o5
CC1=NC2=C([C@@H](C1C(=O)OC)c3ccc(c(c3)COc4ccccc4Cl)OC)C(=O)CC(C2)(C)C
CC(C)CCOc1ccc(cc1OC)[C@H]2C(=C(C(=O)N2c3nnc(s3)SCc4ccccc4)O)C(=O)c5cc6ccccc6o5
CC1=C([C@@H](NC(=O)N1)c2ccc(o2)c3cc(ccc3Cl)Cl)C(=O)OCCOC
CN(C)c1ccc(cc1)[C@@H]2C(=C(C(=O)N2c3nnc(s3)SCc4ccccc4)O)C(=O)c5cc6ccccc6o5
CC1=C([C@H](NC(=O)N1)c2ccc(o2)c3cc(ccc3Cl)Cl)C(=O)OCCOC
c1ccc(cc1)C(=O)N/C(=C\c2ccc(o2)c3ccc(cc3Cl)Cl)/C(=O)NCc4ccco4
CN(C)c1ccc(cc1)[C@H]2C(=C(C(=O)N2c3nnc(s3)SCc4ccccc4)O)C(=O)c5cc6ccccc6o5
CCOC(=O)C1[C@H](C2=C(CC(CC2=O)(C)C)N=C1C)c3ccc(o3)COc4ccccc4Br
Cc1ccc(cc1)C(=O)N/C(=C\c2ccc(o2)c3cc(ccc3Cl)Cl)/C(=O)NCCc4ccccc4
Cc1ccc(cc1)C(=O)N/C(=C\c2ccc(o2)c3cc(ccc3Cl)Cl)/C(=O)NC
c1ccc(cc1)CSc2nnc(s2)N3[C@@H](C(=C(C3=O)O)C(=O)c4cc5ccccc5o4)c6ccccc6F
c1ccc(cc1)C(=O)N/C(=C/c2ccc(o2)c3ccccc3[N+](=O)[O-])/C(=O)N4CCCCC4
CC1CCN(CC1)C(=O)/C(=C\c2ccc(o2)c3ccccc3[N+](=O)[O-])/NC(=O)c4ccccc4
CCOC(=O)C1[C@@H](C2=C(CC(CC2=O)(C)C)N=C1C)c3ccc(o3)COc4ccccc4Br
CCOC(=O)C1=C(N=c2n(c(=O)/c(=C\c3ccco3)/s2)[C@@H]1c4ccc(cc4)C(C)(C)C)C
c1ccc(cc1)CSc2nnc(s2)N3[C@H](C(=C(C3=O)O)C(=O)c4cc5ccccc5o4)c6ccccc6F
CCOC(=O)C1=C(N=c2n(c(=O)/c(=C\c3ccco3)/s2)[C@H]1c4ccc(cc4)C(C)(C)C)C
Cc1ccc(cc1)CSc2nnc(s2)N3[C@@H](C(=C(C3=O)O)C(=O)c4cc5ccccc5o4)c6ccccc6
CC1=C([C@H](n2c(=O)/c(=C\c3cccs3)/sc2=N1)c4ccc(cc4)OC(=O)C)C(=O)OC
COc1ccc(cc1)C(=O)N/C(=C\c2ccc(o2)c3ccc(cc3)Br)/C(=O)NC4CCCCC4
Cc1ccc(cc1)CSc2nnc(s2)N3[C@H](C(=C(C3=O)O)C(=O)c4cc5ccccc5o4)c6ccccc6
Cc1ccc(cc1)C(=O)N/C(=C\c2ccc(o2)c3cc(ccc3Cl)Cl)/C(=O)NC4CCCCC4
Cc1ccc(cc1)C(=O)N/C(=C\c2cn(nc2c3ccccc3)c4ccccc4)/C(=O)NC[C@@H]5CCCO5
Cc1ccc(cc1)C(=O)N/C(=C\c2cn(nc2c3ccccc3)c4ccccc4)/C(=O)NC[C@H]5CCCO5
CCCCCOc1ccc(cc1OC)[C@@H]2C(=C(C(=O)N2CCCn3ccnc3)O)C(=O)c4cc5ccccc5o4
CC1=NC(=C([C@@H](C1C(=O)OC(C)C)c2ccc(o2)c3cc(ccc3Cl)Cl)C(=O)OC(C)C)C
```

```
CC1=NC2=C([C@H](C1C(=O)OC)c3ccc(o3)COc4cccc(c4)Br)C(=O)CC(C2)(C)C
CCCCCOc1ccc(cc1OC)[C@H]2C(=C(C(=O)N2CCCn3ccnc3)O)C(=O)c4cc5ccccc5o4
COc1cc(cc(c1)OC)C[NH+]2CCN(CC2)C(=O)c3cc(c(c(c3)OC)OC)OC
C=CCOc1ccc(cc1)[C@@H]2C(=C(C(=O)N2CCCn3ccnc3)O)C(=O)c4cc5ccccc5o4
CC1=NC2=C([C@@H](C1C(=O)OC)c3ccc(o3)COc4cccc(c4)Br)C(=O)CC(C2)(C)C
CCOC(=O)c1c2c(sc1NC(=O)c3cc(ccc3N4CC[NH+](CC4)C)[N+](=O)[O-])CCCC2
C=CCOc1ccc(cc1)[C@H]2C(=C(C(=O)N2CCCn3ccnc3)O)C(=O)c4cc5ccccc5o4
CCOc1ccc(cc1)N2C(=O)/C(=C\c3cc(n(c3C)c4ccccc4)C)/C(=O)N(C2=S)c5ccccc5
CC(C)CCOc1ccc(cc1OC)[C@H]2C(=C(C(=O)N2CCCn3ccnc3)O)C(=O)c4cc5ccccc5o4
COc1cc(ccc1OCc2ccccc2)[C@@H]3C(=C(C(=O)N3CCCn4ccnc4)O)C(=O)c5cc6ccccc6o5
CCOC(=O)C1[C@H](C2=C(CC(CC2=O)(C)C)N=C1C)c3ccc(c(c3)COc4ccc(cc4)C)OC
CCCCCOc1ccc(cc1)[C@@H]2C(=C(C(=O)N2CCCn3ccnc3)O)C(=O)c4cc5ccccc5o4
CCOc1ccc(cc1)NC(=O)CSc2c(c(cc(n2)c3ccc(cc3)C)c4ccccc4)C#N
CCOC(=O)C1[C@@H](C2=C(CC(CC2=O)(C)C)N=C1C)c3ccc(c(c3)COc4ccc(cc4)C)OC
Cc1ccc(cc1)c2cc(c(c(n2)SCC(=O)Nc3cccc4c3cccc4)C#N)c5ccccc5
CCOC(=O)C1[C@H](C2=C(CCCC2=O)N=C1C)c3ccc(c(c3)COc4ccc(cc4Br)Cl)OC
Cc1ccc(cc1)c2cc(c(c(n2)SCC(=O)Nc3ccccc3C(F)(F)F)C#N)c4ccc(c(c4)OC)OC
Cc1ccc(cc1)c2cc(c(c(n2)SCC(=O)Nc3ccccc3C(F)(F)F)C#N)c4ccccc4
CCOC(=O)C1[C@@H](C2=C(CCCC2=O)N=C1C)c3ccc(c(c3)COc4ccc(cc4Br)Cl)OC
CCc1ccccc1NC(=O)CSc2c(c(cc(n2)c3ccc(cc3)C)c4ccccc4)C#N
CCOc1ccc(cc1OC)[C@@H]2C(=C(C(=O)N2CCC[NH+]3CCOCC3)O)C(=O)c4cc5ccccc5o4
c1ccc(c(c1)c2nnc(o2)SCC(=O)Nc3ccccc3[N+](=O)[O-])Br
CCOc1ccc(cc1OC)[C@H]2C(=C(C(=O)N2CCC[NH+]3CCOCC3)O)C(=O)c4cc5ccccc5o4
CCOC(=O)C1[C@H](C2=C(CC(CC2=O)(C)C)N=C1C)c3ccc(o3)COc4cccc(c4)Br
Cc1ccc(cc1)c2cc(c(c(n2)SCC(=O)Nc3ccc(cc3)C(=O)C)C#N)c4ccccc4
Cc1ccc(cc1)c2cc(c(c(n2)SCC(=O)Nc3cc(cc(c3)C)C)C#N)c4ccccc4
c1ccc2c(c1)cc(o2)C(=O)C3=C(C(=O)N([C@@H]3c4cccnc4)CCC[NH+]5CCOCC5)O
Cc1ccc(cc1)c2cc(c3c(c(sc3n2)C(=O)Nc4c(ccc5c4cccc5)C)N)c6ccccc6
Cc1ccc(cc1)c2cc(c3c(c(sc3n2)C(=O)Nc4ccccc4OC)N)c5ccc(c(c5)OC)OC
CCCCOc1ccc(cc1)[C@@H]2C(=C(C(=O)N2CCC[NH+]3CCOCC3)O)C(=O)c4cc5ccccc5o4
CCOC(=O)C1[C@@H](C2=C(CC(CC2=O)(C)C)N=C1C)c3ccc(o3)COc4cccc(c4)Br
```

```
Cc1ccc(cc1)c2cc(c(c(n2)SCC(=O)Nc3c(ccc4c3cccc4)C)C#N)c5ccccc5
CCCCOc1ccc(cc1)[C@H]2C(=C(C(=O)N2CCC[NH+]3CCOCC3)O)C(=O)c4cc5ccccc5o4
Cc1ccc(cc1)c2cc(c3c(c(sc3n2)C(=O)Nc4ccccc4C(F)(F)F)N)c5ccc(cc5)OC
Cc1ccc(cc1)c2cc(c(c(n2)SCc3ccccc3)C#N)c4ccc(cc4)OC
C=CCOc1ccc(cc1)[C@@H]2C(=C(C(=O)N2CCC[NH+]3CCOCC3)O)C(=O)c4cc5ccccc5o4
CCOC(=O)C1[C@H](C2=C(CC(CC2=O)(C)C)N=C1C)c3ccc(c(c3)COc4ccc(c(c4)Cl)F)OC
Cc1ccc(cc1)c2cc(c(c(n2)SCC(=O)Nc3ccccc3C(F)(F)F)C#N)c4ccc(cc4)OC
Cc1ccc(cc1)c2cc(c(c(n2)SCC(=O)Nc3cccc(c3)C)C#N)c4ccc(c(c4)OC)OC
CC(C)(C)c1ccc(cc1)CSc2c(c(cc(n2)c3ccc(cc3)OC)c4ccccc4)C#N
C=CCOc1ccc(cc1)[C@H]2C(=C(C(=O)N2CCC[NH+]3CCOCC3)O)C(=O)c4cc5ccccc5o4
Cc1cc(cc(c1)NC(=O)c2c(c3c(cc(nc3s2)c4ccc(cc4)OC)c5ccc(cc5)OC)N)C
Cc1cc(cc(c1)NC(=O)c2c(c3c(cc(nc3s2)c4ccc(cc4)OC)c5ccccc5)N)C
c1ccc(cc1)COc2ccc(cc2)[C@@H]3C(=C(C(=O)N3CCC[NH+]4CCOCC4)O)C(=O)c5cc6ccccc6o5
CCOC(=O)C1[C@@H](C2=C(CC(CC2=O)(C)C)N=C1C)c3ccc(c(c3)COc4ccc(c(c4)Cl)F)OC
COc1ccc(cc1)c2cc(c(c(n2)SCC(=O)Nc3ccccc3C(F)(F)F)C#N)c4ccccc4
CCc1ccccc1NC(=O)CSc2c(c(cc(n2)c3ccc(cc3)OC)c4ccc(cc4)OC)C#N
COc1ccc(cc1)c2cc(nc3c2c(c(s3)C(=O)Nc4ccccc4C(F)(F)F)N)c5ccc(cc5)OC
CCc1ccccc1NC(=O)c2c(c3c(cc(nc3s2)c4ccc(cc4)OC)c5ccc(cc5)OC)N
CCCCOc1ccc(cc1OC)[C@@H]2C(=C(C(=O)N2CCC[NH+]3CCOCC3)O)C(=O)c4cc5ccccc5o4
Cc1ccc(cc1C)NC(=O)CSc2c(c(cc(n2)c3ccc(cc3)OC)c4ccc(cc4)OC)C#N
CCOC(=O)c1c(c(sc1NC(=O)CSc2c(c(cc(n2)c3ccc(cc3)OC)c4ccc(cc4)OC)C#N)C)C
Cc1ccc(cc1C)NC(=O)c2c(c3c(cc(nc3s2)c4ccc(cc4)OC)c5ccc(cc5)OC)N
CCCCOc1ccc(cc1OC)[C@H]2C(=C(C(=O)N2CCC[NH+]3CCOCC3)O)C(=O)c4cc5ccccc5o4
CC1=NC2=C([C@H](C1C(=O)OC)c3ccc(c(c3)COc4ccc(c(c4)Cl)F)OC)C(=O)CC(C2)(C)C
COc1ccc(cc1)c2cc(nc3c2c(c(s3)C(=O)Nc4ccc(cc4)OC)N)c5ccc(cc5)OC
CCOc1ccc(cc1OC)[C@@H]2C(=C(C(=O)N2CC[NH+]3CCOCC3)O)C(=O)c4cc5ccccc5o4
c1ccc(cc1)c2cc(c3c(c(sc3n2)C(=O)c4ccc(cc4)Cl)N)c5ccc(cc5)Cl
Cc1ccc(cc1)C(=O)NNC(=O)CSc2c(c(cc(n2)c3ccccc3)c4ccc(cc4)Cl)C#N
CCOc1ccc(cc1OC)[C@H]2C(=C(C(=O)N2CC[NH+]3CCOCC3)O)C(=O)c4cc5ccccc5o4
CC1=NC2=C([C@@H](C1C(=O)OC)c3ccc(c(c3)COc4ccc(c(c4)Cl)F)OC)C(=O)CC(C2)(C)C
CC(C)(C)c1ccc(cc1)CSc2c(c(cc(n2)c3ccccc3)c4ccc(cc4)Cl)C#N
```

```
Cc1cc(ccc1NC(=O)[C@@H](C)Sc2c3c4c(sc3ncn2)CCCC4)[N+](=O)[O-]
Cc1cc(ccc1NC(=O)[C@H](C)Sc2c3c4c(sc3ncn2)CCCC4)[N+](=O)[O-]
c1ccc(cc1)N(c2ccccc2)C(=O)CSc3c4c5c(sc4ncn3)CCCC5
CCOC(=O)C1[C@H](C2=C(CC(CC2=O)(C)C)N=C1C)c3ccc(c(c3)COc4ccccc4Cl)OC
c1ccc(cc1)COc2ccc(cc2)[C@@H]3C(=C(C(=O)N3CC[NH+]4CCOCC4)O)C(=O)c5cc6ccccc6o5
Cc1ccc(cc1[N+](=O)[O-])NC(=O)[C@@H](C)Sc2c3c4c(sc3ncn2)CCCC4
Cc1ccc(cc1[N+](=O)[O-])NC(=O)[C@H](C)Sc2c3c4c(sc3ncn2)CCCC4
Cc1cc(ccc1NC(=O)CSc2nnc(o2)c3ccc(cc3)Br)[N+](=O)[O-]
c1ccc(cc1)COc2ccc(cc2)[C@H]3C(=C(C(=O)N3CC[NH+]4CCOCC4)O)C(=O)c5cc6ccccc6o5
CCOC(=O)C1[C@@H](C2=C(CC(CC2=O)(C)C)N=C1C)c3ccc(c(c3)COc4ccccc4Cl)OC
CC(C)CCOc1ccc(cc1OC)[C@@H]2C(=C(C(=O)N2CC[NH+]3CCOCC3)O)C(=O)c4cc5ccccc5o4
COC(=O)c1ccc(cc1)NC(=O)CSc2c(c(cc(n2)c3ccccc3)c4ccccc4Cl)C#N
CC(C)(C)c1ccc(cc1)c2cc(nc(c2C#N)SCC(=O)Nc3cccc4c3cccc4)c5ccccc5
c1ccc(cc1)c2cc(c(c(n2)SCC(=O)Nc3c(c4c(s3)CCCC4)C#N)C#N)c5ccccc5Cl
CCOC(=O)C1[C@H](C2=C(CCCC2=O)N=C1C)c3ccc(c(c3)COc4ccccc4Cl)OC
CC(C)CCOc1ccc(cc1OC)[C@H]2C(=C(C(=O)N2CC[NH+]3CCOCC3)O)C(=O)c4cc5ccccc5o4
c1ccc(cc1)c2cc(c(c(n2)SCC(=O)N3CCCc4c3cccc4)C#N)c5ccccc5Cl
CCOC(=O)c1c2c(sc1NC(=O)CSc3c(c(cc(n3)c4ccccc4)c5ccccc5Cl)C#N)CCCC2
CCOC(=O)C1[C@@H](C2=C(CCCC2=O)N=C1C)c3ccc(c(c3)COc4ccccc4Cl)OC
C[C@H]1CN(C[C@@H](O1)C)C2=NC(=O)/C(=C/c3cn(nc3c4cc5ccccc5o4)c6ccccc6)/S2
Cc1ccc(cc1)OCc2cc(ccc2OC)[C@@H]3C4=C(CC(CC4=O)(C)C)NC(=C3C(=O)OC)C
COc1ccc(cc1Br)[C@@H]2N(CCS2)S(=O)(=O)c3ccc(cc3)c4ccccc4
COc1ccc(cc1Br)[C@H]2N(CCS2)S(=O)(=O)c3ccc(cc3)c4ccccc4
Cc1ccc(cc1[N+](=O)[O-])S(=O)(=O)N2CCS[C@@H]2c3ccc(c(c3)OC)OC
Cc1ccc(cc1)OCc2cc(ccc2OC)[C@H]3C4=C(CC(CC4=O)(C)C)NC(=C3C(=O)OC)C
Cc1ccc(cc1[N+](=O)[O-])S(=O)(=O)N2CCS[C@H]2c3ccc(c(c3)OC)OC
CCn1c2ccccc2c3c1nc(nn3)SCC(=O)Nc4ccc(cc4[N+](=O)[O-])Cl
CCOC(=O)C1[C@H](C2=C(CC(CC2=O)(C)C)N=C1C)c3ccc(o3)COc4ccc(c(c4)C)Cl
CCOC(=O)C1[C@@H](C2=C(CC(CC2=O)(C)C)N=C1C)c3ccc(o3)COc4ccc(c(c4)C)Cl
Cc1cc(=O)oc2c1ccc(c2)OC/C=C/3\[C@@H](c4cc(ccc4O3)[N+](=O)[O-])[NH+]5CCOCC5
Cc1cc(=O)oc2c1ccc(c2)OC/C=C/3\[C@H](c4cc(ccc4O3)[N+](=O)[O-])[NH+]5CCOCC5
```

```
COc1cc(c(c(c1)[N+](=O)[O-])[O-])[C@H]2Nc3ccc(cc3[C@H]([NH+]2CC(=O)OC)c4ccccc4)Br
CCOC(=O)C1[C@H](C2=C(CCCC2=O)N=C1C)c3ccc(c(c3)COc4ccc(c(c4)Cl)F)OC
c1ccc(c(c1)C(=O)c2cc(ccc2NC(=O)C[NH+]3CCCCC3)Br)Cl
C[C@H]1Cc2cc(ccc2O1)C(=O)C3=C(C(=O)N([C@@H]3c4ccc(c(c4)OC)O)C[C@@H]5CCCO5)[O-]
C[C@@H]1Cc2cc(ccc2O1)C(=O)C3=C(C(=O)N([C@H]3c4ccc(c(c4)OC)O)C[C@@H]5CCCO5)[O-]
CCOC(=O)C1[C@@H](C2=C(CCCC2=O)N=C1C)c3ccc(c(c3)COc4ccc(c(c4)Cl)F)OC
C[C@H]1Cc2cc(ccc2O1)C(=O)C3=C(C(=O)N([C@H]3c4ccc(c(c4)OC)O)C[C@@H]5CCCO5)[O-]
Cc1ccc(cc1)c2cc(nc3c2c(c(s3)C(=O)c4ccc(cc4)Cl)N)c5ccccc5
C[C@@H]1Cc2cc(ccc2O1)C(=O)C3=C(C(=O)N([C@@H]3c4cc(c(c(c4)OC)OC)OC)C[C@@H]5CCCO5)[O-]
CCOC(=O)c1c(c(sc1NC(=O)CSc2c(c(cc(n2)c3ccccc3)c4ccc(cc4)Cl)C#N)C(=O)C)C
CCOC(=O)C1[C@H](C2=C(CCCC2=O)N=C1C)c3ccc(c(c3)COc4ccc(cc4)C)OC
CC(C)OC(=O)c1c(c2c(cc(nc2s1)c3ccccc3)c4ccc(cc4)Cl)N
Cc1ccc(cc1)c2nnc(o2)SCC(=O)Nc3ccc(cc3Br)[N+](=O)[O-]
COc1ccc(cc1OC)c2cc(nc(c2C#N)SCC(=O)N(c3ccccc3)c4ccccc4)c5ccccc5
Cc1cc(cc(c1)NC(=O)CSc2c(c(cc(n2)c3ccccc3)c4ccc(c(c4)OC)OC)C#N)C
C[C@@H]1Cc2cc(ccc2O1)C(=O)C3=C(C(=O)N([C@H]3c4cc(c(c(c4)OC)OC)OC)C[C@@H]5CCCO5)[O-]
CCOC(=O)C1[C@@H](C2=C(CCCC2=O)N=C1C)c3ccc(c(c3)COc4ccc(cc4)C)OC
Cc1cc(c2c(c1)N(C(C3=C2C4(C(=C(S3)C(=O)OC)C(=O)OC)SC(=C(S4)C(=O)OC)C(=O)OC)(C)C)C(=O)C)C
COc1cc(cc(c1OC)OC)[C@@H]2C(=C(C(=O)N2C[C@@H]3CCCO3)[O-])C(=O)c4ccc5c(c4)OCCO5
[H]/N=C/1\[C@H](C2=CCCC[C@H]2[C@H](C1(C#N)C#N)c3ccc(o3)COc4ccc(cc4Cl)F)C#N
COc1cc(cc(c1OC)OC)[C@H]2C(=C(C(=O)N2C[C@@H]3CCCO3)[O-])C(=O)c4ccc5c(c4)OCCO5
[H]/N=C/1\[C@H](C2=CCCC[C@@H]2[C@H](C1(C#N)C#N)c3ccc(o3)COc4ccc(cc4Cl)F)C#N
COc1cc(cc(c1OC)OC)[C@@H]2C(=C(C(=O)N2C[C@H]3CCCO3)[O-])C(=O)c4ccc5c(c4)OCCO5
[H]/N=C\1/[C@@H](C2=CCCC[C@@H]2[C@@H](C1(C#N)C#N)c3ccc(o3)COc4ccc(cc4Cl)F)C#N
CC1=NC2=C([C@H](C1C(=O)OC)c3ccc(c(c3)COc4ccc(cc4Br)Cl)OC)C(=O)CC(C2)(C)C
COc1cc(cc(c1OC)OC)[C@H]2C(=C(C(=O)N2C[C@H]3CCCO3)[O-])C(=O)c4ccc5c(c4)OCCO5
CCOc1ccc(cc1)C[NH+](CCc2ccc3c(c2)OCO3)Cc4ccc(c(c4)Br)OC
CC1=NC2=C([C@@H](C1C(=O)OC)c3ccc(c(c3)COc4ccc(cc4Br)Cl)OC)C(=O)CC(C2)(C)C
CCOc1cc(ccc1OCc2ccccc2)c3cc(nc(c3C#N)SCC(=O)Nc4ccc(cc4)C)c5ccccc5
CCOC(=O)C1[C@H](C2=C(CC(CC2=O)(C)C)N=C1C)c3ccc(o3)COc4ccc(cc4F)F
Cc1ccc(cc1)c2cc(c(c(n2)SCC(=O)N(c3ccccc3)c4ccc5ccccc5c4)C#N)c6ccccc6
```

```
CC1=NC2=C([C@H](C1C(=O)OC)c3ccc(c(c3)COc4ccc(cc4Br)Cl)OC)C(=O)CCC2
Cc1ccc(cc1)c2cc(c(c(n2)SCC(=O)N(c3ccccc3)c4ccc5ccccc5c4)C#N)c6ccc(cc6)OC
Cc1ccc(cc1)c2cc(c(c(n2)SCC(=O)Nc3cccc4c3cccc4)C#N)c5ccc(cc5)OC
Cc1ccc(cc1)c2cc(c(c(n2)SCC(=O)Nc3cc(cc(c3)C)C)C#N)c4ccc(c(c4)OC)OC
Cc1ccc(cc1)c2cc(c(c(n2)SCC(=O)Nc3cc(cc(c3)C)C)C#N)c4ccc(cc4)OC
Cc1ccc(cc1)c2cc(c3c(c(sc3n2)C(=O)Nc4cccc5c4cccc5)N)c6ccccc6OC
CCc1ccccc1NC(=O)CSc2c(c(cc(n2)c3ccc(cc3)C)c4ccc(cc4)OC)C#N
CC1=NC2=C([C@@H](C1C(=O)OC)c3ccc(c(c3)COc4ccc(cc4Br)Cl)OC)C(=O)CCC2
Cc1ccc(cc1)c2cc(c3c(c(sc3n2)C(=O)Nc4cccc(c4)C)N)c5ccc(cc5)OC
Cc1ccc(cc1)c2cc(c(c(n2)SCC(=O)Nc3ccccc3C)C#N)c4ccc(c(c4)OC)OC
Cc1ccc(cc1)c2cc(c(c(n2)SCC(=O)Nc3cccc4c3cccc4)C#N)c5ccccc5OC
CCCOc1ccc(cc1)[C@@H]2C(=C(C(=O)N2C[C@@H]3CCCO3)[O-])C(=O)c4ccc5c(c4)C[C@@H](O5)C
CCCOc1ccc(cc1)[C@H]2C(=C(C(=O)N2C[C@@H]3CCCO3)[O-])C(=O)c4ccc5c(c4)C[C@@H](O5)C
CC1=NC2=C([C@H](C1C(=O)OC)c3ccc(c(c3)COc4ccc(c(c4)Cl)F)OC)C(=O)CCC2
CCOc1ccc(cc1)NC(=O)CSc2c(c(cc(n2)c3ccc(cc3)C)c4ccc(cc4)OC)C#N
CCCOc1ccc(cc1)[C@@H]2C(=C(C(=O)N2C[C@H]3CCCO3)[O-])C(=O)c4ccc5c(c4)C[C@@H](O5)C
Cc1ccc(cc1)c2cc(c3c(c(sc3n2)C(=O)Nc4ccccc4OC)N)c5ccc(cc5)OC
CCOC(=O)c1c(c(sc1NC(=O)CSc2[nH]nc(n2)c3ccc(cc3)OC)C(=O)C)C
CCCOc1ccc(cc1)[C@H]2C(=C(C(=O)N2C[C@H]3CCCO3)[O-])C(=O)c4ccc5c(c4)C[C@@H](O5)C
CCc1ccccc1NC(=O)CSc2c(c(cc(n2)c3ccccc3)c4ccc(c(c4)OC)OC)C#N
CCCOc1ccc(cc1)[C@@H]2C(=C(C(=O)N2C[C@@H]3CCCO3)[O-])C(=O)c4ccc5c(c4)OCCO5
COc1ccc(cc1OC)c2cc(nc(c2C#N)SCC(=O)Nc3cccc4c3cccc4)c5ccccc5
CC1=NC2=C([C@@H](C1C(=O)OC)c3ccc(c(c3)COc4ccc(c(c4)Cl)F)OC)C(=O)CCC2
CCCOc1ccc(cc1)[C@H]2C(=C(C(=O)N2C[C@@H]3CCCO3)[O-])C(=O)c4ccc5c(c4)OCCO5
CCCOc1ccc(cc1)[C@@H]2C(=C(C(=O)N2C[C@H]3CCCO3)[O-])C(=O)c4ccc5c(c4)OCCO5
CCCOc1ccc(cc1)[C@H]2C(=C(C(=O)N2C[C@H]3CCCO3)[O-])C(=O)c4ccc5c(c4)OCCO5
C[C@@H]1Cc2cc(ccc2O1)C(=O)C3=C(C(=O)N([C@@H]3c4ccc(cc4)OCc5ccccc5)C[C@@H]6CCCO6)[O-]
COc1ccc(cc1OC)[C@@H]2C3=C(c4ccccc4CC3)N=c5n2c(=O)/c(=C\c6c[nH]c7c6cccc7)/s5
COc1ccc(cc1OC)[C@H]2C3=C(c4ccccc4CC3)N=c5n2c(=O)/c(=C\c6c[nH]c7c6cccc7)/s5
C[C@H]1Cc2cc(ccc2O1)C(=O)C3=C(C(=O)N([C@@H]3c4ccc(cc4)OCc5ccccc5)C[C@@H]6CCCO6)[O-]
CCOC(=O)C1=C(N=c2n(c(=O)/c(=C/c3c[nH]nc3c4ccccc4)/s2)[C@@H]1c5ccccc5)C
```

```
C[C@@H]1Cc2cc(ccc2O1)C(=O)C3=C(C(=O)N([C@H]3c4ccc(cc4)OCc5ccccc5)C[C@@H]6CCCO6)[O-]
CC1(CC2=C([C@@H](Nc3ccc(cc3N2)C(=O)c4ccccc4)c5ccc(cc5)Cl)C(=O)C1)C
C[C@H]1Cc2cc(ccc2O1)C(=O)C3=C(C(=O)N([C@H]3c4ccc(cc4)OCc5ccccc5)C[C@@H]6CCCO6)[O-]
CCOC(=O)C1=C(N=c2n(c(=O)/c(=C/c3c[nH]nc3c4ccccc4)/s2)[C@@H]1c5ccc(cc5)N(C)C)C
CC1(CC2=C([C@H](Nc3ccc(cc3N2)C(=O)c4ccccc4)c5ccc(cc5)Cl)C(=O)C1)C
C[C@@H]1Cc2cc(ccc2O1)C(=O)C3=C(C(=O)N([C@@H]3c4ccc(c(c4)OC)OCCC(C)C)C[C@@H]5CCCO5)[O-]
CCOC(=O)C1=C(N=c2n(c(=O)/c(=C/c3c[nH]nc3c4ccccc4)/s2)[C@H]1c5ccc(cc5)N(C)C)C
CCOC(=O)C1=C(N=c2n(c(=O)/c(=C\c3c[nH]nc3c4ccccc4)/s2)[C@@H]1c5ccc(cc5)OC)C
C[C@@H]1Cc2cc(ccc2O1)C(=O)C3=C(C(=O)N([C@H]3c4ccc(c(c4)OC)OCCC(C)C)C[C@@H]5CCCO5)[O-]
CCOC(=O)C1=C(N=c2n(c(=O)/c(=C\c3c[nH]nc3c4ccccc4)/s2)[C@H]1c5ccc(cc5)OC)C
C[C@H]1Cc2cc(ccc2O1)C(=O)C3=C(C(=O)N([C@H]3c4ccc(c(c4)OC)OCCC(C)C)C[C@@H]5CCCO5)[O-]
CC1=C([C@@H](n2c(=O)/c(=C/c3c[nH]nc3c4ccccc4)/sc2=N1)c5ccccc5)C(=O)OC
CC(C)CCOc1ccc(cc1OC)[C@@H]2C(=C(C(=O)N2C[C@@H]3CCCO3)O)C(=O)c4ccc5c(c4)OCCO5
COc1cc(nc(n1)OC)[N-]S(=O)(=O)c2ccc(cc2)N/C=C\C(=O)c3ccc(cc3)F
CC1=C([C@H](n2c(=O)/c(=C/c3c[nH]nc3c4ccccc4)/sc2=N1)c5ccccc5)C(=O)OC
Cc1cc(nc(n1)[N-]S(=O)(=O)c2ccc(cc2)N/C=C\C(=O)c3ccc4ccccc4c3)C
CC(C)CCOc1ccc(cc1OC)[C@H]2C(=C(C(=O)N2C[C@@H]3CCCO3)O)C(=O)c4ccc5c(c4)OCCO5
CCOC(=O)C1[C@H](C2=C(CC(CC2=O)(C)C)N=C1C)c3ccc(o3)COc4ccc(cc4Cl)F
CC(C)CCOc1ccc(cc1OC)[C@@H]2C(=C(C(=O)N2C[C@H]3CCCO3)O)C(=O)c4ccc5c(c4)OCCO5
CCc1nnc(s1)[N-]S(=O)(=O)c2ccc(cc2)N/C=C\C(=O)c3ccc4c(c3)OCO4
CC(C)CCOc1ccc(cc1OC)[C@H]2C(=C(C(=O)N2C[C@H]3CCCO3)O)C(=O)c4ccc5c(c4)OCCO5
CCCCOc1ccc(cc1OC)[C@@H]2C(=C(C(=O)N2C[C@@H]3CCCO3)[O-])C(=O)c4ccc5c(c4)C[C@@H](O5)C
CCOC(=O)C1[C@@H](C2=C(CC(CC2=O)(C)C)N=C1C)c3ccc(o3)COc4ccc(cc4Cl)F
CCCCOc1ccc(cc1OC)[C@H]2C(=C(C(=O)N2C[C@@H]3CCCO3)[O-])C(=O)c4ccc5c(c4)C[C@@H](O5)C
CCCCOc1ccc(cc1OC)[C@@H]2C(=C(C(=O)N2C[C@H]3CCCO3)O)C(=O)c4ccc5c(c4)C[C@@H](O5)C
CCOC(=O)C1[C@H](C2=C(CC(CC2=O)(C)C)N=C1C)c3ccc(c(c3)COc4ccc(cc4Cl)Br)OC
CCCCOc1ccc(cc1OC)[C@H]2C(=C(C(=O)N2C[C@H]3CCCO3)O)C(=O)c4ccc5c(c4)C[C@@H](O5)C
C[C@@H]1Cc2cc(ccc2O1)C(=O)C3=C(C(=O)N([C@@H]3c4ccc(c(c4)OC)OCc5ccccc5)C[C@@H]6CCCO6)O
CCOC(=O)C1[C@@H](C2=C(CC(CC2=O)(C)C)N=C1C)c3ccc(c(c3)COc4ccc(cc4Cl)Br)OC
C[C@H]1Cc2cc(ccc2O1)C(=O)C3=C(C(=O)N([C@@H]3c4ccc(c(c4)OC)OCc5ccccc5)C[C@@H]6CCCO6)O
CC1=NC2=C([C@H](C1C(=O)OC)c3ccc(c(c3)COc4cccc(c4)C(F)(F)F)OC)C(=O)CCC2
```

```
C[C@H]1Cc2cc(ccc2O1)C(=O)C3=C(C(=O)N([C@H]3c4ccc(c(c4)OC)OCc5ccccc5)C[C@@H]6CCCO6)O
CCCCCOc1ccc(cc1)[C@@H]2C(=C(C(=O)N2C[C@@H]3CCCO3)[O-])C(=O)c4ccc5c(c4)C[C@@H](O5)C
CCCCCOc1ccc(cc1)[C@H]2C(=C(C(=O)N2C[C@@H]3CCCO3)[O-])C(=O)c4ccc5c(c4)C[C@@H](O5)C
CC1=NC2=C([C@@H](C1C(=O)OC)c3ccc(c(c3)COc4cccc(c4)C(F)(F)F)OC)C(=O)CCC2
CCCCCOc1ccc(cc1)[C@@H]2C(=C(C(=O)N2C[C@H]3CCCO3)[O-])C(=O)c4ccc5c(c4)C[C@@H](O5)C
Cc1ccc(cc1)C[n+]2c3ccccc3n(c2N)CC(=O)c4ccc(c(c4)Cl)Cl
CCOC(=O)c1c(c(sc1NC(=O)CSc2[nH]nc(n2)CNc3ccccc3)C)C
CCOc1ccc(cc1)NC(=O)CSc2c(c(cc(n2)c3ccc(cc3)OC)c4ccc(cc4)OC)C#N
CCCCCOc1ccc(cc1)[C@H]2C(=C(C(=O)N2C[C@H]3CCCO3)[O-])C(=O)c4ccc5c(c4)C[C@@H](O5)C
[H]/N=C/1\[C@@H](C2=CCCC[C@H]2[C@H](C1(C#N)C#N)c3ccc(c(c3)COc4cccc(c4)C(F)(F)F)OC)C#N
Cc1ccc(cc1)c2cc(nc(c2C#N)SCC(=O)Nc3ccccc3)c4ccc(cc4)F
CCCCCOc1ccc(cc1)[C@@H]2C(=C(C(=O)N2C[C@@H]3CCCO3)[O-])C(=O)c4ccc5c(c4)OCCO5
CCc1ccccc1NC(=O)CSc2c(c(cc(n2)c3ccc(cc3)F)c4ccc(cc4)C)C#N
Cc1ccc(cc1)c2cc(nc(c2C#N)SCC(=O)Nc3cccc(c3)C)c4ccc(cc4)F
[H]/N=C/1\[C@H](C2=CCCC[C@@H]2[C@H](C1(C#N)C#N)c3ccc(c(c3)COc4cccc(c4)C(F)(F)F)OC)C#N
CCCCCOc1ccc(cc1)[C@H]2C(=C(C(=O)N2C[C@@H]3CCCO3)[O-])C(=O)c4ccc5c(c4)OCCO5
[H]/N=C/1\[C@@H](C2=CCCC[C@@H]2[C@@H](C1(C#N)C#N)c3ccc(c(c3)COc4cccc(c4)C(F)(F)F)OC)C#N
CCCCCOc1ccc(cc1)[C@@H]2C(=C(C(=O)N2C[C@H]3CCCO3)[O-])C(=O)c4ccc5c(c4)OCCO5
CCCCCOc1ccc(cc1)[C@H]2C(=C(C(=O)N2C[C@H]3CCCO3)[O-])C(=O)c4ccc5c(c4)OCCO5
CCOC(=O)C1[C@H](C2=C(CC(CC2=O)(C)C)N=C1C)c3ccc(c(c3)COc4cccc(c4)C(F)(F)F)OC
Cc1ccc2c(c1)c(cc(n2)c3ccccc3)C(=O)Nc4c(c5c(s4)C[C@@H](CC5)C(C)(C)C)C#N
Cc1ccc2c(c1)c(cc(n2)c3ccccc3)C(=O)Nc4c(c5c(s4)C[C@H](CC5)C(C)(C)C)C#N
Cc1cc(c(cc1C)Br)c2ccc(o2)C=C3C(=O)N(C(=O)N(C3=O)C)C
CCOC(=O)C1[C@@H](C2=C(CC(CC2=O)(C)C)N=C1C)c3ccc(c(c3)COc4cccc(c4)C(F)(F)F)OC
CCCCCOc1ccc(cc1OC)[C@@H]2C(=C(C(=O)N2Cc3ccco3)O)C(=O)c4ccc5c(c4)OCCO5
CC1=NC2=C([C@H](C1C(=O)OC)c3ccc(c(c3)COc4cccc(c4)C(F)(F)F)OC)C(=O)CC(C2)(C)C
CCCCCOc1ccc(cc1OC)[C@H]2C(=C(C(=O)N2Cc3ccco3)O)C(=O)c4ccc5c(c4)OCCO5
CCCCCCn1cc(c(=O)c2c1cc(c(c2)F)N3CCN(CC3)C(=O)OCC)C(=O)Nc4nc(cs4)C56CC7CC(C5)CC(C7)C6
CCn1cc(c(=O)c2c1cc(c(c2)F)N3CCN(CC3)C(=O)c4ccco4)C(=O)Nc5nc(cs5)C67CC8CC(C6)CC(C8)C7
CC1=NC2=C([C@@H](C1C(=O)OC)c3ccc(c(c3)COc4cccc(c4)C(F)(F)F)OC)C(=O)CC(C2)(C)C
CC1=NC2=C([C@H](C1C(=O)OC)c3ccc(c(c3)COc4cccc(c4)Br)OC)C(=O)CC(C2)(C)C
```

```
CC\1=NN(C(=O)/C1=C\c2cc(c(c(c2)OC)OC)OC)c3nc(cs3)c4ccc(cc4)Br
CC1=NC2=C([C@@H](C1C(=O)OC)c3ccc(c(c3)COc4cccc(c4)Br)OC)C(=O)CC(C2)(C)C
C[C@@]1(N2[C@@H](CC(=N2)c3ccc4ccccc4c3)c5cc(ccc5O1)Br)c6ccc(cc6)Br
C[C@]1(N2[C@@H](CC(=N2)c3ccc4ccccc4c3)c5cc(ccc5O1)Br)c6ccc(cc6)Br
c1ccc2cc(ccc2c1)C3=NN4[C@@H](C3)c5cc(cc(c5O[C@@H]4c6ccncc6)Cl)Cl
c1ccc2cc(ccc2c1)C3=NN4[C@H](C3)c5cc(cc(c5O[C@@H]4c6ccncc6)Cl)Cl
CCOC(=O)C1[C@H](C2=C(CCCC2=O)N=C1C)c3ccc(c(c3)COc4ccc(cc4Cl)Br)OC
c1ccc2cc(ccc2c1)C3=NN4[C@@H](C3)c5cc(cc(c5O[C@@H]4c6cccs6)Cl)Cl
c1ccc2cc(ccc2c1)C3=NN4[C@H](C3)c5cc(cc(c5O[C@@H]4c6cccs6)Cl)Cl
c1cc(ccc1C2=NN3[C@@H](C2)c4cc(ccc4O[C@@H]3c5ccc(cc5Cl)Cl)Cl)F
c1cc(ccc1C2=NN3[C@H](C2)c4cc(ccc4O[C@@H]3c5ccc(cc5Cl)Cl)Cl)F
CCOC(=O)C1[C@@H](C2=C(CCCC2=O)N=C1C)c3ccc(c(c3)COc4ccc(cc4Cl)Br)OC
c1cc(ccc1[C@@H]2N3[C@@H](CC(=N3)c4ccc(cc4)F)c5cc(ccc5O2)Br)F
c1cc(ccc1[C@H]2N3[C@@H](CC(=N3)c4ccc(cc4)F)c5cc(ccc5O2)Br)F
CCOC(=O)C1[C@H](C2=C(CC(CC2=O)(C)C)N=C1C)c3ccc(c(c3)COc4cccc(c4)Br)OC
COc1ccc(cc1OC)[C@@H]2N3[C@@H](CC(=N3)c4ccc(cc4)F)c5cc(ccc5O2)Br
COc1ccc(cc1OC)[C@H]2N3[C@@H](CC(=N3)c4ccc(cc4)F)c5cc(ccc5O2)Br
CCCCOc1ccc(cc1)[C@@H]2C(=C(C(=O)N2Cc3ccco3)[O-])C(=O)c4ccc5c(c4)OCCO5
CCCCOc1ccc(cc1)[C@H]2C(=C(C(=O)N2Cc3ccco3)[O-])C(=O)c4ccc5c(c4)OCCO5
CCOC(=O)C1[C@@H](C2=C(CC(CC2=O)(C)C)N=C1C)c3ccc(c(c3)COc4cccc(c4)Br)OC
c1ccc(cc1)COc2ccc(cc2)[C@H]3C(=C(C(=O)N3Cc4ccco4)[O-])C(=O)c5ccc6c(c5)OCCO6
CC1=NC2=C([C@H](C1C(=O)OC)c3ccc(c(c3)COc4ccc(cc4Cl)Br)OC)C(=O)CCC2
c1ccc(cc1)CC2CC[NH+](CC2)Cc3ccc(cc3)C(=O)Nc4ccc5ccccc5c4
COc1cc(ccc1N/C=C\C(=O)c2ccc3c(c2)OCO3)Cc4ccc(c(c4)OC)N/C=C\C(=O)c5ccc6c(c5)OCO6
CC1=NC2=C([C@@H](C1C(=O)OC)c3ccc(c(c3)COc4ccc(cc4Cl)Br)OC)C(=O)CCC2
CC(C)CCOc1ccc(cc1OC)[C@@H]2C(=C(C(=O)N2Cc3ccco3)O)C(=O)c4ccc5c(c4)OCCO5
CC1=NC2=C([C@H](C1C(=O)OC)c3ccc(c(c3)COc4cccc(c4)Br)OC)C(=O)CCC2
CC1=NC2=C([C@@H](C1C(=O)OC)c3ccc(c(c3)COc4cccc(c4)Br)OC)C(=O)CCC2
Cc1c(c(c2cc(ccc2n1)Cl)c3ccccc3)C4=NN([C@@H](C4)c5ccc(cc5Cl)Cl)C(=O)CCC(=O)[O-]
Cc1c(c(c2cc(ccc2n1)Cl)c3ccccc3)C4=NN([C@H](C4)c5ccc(cc5Cl)Cl)C(=O)CCC(=O)[O-]
c1ccc(cc1)c2c3cc(ccc3nc(n2)Nc4cccc(c4)C(=O)Nc5ccc(cc5)Cl)Cl
```

```
Cc1ccc(cc1)NC(=O)c2cccc(c2)Nc3nc4ccc(cc4c(n3)c5ccccc5)Br
CCOC(=O)C1[C@@H](C2=C(CCCC2=O)N=C1C)c3ccc(c(c3)COc4cccc(c4)Br)OC
c1cc(ccc1[C@@H]2CC(=NN2CN3c4ccc(cc4C(=O)C3=O)Br)c5ccc(cc5)Br)F
c1cc(ccc1[C@H]2CC(=NN2CN3c4ccc(cc4C(=O)C3=O)Br)c5ccc(cc5)Br)F
c1ccc2c(c1)C(=O)C(=O)N2CN3[C@@H](CC(=N3)c4ccc(cc4)Br)c5ccc(cc5)F
CCOC(=O)C1[C@H](C2=C(CCCC2=O)N=C1C)c3ccc(o3)COc4ccc(cc4)OC
c1ccc2c(c1)C(=O)C(=O)N2CN3[C@H](CC(=N3)c4ccc(cc4)Br)c5ccc(cc5)F
COc1ccccc1[C@@H]2CC(=NN2CN3c4ccccc4C(=O)C3=O)c5ccc(cc5)Br
COc1ccccc1[C@H]2CC(=NN2CN3c4ccccc4C(=O)C3=O)c5ccc(cc5)Br
CCCCCOc1ccc(cc1)[C@@H]2C(=C(C(=O)N2Cc3ccco3)[O-])C(=O)c4ccc5c(c4)OCCO5
COc1ccc(cc1OC)[C@@H]2CC(=NN2C(=O)CCC(=O)[O-])c3ccc(cc3)Br
CCOC(=O)C1[C@@H](C2=C(CCCC2=O)N=C1C)c3ccc(o3)COc4ccc(cc4)OC
COc1ccc(cc1OC)[C@H]2CC(=NN2C(=O)CCC(=O)[O-])c3ccc(cc3)Br
CCCCCOc1ccc(cc1)[C@H]2C(=C(C(=O)N2Cc3ccco3)[O-])C(=O)c4ccc5c(c4)OCCO5
Cc1ccc(cc1S(=O)(=O)N2CCCN(CC2)S(=O)(=O)c3cc(ccc3C)[N+](=O)[O-])[N+](=O)[O-]
CC1=NC2=C([C@H](C1C(=O)OC)c3ccc(c(c3)COc4ccc(cc4Cl)Br)OC)C(=O)CC(C2)(C)C
CC1=NC2=C([C@@H](C1C(=O)OC)c3ccc(c(c3)COc4ccc(cc4Cl)Br)OC)C(=O)CC(C2)(C)C
Cc1c(sc[n+]1CC(=O)c2ccccc2)CCOC(=O)CC34CC5CC(C3)CC(C5)C4
COc1ccc(c(c1)OC)[C@@H]2C(=C(C(=O)N2C[C@@H]3CCCO3)[O-])C(=O)c4ccc5c(c4)OCCO5
Cc1ccc(cc1[N+](=O)[O-])S(=O)(=O)N(CC(=O)NCc2ccccc2OC)c3ccc(cc3)OC
COc1ccc(c(c1)OC)[C@H]2C(=C(C(=O)N2C[C@@H]3CCCO3)[O-])C(=O)c4ccc5c(c4)OCCO5
CCOC(=O)C1[C@H](C2=C(CCCC2=O)N=C1C)c3ccc(c(c3)COc4cccc(c4)C(F)(F)F)OC
c1ccc2c(c1)C(=O)c3ccc(cc3C2=O)NC(=S)NC(=O)c4ccc(cc4)Cl
COc1ccc(c(c1)OC)[C@@H]2C(=C(C(=O)N2C[C@H]3CCCO3)[O-])C(=O)c4ccc5c(c4)OCCO5
CCCn1c2ccccc2c3c1nc(nn3)SCC(=O)Nc4ccc(cc4)[N+](=O)[O-]
COc1ccc(c(c1)OC)[C@H]2C(=C(C(=O)N2C[C@H]3CCCO3)[O-])C(=O)c4ccc5c(c4)OCCO5
CCOC(=O)C1[C@@H](C2=C(CCCC2=O)N=C1C)c3ccc(c(c3)COc4cccc(c4)C(F)(F)F)OC
C[C@@H]1Cc2cc(ccc2O1)C(=O)C3=C(C(=O)N([C@@H]3c4cccs4)C[C@@H]5CCCO5)[O-]
c1cc2c(ccc(c2nc1)[N+](=O)[O-])N3CCN(CC3)S(=O)(=O)c4ccc(cc4)Cl
c1cc(ccc1C(=O)N2CCN(CC2)c3ccc(c(c3)NC4CC4)[N+](=O)[O-])Br
C[C@@H]1Cc2cc(ccc2O1)C(=O)C3=C(C(=O)N([C@H]3c4cccs4)C[C@@H]5CCCO5)[O-]
```

```
C[C@H]1Cc2cc(ccc2O1)C(=O)C3=C(C(=O)N([C@H]3c4cccs4)C[C@@H]5CCCO5)[O-]
CC1=NC2=C([C@H](C1C(=O)OC)c3ccc(c(c3)COc4cc(ccc4[N+](=O)[O-])F)OC)C(=O)CC(C2)(C)C
Cc1c(cccc1NC(=S)NC(=O)c2ccc(cc2)[N+](=O)[O-])c3nc4c(o3)cccn4
CC1=NC2=C([C@@H](C1C(=O)OC)c3ccc(c(c3)COc4cc(ccc4[N+](=O)[O-])F)OC)C(=O)CC(C2)(C)C
CCCCCOc1ccc(cc1OC)[C@@H]2C(=C(C(=O)N2C[C@@H]3CCCO3)O)C(=O)c4ccc5c(c4)OCCO5
CCCCCOc1ccc(cc1OC)[C@H]2C(=C(C(=O)N2C[C@@H]3CCCO3)O)C(=O)c4ccc5c(c4)OCCO5
CCOC(=O)C1[C@H](C2=C(CC(CC2=O)(C)C)N=C1C)c3ccc(c(c3)COc4cc(ccc4[N+](=O)[O-])F)OC
CCCCCOc1ccc(cc1OC)[C@@H]2C(=C(C(=O)N2C[C@H]3CCCO3)O)C(=O)c4ccc5c(c4)OCCO5
CCCCCOc1ccc(cc1OC)[C@H]2C(=C(C(=O)N2C[C@H]3CCCO3)O)C(=O)c4ccc5c(c4)OCCO5
CCOC(=O)C1[C@@H](C2=C(CC(CC2=O)(C)C)N=C1C)c3ccc(c(c3)COc4cc(ccc4[N+](=O)[O-])F)OC
C[C@@H](C(=O)c1ccccc1)OC(=O)c2ccc3c(c2)C(=O)N(C3=O)c4ccc(cc4)[N+](=O)[O-]
C[C@H](C(=O)c1ccccc1)OC(=O)c2ccc3c(c2)C(=O)N(C3=O)c4ccc(cc4)[N+](=O)[O-]
Cc1ccccc1NC(=O)CN(c2ccc(cc2)OC)S(=O)(=O)c3ccc(c(c3)[N+](=O)[O-])C
Cc1ccc(cc1[N+](=O)[O-])S(=O)(=O)N(CC(=O)N2CC[NH+](CC2)C)c3ccc(cc3)OC
c1cc(ccc1C(=O)NC(=S)Nc2ccc(cc2)[N+](=O)[O-])I
CCCCCCCCCCCC(=O)N/N=C/c1c(c[nH]n1)c2ccc(cc2)F
Cc1ccc2c(c1)sc(n2)N3[C@@H](C(=C(C3=O)O)C(=O)c4cc5ccccc5o4)c6ccc(c(c6)Cl)Cl
CCCCCCCCC(=O)N/N=C/c1c[nH]nc1c2ccc(cc2)Cl
Cc1ccc2c(c1)sc(n2)N3[C@H](C(=C(C3=O)O)C(=O)c4cc5ccccc5o4)c6ccc(c(c6)Cl)Cl
Cc1c(cc(s1)[C@@H]2C3=C(CC(CC3=O)(C)C)NC(=C2C(=O)OC)C)COc4ccc(cc4Cl)F
Cc1ccc2c(c1)sc(n2)N3[C@@H](C(=C(C3=O)O)C(=O)c4cc5ccccc5o4)c6ccc(cc6)Br
Cc1ccc2c(c1)sc(n2)N3[C@H](C(=C(C3=O)O)C(=O)c4cc5ccccc5o4)c6ccc(cc6)Br
Cc1c(cc(s1)[C@H]2C3=C(CC(CC3=O)(C)C)NC(=C2C(=O)OC)C)COc4ccc(cc4Cl)F
CCOC(=O)C1[C@H](C2=C(CC(CC2=O)(C)C)N=C1C)c3cc(c(cc3C)C)COc4ccc(cc4C)Cl
CCCCCOc1ccc(cc1OC)[C@@H]2C(=C(C(=O)N2c3nc4ccc(cc4s3)C)O)C(=O)c5cc6ccccc6o5
CCCCCOc1ccc(cc1OC)[C@H]2C(=C(C(=O)N2c3nc4ccc(cc4s3)C)O)C(=O)c5cc6ccccc6o5
CCOC(=O)C1[C@@H](C2=C(CC(CC2=O)(C)C)N=C1C)c3cc(c(cc3C)C)COc4ccc(cc4C)Cl
Cc1ccc2c(c1)sc(n2)N3[C@@H](C(=C(C3=O)O)C(=O)c4cc5ccccc5o4)c6cccc(c6)Oc7ccccc7
Cc1ccc2c(c1)sc(n2)N3[C@H](C(=C(C3=O)O)C(=O)c4cc5ccccc5o4)c6cccc(c6)Oc7ccccc7
Cc1cc(ccc1OCc2cc(sc2C)[C@@H]3C4=C(CC(CC4=O)(C)C)NC(=C3C(=O)OC)C)Cl
Cc1cc(ccc1OCc2cc(sc2C)[C@H]3C4=C(CC(CC4=O)(C)C)NC(=C3C(=O)OC)C)Cl
```

```
CCCOc1ccc(cc1)[C@@H]2C(=C(C(=O)N2c3nc4ccc(cc4s3)C)O)C(=O)c5cc6ccccc6o5
CCCOc1ccc(cc1)[C@H]2C(=C(C(=O)N2c3nc4ccc(cc4s3)C)O)C(=O)c5cc6ccccc6o5
CCOC(=O)C1[C@H](C2=C(CCCC2=O)N=C1C)c3cc(c(cc3C)C)COc4ccc(cc4Cl)F
Cc1ccc2c(c1)sc(n2)N3[C@@H](C(=C(C3=O)O)C(=O)c4cc5ccccc5o4)c6ccc(c(c6)OC)OCCC(C)C
Cc1ccc2c(c1)sc(n2)N3[C@H](C(=C(C3=O)O)C(=O)c4cc5ccccc5o4)c6ccc(c(c6)OC)OCCC(C)C
CCOC(=O)C1[C@@H](C2=C(CCCC2=O)N=C1C)c3cc(c(cc3C)C)COc4ccc(cc4Cl)F
Cc1ccc2c(c1)sc(n2)N3[C@@H](C(=C(C3=O)O)C(=O)c4cc5ccccc5o4)c6ccc(c(c6)OC)OCc7ccccc7
CCOC(=O)C1[C@H](C2=C(CCCC2=O)N=C1C)c3cc(c(cc3C)C)COc4ccc(cc4C)Cl
Cc1ccc2c(c1)sc(n2)N3[C@H](C(=C(C3=O)O)C(=O)c4cc5ccccc5o4)c6ccc(c(c6)OC)OCc7ccccc7
CCOC(=O)C1[C@@H](C2=C(CCCC2=O)N=C1C)c3cc(c(cc3C)C)COc4ccc(cc4C)Cl
CCOC(=O)C1[C@H](C2=C(CCCC2=O)N=C1C)c3cc(c(s3)C)COc4ccc(cc4C)Cl
CCOC(=O)C1[C@@H](C2=C(CCCC2=O)N=C1C)c3cc(c(s3)C)COc4ccc(cc4C)Cl
Cc1cc(c2c(c1)sc(n2)N3[C@@H](C(=C(C3=O)O)C(=O)c4cc5ccccc5o4)c6ccc(cc6)Cl)C
Cc1cc(c2c(c1)sc(n2)N3[C@H](C(=C(C3=O)O)C(=O)c4cc5ccccc5o4)c6ccc(cc6)Cl)C
Cc1c(cc(s1)[C@@H]2C3=C(CC(CC3=O)(C)C)NC(=C2C(=O)OC)C)COc4ccccc4Br
Cc1ccc(cc1)[C@@H]2C(=C(C(=O)N2c3nc4c(cc(cc4s3)C)C)O)C(=O)c5cc6ccccc6o5
Cc1ccc(cc1)[C@H]2C(=C(C(=O)N2c3nc4c(cc(cc4s3)C)C)O)C(=O)c5cc6ccccc6o5
Cc1c(cc(s1)[C@H]2C3=C(CC(CC3=O)(C)C)NC(=C2C(=O)OC)C)COc4ccccc4Br
Cc1cc(c2c(c1)sc(n2)N3[C@@H](C(=C(C3=O)O)C(=O)c4cc5ccccc5o4)c6cccc(c6)Br)C
Cc1cc(c2c(c1)sc(n2)N3[C@H](C(=C(C3=O)O)C(=O)c4cc5ccccc5o4)c6cccc(c6)Br)C
[H]/N=C/1\[C@@H](C2=CCCC[C@H]2[C@H](C1(C#N)C#N)c3cc(c(cc3C)C)COc4ccc(cc4C)Cl)C#N
[H]/N=C/1\[C@@H](C2=CCCC[C@H]2[C@@H](C1(C#N)C#N)c3cc(c(cc3C)C)COc4ccc(cc4C)Cl)C#N
[H]/N=C/1\[C@H](C2=CCCC[C@@H]2[C@H](C1(C#N)C#N)c3cc(c(cc3C)C)COc4ccc(cc4C)Cl)C#N
Cc1cc(c2c(c1)sc(n2)N3[C@@H](C(=C(C3=O)O)C(=O)c4cc5ccccc5o4)c6ccc(c(c6)Cl)Cl)C
[H]/N=C/1\[C@H](C2=CCCC[C@@H]2[C@@H](C1(C#N)C#N)c3cc(c(cc3C)C)COc4ccc(cc4C)Cl)C#N
Cc1cc(c2c(c1)sc(n2)N3[C@H](C(=C(C3=O)O)C(=O)c4cc5ccccc5o4)c6ccc(c(c6)Cl)Cl)C
Cc1cc(ccc1OCc2cc(sc2C)[C@@H]3C4=C(CCCC4=O)NC(=C3C(=O)OC)C)Cl
Cc1cc(ccc1OCc2cc(sc2C)[C@H]3C4=C(CCCC4=O)NC(=C3C(=O)OC)C)Cl
Cc1cc(c(cc1COc2ccc(cc2C)Cl)[C@@H]3C4=C(CCCC4=O)NC(=C3C(=O)OC)C)C
CCOc1ccc(cc1OC)[C@@H]2C(=C(C(=O)N2c3nc4c(cc(cc4s3)C)C)O)C(=O)c5cc6ccccc6o5
CCOc1ccc(cc1OC)[C@H]2C(=C(C(=O)N2c3nc4c(cc(cc4s3)C)C)O)C(=O)c5cc6ccccc6o5
```

Cc1cc(c(cc1COc2ccc(cc2C)Cl)[C@H]3C4=C(CCCC4=O)NC(=C3C(=O)OC)C)C
Cc1cc(c2c(c1)sc(n2)N3[C@@H](C(=C(C3=O)O)C(=O)c4cc5ccccc5o4)c6cccc(c6)Oc7ccccc7)C
Cc1cc(c2c(c1)sc(n2)N3[C@H](C(=C(C3=O)O)C(=O)c4cc5ccccc5o4)c6cccc(c6)Oc7ccccc7)C
Cc1cc(c(cc1COc2ccc(cc2Cl)F)[C@@H]3C4=C(CC(CC4=O)(C)C)NC(=C3C(=O)OC)C)C
CCc1ccc(cc1)[C@@H]2C(=C(C(=O)N2c3nc4c(cc(cc4s3)C)C)O)C(=O)c5cc6ccccc6o5
CCc1ccc(cc1)[C@H]2C(=C(C(=O)N2c3nc4c(cc(cc4s3)C)C)O)C(=O)c5cc6ccccc6o5
Cc1cc(c(cc1COc2ccc(cc2Cl)F)[C@H]3C4=C(CC(CC4=O)(C)C)NC(=C3C(=O)OC)C)C
Cc1c(cc(s1)[C@@H]2C3=C(CCCC3=O)NC(=C2C(=O)OC)C)COc4ccc(cc4Cl)F
CCCCOc1ccc(cc1)[C@@H]2C(=C(C(=O)N2c3nc4c(cc(cc4s3)C)C)O)C(=O)c5cc6ccccc6o5
CCCCOc1ccc(cc1)[C@H]2C(=C(C(=O)N2c3nc4c(cc(cc4s3)C)C)O)C(=O)c5cc6ccccc6o5
Cc1c(cc(s1)[C@H]2C3=C(CCCC3=O)NC(=C2C(=O)OC)C)COc4ccc(cc4Cl)F
Cc1cc(c2c(c1)sc(n2)N3[C@@H](C(=C(C3=O)O)C(=O)c4cc5ccccc5o4)c6ccc(cc6)OCC=C)C
Cc1cc(c2c(c1)sc(n2)N3[C@H](C(=C(C3=O)O)C(=O)c4cc5ccccc5o4)c6ccc(cc6)OCC=C)C
Cc1c(cc(s1)[C@@H]2C3=C(CCCC3=O)NC(=C2C(=O)OC)C)COc4ccccc4Br
Cc1cc(c2c(c1)sc(n2)N3[C@@H](C(=C(C3=O)O)C(=O)c4cc5ccccc5o4)c6ccc(cc6)OCc7ccccc7)C
Cc1cc(c2c(c1)sc(n2)N3[C@H](C(=C(C3=O)O)C(=O)c4cc5ccccc5o4)c6ccc(cc6)OCc7ccccc7)C
Cc1c(cc(s1)[C@H]2C3=C(CCCC3=O)NC(=C2C(=O)OC)C)COc4ccccc4Br
Cc1cc(c2c(c1)sc(n2)N3[C@@H](C(=C(C3=O)O)C(=O)c4cc5ccccc5o4)c6ccc(c(c6)OC)OCCC(C)C)C
Cc1cc(c2c(c1)sc(n2)N3[C@H](C(=C(C3=O)O)C(=O)c4cc5ccccc5o4)c6ccc(c(c6)OC)OCCC(C)C)C
CCOC(=O)C1[C@H](C2=C(CC(CC2=O)(C)C)N=C1C)c3cc(c(s3)C)COc4ccc(cc4C)Cl
CCCCOc1ccc(cc1OC)[C@@H]2C(=C(C(=O)N2c3nc4c(cc(cc4s3)C)C)O)C(=O)c5cc6ccccc6o5
CCOC(=O)C1[C@@H](C2=C(CC(CC2=O)(C)C)N=C1C)c3cc(c(s3)C)COc4ccc(cc4C)Cl
CCCCOc1ccc(cc1OC)[C@H]2C(=C(C(=O)N2c3nc4c(cc(cc4s3)C)C)O)C(=O)c5cc6ccccc6o5
Cc1cc(c2c(c1)sc(n2)N3[C@@H](C(=C(C3=O)O)C(=O)c4cc5ccccc5o4)c6ccc(c(c6)OC)OCc7ccccc7)C
CCOC(=O)C1[C@H](C2=C(CC(CC2=O)(C)C)N=C1C)c3cc(c(cc3C)C)COc4ccc(cc4Cl)F
Cc1cc(c2c(c1)sc(n2)N3[C@H](C(=C(C3=O)O)C(=O)c4cc5ccccc5o4)c6ccc(c(c6)OC)OCc7ccccc7)C
Cc1cc(c2c(c1)sc(n2)N3[C@@H](C(=C(C3=O)O)C(=O)c4cc5ccccc5o4)c6ccc(cc6)C(C)C)C
CCOC(=O)C1[C@@H](C2=C(CC(CC2=O)(C)C)N=C1C)c3cc(c(cc3C)C)COc4ccc(cc4Cl)F
Cc1cc(c2c(c1)sc(n2)N3[C@H](C(=C(C3=O)O)C(=O)c4cc5ccccc5o4)c6ccc(cc6)C(C)C)C
Cc1cc(c2c(c1)sc(n2)N3[C@@H](C(=C(C3=O)O)C(=O)c4cc5ccccc5o4)c6ccc(cc6)N(C)C)C
CCOC(=O)C1[C@H](C2=C(CC(CC2=O)(C)C)N=C1C)c3cc(c(s3)C)COc4ccccc4Br

```
Cc1cc(c2c(c1)sc(n2)N3[C@H](C(=C(C3=O)O)C(=O)c4cc5ccccc5o4)c6ccc(cc6)N(C)C)C
CCOc1ccc2c(c1)sc(n2)N3[C@@H](C(=C(C3=O)O)C(=O)c4cc5ccccc5o4)c6ccc(cc6)OCC=C
CCOC(=O)C1[C@@H](C2=C(CC(CC2=O)(C)C)N=C1C)c3cc(c(s3)C)COc4ccccc4Br
CCOc1ccc2c(c1)sc(n2)N3[C@H](C(=C(C3=O)O)C(=O)c4cc5ccccc5o4)c6ccc(cc6)OCC=C
CCOc1ccc2c(c1)sc(n2)N3[C@@H](C(=C(C3=O)O)C(=O)c4cc5ccccc5o4)c6ccc(c(c6)OC)OCc7ccccc7
Cc1cc(c(cc1COc2ccc(cc2Cl)F)[C@@H]3C4=C(CCCC4=O)NC(=C3C(=O)OC)C)C
CCOc1ccc2c(c1)sc(n2)N3[C@H](C(=C(C3=O)O)C(=O)c4cc5ccccc5o4)c6ccc(c(c6)OC)OCc7ccccc7
Cc1cc(c(cc1COc2ccc(cc2Cl)F)[C@H]3C4=C(CCCC4=O)NC(=C3C(=O)OC)C)C
CCOc1ccc2c(c1)sc(n2)N3[C@@H](C(=C(C3=O)O)C(=O)c4cc5ccccc5o4)c6cccc(c6)Cl
CCOc1ccc2c(c1)sc(n2)N3[C@H](C(=C(C3=O)O)C(=O)c4cc5ccccc5o4)c6cccc(c6)Cl
Cc1cc(c(cc1COc2ccc(cc2C)Cl)[C@@H]3C4=C(CC(CC4=O)(C)C)NC(=C3C(=O)OC)C)C
Cc1cc(c(cc1COc2ccc(cc2C)Cl)[C@H]3C4=C(CC(CC4=O)(C)C)NC(=C3C(=O)OC)C)C
[H]/N=C/1\[C@@H](C2=CCCC[C@H]2[C@H](C1(C#N)C#N)c3cc(c(cc3C)C)COc4ccccc4Br)C#N
CCOc1ccc2c(c1)sc(n2)N3[C@@H](C(=C(C3=O)O)C(=O)c4cc5ccccc5o4)c6ccc(cc6)Br
CCOc1ccc2c(c1)sc(n2)N3[C@H](C(=C(C3=O)O)C(=O)c4cc5ccccc5o4)c6ccc(cc6)Br
[H]/N=C/1\[C@@H](C2=CCCC[C@H]2[C@@H](C1(C#N)C#N)c3cc(c(cc3C)C)COc4ccccc4Br)C#N
Cc1cc(c(cc1COc2ccccc2Br)[C@@H]3C4=C(CC(CC4=O)(C)C)NC(=C3C(=O)OC)C)C
CCCCCOc1ccc(cc1OC)[C@@H]2C(=C(C(=O)N2c3nc4ccc(cc4s3)OCC)O)C(=O)c5cc6ccccc6o5
Cc1cc(c(cc1COc2ccccc2Br)[C@H]3C4=C(CC(CC4=O)(C)C)NC(=C3C(=O)OC)C)C
CCCCCOc1ccc(cc1OC)[C@H]2C(=C(C(=O)N2c3nc4ccc(cc4s3)OCC)O)C(=O)c5cc6ccccc6o5
CCOc1ccc2c(c1)sc(n2)N3[C@@H](C(=C(C3=O)O)C(=O)c4cc5ccccc5o4)c6cccc(c6)Oc7ccccc7
CCOC(=O)C1=C(N=c2n(c(=O)/c(=C\c3c[nH]nc3c4ccccc4)/s2)[C@@H]1c5ccc(cc5)Cl)C
CCOc1ccc2c(c1)sc(n2)N3[C@H](C(=C(C3=O)O)C(=O)c4cc5ccccc5o4)c6cccc(c6)Oc7ccccc7
CCOC(=O)C1=C(N=c2n(c(=O)/c(=C\c3c[nH]nc3c4ccccc4)/s2)[C@H]1c5ccc(cc5)Cl)C
CCOC(=O)C1[C@H](C2=C(CCCC2=O)N=C1C)c3cc(c(cc3C)C)COc4c(cccc4OC)OC
CCOC(=O)c1c(nc(s1)NC(=O)CSc2nc3c(c4c(s3)CCCC4)c(=O)n2C)C
c1ccc(cc1)c2c(nc([nH]2)c3ccc(cc3)Cl)c4ccc(cc4)Oc5ccccc5
CCc1ccc2c(c1)sc(n2)N3[C@@H](C(=C(C3=O)[O-])C(=O)c4ccco4)c5ccc(cc5)Br
CCc1ccc2c(c1)sc(n2)N3[C@H](C(=C(C3=O)[O-])C(=O)c4ccco4)c5ccc(cc5)Br
CCc1ccc2c(c1)sc(n2)N3[C@H](C(=C(C3=O)[O-])C(=O)c4cccs4)c5ccc(cc5)C(C)(C)C
CCCOc1ccc(cc1)[C@@H]2/C(=C(/c3cccs3)\O)/C(=O)C(=O)N2CCC[NH+]4CCOCC4
```

```
c1ccc(cc1)[C@@H]2C(=C(C(=O)N2CCCn3ccnc3)[O-])C(=O)c4cc5ccccc5o4
c1ccc(cc1)[C@H]2C(=C(C(=O)N2CCCn3ccnc3)[O-])C(=O)c4cc5ccccc5o4
CCCOc1ccc(cc1)[C@H]2/C(=C(/c3cccs3)\O)/C(=O)C(=O)N2CCC[NH+]4CCOCC4
Cc1cccc(c1C)n2c(cc(c2C)/C=C/3\C(=NC(=O)N(C3=O)c4ccc(cc4)F)[O-])C
CCc1ccc(cc1)N2C(=O)/C(=C/c3cc(n(c3C)c4cccc(c4)C)C)/C(=NC2=S)[O-]
CCc1ccc2c(c1)sc(n2)N3[C@@H](C(=C(C3=O)[O-])C(=O)c4cccs4)c5ccc(cc5)C(C)(C)C
CCCOc1ccc(cc1)[C@@H]2/C(=C(/c3cccs3)\O)/C(=O)C(=O)N2CC[NH+](CC)CC
CCCOc1ccc(cc1)[C@H]2/C(=C(/c3cccs3)\O)/C(=O)C(=O)N2CC[NH+](CC)CC
CCn1cc(c(=O)c2c1cc(c(c2)F)N3CCN(CC3)C(=O)c4ccco4)C(=O)Nc5nc(cs5)C
Cc1ccc2c(c1)sc(n2)NC(=O)[C@@H](Cc3ccccc3)NC(=O)OCc4ccccc4
Cc1ccc2c(c1)sc(n2)NC(=O)[C@H](Cc3ccccc3)NC(=O)OCc4ccccc4
C[NH+](C)CCN1[C@@H](/C(=C(/c2ccc3c(c2)OCCO3)\O)/C(=O)C1=O)c4cc(ccc4OC)OC
C[NH+](C)CCN1[C@H](/C(=C(/c2ccc3c(c2)OCCO3)\O)/C(=O)C1=O)c4cc(ccc4OC)OC
CCc1ccc(cc1)N2C(=O)/C(=C/c3cc(n(c3C)c4ccc(cc4)C(=O)[O-])C)/C(=O)N=C2S
Cc1cc(c(n1c2cccc(c2)C(=O)[O-])C)/C=C/3\C(=NC(=O)N(C3=O)c4ccc(cc4)F)O
c1cc(ccc1[C@H]2/C(=C(/c3ccc(cc3)Cl)\O)/C(=O)C(=O)N2CCCC(=O)[O-])F
c1ccc(c(c1)[C@H]2/C(=C(/c3ccc(cc3)Cl)\O)/C(=O)C(=O)N2CCCC(=O)[O-])F
CC[NH+](CC)CCN1[C@@H](/C(=C(/c2ccccc2)\O)/C(=O)C1=O)c3cc(ccc3OC)OC
CC[NH+](CC)CCN1[C@H](/C(=C(/c2ccccc2)\O)/C(=O)C1=O)c3cc(ccc3OC)OC
CCCOc1ccc(cc1)[C@@H]2/C(=C(/c3ccc(cc3)C)\O)/C(=O)C(=O)N2CC[NH+]4CCOCC4
CCCOc1ccc(cc1)[C@H]2/C(=C(/c3ccc(cc3)C)\O)/C(=O)C(=O)N2CC[NH+]4CCOCC4
c1ccc(cc1)c2ccc(cc2)c3[nH]c(c(n3)c4ccccc4)c5ccc(cc5)C(=O)c6ccccc6
COc1cccc(c1)[C@@H]2/C(=C(/c3cc4ccccc4o3)\O)/C(=O)C(=O)N2CC[NH+]5CCOCC5
COc1cccc(c1)[C@H]2/C(=C(/c3cc4ccccc4o3)\O)/C(=O)C(=O)N2CC[NH+]5CCOCC5
c1cc(ccc1[C@@H]2/C(=C(/c3ccc(cc3)Cl)\O)/C(=O)C(=O)N2CCCC(=O)[O-])F
CN(C)c1ccc(cc1)[C@H]2C(=C(C(=O)N2CC=C)[O-])C(=O)c3ccc(cc3)Br
CN(C)c1ccc(cc1)[C@@H]2C(=C(C(=O)N2CC=C)[O-])C(=O)c3ccc(cc3)Br
CCc1c([nH]c2nc3ccccc3n2c1=O)[O-]
CC(=O)Nc1ccc(cc1)N2[C@@H](C(=C(C2=O)[O-])C(=O)c3ccc(cc3)Cl)c4ccccc4F
CC(=O)Nc1ccc(cc1)N2[C@H](C(=C(C2=O)[O-])C(=O)c3ccc(cc3)Cl)c4ccccc4F
c1ccc(c(c1)[C@@H]2/C(=C(/c3ccc(cc3)Cl)\O)/C(=O)C(=O)N2CCCC(=O)[O-])F
```

C=CCN1[C@@H](C(=C(C1=O)[O-])C(=O)c2ccc(cc2)Br)c3cccc(c3)F
CNC(=O)c1ccc(cc1)NC(=O)C2=Cc3ccccc3OC2
CCc1ccc2c(c1)sc(n2)N3[C@@H](C(=C(C3=O)[O-])C(=O)c4ccc(cc4)F)c5ccc(cc5)C(C)(C)C
CCc1ccc2c(c1)sc(n2)N3[C@H](C(=C(C3=O)[O-])C(=O)c4ccc(cc4)F)c5ccc(cc5)C(C)(C)C
COc1cc(cc(c1OC)OC)[C@@H]2/C(=C(/c3cc4ccccc4o3)\O)/C(=O)C(=O)N2CC[NH+]5CCOCC5
C[C@@H]1Cc2cc(c(cc2O1)/C=C/C(=O)OCC(=O)c3ccc(cc3)NC(=O)c4ccccc4)OC
C[C@H]1Cc2cc(c(cc2O1)/C=C/C(=O)OCC(=O)c3ccc(cc3)NC(=O)c4ccccc4)OC
CC[NH+](CC)CCN1[C@@H](/C(=C(/c2ccc(cc2)OC)\O)/C(=O)C1=O)c3ccc(cc3)OC
CC[NH+](CC)CCN1[C@H](/C(=C(/c2ccc(cc2)OC)\O)/C(=O)C1=O)c3ccc(cc3)OC
COc1cc(cc(c1OC)OC)[C@H]2/C(=C(/c3cc4ccccc4o3)\O)/C(=O)C(=O)N2CC[NH+]5CCOCC5
C=CCN1[C@H](C(=C(C1=O)[O-])C(=O)c2ccc(cc2)Br)c3ccc(cc3)F
CC1(CC2=C([C@@H](Nc3ccc(cc3N2)C(=O)c4ccccc4)c5ccc(c(c5)OC)OC)C(=O)C1)C
CC1(CC2=C([C@H](Nc3ccc(cc3N2)C(=O)c4ccccc4)c5ccc(c(c5)OC)OC)C(=O)C1)C
c1cc(ccc1[C@@H]2C(=C(C(=O)N2CCCn3cc[nH+]c3)[O-])C(=O)c4ccc5c(c4)OCCO5)Cl
Cc1ccnc(c1)NC(=O)Cc2cccc3c2cccc3
C=CCN1[C@@H](C(=C(C1=O)[O-])C(=O)c2ccc(cc2)Br)c3ccc(cc3)F
c1ccc(cc1)C2(C=C(c3c4ccccc4ccc3O2)C(c5ccccc5)(c6ccccc6)O)c7ccccc7
COc1ccc(cc1)C2(C=Cc3c(ccc-4c3Cc5c4cccc5)O2)c6ccc(cc6)OC
CC1(Cc2cccc(c2O1)OCc3ccc(cc3)C(=O)Nc4ccc(cc4)OC(F)(F)F)C
Cc1ccccc1[C@@]2(C=Cc3c(ccc4c3cc(cc4)OC(=O)C(C)C)O2)c5ccc(cc5)OC
Cc1ccccc1[C@]2(C=Cc3c(ccc4c3cc(cc4)OC(=O)C(C)C)O2)c5ccc(cc5)OC
CCOc1ccccc1/C=C/C(=O)c2cccc(c2)N3CCOCC3
CC(C)Oc1c(cccc1OC)/C=C/C(=O)c2cccc(c2)N3CCOCC3
c1ccc2c(c1)C=CC3(O2)CC[NH+](CC3)C[C@@H](c4ccc(cc4)F)O
c1ccc2c(c1)C=CC3(O2)CC[NH+](CC3)C[C@H](c4ccc(cc4)F)O
Cc1cc2c(cc1C)OC3(CC[NH+](CC3)C[C@H](c4ccc(cc4)NC(=O)C5CC5)O)C=C2
Cc1cc2c(cc1C)OC3(CC[NH+](CC3)C[C@@H](c4ccc(cc4)NC(=O)C5CC5)O)C=C2
Cc1cc2c(cc1C)OC3(CC[NH+](CC3)C[C@@H](c4ccc(cc4)NC(=O)C)O)C=C2
Cc1cc2c(cc1C)OC3(CC[NH+](CC3)C[C@H](c4ccc(cc4)NC(=O)C)O)C=C2
C/C(=N\N=C/1\NC(=O)[C@@H](S1)CC(=O)Nc2ccccc2OC)/C(C)(C)C
COc1ccc(c(c1)OC)/C=C/C(=O)c2ccc(c(c2)F)N3CCOCC3

```
C=CCOc1ccc(cc1)/C=C/C(=O)c2ccc(c(c2)F)N3CCOCC3
c1cc(cc(c1)OCc2ccc(cc2)F)C(=O)Nc3ccc(cc3)/C=C/C(=O)[O-]
CCOC(=O)/C=C/c1ccc(cc1)NC(=O)c2cccc(c2)OCc3ccc(cc3)F
Cc1cc(c(cc1c2ccc(o2)/C=N/c3c(nc4n3cccc4)c5ccco5)[N+](=O)[O-])C
c1cc(ccc1c2nnc(n2c3ccc(cc3)Br)[S-])Br
CCOC(=O)c1ccc(cc1)/N=C\2/N(C(=O)/C(=C/c3cc(n(c3C)c4cccnc4)C)/S2)C
COc1ccc(c(c1)OC)[C@H]2C(=C(C(=O)N2Cc3ccco3)[O-])C(=O)c4ccc5c(c4)OCCO5
COc1ccc(c(c1)OC)[C@@H]2C(=C(C(=O)N2Cc3ccco3)[O-])C(=O)c4ccc5c(c4)OCCO5
C[C@H]1Cc2cc(ccc2O1)C(=O)C3=C(C(=O)N([C@@H]3c4ccc(c(c4)OC)O)Cc5ccco5)[O-]
C[C@@H]1Cc2cc(ccc2O1)C(=O)C3=C(C(=O)N([C@H]3c4ccc(c(c4)OC)O)Cc5ccco5)[O-]
C[C@@H]1Cc2cc(ccc2O1)C(=O)C3=C(C(=O)N([C@@H]3c4ccc(c(c4)OC)O)Cc5ccco5)[O-]
Cc1ccc(cc1C)NC2=C/C(=N\S(=O)(=O)c3cccs3)/c4ccccc4C2=O
[H]/N=C/1\[C@H](C2=CCCC[C@H]2[C@@H](C1(C#N)C#N)c3ccc(c(c3)COc4cccc(c4)C(F)(F)F)OC)C#N
c1ccc2c(c1)-c3c4c(c(cc(c4no3)NC5CCCC5)Nc6ccc(cc6)Cl)C2=O
COC(=O)CN/C=C\C(=O)c1ccc(c(c1)Cl)Cl
Cc1ccc(cc1)C(=O)Nc2cccc(c2)/C=C/C(=O)c3ccc(cc3)O[C@H](C)C(=O)[O-]
Cc1ccc(cc1)C(=O)Nc2cccc(c2)/C=C/C(=O)c3ccc(cc3)O[C@@H](C)C(=O)[O-]
C[C@H](C(=O)[O-])Oc1ccc(cc1)C(=O)/C=C/c2cccc(c2)NC(=O)c3ccc(cc3)OC
CC(=O)Oc1cc2c(cc1Cl)[C@@H](C[C@]3(O2)CC(NC(=S)N3)(C)C)c4cccs4
CC(=O)Oc1cc2c(cc1Cl)[C@@H](C[C@@]3(O2)CC(NC(=S)N3)(C)C)c4cccs4
CC(=O)Oc1cc2c(cc1Cl)[C@H](C[C@]3(O2)CC(NC(=S)N3)(C)C)c4cccs4
CC(=O)Oc1cc2c(cc1Cl)[C@H](C[C@@]3(O2)CC(NC(=S)N3)(C)C)c4cccs4
CCc1ccc2c(c1)sc(n2)N3[C@H](C(=C(C3=O)[O-])C(=O)c4ccc(o4)C)c5ccc(cc5)C(C)(C)C
CCc1ccc2c(c1)sc(n2)N3[C@@H](C(=C(C3=O)[O-])C(=O)c4ccc(o4)C)c5ccc(cc5)C(C)(C)C
CCOC(=O)c1c(nc(s1)NC(=O)CSc2nc3c(c(c(s3)C)C)c(=O)n2CC=C)C
Cc1ccc(c(c1)C)n2c(cc(c2C)/C=C/3\C(=NC(=O)N(C3=O)c4ccc(cc4)F)[O-])C
COc1ccc(cc1)C(=O)/C=C\c2ccc(cc2)NC(=O)c3ccc(cc3)F
COc1ccccc1/C=C/C(=O)Nc2cccc(c2)C(=O)Nc3ccc(cc3)F
COc1ccccc1C(=O)Nc2ccc(cc2)OC(=O)/C=C/c3ccc(cc3)F
c1ccc(cc1)COc2cccc(c2)NC(=O)c3ccccc3F
CCOc1ccccc1/C=C/C(=O)Nc2ccc(cc2)C(=O)Nc3ccc(cc3)OC
```

```
CCOC(=O)c1ccc(cc1)N2C(=O)C(=Cc3ccc(c(c3)OC)OC)C(=N2)C(F)(F)F
CCOc1ccccc1/C=C/C(=O)Nc2cc(ccc2F)C
C[C@H]1Cc2cc(ccc2O1)C(=O)C3=C(C(=O)N([C@H]3c4cc(c(c(c4)OC)OC)OC)C[C@@H]5CCCO5)[O-]
C[C@H]1Cc2cc(ccc2O1)C(=O)C3=C(C(=O)N([C@@H]3c4cc(c(c(c4)OC)OC)OC)C[C@@H]5CCCO5)[O-]
Cc1ccc(cc1)[C@H]2C(=C(C(=O)N2C[C@@H]3CCCO3)[O-])C(=O)c4ccc5c(c4)OCCO5
Cc1ccc(cc1)[C@@H]2C(=C(C(=O)N2C[C@@H]3CCCO3)[O-])C(=O)c4ccc5c(c4)OCCO5
Cc1ccc(cc1)[C@H]2C(=C(C(=O)N2C[C@H]3CCCO3)[O-])C(=O)c4ccc5c(c4)OCCO5
Cc1ccc(cc1)[C@@H]2C(=C(C(=O)N2C[C@H]3CCCO3)[O-])C(=O)c4ccc5c(c4)OCCO5
Cc1cccc(c1)NC(=O)c2ccc(cc2)COc3cccc4c3OC(C4)(C)C
Cc1ccc(cc1)NC(=O)c2ccc(cc2)COc3cccc4c3OC(C4)(C)C
CCc1ccc(cc1)NC(=O)c2ccc(cc2)COc3cccc4c3OC(C4)(C)C
CC(C)c1ccc(cc1)NC(=O)c2ccc(cc2)COc3cccc4c3OC(C4)(C)C
CC1(Cc2cccc(c2O1)OCc3ccc(cc3)C(=O)Nc4ccc(cc4)F)C
CC1(Cc2cccc(c2O1)OCc3ccc(cc3)C(=O)Nc4ccc(cc4)OC)C
CCOc1ccc(cc1)NC(=O)c2ccc(cc2)COc3cccc4c3OC(C4)(C)C
CC1(Cc2cccc(c2O1)OCc3ccc(cc3)C(=O)Nc4cccc5c4cccc5)C
CC1(Cc2cccc(c2O1)OCc3ccc(cc3)C(=O)Nc4ccc(c(c4)F)F)C
Cc1ccc(cc1C)NC(=O)c2ccc(cc2)COc3cccc4c3OC(C4)(C)C
Cc1cc(cc(c1)NC(=O)c2ccc(cc2)COc3cccc4c3OC(C4)(C)C)C
CC1(Cc2cccc(c2O1)OCc3ccc(cc3)C(=O)Nc4ccc(c(c4)OC)OC)C
Cc1ccc(cc1F)NC(=O)c2ccc(cc2)COc3cccc4c3OC(C4)(C)C
Cc1cc(ccc1OC)NC(=O)c2ccc(cc2)COc3cccc4c3OC(C4)(C)C
CC1(Cc2cccc(c2O1)OCc3ccc(cc3)C(=O)Nc4ccc(cc4)C(=O)N)C
COc1cccc(c1)[C@H]2C(=C(C(=O)N2C[C@@H]3CCCO3)[O-])C(=O)c4ccc5c(c4)OCCO5
COc1cccc(c1)[C@@H]2C(=C(C(=O)N2C[C@@H]3CCCO3)[O-])C(=O)c4ccc5c(c4)OCCO5
COc1cccc(c1)[C@H]2C(=C(C(=O)N2C[C@H]3CCCO3)[O-])C(=O)c4ccc5c(c4)OCCO5
COc1cccc(c1)[C@@H]2C(=C(C(=O)N2C[C@H]3CCCO3)[O-])C(=O)c4ccc5c(c4)OCCO5
COc1cc(cc(c1OC)OC)[C@H]2C(=C(C(=O)N2Cc3ccco3)[O-])C(=O)c4ccc5c(c4)OCCO5
COc1cc(cc(c1OC)OC)[C@@H]2C(=C(C(=O)N2Cc3ccco3)[O-])C(=O)c4ccc5c(c4)OCCO5
COc1cc(ccc1O)[C@H]2C(=C(C(=O)N2Cc3ccco3)[O-])C(=O)c4ccc5c(c4)OCCO5
COc1cc(ccc1O)[C@@H]2C(=C(C(=O)N2Cc3ccco3)[O-])C(=O)c4ccc5c(c4)OCCO5
```

```
Cc1c(c(=O)n(n1C)c2ccccc2)N/C=C/3\c4ccccc4C(=O)N(C3=O)c5ccccc5OC
COc1ccc(cc1)S(=O)(=O)NC2=C/C(=N\c3ccc(cc3)O)/C(=O)c4c2cccc4
Cc1cc(c(c(c1)C)S(=O)(=O)NC2=C/C(=N/S(=O)(=O)c3ccccc3)/C(=O)c4c2cccc4)C
CCc1ccc(cc1)S(=O)(=O)N=C2C=C(C(=O)c3c2cccc3)[N-]S(=O)(=O)c4ccccc4
CCOc1ccc(cc1)[C@H]2C(=C(C(=O)N2C[C@@H]3CCCO3)[O-])C(=O)c4ccc5c(c4)C[C@@H](O5)C
CCOc1ccc(cc1)[C@@H]2C(=C(C(=O)N2C[C@@H]3CCCO3)[O-])C(=O)c4ccc5c(c4)C[C@@H](O5)C
CCOc1ccc(cc1)[C@H]2C(=C(C(=O)N2C[C@H]3CCCO3)[O-])C(=O)c4ccc5c(c4)C[C@@H](O5)C
CCOc1ccc(cc1)[C@@H]2C(=C(C(=O)N2C[C@H]3CCCO3)[O-])C(=O)c4ccc5c(c4)C[C@@H](O5)C
C[C@H]1Cc2cc(ccc2O1)C(=O)C3=C(C(=O)N([C@H]3c4cccc(c4)OC)C[C@@H]5CCCO5)[O-]
C[C@H]1Cc2cc(ccc2O1)C(=O)C3=C(C(=O)N([C@@H]3c4cccc(c4)OC)C[C@@H]5CCCO5)[O-]
COc1ccc(cc1Cl)NCc2c3ccccc3ccc2OCc4ccc(cc4)F
COc1ccc(cc1Cl)NCc2c3ccccc3ccc2OCc4ccccc4F
COc1ccc(cc1)C(=O)Nc2cccc(c2)/C=C/C(=O)c3ccc(c(c3)OC)OC(F)F
COc1ccc(cc1)C(=O)Nc2cccc(c2)/C=C/C(=O)c3ccc(c(c3)F)F
COc1ccc(cc1)C(=O)Nc2cccc(c2)/C=C/C(=O)c3ccc(cc3F)F
CN(C)C(=O)COc1ccc(cc1)C(=O)/C=C/c2cccc(c2)NC(=O)c3ccc(cc3)OC
COc1ccc(cc1)C(=O)Nc2cccc(c2)/C=C/C(=O)c3ccc(c(c3)F)N4CCOCC4
COc1ccc(cc1)C(=O)Nc2cccc(c2)/C=C/C(=O)c3ccccc3
COc1ccc(cc1)C(=O)Nc2cccc(c2)/C=C/C(=O)c3ccc(cc3OC)OC
COc1ccc(cc1)C(=O)Nc2cccc(c2)/C=C/C(=O)c3ccc(cc3)O
COc1ccc(cc1)C(=O)Nc2cccc(c2)/C=C/C(=O)c3ccc(cc3)F
COc1ccc(cc1)C(=O)/C=C/c2cccc(c2)NC(=O)c3ccc(cc3)OC
COc1ccc(cc1)C(=O)Nc2cccc(c2)/C=C/C(=O)c3cccc(c3)OC
COc1ccc(cc1)C(=O)Nc2cccc(c2)/C=C/C(=O)c3ccc4c(c3)OCO4
COc1ccc(cc1)C(=O)Nc2cccc(c2)/C=C/C(=O)c3ccc(c(c3)OC)OC
c1ccc(cc1)Nc2ccc(cc2)NCc3ccccc3OCc4ccccc4F
CCCCc1ccc(cc1)NCc2ccccc2OCc3ccccc3F
Cc1ccc(cc1)NC(=O)/C(=C\C2=Cc3ccccc3O[C@@H]2c4ccccc4)/C#N
Cc1ccc(cc1)NC(=O)/C(=C\C2=Cc3ccccc3O[C@H]2c4ccccc4)/C#N
Cc1ccc(cc1)C(=O)C2=C(C(=O)N([C@@H]2c3ccc(cc3)Br)c4ccc(cc4)F)[O-]
Cc1ccc(cc1)C(=O)C2=C(C(=O)N([C@H]2c3ccc(cc3)Br)c4ccc(cc4)F)[O-]
```

```
COc1cccc(c1)C(=O)C2=C(C(=O)N([C@@H]2c3cccs3)CCCn4ccnc4)[O-]
COc1cccc(c1)C(=O)C2=C(C(=O)N([C@H]2c3cccs3)CCCn4ccnc4)[O-]
Cc1ccccc1NC(=O)C2=C(NC3=C([C@H]2c4ccc(cc4)[N+](=O)[O-])C(=O)CC(C3)(C)C)C
Cc1cccc(c1)[C@@H]2[C@@H]3[C@@H](C(=O)N(C3=O)c4ccc(cc4)Br)C5(O2)C(=O)c6ccccc6C5=O
CC(=O)O[C@@H](CCN1CC2CC[NH+]1CC2)c3ccccc3
CCC(=O)Nc1ccc(cc1)[C@H](C[NH+]2CCC3(CC2)C=Cc4ccccc4O3)O
CCC(=O)Nc1ccc(cc1)[C@@H](C[NH+]2CCC3(CC2)C=Cc4ccccc4O3)O
c1ccc(cc1)COc2ccc(cc2)NCc3ccc(cc3)F
CC(C)COc1ccccc1CNc2ccc(cc2)OCc3ccccc3
CC(C)Oc1ccc(cc1)NCc2ccccc2F
CC(C)Oc1ccc(cc1)NCc2ccccc2OCCc3ccccc3
c1ccc(cc1)COc2cccc(c2)NCc3ccc(cc3)F
CC(C)CCOc1ccccc1CNc2cccc(c2)OCc3ccccc3
CCOc1ccc(cc1)N[C@@H](C)c2ccccc2F
CCOc1ccc(cc1)N[C@H](C)c2ccccc2F
CCOc1ccc(cc1OCC)NCc2ccccc2F
C[C@H](c1ccccc1F)Nc2ccc3c(c2)OCCO3
C[C@@H](c1ccccc1F)Nc2ccc3c(c2)OCCO3
c1cc(cc(c1)Cl)NNC(=O)c2cc(cnc2)Br
CCOc1ccccc1CNc2cc(ccc2F)C
Cc1ccc(c(c1)NCc2ccc(cc2)OCC=C)F
Cc1ccc(c(c1)N[C@H](C)c2ccc(cc2)OC(C)C)F
Cc1ccc(c(c1)N[C@@H](C)c2ccc(cc2)OC(C)C)F
C[C@@H](c1cc(ccc1F)F)Nc2ccc(cc2)OC(C)C
C[C@H](c1cc(ccc1F)F)Nc2ccc(cc2)OC(C)C
C[C@@H](c1ccccc1F)Nc2ccc(cc2)OC(C)C
C[C@H](c1ccccc1F)Nc2ccc(cc2)OC(C)C
C[C@@H](c1c(cccc1F)F)Nc2ccc(cc2)OC(C)C
C[C@H](c1c(cccc1F)F)Nc2ccc(cc2)OC(C)C
CC(C)Oc1ccc(cc1)NCc2c(cccc2F)F
CC1([C@@]2(C=Cc3cc(ccc3O2)[N+](=O)[O-])c4ccccc4N1CCO)C
```

```
CC1([C@]2(C=Cc3cc(ccc3O2)[N+](=O)[O-])c4ccccc4N1CCO)C
CC(C)CNC(=O)c1cccc(c1)NCc2ccccc2OCc3ccc(cc3)F
CC(C)CNC(=O)c1cccc(c1)NCc2ccccc2OCc3ccccc3F
CC(C)CNC(=O)c1cccc(c1)NCc2c3ccccc3ccc2OCc4ccccc4F
CC(C)CNC(=O)c1cccc(c1)NCc2c3ccccc3ccc2OCc4ccc(cc4)F
Cc1c(cccc1NCc2ccc(c(c2)OC)OCc3ccccc3F)C(=O)[O-]
Cc1c(cccc1NCc2ccccc2OCc3ccc(cc3)F)C(=O)[O-]
Cc1c(cccc1NCc2ccccc2OCc3ccccc3F)C(=O)[O-]
Cc1ccc(cc1NCc2ccccc2OCc3ccccc3F)C(=O)[O-]
Cc1c(cccc1NCc2cccc(c2OCc3ccccc3F)OC)C(=O)[O-]
Cc1ccc(cc1NCc2cccc(c2OCc3ccccc3F)OC)C(=O)[O-]
Cc1c(cccc1NCc2cccc(c2OCc3ccc(cc3)F)OC)C(=O)[O-]
Cc1ccc(cc1NCc2cccc(c2OCc3ccc(cc3)F)OC)C(=O)[O-]
Cc1c(cccc1NCc2cccc(c2)OCc3ccccc3F)C(=O)[O-]
Cc1c(cccc1NCc2c3ccccc3ccc2OCc4ccccc4F)C(=O)[O-]
Cc1ccc(cc1NCc2c3ccccc3ccc2OCc4ccccc4F)C(=O)[O-]
Cc1c(cccc1NCc2c3ccccc3ccc2OCc4ccc(cc4)F)C(=O)[O-]
Cc1ccc(cc1NCc2c3ccccc3ccc2OCc4ccc(cc4)F)C(=O)[O-]
c1ccc(cc1)[C@@H]2C(=Cc3ccccc3O2)C=O
c1ccc(cc1)[C@H]2C(=Cc3ccccc3O2)C=O
c1ccc(cc1)[C@@H]2C(=Cc3ccccc3O2)C(=O)[O-]
c1ccc(cc1)[C@H]2C(=Cc3ccccc3O2)C(=O)[O-]
COc1ccc(cc1)C(=O)Nc2cccc(c2)/C=C/C(=O)c3ccc(c(c3)F)OC
COc1ccc(cc1)C(=O)Nc2cccc(c2)/C=C/C(=O)c3cccc(c3)F
COc1ccc(cc1)C(=O)Nc2cccc(c2)/C=C/3\COc4ccc(cc4C3=O)OC
c1ccc(c(c1)/C=C/C(=O)c2ccc(c(c2)F)N3CCOCC3)OCc4ccccn4
CC(=O)Nc1cccc(c1)C(=O)/C=C/c2ccccc2OCC=C
Cc1cccc(c1)NC(=O)c2ccccc2NC(=O)C3=Cc4ccccc4OC3
C[C@H](c1cccc(c1)NC(=O)c2ccccc2)N(C)C(=O)C3=Cc4ccccc4OC3
C[C@@H](c1cccc(c1)NC(=O)c2ccccc2)N(C)C(=O)C3=Cc4ccccc4OC3
COc1ccc(cc1)[C@@]2(C=Cc3c(ccc4c3cc5ccccc5c4)O2)c6ccccc6F
```

```
COc1ccc(cc1)[C@]2(C=Cc3c(ccc4c3cc5ccccc5c4)O2)c6ccccc6F
COc1ccc(cc1)C2(C=Cc3c(ccc4c3cc5ccccc5c4)O2)c6ccc(cc6)OC
CC(C)Oc1ccccc1/C=C/C(=O)c2cccc(c2)NC(=O)C
c1ccc(c(c1)C(=O)Nc2ccc3c(c2)CCO3)F
CC(C)CC1=Cc2ccccc2O[C@]13C=Cc4ccc(cc4O3)N(Cc5ccccc5)Cc6ccccc6
Cc1cccc(c1)NCc2ccccc2OC(C)C
CC(C)Oc1ccccc1CNc2ccccc2F
CC(C)Oc1ccccc1CNc2ccc(cc2)F
CC(C)Oc1ccccc1CNc2cccc(c2)F
Cc1ccc(cc1C)NCc2ccccc2OC(C)C
CCOc1ccccc1/C=C/C(=O)c2ccc(cc2O)NC(=O)C
CCOc1ccccc1/C=C/C(=O)c2cc(ccc2O)NC(=O)C
c1ccc(cc1)C2(C=Cc3c4ccc(cc4ccc3O2)C(=O)[O-])c5ccccc5
CC(C)[C@@H](C(=O)[O-])NC(=O)c1ccc2c(c1)ccc3c2C=CC(O3)(c4ccccc4)c5ccccc5
c1ccc(cc1)C2(C=Cc3c4ccc(cc4ccc3O2)C(=O)NCCCCCC(=O)[O-])c5ccccc5
c1ccc(cc1)C2(C=Cc3c4ccc(cc4ccc3O2)C(=O)NCCCCC(=O)[O-])c5ccccc5
c1ccc(cc1)C2(C=Cc3c4cccc(c4ccc3O2)C(=O)[O-])c5ccccc5
c1ccc(cc1)C2(C=Cc3c4ccc(cc4ccc3O2)C(=O)NCCCCCCC(=O)[O-])c5ccccc5
c1ccc(cc1)C2(C=Cc3c4cccc(c4ccc3O2)C(=O)NCCCCCCC(=O)[O-])c5ccccc5
c1ccc(cc1)C2(C=Cc3c4cccc(c4ccc3O2)C(=O)NCCCCCC(=O)[O-])c5ccccc5
c1ccc(cc1)C2(C=Cc3c4cccc(c4ccc3O2)C(=O)NCCCCC(=O)[O-])c5ccccc5
c1ccc(cc1)C2(C=Cc3c4ccc(cc4ccc3O2)C(=O)NCCCCCCCC(=O)[O-])c5ccccc5
c1ccc(cc1)C2(C=Cc3c4ccc(cc4ccc3O2)C(=O)NCCCC(=O)[O-])c5ccccc5
CC(=O)Nc1cccc(c1)C(=O)/C=C/c2ccccc2OCc3ccccc3
Cc1c(cccc1NC(=O)C2=Cc3ccccc3OC2)C(=O)NC
c1ccc(c(c1)C(=O)Nc2ccc(c(c2)C#N)OCC(F)(F)F)F
Cc1ccc(cc1)NC(=O)/C(=C/C2=Cc3ccccc3O[C@@H]2c4ccccc4)/C#N
Cc1ccc(cc1)NC(=O)/C(=C/C2=Cc3ccccc3O[C@H]2c4ccccc4)/C#N
c1ccc(cc1)C2(C=Cc3ccc4ccccc4c3O2)c5ccccc5
CCOC(=O)c1ccc(cc1)NC(=O)/C=C/C2=Cc3ccccc3O[C@H]2C
CCOc1ccc(cc1)NC(=O)/C(=C/C2=Cc3ccccc3O[C@@H]2c4ccccc4)/C#N
```

```
CCOc1ccc(cc1)NC(=O)/C(=C/C2=Cc3ccccc3O[C@H]2c4ccccc4)/C#N
c1ccc(c(c1)CNc2ccc(cc2)OC3CCCC3)F
Cc1ccccc1CNc2ccc3c(c2)CCO3
c1cc2c(cc1NCc3ccc(cc3F)Br)CCO2
c1cc(cc(c1)F)CNc2ccc3c(c2)CCO3
c1ccc2c(c1)cccc2CNc3ccc4c(c3)CCO4
c1cc(c(c(c1)Cl)CNc2ccc3c(c2)CCO3)F
c1ccc(c(c1)CNc2ccc3c(c2)CCO3)F
Cc1ccccc1COc2ccccc2CNc3ccc(cc3)F
c1ccc2c(c1)C(=C[C@@H](O2)c3cccc(c3)[N+](=O)[O-])Cl
c1ccc2c(c1)C(=C[C@H](O2)c3cccc(c3)[N+](=O)[O-])Cl
CCc1cccc(c1NC(=O)COc2ccc3c(c2)OC(C=C3)(C)C)CC
c1ccc(cc1)COc2ccc3c(c2)OC4(CC[NH2+]CC4)C=C3
c1ccc(cc1)COc2ccc3c(c2)O[C@@]4(CC[NH2+]C4)C=C3
c1ccc(cc1)COc2ccc3c(c2)O[C@]4(CC[NH2+]C4)C=C3
c1ccc(cc1)COc2cccc3c2C=C[C@@]4(O3)CC[NH2+]C4
c1ccc(cc1)COc2cccc3c2C=C[C@]4(O3)CC[NH2+]C4
Cc1ccc(c(c1)NCc2cccc3c2OCCO3)F
CC(C)Oc1ccc(cc1)NCc2cccc(c2F)F
C[C@@H](c1ccc(c(c1)F)OC)Nc2cccc(c2)COC
C[C@H](c1ccc(c(c1)F)OC)Nc2cccc(c2)COC
CCOc1ccc(cc1OC)N[C@@H](C)c2ccccc2F
CCOc1ccc(cc1OC)N[C@H](C)c2ccccc2F
CCOc1cc(ccc1OC)N[C@@H](C)c2ccccc2F
CCOc1cc(ccc1OC)N[C@H](C)c2ccccc2F
CCOc1cc(ccc1OC)NCc2ccccc2F
C=CCOc1ccc(cc1)CNc2ccc3c(c2)CCO3
c1cc2c(cc1NCc3cc(ccc3F)F)CCO2
CC(C)(C)c1ccc(cc1)CNc2ccc3c(c2)CCO3
c1ccc2cc(ccc2c1)CNc3ccc4c(c3)CCO4
c1cc2c(cc1NCc3cc(ccc3F)Br)CCO2
```

```
c1cc(ccc1CNc2ccc3c(c2)CCO3)F
c1cc2c(cc1NCc3ccc(cc3F)F)CCO2
c1cc(ccc1CNc2ccc3c(c2)CCO3)OC(F)F
CCOc1ccccc1CNc2ccc3c(c2)CCO3
Cc1ccc(cc1)CNc2ccc3c(c2)CCO3
CCc1ccc(cc1)CNc2ccc3c(c2)CCO3
c1cc(c(c(c1)F)CNc2ccc3c(c2)CCO3)F
c1cc(c(cc1CNc2ccc3c(c2)CCO3)F)F
CC(C)c1ccc(cc1)CNc2ccc3c(c2)CCO3
C[C@@H](c1c(cccc1F)F)Nc2ccc3c(c2)CCO3
C[C@H](c1c(cccc1F)F)Nc2ccc3c(c2)CCO3
C[C@@H](c1ccc(c(c1)F)F)Nc2ccc3c(c2)CCO3
C[C@H](c1ccc(c(c1)F)F)Nc2ccc3c(c2)CCO3
C[C@@H](c1ccc(cc1F)F)Nc2ccc3c(c2)CCO3
C[C@H](c1ccc(cc1F)F)Nc2ccc3c(c2)CCO3
c1ccc(cc1)CNc2ccc3c(c2)CCO3
C[C@@H](c1ccc(c(c1)F)OC)Nc2ccc3c(c2)CCO3
C[C@H](c1ccc(c(c1)F)OC)Nc2ccc3c(c2)CCO3
C[C@H](c1ccc(cc1)C(C)C)Nc2ccc3c(c2)CCO3
C[C@@H](c1ccc(cc1)C(C)C)Nc2ccc3c(c2)CCO3
C[C@@H](c1cc(ccc1F)F)Nc2ccc3c(c2)CCO3
C[C@H](c1cc(ccc1F)F)Nc2ccc3c(c2)CCO3
C[C@@H](c1ccccc1F)Nc2ccc3c(c2)CCO3
C[C@H](c1ccccc1F)Nc2ccc3c(c2)CCO3
C[C@@H](c1ccc(cc1)F)Nc2ccc3c(c2)CCO3
C[C@H](c1ccc(cc1)F)Nc2ccc3c(c2)CCO3
C[C@@H](c1cccc(c1)F)Nc2ccc3c(c2)CCO3
C[C@H](c1cccc(c1)F)Nc2ccc3c(c2)CCO3
CC(C)c1ccc(cc1)NCc2ccccc2OCC=C
Cc1ccc(cc1C)N[C@@H](C)c2ccc(cc2F)OC
Cc1ccc(cc1C)N[C@H](C)c2ccc(cc2F)OC
```

```
CC(C)Oc1ccccc1CNc2cccc(c2)C#N
C=CCOc1ccccc1CNc2cccc(c2)C#N
Cc1cc(cc(c1)NCc2ccccc2OCC=C)C
Cc1ccc(cc1F)NCc2ccccc2OC(C)C
Cc1ccc(cc1F)NCc2ccccc2OCC=C
CCc1cccc(c1)NCc2ccccc2OC(C)C
CCc1cccc(c1)NCc2ccccc2OCC=C
CC(C)c1cccc(c1)NCc2ccccc2OC(C)C
CC(C)c1cccc(c1)NCc2ccccc2OCC=C
Cc1ccc(c(c1)F)NCc2ccccc2OC(C)C
Cc1ccc(c(c1)F)NCc2ccccc2OCC=C
Cc1ccc(c(c1)NCc2ccc(cc2)OC(C)C)F
Cc1ccc(c(c1)NCc2ccccc2OC(C)C)F
Cc1ccc(c(c1)NCc2ccccc2OCC=C)F
C=CCOc1ccccc1CNc2c(cccc2F)F
CC(C)Oc1ccccc1CNc2ccc(c(c2)F)OC
COc1ccc(cc1F)NCc2ccccc2OCC=C
CC(C)Oc1ccccc1CNc2cccc(c2)COC
COCc1cccc(c1)NCc2ccccc2OCC=C
C[C@@H](c1ccc(cc1F)OC)Nc2cccc(c2)COC
C[C@H](c1ccc(cc1F)OC)Nc2cccc(c2)COC
CC(C)Oc1ccccc1CNc2cccc(c2)CO
C=CCOc1ccccc1CNc2cccc(c2)CO
C[C@@H](c1ccc(cc1F)OC)Nc2cccc(c2)CO
C[C@H](c1ccc(cc1F)OC)Nc2cccc(c2)CO
CC(C)Oc1ccc(cc1)CNc2ccc3c(c2)CCO3
CC(C)Oc1ccccc1CNc2ccc3c(c2)CCO3
C=CCOc1ccccc1CNc2ccc3c(c2)CCO3
C[C@@H](c1ccc(cc1F)OC)Nc2ccc3c(c2)CCO3
C[C@H](c1ccc(cc1F)OC)Nc2ccc3c(c2)CCO3
COc1ccc(cc1O)NCc2ccccc2OCC=C
```

```
COCc1cccc(c1)NCc2ccc3c(c2)CCO3
COc1ccc(cc1F)CNc2ccc3c(c2)CCO3
Cc1cc(ccc1NCc2ccccc2F)OC(C)C
Cc1cc(ccc1N[C@@H](C)c2ccccc2F)OC(C)C
Cc1cc(ccc1N[C@H](C)c2ccccc2F)OC(C)C
CCOc1ccc(cc1CO)NCc2cc(ccc2F)Br
CCOc1ccc(cc1CO)NCc2ccc(cc2)F
CCOc1ccc(cc1CO)NCc2ccc(cc2F)F
CCOc1ccc(cc1CO)NCc2cccc(c2)OC
CCOc1ccc(cc1CO)NCc2cccc(c2)F
CCOc1ccc(cc1CO)NCc2ccccc2C
CCOc1ccc(cc1CO)NCc2ccc(cc2)C
CCc1ccc(cc1)CNc2ccc(c(c2)CO)OCC
CCOc1ccc(cc1CO)NCc2c(cccc2F)F
CCOc1ccc(cc1CO)NCc2ccc(c(c2)F)F
CCOc1ccc(cc1CO)N[C@@H](C)c2ccccc2
CCOc1ccc(cc1CO)N[C@H](C)c2ccccc2
CCOc1ccc(cc1CO)N[C@@H](C)c2ccc(cc2)C
CCOc1ccc(cc1CO)N[C@H](C)c2ccc(cc2)C
CC[C@@H](c1ccccc1)Nc2ccc(c(c2)CO)OCC
CC[C@H](c1ccccc1)Nc2ccc(c(c2)CO)OCC
CCOc1ccc(cc1CO)N[C@@H](C)c2ccc(cc2)O
CCOc1ccc(cc1CO)N[C@H](C)c2ccc(cc2)O
CCOc1ccc(cc1CO)N[C@@H](C)c2ccc(cc2)Cl
CCOc1ccc(cc1CO)N[C@H](C)c2ccc(cc2)Cl
CCOc1ccc(cc1CO)N[C@@H](C)c2ccccc2O
CCOc1ccc(cc1CO)N[C@H](C)c2ccccc2O
CCOc1ccc(cc1CO)N[C@@H](C)c2ccccc2F
CCOc1ccc(cc1CO)N[C@H](C)c2ccccc2F
CCOc1ccc(cc1CO)N[C@@H](C)c2ccc(cc2)F
CCOc1ccc(cc1CO)N[C@H](C)c2ccc(cc2)F
```

```
CCOc1ccc(cc1CO)N[C@@H](C)c2cccc(c2)O
CCOc1ccc(cc1CO)N[C@H](C)c2cccc(c2)O
CCOc1ccc(cc1CO)N[C@@H](C)c2ccccc2C
CCOc1ccc(cc1CO)N[C@H](C)c2ccccc2C
CCOc1ccc(cc1CO)NCc2c(cccc2Cl)F
CCOc1ccc(cc1CO)NCc2ccc(c(c2)Br)F
CCOc1ccc(cc1CO)NCc2cccc(c2)O
CCOc1ccc(cc1CO)N[C@@H](C)c2cccc(c2)F
CCOc1ccc(cc1CO)N[C@H](C)c2cccc(c2)F
CCOc1ccc(cc1CO)NCc2ccccc2F
CCOc1ccc(cc1CO)NCc2ccccc2
CCOc1ccc(cc1CO)NCc2ccc(cc2F)Br
CCOc1ccc(cc1CO)NCc2cc(ccc2F)F
CCOc1ccc(cc1CO)N[C@@H](C)c2ccc(cc2)I
CCOc1ccc(cc1CO)N[C@H](C)c2ccc(cc2)I
CCOc1ccc(cc1CO)NC(=O)c2ccccc2F
CCOc1ccc(cc1CO)NC(=O)c2cccc(c2)F
c1ccc(cc1)[C@@H]2C=C(c3ccccc3O2)c4ccccc4
c1ccc(cc1)[C@H]2C=C(c3ccccc3O2)c4ccccc4
c1cc(ccc1[C@@H]2C=Cc3cc(ccc3O2)Cl)Cl
c1cc(ccc1[C@H]2C=Cc3cc(ccc3O2)Cl)Cl
CC1=Cc2ccc(cc2O[C@@]13C=Cc4ccccc4O3)N(Cc5ccccc5)Cc6ccccc6
CC1=Cc2ccc(cc2O[C@]13C=Cc4ccccc4O3)N(Cc5ccccc5)Cc6ccccc6
CCOC1=C([C@@H](Oc2c1cccc2)C)C
CCOC1=C([C@H](Oc2c1cccc2)C)C
Cc1c(c2c(cc1N)C=CC(O2)(C)C)C
Cc1cc(c(c2c1OC(C=C2)(C)C)C)N
Cc1c(c2c(c(c1N)C)C=CC(O2)(C)C)C
CC1=Cc2cc(ccc2O[C@@]13C=Cc4cc(ccc4O3)[N+](=O)[O-])[N+](=O)[O-]
CC1=Cc2cc(ccc2O[C@]13C=Cc4cc(ccc4O3)[N+](=O)[O-])[N+](=O)[O-]
CCOC1=C[C@@H](Oc2c1cc(cc2)C)c3ccc(cc3)OC
```

```
CCOC1=C[C@H](Oc2c1cc(cc2)C)c3ccc(cc3)OC
CC(C)Oc1cccc(c1)CNc2ccc3c(c2)CCO3
CC(C)Oc1ccccc1CNc2ccc(cc2)CCO
C=CCOc1ccccc1CNc2ccc(cc2)CCO
Cc1ccc(cc1)NC(=O)c2ccc(cc2)OC(=O)/C=C/c3ccc(c(c3)OC)OCc4ccccc4
Cc1ccc(cc1)NC(=O)c2ccc(cc2)OC(=O)/C=C/c3ccc(cc3)OCc4ccccc4
COc1ccc(cc1)NC(=O)c2ccc(cc2)OC(=O)/C=C/c3ccc(cc3)OCc4ccccc4
Cc1ccc(c(c1)NC(=O)c2ccc(cc2)OC(=O)/C=C/c3ccc(cc3)OCc4ccccc4)C
COc1ccc(cc1)NC(=O)c2ccc(cc2)OC(=O)/C=C/c3ccc(c(c3)OC)OCc4ccccc4
Cc1ccc(c(c1)NC(=O)c2ccc(cc2)OC(=O)/C=C/c3ccc(c(c3)OC)OCc4ccccc4)C
COc1ccc(cc1)NC(=O)c2ccc(cc2)OC(=O)/C=C/c3ccccc3OC
CCOc1ccccc1/C=C/C(=O)Oc2ccc(cc2)C(=O)Nc3ccc(cc3)C
CCOc1ccccc1/C=C/C(=O)Oc2ccc(cc2)C(=O)Nc3ccccc3
CCOc1ccccc1/C=C/C(=O)Oc2ccc(cc2)C(=O)Nc3ccc(cc3)OC
CCOc1ccccc1/C=C/C(=O)Oc2ccc(cc2)C(=O)Nc3cc(ccc3C)C
CCCOc1ccccc1/C=C/C(=O)Oc2ccc(cc2)C(=O)Nc3ccc(cc3)C
CCCOc1ccccc1/C=C/C(=O)Oc2ccc(cc2)C(=O)Nc3ccccc3
CCCOc1ccccc1/C=C/C(=O)Oc2ccc(cc2)C(=O)Nc3ccc(cc3)OC
CCCOc1ccccc1/C=C/C(=O)Oc2ccc(cc2)C(=O)Nc3cc(ccc3C)C
CCCOc1ccccc1/C=C/C(=O)Oc2ccc(cc2)C(=O)Nc3ccc(cc3)Cl
CCCOc1ccccc1/C=C/C(=O)Oc2ccc(cc2)C(=O)Nc3ccc(cc3C)C
Cc1cc(ccc1OC)NCc2ccccc2F
Cc1c(cccc1F)NCc2ccccc2OCC=C
c1cc(c(cc1CNc2ccc3c(c2)CCO3)F)Cl
CCOc1ccc(cc1CO)NCc2ccc(c(c2)F)Cl
c1ccc(c(c1)/C=C/C(=O)c2ccc(cc2)N3CCOCC3)OCc4cccnc4
CC1(C=Cc2cccc(c2O1)N3CCN(CC3)C(=O)OC(C)(C)C)C
Cc1ccc(cc1)[C@@H]2C=C(c3ccccc3O2)Cl
Cc1ccc(cc1)[C@H]2C=C(c3ccccc3O2)Cl
CC(C)C1=Cc2ccccc2O[C@@]13C=Cc4ccc(cc4O3)N(Cc5ccccc5)Cc6ccccc6
CC(C)C1=Cc2ccccc2O[C@]13C=Cc4ccc(cc4O3)N(Cc5ccccc5)Cc6ccccc6
```

```
Cc1cc(ccc1OC)NCc2cc(ccc2F)F
Cc1cc(ccc1OC)NCc2ccc(cc2)OC(C)C
Cc1cc(ccc1OC)NCc2ccccc2OCC=C
Cc1cc(ccc1OC)NCc2ccc(cc2)OCC=C
Cc1cc(ccc1OC)NCc2ccccc2OC(C)C
Cc1cc(ccc1OC)NCc2c(cccc2F)F
Cc1cc(ccc1OC)NCc2ccc(cc2F)F
Cc1cc(ccc1OC)N[C@@H](C)c2c(cccc2F)F
Cc1cc(ccc1OC)N[C@H](C)c2c(cccc2F)F
Cc1cc(ccc1OC)N[C@@H](C)c2ccccc2F
Cc1cc(ccc1OC)N[C@H](C)c2ccccc2F
Cc1cc(ccc1OC)N[C@@H](C)c2ccc(cc2)F
Cc1cc(ccc1OC)N[C@H](C)c2ccc(cc2)F
CCOc1ccc(cc1)[C@@H](C)Nc2ccc(c(c2)C)OC
CCOc1ccc(cc1)[C@H](C)Nc2ccc(c(c2)C)OC
Cc1cc(ccc1OC)N[C@@H](C)c2ccc(cc2F)F
Cc1cc(ccc1OC)N[C@H](C)c2ccc(cc2F)F
Cc1cc(ccc1OC)N[C@@H](C)c2cc(ccc2F)F
Cc1cc(ccc1OC)N[C@H](C)c2cc(ccc2F)F
Cc1cc(ccc1OC)N[C@@H](C)c2cccc(c2)F
Cc1cc(ccc1OC)N[C@H](C)c2cccc(c2)F
Cc1cc(ccc1OC)N[C@@H](C)c2ccc(c(c2)F)OC
Cc1cc(ccc1OC)N[C@H](C)c2ccc(c(c2)F)OC
Cc1cc(ccc1OC)N[C@@H](C)c2ccc(cc2F)OC
Cc1cc(ccc1OC)N[C@H](C)c2ccc(cc2F)OC
Cc1cc(ccc1OC)NCc2cccc(c2)OC(C)C
C[C@@H](c1ccccc1F)Nc2cc3c(cc2N)OCCO3
C[C@H](c1ccccc1F)Nc2cc3c(cc2N)OCCO3
Cc1cc(ccc1OC)NC[C@@H](c2ccccc2F)O
Cc1cc(ccc1OC)NC[C@H](c2ccccc2F)O
Cc1cc(ccc1OC)NC[C@@H](c2c(cccc2F)F)O
```

```
Cc1cc(ccc1OC)NC[C@H](c2c(cccc2F)F)O
Cc1cc(ccc1OC)NCc2cccc(c2F)F
Cc1cc(ccc1OC)NCc2ccc(cc2F)C#N
Cc1cc(ccc1OC)NCc2cc(ccc2F)C#N
C[C@H](c1ccccc1NCc2ccccc2OC(C)C)O
C[C@@H](c1ccccc1NCc2ccccc2OC(C)C)O
C[C@@H](c1ccccc1NCc2ccccc2OCC=C)O
C[C@H](c1ccccc1NCc2ccccc2OCC=C)O
Cc1ccc(cc1NCc2ccccc2OC(C)C)C#N
Cc1ccc(cc1NCc2ccccc2OCC=C)C#N
Cc1cc(ccc1F)CNc2ccc3c(c2)CCO3
CCOc1ccc(cc1CO)NCc2ccc(c(c2)C)F
Cc1cc(ccc1CNc2ccc3c(c2)CCO3)F
CCOc1ccc(cc1CO)NCc2ccc(cc2C)F
CCOc1ccccc1CNc2ccc(c(c2)C#N)F
CCOc1ccc(cc1)[C@@H](C)Nc2ccc(c(c2)C#N)F
CCOc1ccc(cc1)[C@H](C)Nc2ccc(c(c2)C#N)F
CCOc1ccccc1[C@@H](C)Nc2ccc(c(c2)C#N)F
CCOc1ccccc1[C@H](C)Nc2ccc(c(c2)C#N)F
C=CCOc1ccc(cc1)CNc2ccc(c(c2)C#N)F
C#CCOc1ccccc1CNc2ccc(c(c2)C#N)F
c1cc(c2c(c1)OCCO2)CNc3ccc(c(c3)C#N)F
c1ccc(c(c1)CNc2ccc(c(c2)C#N)F)OCC#N
CC(C)Oc1ccc(cc1)CNc2ccc(c(c2)C#N)F
CC(C)Oc1ccccc1CNc2ccc(c(c2)C#N)F
C=CCOc1ccccc1CNc2ccc(c(c2)C#N)F
c1cc2c(cc1CNc3ccc(c(c3)C#N)F)CCO2
CC(C)Oc1cccc(c1)CNc2ccc(c(c2)C#N)F
CCc1ccccc1CNc2ccc(c(c2)CO)OCC
CC1(C=Cc2cc(ccc2O1)CC(=O)[O-])C
CC(=O)Nc1cccc(c1)C(=O)/C=C/c2cccc3c2OCCO3
```

```
Cc1cccc(c1)NC(=O)/C(=C/c2ccccc2OCc3ccc(cc3)F)/C#N
Cc1ccc(c(c1)NC(=O)/C(=C/c2ccccc2OCc3ccccc3F)/C#N)C
Cc1cccc(c1C)NC(=O)/C(=C\c2ccccc2OCc3ccccc3F)/C#N
Cc1ccc(cc1C)NC(=O)/C(=C/c2ccccc2OCc3ccccc3F)/C#N
CCc1ccc(cc1)NC(=O)/C(=C/c2ccccc2OCc3ccccc3F)/C#N
Cc1ccc(cc1C)NC(=O)/C(=C/c2ccccc2OCc3ccc(cc3)F)/C#N
Cc1cc(ccc1F)NCc2cccc(c2)OC(C)C
Cc1cc(ccc1F)NCc2ccc3c(c2)CCO3
Cc1cc(ccc1F)NCc2ccc(cc2)OC(C)C
Cc1cc(ccc1F)NCc2ccccc2OC(C)C
Cc1cc(ccc1F)NCc2ccccc2OCC=C
CCOc1cccc(c1OC)CNc2ccc(c(c2)C)F
Cc1cc(ccc1F)NCc2cccc3c2OCCO3
Cc1cc(ccc1F)NCc2cccc3c2OCCCO3
Cc1cc(ccc1F)NCc2ccccc2OCC#N
CCOc1ccccc1CNc2ccc(c(c2)C)F
CCOc1ccccc1[C@@H](C)Nc2ccc(c(c2)C)F
CCOc1ccccc1[C@H](C)Nc2ccc(c(c2)C)F
Cc1cc(ccc1F)NCc2ccc(cc2)OCC=C
Cc1cc(ccc1F)NCc2ccccc2OCC#C
Cc1cccc2c1O[C@@H](CN2)c3ccc(cc3)F
Cc1cccc2c1O[C@H](CN2)c3ccc(cc3)F
Cc1cc(c2c(c1)NC[C@@H](O2)c3ccc(cc3)F)C
Cc1cc(c2c(c1)NC[C@H](O2)c3ccc(cc3)F)C
c1cc2c(cc1NCc3cc(c(c(c3)F)F)F)CCO2
CCNc1ncc(cn1)C[NH+]2CCC[C@@]3(CC2)C=Cc4ccccc4O3
CCNc1ncc(cn1)C[NH+]2CCC[C@]3(CC2)C=Cc4ccccc4O3
CC(C)(C)c1ccc(cc1)CNc2ccc(cc2)OCc3ccccc3
COc1ccccc1CNc2ccc(cc2)OCc3ccccc3
CCOc1ccccc1CNc2ccc(cc2)OCc3ccccc3
CCCCOc1ccccc1CNc2ccc(cc2)OCc3ccccc3
```

```
CC(C)CCOc1ccccc1CNc2ccc(cc2)OCc3ccccc3
CCCCCOc1ccccc1CNc2ccc(cc2)OCc3ccccc3
CCCOc1ccccc1CNc2ccc(cc2)OCc3ccccc3
c1ccc(cc1)COc2ccc(cc2)NCc3ccccc3F
CC(C)Oc1ccccc1CNc2ccc(cc2)OCc3ccccc3
CC(C)Oc1cccc(c1)CNc2ccc(cc2)OCc3ccccc3
CC(C)c1ccc(cc1)CNc2ccc(cc2)OCc3ccccc3
c1ccc(cc1)CCOc2ccc(cc2)NCc3ccccc3F
CC(C)Oc1ccccc1CNc2ccc(cc2)OCCc3ccccc3
CCCCOc1ccccc1CNc2cccc(c2)OCc3ccccc3
CC(C)COc1ccccc1CNc2cccc(c2)OCc3ccccc3
CCCOc1ccccc1CNc2cccc(c2)OCc3ccccc3
c1ccc(cc1)COc2cccc(c2)NCc3ccccc3F
CC(C)Oc1ccccc1CNc2cccc(c2)OCc3ccccc3
c1ccc(cc1)COc2ccccc2NCc3ccccc3F
CC(C)Oc1cccc(c1)NCc2ccccc2F
CC[C@@H](C)Oc1ccc(cc1)NCc2ccccc2F
CC[C@H](C)Oc1ccc(cc1)NCc2ccccc2F
CC[C@@H](C)Oc1ccc(cc1)NCc2ccccc2OCCc3ccccc3
CC[C@H](C)Oc1ccc(cc1)NCc2ccccc2OCCc3ccccc3
CC(C)Oc1ccccc1CNc2ccc(c(c2)F)O
C=CCOc1ccccc1CNc2ccc(c(c2)F)O
Cc1ccc2c(c1)C(=C[C@@H](O2)c3ccccc3)Cl
Cc1ccc2c(c1)C(=C[C@H](O2)c3ccccc3)Cl
c1ccc2c(c1)C(=C[C@@H](O2)c3c(cccc3Cl)F)Cl
c1ccc2c(c1)C(=C[C@H](O2)c3c(cccc3Cl)F)Cl
c1ccc2c(c1)C(=C[C@@H](O2)c3cccc(c3)F)Cl
c1ccc2c(c1)C(=C[C@H](O2)c3cccc(c3)F)Cl
COc1cccc(c1)[C@@H]2C=C(c3ccccc3O2)Cl
COc1cccc(c1)[C@H]2C=C(c3ccccc3O2)Cl
c1ccc2c(c1)C(=C[C@@H](O2)c3cccc(c3)Cl)Cl
```

```
c1ccc2c(c1)C(=C[C@H](O2)c3cccc(c3)Cl)Cl
c1ccc2c(c1)C(=C[C@@H](O2)c3ccc(cc3)Cl)Cl
c1ccc2c(c1)C(=C[C@H](O2)c3ccc(cc3)Cl)Cl
c1ccc(c(c1)[C@@H]2C=C(c3ccccc3O2)Cl)Cl
c1ccc(c(c1)[C@H]2C=C(c3ccccc3O2)Cl)Cl
c1ccc(cc1)[C@@H]2C=C(c3ccccc3O2)Cl
c1ccc(cc1)[C@H]2C=C(c3ccccc3O2)Cl
CCOC1=C([C@@H](Oc2c1cccc2)c3ccc(cc3)C)CN(C)C
CCOC1=C([C@H](Oc2c1cccc2)c3ccc(cc3)C)CN(C)C
CCOC1=C([C@@H](Oc2c1cccc2)c3ccc(cc3)F)CN(C)C
CCOC1=C([C@H](Oc2c1cccc2)c3ccc(cc3)F)CN(C)C
CCOc1ccc(cc1CO)N[C@@H](C)c2c(cccc2F)F
CCOc1ccc(cc1CO)N[C@H](C)c2c(cccc2F)F
CCOc1ccc(cc1CO)N[C@@H](C)c2ccc(c(c2)F)F
CCOc1ccc(cc1CO)N[C@H](C)c2ccc(c(c2)F)F
CCOc1ccc(cc1CO)N[C@@H](C)c2ccc(cc2F)F
CCOc1ccc(cc1CO)N[C@H](C)c2ccc(cc2F)F
CCOc1ccc(cc1CO)N[C@@H](C)c2cc(ccc2F)F
CCOc1ccc(cc1CO)N[C@H](C)c2cc(ccc2F)F
Cc1ccc(cc1F)[C@H](C)Nc2ccc(c(c2)C)OC
CCOc1ccccc1/C=C/C(=O)NCc2cccc(c2)NC(=O)c3ccc(cc3)F
COc1cccc(c1)NCc2ccc(c(c2)OC)OCc3ccccc3F
COc1cccc(c1OCc2ccccc2)CNc3ccc(cc3)O
Cc1cccc(c1)COc2c(cccc2OC)CNc3ccc(cc3)O
CC(C)C(=O)Nc1ccc(cc1)CC(=O)N2CCC3(CC2)C=Cc4ccccc4O3
c1ccc(cc1)C(=O)Nc2ccc(cc2)CC(=O)N3CCC4(CC3)C=Cc5ccccc5O4
c1ccc(cc1)COc2cccc(c2)C(=O)N3CCC4(CC3)C=Cc5ccccc5O4
c1ccc(cc1)COc2ccccc2C(=O)N3CCC4(CC3)C=Cc5ccccc5O4
CN(C)c1cccc(c1)C(=O)N2CCC3(CC2)C=Cc4ccccc4O3
c1ccc2c(c1)C=CC3(O2)CCN(CC3)C(=O)c4ccc(cc4)F
c1ccc2c(c1)C=CC3(O2)CCN(CC3)C(=O)c4cccc(c4)F
```

```
c1ccc2c(c1)C=CC3(O2)CCN(CC3)C(=O)c4ccccc4F
Cc1cc(ccc1NC(=O)c2cccc(c2F)F)O[C@@H](C)c3ccccc3
Cc1cc(ccc1NC(=O)c2cccc(c2F)F)O[C@H](C)c3ccccc3
Cc1ccc(c(c1)C(=O)Nc2ccc(cc2C)O[C@@H](C)c3ccccc3)F
Cc1ccc(c(c1)C(=O)Nc2ccc(cc2C)O[C@H](C)c3ccccc3)F
C[C@@H](c1ccccc1F)Nc2ccc(cc2)OCc3cccc(c3)C#N
C[C@H](c1ccccc1F)Nc2ccc(cc2)OCc3cccc(c3)C#N
CCOc1ccc(cc1CO)N[C@@H](C)C(=O)NCc2ccccc2F
CCOc1ccc(cc1CO)N[C@H](C)C(=O)NCc2ccccc2F
CC1(Cc2cccc(c2O1)CNc3cccc(c3)CO)C
Cc1cc(ccc1F)NCc2cccc3c2OC(C3)(C)C
CCOc1ccc(cc1CCl)NCc2ccccc2F
CCOc1ccc(cc1CCl)NCc2c(cccc2Cl)F
CCOc1ccc(cc1CCl)NCc2ccc(cc2)F
CCOc1ccc(cc1CCl)NCc2cccc(c2F)F
CCOc1ccc(cc1CCl)NCc2cc(ccc2F)F
CCOc1ccc(cc1CCl)NCc2cccc(c2)F
CCOc1ccc(cc1CCl)NCc2ccc(cc2F)F
COCc1cccc(c1)CNc2ccc3c(c2)CCO3
COCc1ccccc1CNc2ccc3c(c2)CCO3
Cc1cc(ccc1OC)NCc2ccccc2COC
COCc1ccc(cc1)CNc2ccc3c(c2)CCO3
C=CCc1cc(ccc1OCc2ccccc2F)N
c1ccc2c(c1)C=C(CO2)CNc3ccc(cc3)Cl
C[C@@H](c1c(cc(cc1F)OC)F)Nc2cccc(c2)CO
C[C@H](c1c(cc(cc1F)OC)F)Nc2cccc(c2)CO
Cc1cc(cc(c1)F)NCc2ccccc2OC(C)C
Cc1cc(cc(c1)F)NCc2ccc(cc2)OCC=C
Cc1cc(cc(c1)F)NCc2ccccc2OCC=C
c1cc2c(cc1NCc3cc(c(c(c3)F)O)F)CCO2
c1cc(ccc1CNc2ccc3c(c2)CCO3)C4CC4
```

```
Cc1cc(c(cc1F)[C@@H](C)Nc2ccc3c(c2)CCO3)F
Cc1cc(c(cc1F)[C@H](C)Nc2ccc3c(c2)CCO3)F
Cc1cc(ccc1OC)N[C@@H](C)c2cc(c(cc2F)C)F
Cc1cc(ccc1OC)N[C@H](C)c2cc(c(cc2F)C)F
C[C@@H](c1cc(cc(c1)F)F)Nc2ccc3c(c2)CCO3
C[C@H](c1cc(cc(c1)F)F)Nc2ccc3c(c2)CCO3
```
